# Evaluation of bone health and fracture risk in type 2 diabetes: a network meta-analysis of anti-diabetic treatments versus placebo

**DOI:** 10.1007/s12272-025-01552-2

**Published:** 2025-06-20

**Authors:** SuA Oh, Se-eun Park, Eunyoung Kim

**Affiliations:** 1 Data Science, Evidence-Based and Clinical Research Laboratory, Department of Health, Social and Clinical Pharmacy, College of Pharmacy, Chung-Ang University, Seoul, 06974, Republic of Korea; 2 Department of Internal Medicine, Division of Endocrinology and Metabolism, Kangbuk Samsung Hospital, Sungkyunkwan University School of Medicine, Seoul, 03063 Korea; 3 Central Research Centre of Epigenome based platform and its Application for Drug Development, College of Pharmacy, Chung-Ang University, Seoul, Republic of Korea; 4 The Graduate School of Pharmaceutical Industry Management, Chung-Ang University, Seoul, Republic of Korea; 5 Department of Pharmaceutical Regulatory Sciences, Chung-Ang University, Seoul, Republic of Korea

**Keywords:** Network meta-analysis, Type 2 diabetes, RCT, Anti-diabetic, Fracture, Placebo

## Abstract

While studies have highlighted the negative effects of certain antidiabetic agents, similar evidence for other antidiabetic agents remains limited. In this study, we aimed to analyze bone fractures and bone mineral densities (BMDs) across patients receiving antidiabetic treatments, considering both overall and specific sites. We comprehensively searched PubMed, Embase, and ClinicalTrials.gov up to March 2024 to determine the effect of antidiabetic agent use on bone health in patients with type 2 diabetes mellitus. The primary outcome was to reveal variations in fractures across different anti-diabetic treatment modalities. The secondary outcome was the differences in BMDs based on treatment type. The fractures were grouped based on the division of specific sites. We also performed a subgroup analysis to identify differences between treatment types by dividing the study by treatment duration. The protocol is registered (CRD42024538789). A total of 234,759 individuals were enrolled in the 242 studies. We observed a trend wherein all anti-diabetic treatments were associated with decreased risk of fracture compared with placebo; however, this was not significant in direct analysis (OR 0.92, 95% CI 0.84–1.01, I^2^ = 0, P = 0.07). For indirect analysis, glucagon-like peptide-1 receptor agonists (GLP1RAs) demonstrated a significant effect on preventing fractures in non-vertebral fracture, hip fracture, vertebral and hip fracture, and overall fracture (odds ratio [OR] 0.62, 95% confidence interval [CI] 0.43–0.90; OR 0.67, 95% CI 0.49–0.92; OR 0.64, 95% CI 0.47–0.87; OR 0.58, 95% CI 0.48–0.69, respectively). Additionally, dipeptidyl peptidase-4 inhibitors (DPP4i) significantly reduced incidences of non-vertebral fracture, vertebral and hip fracture, and overall fracture risk (OR 0.34, 95% CI 0.25–0.45; OR 0.72, 95% CI 0.55–0.95; OR 0.67, 95% CI 0.55–0.82, respectively). Meanwhile, metformin also reduces overall fracture risk (OR 0.60, 95% CI 0.42–0.88). We observed no significant differences between the antidiabetic agents according to the specific fracture site or study time point. Most antidiabetic treatments except thiazolidinediones did not increase the risk of fractures compared with the placebo. Incretin-based therapies (GLP1RA and DPP4i) exerted beneficial effects on fracture prevention compared with other treatments. These findings underscore the need for well-conducted RCTs to provide further evidence.

**Supplementary Information:**

The online version contains supplementary material available at 10.1007/s12272-025-01552-2.

## Introduction

Type 2 diabetes mellitus (T2DM) and osteoporosis are increasingly prevalent diseases among older adults. Epidemiological studies have revealed a notably high prevalence of osteoporosis among patients with T2DM, which is estimated to be 27.67% (Liu et al. 2023). The high prevalence and mortality rates among patients with T2DM and osteoporosis impose a significant health burden on society.

Patients with T2DM exhibit elevated bone mineral density (BMD); however, they also possess a heightened risk of bone fractures. In a retrospective cohort study, a fully adjusted multivariate analysis revealed statistically significant increases in hazard ratios (HRs) at follow-up intervals of 1, 3, and 5 years (Guo et al. 2025). Furthermore, several previous studies have reported a correlation between diabetes and an increased risk of fractures (van Hulten et al. 2024; Yamamoto et al. 2009). Advanced glycation end-products (AGEs) contribute to the incidence of fractures in patients with T2DM. AGEs are formed when glucose in the blood reacts with proteins such as hemoglobin, and they accumulate in the body, contributing to the development of diseases such as diabetes complications and osteoporosis (Twarda-Clapa et al. 2022; Saito and Marumo 2010). In addition, chronic low-grade inflammation, which is frequently observed in diabetic patients, exacerbates bone resorption and suppresses bone formation, thereby negatively affecting overall bone health (Napoli et al. 2017). Moreover, metabolic disturbances, such as impaired calcium and vitamin D metabolism associated with insulin resistance, have been linked to deterioration in bone quality and increased susceptibility to fractures in T2DM patients (Wongdee et al. 2017). Thus, patients with T2DM experience complex and multifaceted risks of osteoporosis and fractures beyond the accelerated formation of AGEs alone. Therefore, preventive and therapeutic strategies against osteoporosis in patients with T2DM are needed.

The antidiabetic agents commonly used in the treatment of type 2 diabetic patients have been used for an extended period. These include sodium-glucose cotransporter 2 inhibitors (SGLT2i), glucagon-like peptide-1 receptor agonists (GLP1RA), metformin, dipeptidyl peptidase 4 inhibitors (DPP4i), thiazolidinediones, sulfonylureas, and insulin. A correlation between diabetes treatment and bone metastasis continues to be reported. In a previous meta-analysis, anti-diabetic agents were compared with placebo for overall fractures (Zhang et al. 2021a; Monami et al. 2011). However, thiazolidinediones have been shown to cause a higher incidence of bone fractures in the extremities (Meier et al. 2008; Valderrábano and Linares 2018). Therefore, the analysis of specific fracture sites is important, and there are reports that certain diabetes treatments have negative effects on bones.

Previous studies on the effects of diabetes medications on the bone have been heterogeneous. Previous studies have reported that the osmotic diuretic effect of SGLT2 inhibitors can lead to volume depletion and electrolyte imbalances, and potential changes in calcium and phosphate can adversely affect bone health (Garber et al. 2016; Londzin et al. 2022). Some studies found that SGLT2i were associated with a non-significant decrease in bone fracture risk (OR 1.18, 95% CI 0.58 2.41). On the other hand, other study reported SGLT2i had effect to prevent fracture (OR 0.31, 95% CI 0.11 to 0.89) (Cheng et al. 2019). Meta-analyses have also been conducted; however, their conclusions have been controversial. In addition, previous studies have reported the efficacy of only specific antidiabetic agents in the treatment of T2DM (Wang et al. 2023); most studies have only reported overall fracture events (Chai et al. 2022).

Therefore, we aimed to ascertain the specific impact of anti-diabetic medications on the fracture risk and BMD in patients with T2DM by conducting a systematic review and meta-analysis was conducted using all available RCTs. We classified the specific fracture sites into treatment- and skeleton-based classifications. A subgroup analysis was also conducted according to the study period. We performed a direct meta-analysis to determine the differences between all anti-diabetic treatments and the placebo. We then determined the relative differences between the anti-diabetic treatments and conducted an indirect meta-analysis.

## Materials and methods

The study adhered to the procedures outlined in the Preferred Reporting Items for Systematic Reviews and Meta-Analyses (PRISMA) framework. (Supplementary Table 1) (Page et al. 2021). Additionally, the study protocol was officially registered in the PROSPERO database under the registration number CRD42024538789.

### Data sources and searches

Until March 2024, two researchers conducted independent searches of PubMed, EMBASE, and ClinicalTrials.gov, using each database from its inception. A detailed search query is presented in Supplementary Table 2. Studies evaluating the effects of fractures or bone in patients with T2DM were the aim of the search approach. An emphasis was placed on English language studies in the search. References from the selected articles and systematic reviews were manually screened to identify relevant studies. Any discrepancies between the investigators were resolved through discussion and consensus.

### Study selection

This study included only randomized controlled trials (RCTs) in adult subjects with T2DM who received treatment with anti-diabetic agents. Pre- and postoperative data on the incidence of one or more outcomes linked to fracture or bone mineral density (BMD) were considered. Reviews, letters, commentaries, case reports, single-arm studies, conference abstracts, preclinical, phase 1–2 trials, pediatric studies, and studies unrelated to antidiabetic treatments were excluded. Furthermore, studies that focused on combinations of different antidiabetic agents were excluded. Our study, which did not include NCT and/or register numbers, was excluded for clarity.

### Data extraction and quality assessment

Data were gathered from the studies, including various characteristics, such as publication year, first author, registration number, disease type, intervention details, sample size, mean age, percentage of male participants, study duration, and study design. Additionally, pre- and post-intervention data and the incidence rates of BMD or fractures were extracted from the retrieved studies. Two investigators independently conducted the data extraction. The study was re-evaluated for discrepancies, and a consensus was achieved through discussion. The follow-up durations were 52, 78, and 104 weeks, and the fracture risk prevention effects of different drug types were compared at the three time points. These time points were selected because they demonstrated significant effects with existing oral osteoporosis drugs (Black et al. 2000). We identified fractures in two classifications: first, the treatment-based classification was vertebral, non-vertebral fracture, and hip fracture according to osteoporosis treatment guidelines (Morin et al. 2023). Vertebral fractures involve one or more vertebrae. A nonvertebral fracture is defined as a fracture that does not occur in the spine and thus excludes skull fractures (Waterloo et al. 2012). Hip fractures are proximal to femoral fractures. Second, we analyzed the vertebral column, thoracic cage, cranial and facial regions, pelvic girdle, upper extremities, and lower extremities. The classification was based on the anatomy and the National Institutes of Health (NIH) division of the skeleton (NIH, Anderson et al. 2024). We added the collected vertebral and hip or multiple fractures to investigate various fracture sites.

The risk of bias (ROB) assessment tool version 2.0, developed by the Cochrane Collaboration, was used to evaluate the quality of the RCTs (Sterne et al. 2019). Evidence quality was assessed using the Grading of Recommendations, Assessment, Development, and Evaluation (GRADE) approach, which considers study limitations, inconsistency, indirectness, imprecision, and publication bias. The GRADE assessment categorizes evidence quality as high, moderate, low, or very low (Howard Balshem 2011).

### Data synthesis and analysis

The primary outcome was the difference in fracture risk between the anti-diabetic treatment groups. Treatment options include glucagon-like peptide-1 receptor agonists (GLP1RA), sodium-glucose cotransporter-2 inhibitors (SGLT2i), thiazolidinediones, metformin, sulfonylurea, insulin, and dipeptidyl peptidase-4 inhibitors (DPP4i). Fractures were divided into two classifications: treatment-based and anatomy-based fractures. The secondary outcome assessed was the difference in bone mineral density (BMD) based on the type of treatment administered. A subgroup analysis was performed to identify differences between treatment types by dividing the study by duration. In the case of direct analysis, the studies selected for analysis were those with a placebo as the control group and those that included intervention in the control group.

R Studio (version 4.1.2) was used to compute the total effect size of the studies, which was expressed as the standardized mean difference (SMD), odds ratio (OR), and 95% confidence interval (CI). Statistical significance was set at p < 0.05; statistical significance was established. The significance of heterogeneity across studies, defined as 25%, 50%, and 75%, indicated moderate, medium, and high heterogeneity, respectively, and was assessed using ${\text{I}}^{2}$ statistics (Higgins et al. 2003). Publication bias was evaluated using the Egger’s test of funnel plot (Egger et al. 1997). Sensitivity analysis was conducted, wherein studies with the highest number of participants were excluded. A meta-regression analysis was performed.

We used the Bayesian fixed-effects model to incorporate the estimates of the direct and indirect treatment comparisons and ranked the interventions in order. The deviance information criterion was also used to select between a fixed- and random-effects model (Supplementary Table 4) (Béliveau et al. 2019). The Markov chain Monte Carlo method was used to obtain results from the aggregate data. We calculated the relative ranking of interventions for efficacy as their surface under the cumulative ranking (SUCRA), with higher SUCRA scores corresponding to a higher ranking for efficacy. SUCRA is based solely on the point estimates and standard errors of the network estimates. It measures the mean extent of the network estimates and the mean extent of certainty that one intervention is superior to another and is averaged over all competing interventions (Rücker and Schwarzer 2015). Furthermore, we measured the heterogeneity of indirect meta-analyses.

## Results

### Study and patient characteristics

**Fig. 1:**
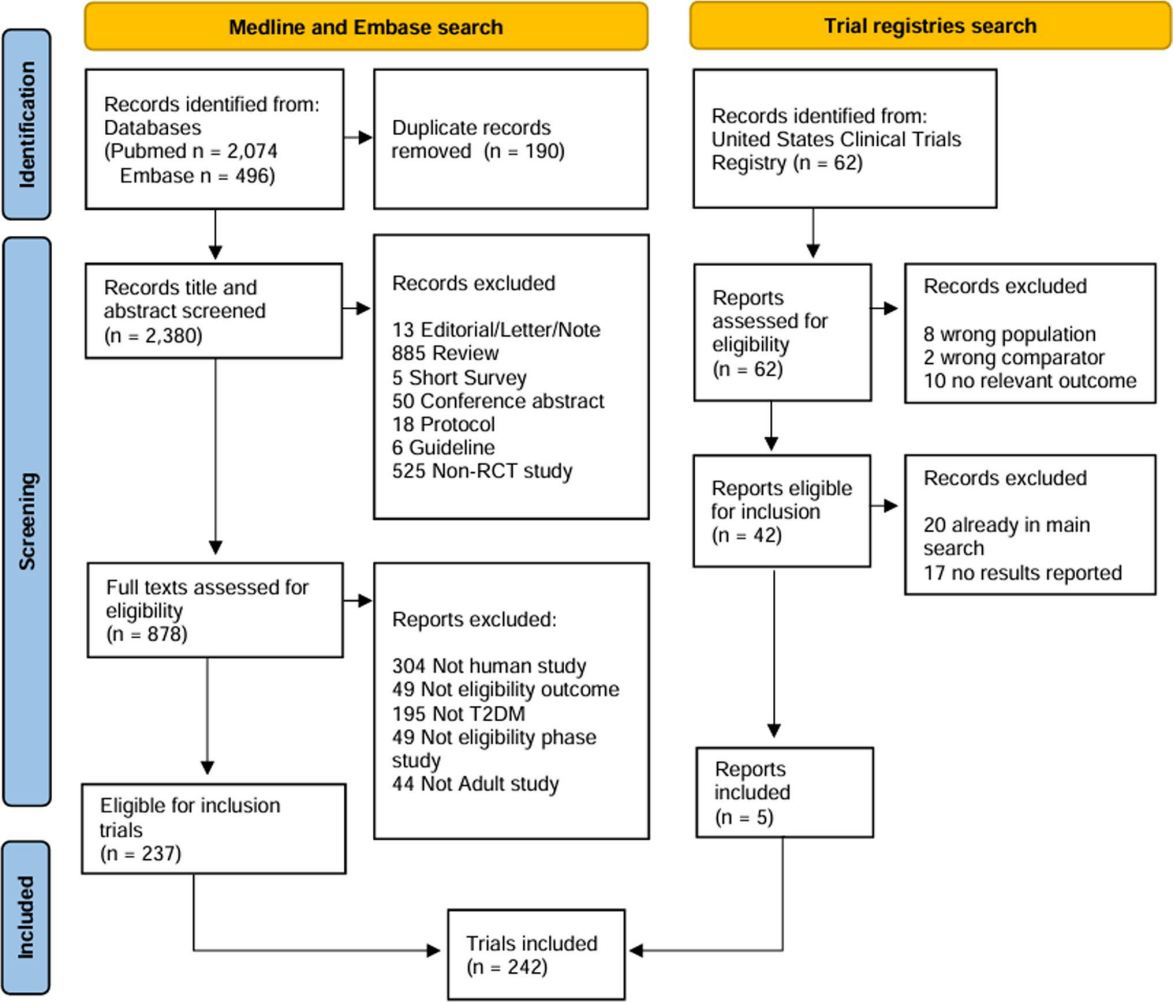
Flow diagram of the study selection process. This diagram illustrates the identification, screening, eligibility assessment, and inclusion stages of the included studies. The reasons for exclusion at each step and the final number of studies included in the analysis were clearly indicated

**Fig. 2:**
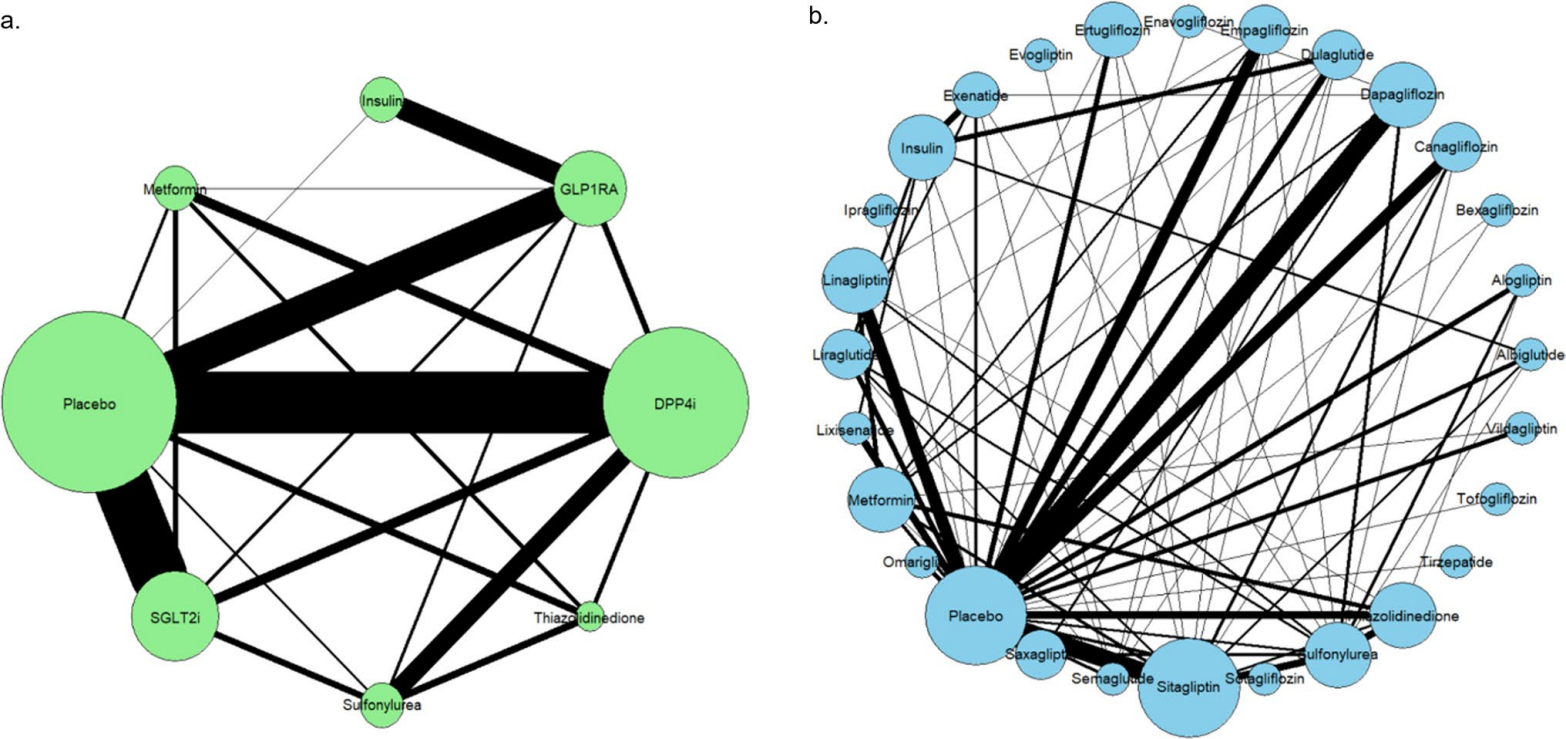
Network plot for the overall fracture. **a** Fracture of anti-diabetic agents type; **b** Fracture of specific anti-diabetic agents; **a** Network plot representing individual anti-diabetic agents. Each node represents a specific anti-diabetic agent, and node size reflects the number of participants assigned to the respective treatment. Edges (lines) between nodes indicate direct comparisons between treatments, with line thickness proportional to the number of studies directly comparing each pair.; **b** Network plot grouped by anti-diabetic agent types. Each node represents an anti-diabetic agent class, with node size proportional to the total number of participants. Lines connecting nodes indicate available direct comparisons, with thickness corresponding to the number of trials for each comparison

A thorough search yielded 2632 records, of which 190 duplicates were excluded. The screening removed 1502 studies that did not meet the inclusion criteria. After full-text assessment of the 936 articles, 242 randomized controlled trials (RCTs) were included. The included studies involved a total of 234,759 participants with type 2 diabetes mellitus (T2DM) (Fig. 1). Figure 2 and Supplementary Figs. 1–6 present the network graphs, while Supplementary Table 3 summarizes the included studies' baseline characteristics.

### Effect of anti-diabetic agents on overall fracture

**Fig. 3:**
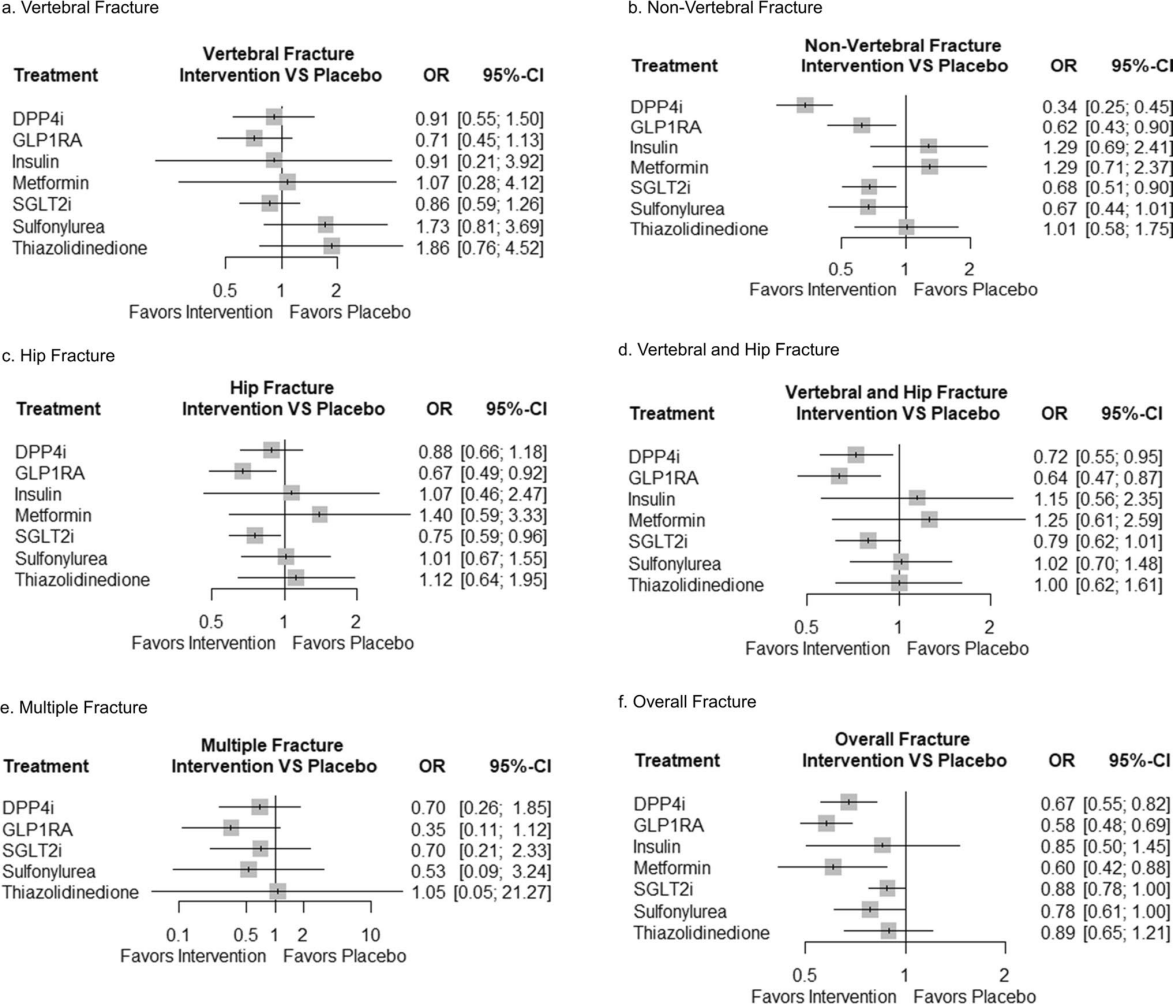
Forest plot of network meta-analysis results for site of fracture between type of anti-diabetic agents. **a** Vertebral Fracture **b** Non-Vertebral Fracture **c** Hip Fracture **d** Vertebral and Hip Fracture **e** Multiple fractures **f** Overall Fracture; Results are presented as odds risks (ORs) with 95% confidence intervals (CIs). Treatments were ranked according to their relative effectiveness profiles compared with those of the reference treatment. The plots depict specific fracture sites. Vertical dashed lines indicate the reference line for the placebo (OR = 1). Treatments with credible intervals that did not cross the reference line indicated significant differences in fracture risk compared with the reference treatment

This outcome included 137 trials, including 156,117 participants with overall fractures. The anti-diabetic agents effectively reduced fracture risk, except thiazolidinedione (OR 0.92, 95% CI 0.84–1.01, I^2^ = 0, P = 0.07). In addition, the comparison of groups based on antidiabetic agent type revealed no significant differences between the various antidiabetic agents (Table 1). For indirect analysis, GLP1RA, DPP4i, and metformin prevented significantly in overall fracture (OR 0.58, 95% CI 0.48- 0.69, SUCRA 0.91; OR 0.67, 95% CI 0.55–0.82, SUCRA 0.76; OR 0.60, 95% CI 0.42–0.88, SUCRA 0.73; respectively) (Supplementary Table 40–41). Detailed indirect comparisons of the efficacy of fractures between individual drugs and interventions are presented in Supplementary Tables 9–10, Fig. 3, and Supplementary Fig. 7. In the subgroup analysis to determine the differences between treatment types by dividing the study by period, no significant differences were observed (Supplementary Table 37–39).

**Table 1:** Effect of intervention compared placebo in meta-analysis results

Outcome	Total Effect	P-value	Heterogeneity	Subgroup difference	GLP1RA	SGLT2i	DPP4i	Metformin	TZD
Fracture | OR [95% CI]									
Overall Fracture	0.92 [0.84, 1.01]	0.07	0%	0.68	0.84 [0.69, 1.02]	0.97 [0.84, 1.12]	0.87 [0.71, 1.06]	0.96 [0.09,10.73]	1.33 [0.54, 3.29]
Vertebral Fracture	0.81 [0.62, 1.04]	0.10	0%	0.82	0.68 [0.43, 1.10]	0.86 [0.59, 1.26]	0.88 [0.52, 1.50]	NA	0.41 [0.03, 6.66]
Non-Vertebral Fracture	0.95 [0.85, 1.06]	0.34	0%	0.43	0.83 [0.67, 1.01]	1.03 [0.88, 1.21]	0.95 [0.78, 1.17]	NA	1.09 [0.36, 3.30]
Treatment based Hip Fracture	0.93 [0.80, 1.09]	0.36	0%	0.94	0.89 [0.67, 1.19]	0.91 [0.71, 1.16]	1.01 [0.76, 1.34]	NA	0.87 [0.11, 6.77]
Vertebral + Hip Fracture	0.89 [0.78, 1.02]	0.10	0%	0.80	0.82 [0.65, 1.05]	0.90 [0.73, 1.10]	0.98 [0.76, 1.25]	NA	0.67 [0.13, 3.49]
Multiple Fracture	0.85 [0.45, 1.62]	0.85	0%	0.79	1.19 [0.36, 3.96]	0.83 [0.24, 2.85]	0.70 [0.26, 1.85]	NA	NA
Anatomy Fracture | OR [95% CI]									
Vertebral Column Fracture	0.78 [0.58, 1.05]	0.11	0%	0.68	0.64 [0.39, 1.05]	0.82 [0.48, 1.37]	0.98 [0.57, 1.67]	NA	NA
Lower Extremity Fracture	0.96 [0.85, 1.08]	0.51	0%	0.63	0.84 [0.67, 1.06]	1.00 [0.82, 1.22]	1.01 [0.80, 1.26]	NA	1.19 [0.38, 3.74]
Upper Extremity Fracture	0.90 [0.77, 1.06]	0.20	0%	0.81	0.83 [0.62, 1.11]	0.98 [0.77, 1.25]	0.84 [0.60, 1.19]	NA	0.71 [0.09, 5.37]
Pelvic Girdle Fracture	0.91 [0.71, 1.16]	0.45	0%	0.92	1.01 [0.64, 1.61]	0.83 [0.56, 1.21]	0.94 [0.59, 1.50]	NA	0.87 [0.11, 6.77]
Cranial and Facial Fracture	1.01 [0.70, 1.47]	0.95	0%	0.42	0.84 [0.40, 1.75]	1.52 [0.83, 2.80]	0.79 [0.43, 1.45]	NA	0.51 [0.03, 8.18]
Thoracic Cage Fracture	0.87 [0.67, 1.13]	0.28	0%	0.63	0.74 [0.45, 1.23]	1.08 [0.71, 1.63]	0.78 [0.47, 1.32]	NA	0.54 [0.06, 4.96]
BMD | SMD [95% CI]									
Femoral neck BMD	0.02 [-0.07, 0.12]	0.64	0%	0.87	NA	0.03 [-0.09, 0.16]	NA	0.00 [-0.19, 0.19]	-0.10 [-0.62, 0.43]
Total hip BMD	-0.12 [-1.09,0.85]	< 0.01	98%	< 0.01	2.50 [1.81, 3.18]	0.00 [-0.15, 0.16]	NA	-2.74 [-3.01, -2.47]	-0.35 [-0.77, 0.07]
Lumber spine BMD	0.06 [-0.03, 0.16]	0.21	21%	0.22	NA	0.04 [-0.08, 0.15]	NA	0.00 [-0.19, 0.19]	0.49 [-0.03, 1.01]

*BMD* Bone Mineral Density, *DPP4i* dipeptidyl peptidase 4 inhibitor, *GLP1RA* Glucagon-like-peptide-1 receptor agonists, *NA* not applicable, *OR* Odds ratio, *SGLT2i* Sodium-glucose cotransporter 2 inhibitor

### Effect of anti-diabetic agents on the fracture site

In the treatment-based fracture site analysis, most anti-diabetic agents prevented the placebo in five fracture groups and multiple fracture risks. The classification of the specific fracture sites was not significantly different (Table 1). According to SUCRA probabilities, most results showed that GLP1RA and DPP4i, compared with placebo, tended to be effective in preventing fracture risk (Supplementary Table 40–41). The GLP1RA had a significant effect on the prevention of non-vertebral fractures, hip fractures, and vertebral and hip fractures (OR 0.62, 95% CI 0.43–0.90; OR 0.67, 95% CI 0.49–0.92; OR 0.64, 95% CI 0.47–0.87, respectively). DPP4i significantly reduced fracture risk in non-vertebral and vertebral and hip fractures (OR 0.34, 95% CI 0.25–0.45; OR 0.72, 95% CI 0.55–0.95, respectively) (Fig. 3).

**Fig. 4:**
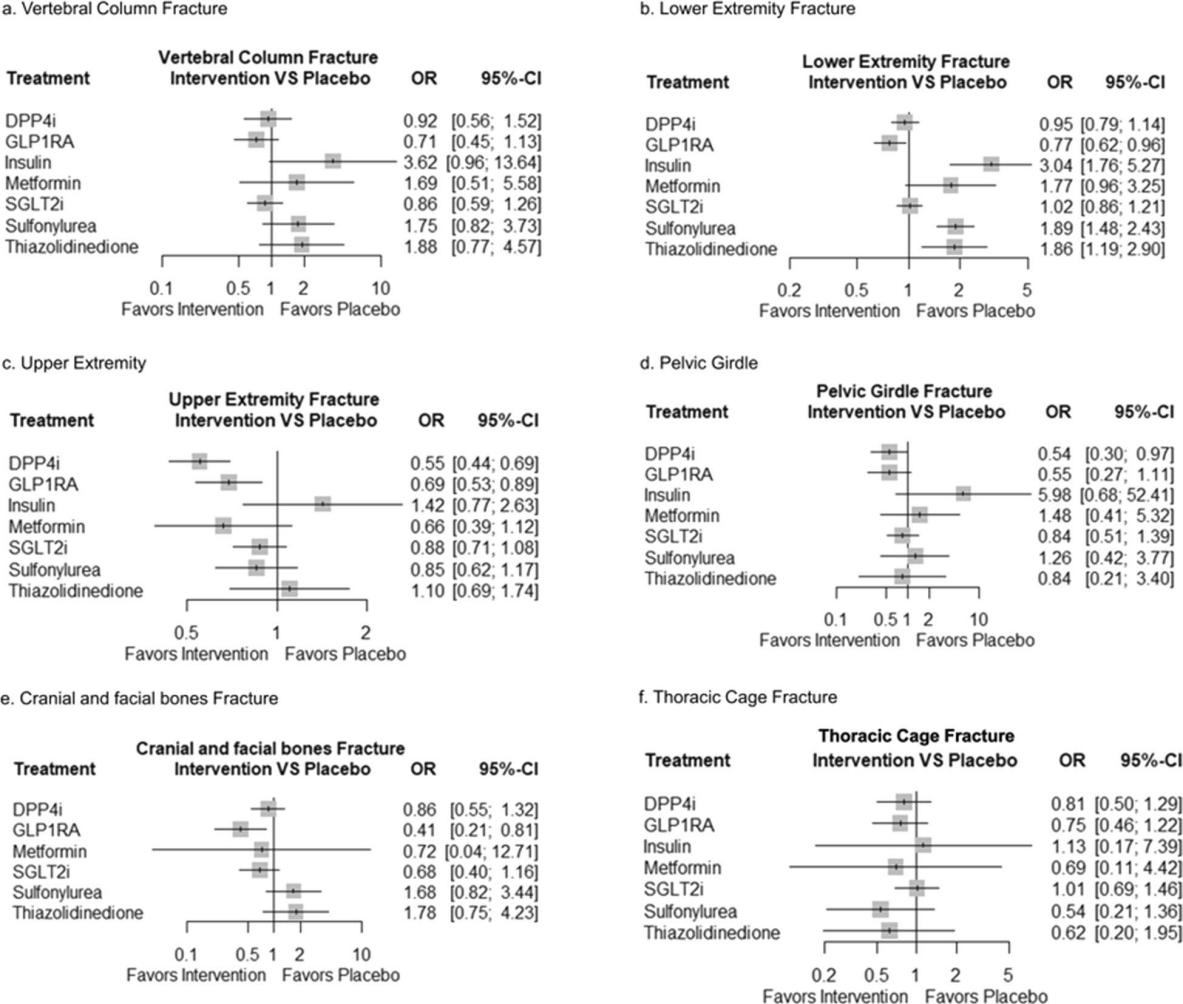
Forest plot of network meta-analysis results for anatomy fracture site between type of anti-diabetic agents. **a** Vertebral Column fracture **b** Lower Extremity Fracture, **c** Upper Extremity fracture, **d** Pelvic Girdle fracture, **e** Cranial and facial bone fracture **f** Thoracic Cage Fracture; Results are presented as odds ratios (ORs) with 95% confidence intervals (CIs). Treatments were ranked according to their relative effectiveness profiles compared with those of the reference treatment. The plots depict specific fracture sites. Vertical dashed lines indicate the reference line for the placebo (OR = 1). Treatments with credible intervals that did not cross the reference line indicated significant differences in fracture risk compared with the reference treatment

GLP1RA was the best in direct or indirect analyses for classifying anatomical fracture sites. GLP1RA reduced fracture risk in the lower extremity, cranial and facial, and upper extremity compared to placebo (OR 0.77, 95% CI 0.62–0.96; OR 0.41, 95% CI 0.21–0.81; OR 0.69, 95% CI 0.53–0.89, respectively). DPP4i also prevented the fracture risk of upper extremity fractures and pelvic girdle (OR 0.55, 95% CI 0.44–0.69; OR 0.54, 95% CI 0.30–0.97, respectively). Thiazolidinedione, sulfonylurea, and insulin increased the lower extremity fracture (OR 1.86, 95% CI 1.19–2.90; OR 1.89, 95% CI 1.48–2.43; OR 3.04, 95% CI 1.76–5.27, respectively) (Fig. 4). In the direct analysis, overall diabetes treatment tended to prevent fracture risk compared with the placebo, but the difference was not significant (Table 1).

Supplementary Table 11–30 and Supplementary Fig. 7–8 present a comprehensive indirect comparison of the effectiveness of various anti-diabetic agents in reducing fractures and a comparison of specific anti-diabetic agents. Moreover, no discernible alterations were observed in the subgroup analyses at any study time points (52, 78, or 104 weeks). (Supplementary Table 37–39).

### Effect of anti-diabetic agents on bone mineral density

The analysis of the nine RCTs included 1,777 participants with BMD of the femoral neck, total hip, or lumbar spine. The femoral neck and lumbar spine interventions were effective than placebo, but not significantly (SMD 0.02, 95% CI − 0.07–0.12, I^2^ = 0, P = 0.64; SMD 0.06, 95% CI − 0.03–0.16, I^2^ = 21%, P = 0.21, respectively). Moreover, intervention to the total hip decreased BMD compared to placebo (SMD − 0.12, 95% CI − 1.09–0.85, I^2^ = 98%, P < 0.01) (Table 1). In the indirect analysis, the GLP1RA (SUCRA 1) ranked highest in the total hip, followed by placebo (SUCRA 0.49), SGLT2i (SUCRA 0.49), metformin (SUCRA 0.2), and thiazolidinedione (0.00). The lumbar spine ranked best for metformin (SUCRA 0.98) and worst for thiazolidinedione (SUCRA 0). For the femoral neck, SGLT2i (SUCA 0.87) was the best, and metformin (SUCA 0.00) was the worst (Supplementary Table 42–43). Additional information, including comprehensive indirect comparison of efficacy and detailed anti-diabetic agents’ indirect analysis, is reported in Supplementary Table 31–36 and Supplementary Fig. 9–10. However, the interpretation of BMD results should be approached with caution owing to the inclusion of only a few trials.

### Quality assessment

The risk of bias (ROB) assessment of RCTs provided a framework for evaluating the quality of the included studies. This assessment considered several aspects of the research, such as randomization, allocation concealment, blinding, handling of missing data, and potential selection bias. The quality of the included studies was mainly low risk or of some concern, except for six studies. No information was reported on the ClinicalTrial.gov data (Supplementary Fig. 17–18). Table S42 presents the quality of evidence obtained using the GRADEpro method to evaluate the effects of anti-diabetic agents on the specified outcome. The results were consistent, with agreement between direct and indirect comparisons using global approaches for all included outcomes (Supplementary Table 5–8). Additionally, a funnel plot of the included studies, depicted in Supplementary Table 40 and Supplementary Fig. 11–16, did not exhibit any asymmetry, suggesting that a significant publication bias was unlikely. In the context of sensitivity analysis, the study with the largest number of patients was excluded, which did not affect the results (Supplementary Table 41). The results of the meta-regression analysis were not significant (Supplementary Table 43).

## Discussion

In this systematic review and meta-analysis, we employed direct and indirect evidence to compare anti-diabetic drug alternatives for bone health in patients with T2DM. Our analysis yields two main findings. First, in the direct analysis, any anti-diabetic agent tended to reduce the fracture risk in patients with T2DM. Second, in indirect analysis, GLP1RAs or DPP4i may be effective agents for reducing the overall and specific fracture risk among anti-diabetic agents.

Anti-diabetic agents tended to be effective in preventing overall fracture risk (P = 0.07). The findings of our investigation are consistent with those of a meta-analysis of previous research that found that the use of DPP4i, GLP1RA, or SGLT2i reduced fracture risk compared to placebo (Shen et al. 2024). Conversely, existing meta-analyses have indicated that anti-diabetic agents may significantly reduce the risk of fractures compared to a control group (Zhang et al. 2019); however, the number of included studies was small. In most of the included studies, fracture incidence was identified as an adverse event, and this study was not significant because of the low incidence; however, the intervention tended to be protective compared to placebo.

GLP1RA is associated with a decreased risk of overall and specific fracture sites. GLP-1 decreases bone resorption and promotes new bone creation. GLP-1 promotes osteoblast growth, expression of bone-forming genes, and serum levels of markers associated with bone production. The effects of GLP-1 may be mediated through the Wnt pathway, c-Fos transcriptional stimulation, or mitogen-activated protein kinase pathways. GLP-1 may bind to its receptor on osteoblasts, shifting the development of mesenchymal stem cells from adipocytes to osteoblasts (Nuche-Berenguer et al. 2010). In addition, GLP-1 has been observed to decrease the expression of the receptor activator for nuclear factor-κB ligand while simultaneously boosting OPG expression (McClung 2007). This mechanism allows GLP1RA to exert a direct or indirect influence on the bone and can lead to the prevention of fractures and an increase in BMD. Animal studies have shown that GLP1RA improves bone quality. In aged rats with osteoporosis resulting from ovariectomy, exendin-4 demonstrated the capacity to reinforce bone tissue while avoiding adverse effects on trabecular microarchitecture (Nuche-Berenguer et al. 2011). GLP1RA reduced the risk of extremity, cranial, and facial fractures; however, the specific mechanism involved was not revealed. Given that GLP1RA, which has a weight loss effect, is likely to be used by relatively young people, future research is required to correct these data by age. In this study, GLP1RA improved BMD. However, the number of individual bone mineral density studies included in this study was very small, so interpretations must be made cautiously, and future analyses, including large-scale studies, are needed.

DPP4i prevented certain fracture risks in treatment-based, anatomy-based, and overall fracture sites. DPP4i has been demonstrated to enhance glucose tolerance in diabetic individuals whose bone formation is compromised by insulin deficiency and hyperglycemia. This is achieved by extending the half-lives of GLP-1 and GIP and stimulating insulin production from β-pancreatic cells (Idris and Donnelly 2007). Furthermore, DPP4i exerts its effects on bone metabolism via a mechanism connected to vitamin D. The administration of DPP4i resulted in a significant elevation in serum 25(OH)-D concentrations, which in turn leads to an increase in bone development and remodeling (Barchetta et al. 2016). DPP4i expression is decreased via this pathway to significantly mitigate the deleterious effects of elevated glycemic levels on bone health. The results of this study corroborate those of other studies suggesting that DPP4i and GLP1RA effectively reduce the fracture risk (Dombrowski et al. 2017).

Incretin-based therapies, including GLP-1RA and DPP4i, have shown promising effects on bone health, particularly in patients with T2DM (Mabilleau 2017). These therapeutic agents affect bone metabolism through multiple molecular pathways. GLP-1RAs, for instance, have been reported to elevate the serum levels of osteocalcin, a key marker of bone formation secreted by osteoblasts. This increase in osteocalcin is associated with improved bone mass and mechanical strength (Sedky 2021). On the other hand, DPP4 inhibitors, by extending the action of GLP-1 through the inhibition of DPP-4 activity, may exert beneficial effects on the bone, similar to those observed with GLP-1 (Glorie et al. 2014). In addition, incretin-based therapies have been reported to suppress inflammation-induced osteoclast differentiation and bone resorption (Kitaura et al. 2021). Despite these promising findings, large-scale well-designed clinical studies are required to evaluate the long-term effects of incretin therapy on bone mineral density, fracture risk, and overall bone health in patients with T2DM. Recently, a meta-analysis has been published evaluating the effects of GLP-1 receptor agonists (GLP-1RAs) on fracture risk. Zhang et al. (2025) demonstrated that GLP-1RA treatment was associated with a significantly reduced fracture risk in patients with type 2 diabetes mellitus (RR 0.77; 95% CI: 0.61–0.96). The protective effect was particularly evident with longer treatment durations (> 78 weeks). These findings are consistent with our results and support the potential clinical relevance of GLP-1RAs in patients with high fracture risk (Zhang et al. 2025).

Overall, metformin and SGLT2i had neutral effects. In bone metabolism, metformin exerts its effects primarily by inhibiting mitochondrial respiratory chain complex I and activating adenosine 5'-monophosphate-activated protein kinase. Furthermore, metformin may benefit bone via a mechanism that controls the expression of genes linked to bone formation by upregulating the expression of sirtuin protein and runt-related transcription factor 2 (Molinuevo et al. 2010). The results indicated that metformin demonstrated differences in the overall and indirect analysis of fractures by region. This discrepancy is likely attributable to differences in the number of studies and patients included in the indirect analysis of all fractures. SGLT2 inhibitors can indirectly increase bone turnover through weight loss and improve bone metabolism disorders in diabetes by reducing blood sugar levels. However, compared to DPP4i and GLP1RA, SGLT2i pose a risk of hypoglycemia and fractures due to falls (Ye et al. 2019).

Diabetes treatments, including insulin, sulfonylurea, and thiazolidinedione, are associated with an elevated risk of extremity fractures. The precise mechanisms underlying the effects of insulin and sulfonylureas on lower-extremity fractures remain unclear. However, insulin preserves the calories consumed by improving glycemic control, returning patients to glycemic levels below the renal threshold (Russell-Jones and Khan 2007), elevating insulin levels within the body, and concomitantly increasing calorie intake (Group 1998). The coexistence of type 2 diabetes and obesity is associated with an increased risk of fracture. Obesity has been shown to affect bone metabolism negatively (Vilaca et al. 2020). Given the lack of evidence regarding the precise mechanism of action of insulin or sulfonylureas in lower extremity fractures, weight gain may contribute to an increased risk of such fractures. Thiazolidinediones increase insulin sensitivity by activating peroxisome proliferator-activated receptor-gamma (PPARγ). Activation of PPARγ in bone marrow stromal cells promotes mesenchymal stem cell development into adipocytes while inhibiting differentiation into osteoblasts. PPARγ activity has been demonstrated to inhibit osteoblast differentiation and to induce osteoclast differentiation, resulting in a net decrease in bone production (Yki-Järvinen 2004). In addition, thiazolidinediones gained weight. A PROactive study reported that pioglitazone use in T2DM increased non-vertebral fracture risk compared to the control [5.1% (44/870), 2.5% (23/905)], and the risk of extremity fractures also increased in postmenopausal women (Lecka-Czernik 2013). In a real-world retrospective cohort study conducted in 2018 involving 12,277 patients with type 2 diabetes, sulfonylurea use was significantly associated with an increased risk of fractures (Losada et al. 2018). This finding is consistent with those of our previous study. However, individual RCTs involving metformin, sulfonylureas, thiazolidinediones, and insulin were mainly used as controls and had small sample sizes compared to other antidiabetic agents. In addition, the small number of individual RCTs reporting fractures with different risk factors, such as sex, precluded further analysis. Therefore, further large-scale studies that include these risk factors are required.

Recently, studies confirming the effectiveness of antidiabetic agents according to study duration have been reported. This study compared the fracture risk prevention effects of different drug types at 52, 78, and 104 weeks of follow-up. Nevertheless, the study findings did not demonstrate a statistically significant decrease in the fracture risk at any selected time interval. This finding corroborates the findings of an earlier study (Zhang et al. 2021b). To our knowledge, no study has analyzed various time points and fracture sites. Therefore, the increase in fracture risk may not be affected by the duration of antidiabetic agent use.

No large RCTs have confirmed the osteoporotic effect of antidiabetic agents as a clinical endpoint. Recent studies have suggested that diabetes may contribute to osteoporosis development (Cao et al. 2025). However, further research involving placebo-controlled trials is essential to evaluate the effects of diabetic medications on bone health. In 2008, the risk of heart failure associated with rosiglitazone led the FDA and EMA to introduce a cardiovascular outcome trial (CVOT) to evaluate CVD during the development of new diabetes drugs. Following guideline revisions, data related to the CVOT trial for monitoring cardiovascular safety in patients with T2DM who have developed cardiovascular disease or are at high risk for cardiovascular disease (Marx et al. 2015). The collected data have reported the potential cardioprotective effects of specific antidiabetic agents, including GLP1RA and SGLT2i. GLP1RA and SGLT2i have additional benefits in some CVOTs through multiple mechanisms, such as reduced major adverse cardiovascular events and CV mortality, reduced risk of hospitalization for heart failure, renal disease progression, and all-cause mortality (Zelniker et al. 2019). Currently, reporting osteoporosis is optional for the development of diabetes treatments. Most RCTs have not selected bone fractures or BMD as primary or secondary outcomes. Because data were collected as adverse events, there were limitations in the sample size and incidence. Therefore, we conducted the most comprehensive analysis, including all RCTs conducted. For some treatments, the results were statistically significant and neutral. Consequently, future RCTs are needed to elucidate the precise effect of antidiabetic drugs on osteoporosis by monitoring the impact of new medicines on fracture risk and BMD as primary or secondary endpoints.

This study has several limitations. First, although the sample size was significant, when analyzed separately by type of diabetic agent or type of fracture, small sample size groups were reported, and analyses by other risk factors, such as sex, could not be performed. Although we included only adult patients and conducted subgroup analyses by drug class to reduce heterogeneity, variations in age ranges, drug types, and inconsistent dose reporting across studies remained limitations. Therefore, this analysis must be treated with caution, and it is essential that future studies include a sufficiently large number of patients for each study or treatment type, and that analyses are performed using other risk factors. Further analysis of real-world data is required to bridge the gap between the efficacy demonstrated in RCTs and the outcomes observed in clinical practice. Second, most individual RCTs in this study reported fractures as adverse events, and heterogeneity was observed among the included studies. Therefore, there may be differences in the collection, reporting, and analysis of fracture data depending on individual studies. Bone turnover markers (BTM) are used to assess bone health, predict fracture risk, and monitor osteoporosis treatment (Civitelli et al. 2009). However, most individual RCTs have not reported BTM. Therefore, further studies are needed to analyze the role of BTM in antidiabetic treatments. The lack of direct data on these markers limits our ability to elucidate the precise biological mechanisms through which different hypoglycemic agents influence bone metabolism and turnover. Finally, our study's BMD analysis must be interpreted cautiously because the included studies were very few and heterogeneous. Nevertheless, this study was a large-scale direct and indirect meta-analysis that confirmed the effects of antidiabetic agents on bone health in patients with type 2 diabetes, including only RCTs. This study offers novel insights beyond previous meta-analyses by incorporating the most significant number of RCTs to date and applying both treatment- and anatomy-based fracture classifications. Unlike previous studies, we conducted duration-based subgroup analyses. These methodological advances have allowed for more precise and clinically accurate comparisons, enhancing the relevance and applicability of our findings. Without large-scale RCTs with fracture endpoints, a comprehensive synthesis remains the most effective method for demonstrating intervention efficacy. The quality of evidence from most of the included RCTs was moderate to high, supporting the reliability of our findings. Although this study did not introduce a novel antidiabetic agent, it provides the most comprehensive synthesis of the skeletal effects of these medications in patients with type 2 diabetes. Without large-scale RCTs with fracture as a primary endpoint, our findings, based on 242 trials and incorporating treatment- and anatomy-specific classifications and duration-based analyses, offer valuable comparative evidence. While immediate changes to clinical guidelines may be limited, this study establishes a critical foundation for future trials. It provides insights that may inform public health strategies for fracture prevention in diabetic populations.

This study is a large-scale direct and indirect meta-analysis confirming anti-diabetic agents' effectiveness for analyzing bone health in T2DM patients. In conclusion, antidiabetic agents tended to prevent fracture risk compared with placebo, and incretin-based therapies (GLP1RA or DPP4i) showed relatively better effects than other anti-diabetic agents. Diabetes treatment did not significantly affect BMD. Our study was a direct and indirect comparative study that included all currently available RCTs that confirmed the effects of diabetes treatment agents by site through detailed classification and impact according to period. This finding can be used as evidence at the beginning of well-conducted RCTs.

## Supplementary Information

Below is the link to the electronic supplementary material.

Supplementary file1 (DOCX 139893 KB)

## Data Availability

The data supporting this study's findings are available from the corresponding author upon reasonable request.
