# Supplementary material for: Evaluation of bone health and fracture risk in type 2 diabetes: a network meta-analysis of anti-diabetic treatments versus placebo
Source: Arch Pharm Res. 2025 Jun 20;48(6):563–75. doi: 10.1007/s12272-025-01552-2 (PMC12241289; doi:10.1007/s12272-025-01552-2)
Supplement: Supplementary file 1 — Supplementary file1 (DOCX 139893 KB) [file 12272_2025_1552_MOESM1_ESM.docx]

**Title**

Evaluation of Bone Health and Fracture Risk in Type 2 Diabetes: A Network Meta-analysis of Anti-Diabetic Treatments versus Placebo

[Authors]

SuA Oh, PhD^1^; Se-eun Park, MD^2^; EunYoung Kim, PharmD, PhD^1,*^

[Affiliations]

^1^Data science, Evidence-Based and Clinical Research Laboratory, Department of Health, Social and Clinical Pharmacy, College of Pharmacy, Chung-Ang University, Seoul, Republic of Korea. E-mail: [dhtndk0@cau.ac.kr](mailto:dhtndk0@cau.ac.kr) ORCID: 0000-0002-3809-2268

^2^Department of Internal Medicine, Division of Endocrinology and Metabolism, Kangbuk Samsung Hospital, Sungkyunkwan University School of Medicine, Seoul 03063, Korea. E-mail: [seraph.park@samsung.com](mailto:seraph.park@samsung.com) ORCID: 0000-0001-5682-4066

**Running title:** Effects of T2DM treatments on bone health

*Corresponding author: EunYoung Kim, Data science, Evidence-Based and Clinical Research Laboratory, Department of Health, Social and Clinical Pharmacy, College of Pharmacy, Chung-Ang University, 06974, Seoul, Republic of Korea. E-mail: [eykimjcb777@cau.ac.kr](mailto:eykimjcb777@cau.ac.kr). Phone: /82-02-820-5791 ORCID: 0000-0003-3525-8805

[PROSPERO registration number] CRD42024538789 https://www.crd.york.ac.uk/prospero/display_record.php?RecordID=538789

*Supplementary of Contents*

[Table S1. PRISMA checklist	6](#_Toc195023694)

[Table S2. Search strategies sued for systematic review	9](#_Toc195023695)

[Table S3. Baseline characteristics	14](#_Toc195023696)

[Table S4. Deviance information criterion for model selection	18](#_Toc195023697)

[Table S5. Network meta-analysis heterogeneity/inconsistency test (Fracture)_Treatment type	19](#_Toc195023698)

[Table S6. Network meta-analysis heterogeneity/inconsistency test(Fracture)_specific treatment	22](#_Toc195023699)

[Table S7. Network meta-analysis heterogeneity/inconsistency test (BMD)_Treatment type	30](#_Toc195023700)

[Table S8. Network meta-analysis heterogeneity/inconsistency test(BMD)_specific treatment	31](#_Toc195023701)

[Table S9. Network estimated effect sizes (95% confidence interval) for Overall Fracture	32](#_Toc195023702)

[Table S10. Network estimated effect sizes (95% confidence interval) for Overall Fracture_Treatment Type	33](#_Toc195023703)

[Table S11. Network estimated effect sizes (95% confidence interval) for Vertebral Fracture	34](#_Toc195023704)

[Table S12. Network estimated effect sizes (95% confidence interval) for Vertebral Fracture_Treatment Type	35](#_Toc195023705)

[Table S13. Network estimated effect sizes (95% confidence interval) for Hip Fracture	36](#_Toc195023706)

[Table S14. Network estimated effect sizes (95% confidence interval) for Hip Fracture_Treatment Type	37](#_Toc195023707)

[Table S15. Network estimated effect sizes (95% confidence interval) for Non-Vertebral Fracture	38](#_Toc195023708)

[Table S16. Network estimated effect sizes (95% confidence interval) for Non-Vertebral Fracture_Treatment Type	39](#_Toc195023709)

[Table S17. Network estimated effect sizes (95% confidence interval) for Vertebral & Hip Fracture	40](#_Toc195023710)

[Table S18. Network estimated effect sizes (95% confidence interval) for Vertebral & Hip Fracture_Treatment Type	41](#_Toc195023711)

[Table S19. Network estimated effect sizes (95% confidence interval) for Multiple Fracture	42](#_Toc195023712)

[Table S20. Network estimated effect sizes (95% confidence interval) for Multiple Fracture_Treatment Type	43](#_Toc195023713)

[Table S21. Network estimated effect sizes (95% confidence interval) for Cranial and Facial bones Fracture	44](#_Toc195023714)

[Table S22. Network estimated effect sizes (95% confidence interval) for Cranial and Facial bones Fracture_Treatment Type	45](#_Toc195023715)

[Table S23. Network estimated effect sizes (95% confidence interval) for Lower Extremity Fracture	46](#_Toc195023716)

[Table S24. Network estimated effect sizes (95% confidence interval) for Lower Extremity Fracture_Treatment Type	47](#_Toc195023717)

[Table S25. Network estimated effect sizes (95% confidence interval) for Pelvic Girdle Fracture	48](#_Toc195023718)

[Table S26. Network estimated effect sizes (95% confidence interval) for Pelvic Girdle Fracture_Treatment Type	49](#_Toc195023719)

[Table S27. Network estimated effect sizes (95% confidence interval) for Upper Extremity Fracture	50](#_Toc195023720)

[Table S28. Network estimated effect sizes (95% confidence interval) for Upper Extremity Fracture_Treatment Type	51](#_Toc195023721)

[Table S29. Network estimated effect sizes (95% confidence interval) for Vertebral Column Fracture	52](#_Toc195023722)

[Table S30. Network estimated effect sizes (95% confidence interval) for Vertebral Column Fracture_Treatment Type	53](#_Toc195023723)

[Table S31. Network estimated effect sizes (95% confidence interval) for Femoral Neck BMD	54](#_Toc195023724)

[Table S32. Network estimated effect sizes (95% confidence interval) for Femoral Neck BMD_Treatment Type	55](#_Toc195023725)

[Table S33. Network estimated effect sizes (95% confidence interval) for Total Hip BMD	56](#_Toc195023726)

[Table S34. Network estimated effect sizes (95% confidence interval) for Total Hip BMD_Treatment Type	57](#_Toc195023727)

[Table S35. Network estimated effect sizes (95% confidence interval) for Lumber spine BMD	58](#_Toc195023728)

[Table S36. Network estimated effect sizes (95% confidence interval) for Lumber Spine BMD_Treatment Type	59](#_Toc195023729)

[Table S37. Subgroup analysis of osteoporosis parameters by treatment type based on 52 weeks	60](#_Toc195023730)

[Table S38. Subgroup analysis of osteoporosis parameters by treatment type based on 78 weeks	62](#_Toc195023731)

[Table S39. Subgroup analysis of osteoporosis parameters by treatment type based on 104 weeks	64](#_Toc195023732)

[Table S40. Publication bias of meta-analysis results between anti-diabetic agents	66](#_Toc195023733)

[Table S41. Sensitivity analysis that excludes studies with the most participants	67](#_Toc195023734)

[Table S42. GRADE summary	68](#_Toc195023735)

[Table S43. Meta-regression of BMD	69](#_Toc195023736)

[Figure S1. Network plot for site of fracture between specific anti-diabetic agents.	70](#_Toc195023737)

[Figure S2. Network plot for anatomy fracture site between specific anti-diabetic agents.	71](#_Toc195023738)

[Figure S3. Network plot for BMD between specific anti-diabetic agents.	72](#_Toc195023739)

[Figure S4. Network plot for site of fracture between type of anti-diabetic agents	73](#_Toc195023740)

[Figure S5. Network plot for anatomy fracture site between specific anti-diabetic agents.	74](#_Toc195023741)

[Figure S6. Network plot for site of BMD between type of anti-diabetic agents.	75](#_Toc195023742)

[Figure S7. Forest plot of network meta-analysis results for site of fracture between specific anti-diabetic agents.	76](#_Toc195023743)

[Figure S7. Forest plot of network meta-analysis results for site of fracture between specific anti-diabetic agents (Continuous).	77](#_Toc195023744)

[Figure S8. Forest plot of network meta-analysis results for anatomy fracture site between specific anti-diabetic agents.	78](#_Toc195023745)

[Figure S9. Forest plot of network meta-analysis results for BMD between type of anti-diabetic agents.	79](#_Toc195023746)

[Figure S10. Forest plot of network meta-analysis results for BMD between specific anti-diabetic agents	80](#_Toc195023747)

[Figure S11. Funnel plot of network meta-analysis results for site of fracture between specific anti-diabetic agents.	81](#_Toc195023748)

[Figure S12. Funnel plot of network meta-analysis results for anatomy fracture site between specific anti-diabetic agents.	82](#_Toc195023749)

[Figure S13. Funnel plot of network meta-analysis results for site of fracture between type of anti-diabetic agents.	83](#_Toc195023750)

[Figure S14. Funnel plot of network meta-analysis results for anatomy fracture site between type of anti-diabetic agents.	84](#_Toc195023751)

[Figure S15. Funnel plot of network meta-analysis results for BMD between specific anti-diabetic agents	85](#_Toc195023752)

[Figure S16. Funnel plot of network meta-analysis results for BMD between type of anti-diabetic agents	86](#_Toc195023753)

[Figure S17. Risk of bias assessment (Traffic-light plot)	87](#_Toc195023754)

[Figure S17. Risk of bias assessment (Traffic-light plot)	88](#_Toc195023755)

[Figure S17. Risk of bias assessment (Traffic-light plot)	89](#_Toc195023756)

[Figure S17. Risk of bias assessment (Traffic-light plot)	90](#_Toc195023757)

[Figure S17. Risk of bias assessment (Traffic-light plot)	91](#_Toc195023758)

[Figure S18. Risk of bias assessment (Summary plot)	92](#_Toc195023759)

Table S1. PRISMA checklist

| **Section and Topic** | **Item #** | **Checklist item** | **Location where item is reported** |
| --- | --- | --- | --- |
| TITLE | | |  |
| Title | 1 | Identify the report as a systematic review. | 1 |
| ABSTRACT | | |  |
| Abstract | 2 | See the PRISMA 2020 for Abstracts checklist. | 2-3 |
| INTRODUCTION | | |  |
| Rationale | 3 | Describe the rationale for the review in the context of existing knowledge. | 3 |
| Objectives | 4 | Provide an explicit statement of the objective(s) or question(s) the review addresses. | 4 |
| METHODS | | |  |
| Eligibility criteria | 5 | Specify the inclusion and exclusion criteria for the review and how studies were grouped for the syntheses. | 6 |
| Information sources | 6 | Specify all databases, registers, websites, organisations, reference lists and other sources searched or consulted to identify studies. Specify the date when each source was last searched or consulted. | 6-7 |
| Search strategy | 7 | Present the full search strategies for all databases, registers and websites, including any filters and limits used. | ESM Table S2 |
| Selection process | 8 | Specify the methods used to decide whether a study met the inclusion criteria of the review, including how many reviewers screened each record and each report retrieved, whether they worked independently, and if applicable, details of automation tools used in the process. | Figure1 |
| Data collection process | 9 | Specify the methods used to collect data from reports, including how many reviewers collected data from each report, whether they worked independently, any processes for obtaining or confirming data from study investigators, and if applicable, details of automation tools used in the process. | Figure1 |
| Data items | 10a | List and define all outcomes for which data were sought. Specify whether all results that were compatible with each outcome domain in each study were sought (e.g. for all measures, time points, analyses), and if not, the methods used to decide which results to collect. | 7-8 |
|  | 10b | List and define all other variables for which data were sought (e.g. participant and intervention characteristics, funding sources). Describe any assumptions made about any missing or unclear information. | 6 |
| Study risk of bias assessment | 11 | Specify the methods used to assess risk of bias in the included studies, including details of the tool(s) used, how many reviewers assessed each study and whether they worked independently, and if applicable, details of automation tools used in the process. | 7-8 |
| Effect measures | 12 | Specify for each outcome the effect measure(s) (e.g. risk ratio, mean difference) used in the synthesis or presentation of results. | 7-8 |
| Synthesis methods | 13a | Describe the processes used to decide which studies were eligible for each synthesis (e.g. tabulating the study intervention characteristics and comparing against the planned groups for each synthesis (item #5)). | 7-8 |
|  | 13b | Describe any methods required to prepare the data for presentation or synthesis, such as handling of missing summary statistics, or data conversions. | 7-8 |
|  | 13c | Describe any methods used to tabulate or visually display results of individual studies and syntheses. | 7-8 |
|  | 13d | Describe any methods used to synthesize results and provide a rationale for the choice(s). If meta-analysis was performed, describe the model(s), method(s) to identify the presence and extent of statistical heterogeneity, and software package(s) used. | 7-8 |
|  | 13e | Describe any methods used to explore possible causes of heterogeneity among study results (e.g. subgroup analysis, meta-regression). | 7-8 |
|  | 13f | Describe any sensitivity analyses conducted to assess robustness of the synthesized results. | 7-8 |
| Reporting bias assessment | 14 | Describe any methods used to assess risk of bias due to missing results in a synthesis (arising from reporting biases). | 7-8 |
| Certainty assessment | 15 | Describe any methods used to assess certainty (or confidence) in the body of evidence for an outcome. | 8-9 |
| RESULTS | | |  |
| Study selection | 16a | Describe the results of the search and selection process, from the number of records identified in the search to the number of studies included in the review, ideally using a flow diagram. | 10-11 |
|  | 16b | Cite studies that might appear to meet the inclusion criteria, but which were excluded, and explain why they were excluded. | 10-11 |
| Study characteristics | 17 | Cite each included study and present its characteristics. | Table 1 |
| Risk of bias in studies | 18 | Present assessments of risk of bias for each included study. | 15 |
| Results of individual studies | 19 | For all outcomes, present, for each study: (a) summary statistics for each group (where appropriate) and (b) an effect estimate and its precision (e.g. confidence/credible interval), ideally using structured tables or plots. | Table1 |
| Results of syntheses | 20a | For each synthesis, briefly summarise the characteristics and risk of bias among contributing studies. | 10-16 |
|  | 20b | Present results of all statistical syntheses conducted. If meta-analysis was done, present for each the summary estimate and its precision (e.g. confidence/credible interval) and measures of statistical heterogeneity. If comparing groups, describe the direction of the effect. | 10-16 |
|  | 20c | Present results of all investigations of possible causes of heterogeneity among study results. | 10-16 |
|  | 20d | Present results of all sensitivity analyses conducted to assess the robustness of the synthesized results. | 10-16 |
| Reporting biases | 21 | Present assessments of risk of bias due to missing results (arising from reporting biases) for each synthesis assessed. | 15 |
| Certainty of evidence | 22 | Present assessments of certainty (or confidence) in the body of evidence for each outcome assessed. | 10-16 |
| DISCUSSION | | |  |
| Discussion | 23a | Provide a general interpretation of the results in the context of other evidence. | 16-21 |
|  | 23b | Discuss any limitations of the evidence included in the review. | 16-21 |
|  | 23c | Discuss any limitations of the review processes used. | 16-21 |
|  | 23d | Discuss implications of the results for practice, policy, and future research. | 16-21 |
| OTHER INFORMATION | | |  |
| Registration and protocol | 24a | Provide registration information for the review, including register name and registration number, or state that the review was not registered. | 6 |
|  | 24b | Indicate where the review protocol can be accessed, or state that a protocol was not prepared. | 6 |
|  | 24c | Describe and explain any amendments to information provided at registration or in the protocol. | 6 |
| Support | 25 | Describe sources of financial or non-financial support for the review, and the role of the funders or sponsors in the review. | 21 |
| Competing interests | 26 | Declare any competing interests of review authors. | 22 |
| Availability of data, code and other materials | 27 | Report which of the following are publicly available and where they can be found: template data collection forms; data extracted from included studies; data used for all analyses; analytic code; any other materials used in the review. | 23 |

Table S2. Search strategies sued for systematic review

| **Database/Registry** | **Search criteria (~2024.03.18)** |
| --- | --- |
| MEDLINE-Pubmed | (((("sodium glucose transport proteins"[MeSH Terms] OR ("sodium glucose"[All Fields] AND "transport"[All Fields] AND "proteins"[All Fields]) OR "sodium glucose transport proteins"[All Fields] OR ("sodium"[All Fields] AND "glucose"[All Fields] AND "cotransporter"[All Fields]) OR "sodium glucose cotransporter"[All Fields]) AND "2"[All Fields] AND ("antagonists and inhibitors"[MeSH Subheading] OR ("antagonists"[All Fields] AND "inhibitors"[All Fields]) OR "antagonists and inhibitors"[All Fields] OR "inhibitors"[All Fields] OR "inhibitor"[All Fields] OR "inhibitor s"[All Fields])) OR ("sodium glucose transporter 2 inhibitors"[Pharmacological Action] OR "sodium glucose transporter 2 inhibitors"[MeSH Terms] OR "sodium glucose transporter 2 inhibitors"[All Fields] OR ("sglt2"[All Fields] AND "inhibitor"[All Fields]) OR "sglt2 inhibitor"[All Fields]) OR ("sodium glucose transporter 2 inhibitors"[Pharmacological Action] OR "sodium glucose transporter 2 inhibitors"[MeSH Terms] OR "sodium glucose transporter 2 inhibitors"[All Fields] OR "gliflozin"[All Fields] OR "gliflozins"[All Fields]) OR ("glucagon like peptide 1 receptor agonists"[Pharmacological Action] OR "glucagon like peptide 1 receptor agonists"[MeSH Terms] OR ("glucagon like"[All Fields] AND "peptide 1"[All Fields] AND "receptor"[All Fields] AND "agonists"[All Fields]) OR "glucagon like peptide 1 receptor agonists"[All Fields] OR "glucagon like peptide 1 receptor agonist"[All Fields]) OR (("glucagon like peptide 1"[MeSH Terms] OR "glucagon like peptide 1"[All Fields] OR "glp 1"[All Fields]) AND ("agonist"[All Fields] OR "agonist s"[All Fields] OR "agonistic"[All Fields] OR "agonistically"[All Fields] OR "agonistics"[All Fields] OR "agonists"[MeSH Subheading] OR "agonists"[All Fields])) OR (("glucagon like peptide 1 receptor"[MeSH Terms] OR ("glucagon like"[All Fields] AND "peptide 1"[All Fields] AND "receptor"[All Fields]) OR "glucagon like peptide 1 receptor"[All Fields] OR "glucagon like peptide 1 receptor"[All Fields]) AND ("stimulate"[All Fields] OR "stimulated"[All Fields] OR "stimulates"[All Fields] OR "stimulating"[All Fields] OR "stimulation"[All Fields] OR "stimulations"[All Fields] OR "stimulative"[All Fields] OR "stimulator"[All Fields] OR "stimulator s"[All Fields] OR "stimulators"[All Fields]) AND ("agent"[All Fields] OR "agents"[All Fields])) OR ("dipeptidyl peptidase iv inhibitors"[Pharmacological Action] OR "dipeptidyl peptidase iv inhibitors"[MeSH Terms] OR ("dipeptidyl peptidase"[All Fields] AND "iv"[All Fields] AND "inhibitors"[All Fields]) OR "dipeptidyl peptidase iv inhibitors"[All Fields] OR ("dipeptidyl"[All Fields] AND "peptidase"[All Fields] AND "iv"[All Fields] AND "inhibitor"[All Fields]) OR "dipeptidyl peptidase iv inhibitor"[All Fields]) OR ("dipeptidyl peptidase iv inhibitors"[Pharmacological Action] OR "dipeptidyl peptidase iv inhibitors"[MeSH Terms] OR ("dipeptidyl peptidase"[All Fields] AND "iv"[All Fields] AND "inhibitors"[All Fields]) OR "dipeptidyl peptidase iv inhibitors"[All Fields] OR "dpp 4 inhibitor"[All Fields]) OR ("dipeptidyl peptidase iv inhibitors"[Pharmacological Action] OR "dipeptidyl peptidase iv inhibitors"[MeSH Terms] OR ("dipeptidyl peptidase"[All Fields] AND "iv"[All Fields] AND "inhibitors"[All Fields]) OR "dipeptidyl peptidase iv inhibitors"[All Fields] OR ("dpp"[All Fields] AND "iv"[All Fields] AND "inhibitor"[All Fields]) OR "dpp iv inhibitor"[All Fields]) OR (("dipeptidylpeptidase"[All Fields] OR "dipeptidylpeptidases"[All Fields]) AND ("ieee intell veh symp"[Journal] OR "iv"[All Fields]) AND ("antagonists and inhibitors"[MeSH Subheading] OR ("antagonists"[All Fields] AND "inhibitors"[All Fields]) OR "antagonists and inhibitors"[All Fields] OR "inhibitors"[All Fields] OR "inhibitor"[All Fields] OR "inhibitor s"[All Fields])) OR ("dipeptidyl peptidase iv inhibitors"[Pharmacological Action] OR "dipeptidyl peptidase iv inhibitors"[MeSH Terms] OR ("dipeptidyl peptidase"[All Fields] AND "iv"[All Fields] AND "inhibitors"[All Fields]) OR "dipeptidyl peptidase iv inhibitors"[All Fields] OR "gliptin"[All Fields] OR "gliptins"[All Fields]) OR ("insulin"[MeSH Terms] OR "insulin"[All Fields] OR "insulin s"[All Fields] OR "insuline"[All Fields] OR "insulinic"[All Fields] OR "insulinization"[All Fields] OR "insulinized"[All Fields] OR "insulins"[MeSH Terms] OR "insulins"[All Fields]) OR ("glucosidases"[MeSH Terms] OR "glucosidases"[All Fields] OR "glucosidase"[All Fields]) OR (("bromocriptine"[MeSH Terms] OR "bromocriptine"[All Fields] OR "bromocriptin"[All Fields] OR "bromocriptine s"[All Fields]) AND ("quat res"[Journal] OR "qual res"[Journal] OR "qr"[All Fields])) OR ("meglitinide"[Supplementary Concept] OR "meglitinide"[All Fields] OR "meglitinides"[All Fields]) OR (("biguanid"[All Fields] OR "biguanides"[MeSH Terms] OR "biguanides"[All Fields] OR "biguanide"[All Fields] OR "biguanids"[All Fields]) AND ("analogs and derivatives"[MeSH Subheading] OR ("analogs"[All Fields] AND "derivatives"[All Fields]) OR "analogs and derivatives"[All Fields] OR "derivatives"[All Fields] OR "derivable"[All Fields] OR "derivant"[All Fields] OR "derivants"[All Fields] OR "derivate"[All Fields] OR "derivated"[All Fields] OR "derivates"[All Fields] OR "derivation"[All Fields] OR "derivations"[All Fields] OR "derivative"[All Fields] OR "derive"[All Fields] OR "derived"[All Fields] OR "derives"[All Fields] OR "deriving"[All Fields])) OR ("sulfonylurea compounds"[MeSH Terms] OR ("sulfonylurea"[All Fields] AND "compounds"[All Fields]) OR "sulfonylurea compounds"[All Fields] OR "sulfonylurea"[All Fields] OR "sulfonylureas"[All Fields] OR "sulphonylurea"[All Fields] OR "sulphonylureas"[All Fields]) OR ("metformin"[MeSH Terms] OR "metformin"[All Fields] OR "metformine"[All Fields] OR "metformin s"[All Fields] OR "metformins"[All Fields]) OR ("2 4 thiazolidinedione"[Supplementary Concept] OR "2 4 thiazolidinedione"[All Fields] OR "thiazolidinedione"[All Fields] OR "thiazolidinediones"[MeSH Terms] OR "thiazolidinediones"[All Fields])) AND ("randomized controlled trial"[Publication Type] OR "randomized controlled trials as topic"[MeSH Terms] OR "randomized controlled trial"[All Fields] OR "randomised controlled trial"[All Fields] OR ("controlled clinical trial"[Publication Type] OR "controlled clinical trials as topic"[MeSH Terms] OR "controlled clinical trial"[All Fields]) OR ("random allocation"[MeSH Terms] OR ("random"[All Fields] AND "allocation"[All Fields]) OR "random allocation"[All Fields] OR "randomization"[All Fields] OR "randomized"[All Fields] OR "random"[All Fields] OR "randomisation"[All Fields] OR "randomisations"[All Fields] OR "randomise"[All Fields] OR "randomised"[All Fields] OR "randomising"[All Fields] OR "randomizations"[All Fields] OR "randomize"[All Fields] OR "randomizes"[All Fields] OR "randomizing"[All Fields] OR "randomness"[All Fields] OR "randoms"[All Fields]) OR "randomly"[All Fields] OR "RCTs"[All Fields] OR ("diabetes mellitus, type 2"[MeSH Terms] OR "type 2 diabetes mellitus"[All Fields] OR "type 2 diabetes"[All Fields]) OR ("randomized controlled trial"[Publication Type] OR "randomized controlled trials as topic"[MeSH Terms] OR "clinical trials randomized"[All Fields] OR "clinical trials randomised"[All Fields]) OR ("randomized controlled trial"[Publication Type] OR "randomized controlled trials as topic"[MeSH Terms] OR "trials randomized clinical"[All Fields] OR "trials randomised clinical"[All Fields]) OR ("randomized controlled trial"[Publication Type] OR "randomized controlled trials as topic"[MeSH Terms] OR "controlled clinical trials randomized"[All Fields] OR "controlled clinical trials randomised"[All Fields]) OR ("diabetes mellitus, type 2"[MeSH Terms] OR "type 2 diabetes mellitus"[All Fields] OR ("diabetes"[All Fields] AND "mellitus"[All Fields] AND "type"[All Fields] AND "ii"[All Fields]) OR "diabetes mellitus type ii"[All Fields]) OR ("diabetes mellitus, type 2"[MeSH Terms] OR "type 2 diabetes mellitus"[All Fields]) OR ("diabetes mellitus, type 2"[MeSH Terms] OR "type 2 diabetes mellitus"[All Fields] OR "diabetes type 2"[All Fields]) OR ("diabetes mellitus, type 2"[MeSH Terms] OR "type 2 diabetes mellitus"[All Fields] OR ("diabetes"[All Fields] AND "mellitus"[All Fields] AND "adult"[All Fields] AND "onset"[All Fields]) OR "diabetes mellitus adult onset"[All Fields]) OR ("diabetes mellitus, type 2"[MeSH Terms] OR "type 2 diabetes mellitus"[All Fields] OR ("adult"[All Fields] AND "onset"[All Fields] AND "diabetes"[All Fields] AND "mellitus"[All Fields]) OR "adult onset diabetes mellitus"[All Fields]) OR ("diabetes mellitus, type 2"[MeSH Terms] OR "type 2 diabetes mellitus"[All Fields] OR ("diabetes"[All Fields] AND "mellitus"[All Fields] AND "adult"[All Fields] AND "onset"[All Fields]) OR "diabetes mellitus adult onset"[All Fields]) OR ("randomized controlled trials as topic"[MeSH Terms] OR ("randomized"[All Fields] AND "controlled"[All Fields] AND "trials"[All Fields] AND "topic"[All Fields]) OR "randomized controlled trials as topic"[All Fields] OR ("controlled"[All Fields] AND "trial"[All Fields] AND "randomized"[All Fields]) OR "controlled trial randomized"[All Fields]) OR (("random allocation"[MeSH Terms] OR ("random"[All Fields] AND "allocation"[All Fields]) OR "random allocation"[All Fields] OR "randomization"[All Fields] OR "randomized"[All Fields] OR "random"[All Fields] OR "randomisation"[All Fields] OR "randomisations"[All Fields] OR "randomise"[All Fields] OR "randomised"[All Fields] OR "randomising"[All Fields] OR "randomizations"[All Fields] OR "randomize"[All Fields] OR "randomizes"[All Fields] OR "randomizing"[All Fields] OR "randomness"[All Fields] OR "randoms"[All Fields]) AND "controlled"[All Fields] AND ("studies"[All Fields] OR "study"[All Fields] OR "study s"[All Fields] OR "studying"[All Fields] OR "studys"[All Fields])) OR ("randomized controlled trial"[Publication Type] OR "randomized controlled trials as topic"[MeSH Terms] OR "randomised controlled trial"[All Fields] OR "randomized controlled trial"[All Fields]) OR (("random allocation"[MeSH Terms] OR ("random"[All Fields] AND "allocation"[All Fields]) OR "random allocation"[All Fields] OR "randomization"[All Fields] OR "randomized"[All Fields] OR "random"[All Fields] OR "randomisation"[All Fields] OR "randomisations"[All Fields] OR "randomise"[All Fields] OR "randomised"[All Fields] OR "randomising"[All Fields] OR "randomizations"[All Fields] OR "randomize"[All Fields] OR "randomizes"[All Fields] OR "randomizing"[All Fields] OR "randomness"[All Fields] OR "randoms"[All Fields]) AND "controlled"[All Fields] AND ("studies"[All Fields] OR "study"[All Fields] OR "study s"[All Fields] OR "studying"[All Fields] OR "studys"[All Fields])) OR (("clinical trials as topic"[MeSH Terms] OR ("clinical"[All Fields] AND "trials"[All Fields] AND "topic"[All Fields]) OR "clinical trials as topic"[All Fields] OR "trial"[All Fields] OR "trial s"[All Fields] OR "trialed"[All Fields] OR "trialing"[All Fields] OR "trials"[All Fields]) AND ("random allocation"[MeSH Terms] OR ("random"[All Fields] AND "allocation"[All Fields]) OR "random allocation"[All Fields] OR "randomization"[All Fields] OR "randomized"[All Fields] OR "random"[All Fields] OR "randomisation"[All Fields] OR "randomisations"[All Fields] OR "randomise"[All Fields] OR "randomised"[All Fields] OR "randomising"[All Fields] OR "randomizations"[All Fields] OR "randomize"[All Fields] OR "randomizes"[All Fields] OR "randomizing"[All Fields] OR "randomness"[All Fields] OR "randoms"[All Fields]) AND "controlled"[All Fields]) OR ("diabetes mellitus, type 2"[MeSH Terms] OR "type 2 diabetes mellitus"[All Fields] OR ("adult"[All Fields] AND "onset"[All Fields] AND "diabetes"[All Fields]) OR "adult onset diabetes"[All Fields]) OR ("diabetes mellitus, type 2"[MeSH Terms] OR "type 2 diabetes mellitus"[All Fields] OR "diabetes mellitus type 2"[All Fields]) OR ("diabetes mellitus, type 2"[MeSH Terms] OR "type 2 diabetes mellitus"[All Fields] OR ("type"[All Fields] AND "ii"[All Fields] AND "diabetes"[All Fields]) OR "type ii diabetes"[All Fields]) OR ("diabetes mellitus, type 2"[MeSH Terms] OR "type 2 diabetes mellitus"[All Fields] OR "type 2 diabetes"[All Fields]) OR "T2DM"[All Fields] OR ("diabetes mellitus, type 2"[MeSH Terms] OR "type 2 diabetes mellitus"[All Fields] OR ("noninsulin"[All Fields] AND "dependent"[All Fields] AND "diabetes"[All Fields] AND "mellitus"[All Fields]) OR "noninsulin dependent diabetes mellitus"[All Fields]) OR ("diabetes mellitus, type 2"[MeSH Terms] OR "type 2 diabetes mellitus"[All Fields] OR ("noninsulin"[All Fields] AND "dependent"[All Fields] AND "diabetes"[All Fields]) OR "noninsulin dependent diabetes"[All Fields]) OR (("diabetes mellitus, type 2"[MeSH Terms] OR "type 2 diabetes mellitus"[All Fields] OR "niddm"[All Fields] OR "niddms"[All Fields]) AND ("diabetes mellitus, type 2"[MeSH Terms] OR "type 2 diabetes mellitus"[All Fields] OR ("non"[All Fields] AND "insulin"[All Fields] AND "dependent"[All Fields] AND "diabetes"[All Fields] AND "mellitus"[All Fields]) OR "non insulin dependent diabetes mellitus"[All Fields])) OR ("diabetes mellitus, type 2"[MeSH Terms] OR "type 2 diabetes mellitus"[All Fields] OR ("non"[All Fields] AND "insulin"[All Fields] AND "dependent"[All Fields] AND "diabetes"[All Fields]) OR "non insulin dependent diabetes"[All Fields]) OR ("diabetes mellitus, type 2"[MeSH Terms] OR "type 2 diabetes mellitus"[All Fields] OR "niddm"[All Fields] OR "niddms"[All Fields]) OR ("diabetes mellitus, type 2"[MeSH Terms] OR "type 2 diabetes mellitus"[All Fields] OR ("ketosis"[All Fields] AND "resistant"[All Fields] AND "diabetes"[All Fields] AND "mellitus"[All Fields]) OR "ketosis resistant diabetes mellitus"[All Fields]) OR ("diabetes mellitus, type 2"[MeSH Terms] OR "type 2 diabetes mellitus"[All Fields] OR ("maturity"[All Fields] AND "onset"[All Fields] AND "diabetes"[All Fields]) OR "maturity onset diabetes"[All Fields]) OR ("diabetes mellitus, type 2"[MeSH Terms] OR "type 2 diabetes mellitus"[All Fields] OR ("maturity"[All Fields] AND "onset"[All Fields] AND "diabetes"[All Fields] AND "mellitus"[All Fields]) OR "maturity onset diabetes mellitus"[All Fields]) OR ("mason type diabetes"[Supplementary Concept] OR "mason type diabetes"[All Fields] OR "maturity onset diabetes of the young"[All Fields]) OR ("diabetes mellitus, type 2"[MeSH Terms] OR "type 2 diabetes mellitus"[All Fields] OR ("diabetes"[All Fields] AND "mellitus"[All Fields] AND "type"[All Fields] AND "ii"[All Fields]) OR "diabetes mellitus type ii"[All Fields]) OR (("insulin"[MeSH Terms] OR "insulin"[All Fields] OR "insulin s"[All Fields] OR "insuline"[All Fields] OR "insulinic"[All Fields] OR "insulinization"[All Fields] OR "insulinized"[All Fields] OR "insulins"[MeSH Terms] OR "insulins"[All Fields]) AND ("independent"[All Fields] OR "independently"[All Fields] OR "independents"[All Fields]) AND ("diabetes mellitus"[MeSH Terms] OR ("diabetes"[All Fields] AND "mellitus"[All Fields]) OR "diabetes mellitus"[All Fields])) OR (("insulin"[MeSH Terms] OR "insulin"[All Fields] OR "insulin s"[All Fields] OR "insuline"[All Fields] OR "insulinic"[All Fields] OR "insulinization"[All Fields] OR "insulinized"[All Fields] OR "insulins"[MeSH Terms] OR "insulins"[All Fields]) AND ("independent"[All Fields] OR "independently"[All Fields] OR "independents"[All Fields]) AND ("diabete"[All Fields] OR "diabetes mellitus"[MeSH Terms] OR ("diabetes"[All Fields] AND "mellitus"[All Fields]) OR "diabetes mellitus"[All Fields] OR "diabetes"[All Fields] OR "diabetes insipidus"[MeSH Terms] OR ("diabetes"[All Fields] AND "insipidus"[All Fields]) OR "diabetes insipidus"[All Fields] OR "diabetic"[All Fields] OR "diabetics"[All Fields] OR "diabets"[All Fields])) OR (("dyn med"[Journal] OR "dis mon"[Journal] OR "dis manag"[Journal] OR "dm"[All Fields]) AND "2"[All Fields]) OR ("diabetes mellitus, type 2"[MeSH Terms] OR "type 2 diabetes mellitus"[All Fields] OR ("diabetes"[All Fields] AND "adult"[All Fields] AND "onset"[All Fields]) OR "diabetes adult onset"[All Fields]) OR ("diabetes mellitus, type 2"[MeSH Terms] OR "type 2 diabetes mellitus"[All Fields] OR ("diabetes"[All Fields] AND "type"[All Fields] AND "ii"[All Fields]) OR "diabetes type ii"[All Fields]) OR ("diabetes mellitus, type 2"[MeSH Terms] OR "type 2 diabetes mellitus"[All Fields] OR ("diabetes"[All Fields] AND "mellitus"[All Fields] AND "type"[All Fields] AND "ii"[All Fields]) OR "diabetes mellitus type ii"[All Fields])) AND ((("bone and bones"[MeSH Terms] OR ("bone"[All Fields] AND "bones"[All Fields]) OR "bone and bones"[All Fields] OR "osseous"[All Fields]) AND ("densities"[All Fields] OR "density"[All Fields])) OR (("unstability"[All Fields] OR "unstable"[All Fields]) AND ("fractur"[All Fields] OR "fractural"[All Fields] OR "fracture s"[All Fields] OR "fractures, bone"[MeSH Terms] OR ("fractures"[All Fields] AND "bone"[All Fields]) OR "bone fractures"[All Fields] OR "fracture"[All Fields] OR "fractured"[All Fields] OR "fractures"[All Fields] OR "fracturing"[All Fields])) OR (("skeleton"[MeSH Terms] OR "skeleton"[All Fields] OR "skeletons"[All Fields] OR "skeleton s"[All Fields]) AND ("fractur"[All Fields] OR "fractural"[All Fields] OR "fracture s"[All Fields] OR "fractures, bone"[MeSH Terms] OR ("fractures"[All Fields] AND "bone"[All Fields]) OR "bone fractures"[All Fields] OR "fracture"[All Fields] OR "fractured"[All Fields] OR "fractures"[All Fields] OR "fracturing"[All Fields])) OR ("fractures, closed"[MeSH Terms] OR ("fractures"[All Fields] AND "closed"[All Fields]) OR "closed fractures"[All Fields] OR "fractures closed"[All Fields]) OR ("fractures, bone"[MeSH Terms] OR ("fractures"[All Fields] AND "bone"[All Fields]) OR "bone fractures"[All Fields] OR "fractures bone"[All Fields]) OR ("fractur"[All Fields] OR "fractural"[All Fields] OR "fracture s"[All Fields] OR "fractures, bone"[MeSH Terms] OR ("fractures"[All Fields] AND "bone"[All Fields]) OR "bone fractures"[All Fields] OR "fracture"[All Fields] OR "fractured"[All Fields] OR "fractures"[All Fields] OR "fracturing"[All Fields]) OR ("fractures, closed"[MeSH Terms] OR ("fractures"[All Fields] AND "closed"[All Fields]) OR "closed fractures"[All Fields] OR ("closed"[All Fields] AND "fracture"[All Fields]) OR "closed fracture"[All Fields]) OR (("bone cements"[Pharmacological Action] OR "bone cements"[MeSH Terms] OR ("bone"[All Fields] AND "cements"[All Fields]) OR "bone cements"[All Fields] OR ("bone"[All Fields] AND "cement"[All Fields]) OR "bone cement"[All Fields]) AND ("fractur"[All Fields] OR "fractural"[All Fields] OR "fracture s"[All Fields] OR "fractures, bone"[MeSH Terms] OR ("fractures"[All Fields] AND "bone"[All Fields]) OR "bone fractures"[All Fields] OR "fracture"[All Fields] OR "fractured"[All Fields] OR "fractures"[All Fields] OR "fracturing"[All Fields])) OR ("bone density"[MeSH Terms] OR ("bone"[All Fields] AND "density"[All Fields]) OR "bone density"[All Fields] OR ("bone"[All Fields] AND "mineral"[All Fields] AND "contents"[All Fields]) OR "bone mineral contents"[All Fields]) OR ("bone density"[MeSH Terms] OR ("bone"[All Fields] AND "density"[All Fields]) OR "bone density"[All Fields] OR ("bone"[All Fields] AND "mineral"[All Fields] AND "content"[All Fields]) OR "bone mineral content"[All Fields]) OR ("bone density"[MeSH Terms] OR ("bone"[All Fields] AND "density"[All Fields]) OR "bone density"[All Fields] OR ("density"[All Fields] AND "bone"[All Fields] AND "mineral"[All Fields]) OR "density bone mineral"[All Fields]) OR ("bone density"[MeSH Terms] OR ("bone"[All Fields] AND "density"[All Fields]) OR "bone density"[All Fields] OR ("bone"[All Fields] AND "mineral"[All Fields] AND "densities"[All Fields]) OR "bone mineral densities"[All Fields]) OR ("bone density"[MeSH Terms] OR ("bone"[All Fields] AND "density"[All Fields]) OR "bone density"[All Fields] OR ("density"[All Fields] AND "bone"[All Fields]) OR "density bone"[All Fields]) OR ("bone density"[MeSH Terms] OR ("bone"[All Fields] AND "density"[All Fields]) OR "bone density"[All Fields] OR ("bone"[All Fields] AND "densities"[All Fields]) OR "bone densities"[All Fields]) OR ("fractures, bone"[MeSH Terms] OR ("fractures"[All Fields] AND "bone"[All Fields]) OR "bone fractures"[All Fields] OR ("torsion"[All Fields] AND "fracture"[All Fields]) OR "torsion fracture"[All Fields]) OR ("fractures, bone"[MeSH Terms] OR ("fractures"[All Fields] AND "bone"[All Fields]) OR "bone fractures"[All Fields] OR ("fractures"[All Fields] AND "torsion"[All Fields]) OR "fractures torsion"[All Fields]) OR ("fractures, bone"[MeSH Terms] OR ("fractures"[All Fields] AND "bone"[All Fields]) OR "bone fractures"[All Fields] OR ("fracture"[All Fields] AND "torsion"[All Fields]) OR "fracture torsion"[All Fields]) OR ("fractures, bone"[MeSH Terms] OR ("fractures"[All Fields] AND "bone"[All Fields]) OR "bone fractures"[All Fields] OR ("torsion"[All Fields] AND "fractures"[All Fields]) OR "torsion fractures"[All Fields]) OR ("fractures, bone"[MeSH Terms] OR ("fractures"[All Fields] AND "bone"[All Fields]) OR "bone fractures"[All Fields] OR ("spiral"[All Fields] AND "fracture"[All Fields]) OR "spiral fracture"[All Fields]) OR ("fractures, bone"[MeSH Terms] OR ("fractures"[All Fields] AND "bone"[All Fields]) OR "bone fractures"[All Fields] OR ("fractures"[All Fields] AND "spiral"[All Fields]) OR "fractures spiral"[All Fields]) OR ("fractures, bone"[MeSH Terms] OR ("fractures"[All Fields] AND "bone"[All Fields]) OR "bone fractures"[All Fields] OR ("fracture"[All Fields] AND "spiral"[All Fields]) OR "fracture spiral"[All Fields]) OR ("fractures, bone"[MeSH Terms] OR ("fractures"[All Fields] AND "bone"[All Fields]) OR "bone fractures"[All Fields] OR ("spiral"[All Fields] AND "fractures"[All Fields]) OR "spiral fractures"[All Fields]) OR ("fractures, bone"[MeSH Terms] OR ("fractures"[All Fields] AND "bone"[All Fields]) OR "bone fractures"[All Fields] OR ("fracture"[All Fields] AND "bone"[All Fields]) OR "fracture bone"[All Fields]) OR ("fractures, bone"[MeSH Terms] OR ("fractures"[All Fields] AND "bone"[All Fields]) OR "bone fractures"[All Fields] OR ("bone"[All Fields] AND "fracture"[All Fields]) OR "bone fracture"[All Fields]) OR ("fractures, bone"[MeSH Terms] OR ("fractures"[All Fields] AND "bone"[All Fields]) OR "bone fractures"[All Fields] OR ("broken"[All Fields] AND "bone"[All Fields]) OR "broken bone"[All Fields]) OR ("fractures, bone"[MeSH Terms] OR ("fractures"[All Fields] AND "bone"[All Fields]) OR "bone fractures"[All Fields] OR ("bones"[All Fields] AND "broken"[All Fields]) OR "bones broken"[All Fields]) OR ("fractures, bone"[MeSH Terms] OR ("fractures"[All Fields] AND "bone"[All Fields]) OR "bone fractures"[All Fields] OR ("bone"[All Fields] AND "broken"[All Fields]) OR "bone broken"[All Fields]) OR ("fractures, bone"[MeSH Terms] OR ("fractures"[All Fields] AND "bone"[All Fields]) OR "bone fractures"[All Fields] OR ("broken"[All Fields] AND "bones"[All Fields]) OR "broken bones"[All Fields]) OR "BMD"[All Fields] OR ("bone density"[MeSH Terms] OR ("bone"[All Fields] AND "density"[All Fields]) OR "bone density"[All Fields] OR ("bone"[All Fields] AND "mineral"[All Fields] AND "density"[All Fields]) OR "bone mineral density"[All Fields]) OR ("fractur"[All Fields] OR "fractural"[All Fields] OR "fracture s"[All Fields] OR "fractures, bone"[MeSH Terms] OR ("fractures"[All Fields] AND "bone"[All Fields]) OR "bone fractures"[All Fields] OR "fracture"[All Fields] OR "fractured"[All Fields] OR "fractures"[All Fields] OR "fracturing"[All Fields]))) |
| Embase | '('non insulin dependent diabetes mellitus'/exp OR 'niddm (non insulin dependent diabetes mellitus)' OR 't2dm' OR 'tiidm' OR 'adult onset diabetes' OR 'adult onset diabetes mellitus' OR 'diabetes mellitus type 2' OR 'diabetes mellitus type ii' OR 'diabetes mellitus, maturity onset' OR 'diabetes mellitus, non insulin dependent' OR 'diabetes mellitus, non-insulin-dependent' OR 'diabetes mellitus, type 2' OR 'diabetes mellitus, type ii' OR 'diabetes type 2' OR 'diabetes type ii' OR 'diabetes, adult onset' OR 'dm 2' OR 'insulin independent diabetes' OR 'insulin independent diabetes mellitus' OR 'ketosis resistant diabetes mellitus' OR 'maturity onset diabetes' OR 'maturity onset diabetes mellitus' OR 'niddm' OR 'non insulin dependent (type 2) diabetes mellitus' OR 'non insulin dependent diabetes' OR 'non insulin dependent diabetes mellitus' OR 'non-insulin-dependent diabetes mellitus' OR 'noninsulin dependent (type 2) diabetes mellitus' OR 'noninsulin dependent diabetes' OR 'noninsulin dependent diabetes mellitus' OR 'type 2 (insulin independent) diabetes' OR 'type 2 diabetes' OR 'type 2 diabetes mellitus' OR 'type ii diabetes' OR 'type ii diabetes mellitus') AND ('sodium glucose cotransporter 2 inhibitor'/exp OR 'sglt2 inhibitor' OR 'sglt2 inhibitors' OR 'gliflozin' OR 'gliflozin derivative' OR 'gliflozins' OR 'sodium dependent glucose cotransporter 2 inhibitor' OR 'sodium glucose co-transporter 2 inhibitor' OR 'sodium glucose cotransporter 2 inhibitor' OR 'sodium-glucose transporter 2 inhibitors' OR 'glucagon like peptide 1 receptor agonist'/exp OR 'glp 1 agonist' OR 'glp 1 receptor agonist' OR 'glucagon like peptide 1 agonist' OR 'glucagon like peptide 1 receptor agonist' OR 'glucagon like peptide 1 receptor stimulating agent' OR 'glucagon-like peptide-1 receptor agonists' OR 'incretin mimetics' OR 'long acting glp 1 agonist' OR 'long acting glp 1 receptor agonist' OR 'long acting glucagon like peptide 1 agonist' OR 'long acting glucagon like peptide 1 receptor agonist' OR 'dipeptidyl peptidase iv inhibitor'/exp OR 'dpp 4 inhibitor' OR 'dpp iv inhibitor' OR 'dipeptidyl peptidase 4 inhibitor' OR 'dipeptidyl peptidase iv inhibitor' OR 'dipeptidyl peptidase iv inhibitors' OR 'dipeptidyl-peptidase iv inhibitors' OR 'dipeptidylpeptidase 4 inhibitor' OR 'dipeptidylpeptidase iv inhibitor' OR 'gliptin' OR 'gliptins' OR 'bromocriptine'/exp OR '10 bromo n [2 hydroxy 7 (2 methylpropyl) 5, 8 dioxo 4 (propan 2 yl) 3 oxa 6, 9 diazatricyclo [7.3.0.0 (2, 6)] dodecan 4 yl] 6 methyl 6, 11 diazatetracyclo [7.6.1.0 (2, 7) .0 (12, 16)] hexadeca 1 (16), 2, 9, 12, 14 pentaene 4 carboxamide' OR '2 bromo 12` hydroxy 2` (1 methylethyl) 5` (2 methylpropyl) 3`, 6`, 18 trioxoergotaman' OR '2 bromo 12` hydroxy 2` (1 methylethyl) 5` (2 methylpropyl) ergotaman 3`, 6`, 18 trione' OR '2 bromo 12` hydroxy 5` isobutyl 2` isopropyl 3`, 6`, 18 trioxoergotaman' OR '2 bromo alpha ergocriptine' OR '2 bromo alpha ergocryptin' OR '2 bromo alpha ergocryptine' OR '2 bromoergocryptine' OR '5 bromo n (10b hydroxy 5 isobutyl 2 isopropyl 3, 6 dioxooctahydro 2h oxazolo [3, 2 a] pyrrolo [2, 1 c] pyrazin 2 yl) 7 methyl 4, 6, 6a, 7, 8, 9 hexahydroindolo [4, 3 fg] quinoline 9 carboxamide' OR '5 bromo n [2 hydroxy 7 (2 methylpropyl) 5, 8 dioxo 4 (2 propanyl) 3 oxa 6, 9 diazatricyclo [7.3.0.0 (2, 6) ] 4 dodecanyl] 7 methyl 6, 6a, 8, 9 tetrahydro 4h indolo [4, 3 fg] quinoline 9 carboxamide' OR '5 bromo n [2 hydroxy 7 (2 methylpropyl) 5, 8 dioxo 4 propan 2 yl 3 oxa 6, 9 diazatricyclo [7.3.0.0 (2, 6)] dodecan 4 yl] 7 methyl 6, 6a, 8, 9 tetrahydro 4h indolo [4, 3 fg] quinoline 9 carboxamide' OR 'antipark' OR 'bromed' OR 'bromocriptin' OR 'bromocriptine' OR 'bromocryptine' OR 'bromoergocriptine' OR 'bromoergocryptine' OR 'bromokin' OR 'bromopar' OR 'cb 154' OR 'cb154' OR 'ergobromocriptine' OR 'ergoset' OR 'serocryptin' OR 'meglitinide'/exp OR '4 [2 (5 chloro 2 methoxybenzamido) ethyl] benzoic acid' OR 'hb 699' OR 'hb699' OR 'meglitinide' OR 'n (4 carboxyphenethyl) 5 chloro 2 methoxybenzamide' OR 'biguanide derivative'/exp OR 'biguanide derivative' OR 'biguanides' OR 'sulfonylurea'/exp OR 'sulfonurea' OR 'sulfonyl urea' OR 'sulfonylcarbamide' OR 'sulfonylurea' OR 'sulphonurea' OR 'sulphonylurea' OR 'metformin'/exp OR '1, 1 dimethylbiguanide' OR 'anj 900' OR 'anj900' OR 'apophage' OR 'aron' OR 'benofomin' OR 'dabex' OR 'denkaform' OR 'deson' OR 'dextin' OR 'diabetase' OR 'diabetase s' OR 'diabetformin' OR 'diabetmin' OR 'diabetmin retard' OR 'diabetosan' OR 'diabex' OR 'diafat' OR 'diaformin' OR 'diaformina' OR 'diaformina lp' OR 'diametin' OR 'diamin' OR 'dianben' OR 'diformin' OR 'diformin retard' OR 'dimefor' OR 'dimethylbiguanide' OR 'dimethyldiguanide' OR 'dmgg' OR 'dybis' OR 'efb 0027' OR 'efb0027' OR 'eraphage' OR 'espa-formin' OR 'euform retard' OR 'fluamine' OR 'flumamine' OR 'fornidd' OR 'fortamet' OR 'glafornil' OR 'glibudon' OR 'glifage' OR 'gliguanid' OR 'glucaminol' OR 'glucofage' OR 'glucofago' OR 'glucoform' OR 'glucoformin' OR 'glucohexal' OR 'glucoless' OR 'glucomet' OR 'glucomin' OR 'glucomine' OR 'gluconil' OR 'glucophage' OR 'glucophage forte' OR 'glucophage retard' OR 'glucophage sr' OR 'glucophage xr' OR 'glucophage xr extended release' OR 'glucophage-mite' OR 'glucostop' OR 'glucotika' OR 'gludepatic' OR 'glufor' OR 'gluformin' OR 'glukophage' OR 'glumeformin' OR 'glumet' OR 'glumetza' OR 'glupa' OR 'glustress' OR 'glyciphage' OR 'glycomet' OR 'glycon' OR 'glycoran' OR 'glyformin' OR 'glymet' OR 'haurymellin' OR 'hipoglucin' OR 'i-max' OR 'islotin' OR 'jesacrin' OR 'juformin' OR 'la 6023' OR 'la6023' OR 'lyomet (drug)' OR 'maformin' OR 'meglucon' OR 'meguan' OR 'melbin' OR 'melformin' OR 'mellittin' OR 'merckformin' OR 'mescorit' OR 'metaformin' OR 'metfogamma' OR 'metfoliquid geriasan' OR 'metforal' OR 'metformax' OR 'metformin' OR 'metformin hydrochloride' OR 'metformina' OR 'metformine' OR 'metformine hcl' OR 'methformin' OR 'metiguanide' OR 'metomin' OR 'metphormin' OR 'miformin' OR 'n` dimethylguanylguanide' OR 'n` dimethylguanylguanidine' OR 'n`, n` dimethyldiguanide' OR 'n, n dimethyl biguanidine' OR 'n, n dimethylbiguanide' OR 'n, n dimethylbiguanide retard' OR 'n, n dimethylbiguanidine' OR 'n, n dimethyldiguanide' OR 'n, n dimethylguanylguanidine' OR 'neoform' OR 'newmet' OR 'nndg' OR 'reglus-500' OR 'riomet' OR 'riomet er' OR 'risidon' OR 'rudimet' OR 'siamformet' OR 'siofor' OR 'thiabet' OR 'vimetrol' OR 'walaphage' OR '2,4 thiazolidinedione derivative'/exp OR '2,4 thiazolidinedione derivative' OR 'thiazolidine 2, 4 dione derivative' OR 'thiazolidinedione' OR 'thiazolidinedione derivative' OR 'thiazolidinediones') AND ('fracture'/exp OR 'bone cement fracture' OR 'bone fracture' OR 'bone fractures' OR 'broken bone' OR 'broken bones' OR 'closed fracture' OR 'closed fractures' OR 'fracture' OR 'fractured bone' OR 'fractures' OR 'fractures, bone' OR 'fractures, closed' OR 'skeleton fracture' OR 'unstable fracture' OR 'bone density'/exp OR 'bone density' OR 'bone mineral density' OR 'density, bone' OR 'osseous density') AND ('randomized controlled trial'/exp OR 'controlled trial, randomized' OR 'randomised controlled study' OR 'randomised controlled trial' OR 'randomized controlled study' OR 'randomized controlled trial' OR 'trial, randomized controlled') |

Table S3. Baseline characteristics

| Author | Year | Register number | sample size | Study design | Patient | Phase | Treatment | | | | | | Control | | | | | | Medication duration week | Duration of T2DM (year) | Outcome | Reference |
| --- | --- | --- | --- | --- | --- | --- | --- | --- | --- | --- | --- | --- | --- | --- | --- | --- | --- | --- | --- | --- | --- | --- |
|  |  |  |  |  |  |  | Intervention | Age(year) | Male/Female (%) | BMI(kg/m2) | HbA1c (%) | Total patients | Comparison | Age(year) | Male/Female (%) | BMI(kg/m2) | HbA1c (%) | Total patients |  |  |  |  |
| Heine | 2005 | NCT00082381 | 549 | RCT | T2DM | 3 | Exenatide | 59.8±8.8 | 155/127 | 31.4±4.4 | 8.2±1.0 | 282 | Insulin | 58.0±9.5 | 151/116 | 31.3±4.6 | 8.3±1.0 | 267 | 26 weeks | 9.55 | Fracture | (Heine *et al.*, 2005) |
| Aschner | 2006 | NCT00087516 | 491 | RCT | T2DM | 3 | Sitagliptin | 53.4±9.5 | 136/102 | 30.3±5.2 | 8.0±0.9 | 238 | Placebo | 54.3±10.1 | 130/123 | 30.8±5.5 | 8.0±0.8 | 253 | 24 weeks | 4.4 | Fracture | (Aschner *et al.*, 2006) |
| Kahn | 2006 | NCT00279045 | 2910 | RCT | T2DM | 3 | Rosiglitazone | 56.3±10.0 | 811/645 | 32.2±6.7 | 7.36±0.93 | 1456 | Metformin | 57.9±9.9 | 864/590 | 32.1±6.1 | 7.36±0.93 | 1454 | 260 weeks | 4 | Fracture | (Kahn *et al.*, 2006) |
| Rosenstock | 2006 | NCT00086502 | 350 | RCT | T2DM | 3 | Sitagliptin | 55.6±10.4 | 93/82 | 32.0±5.2 | 8.1±0.8 | 175 | Placebo | 56.9±11.1 | 103/75 | 31.0±5.0 | 8.0±0.8 | 175 | 24 weeks | 6.1 | Fracture | (Rosenstock *et al.*, 2006) |
| Fonseca | 2007 | NCT00099931 | 296 | RCT | T2DM | 3 | Vildagliptin | 59.6±10.3 | 69/75 | 33.3±5.2 | 8.4±1.0 | 144 | Placebo | 58.9±10.8 | 83/69 | 32.9±5.9 | 8.4±1.1 | 152 | 24 weeks | 14.65 | Fracture | (Fonseca *et al.*, 2007) |
| Goldstein | 2007 | NCT00103857 | 353 | RCT | T2DM | 3 | Sitagliptin | 53.3±10.2 | 93/86 | 31.2±5.9 | 8.87±0.99 | 175 | Metformin | 53.4±10.2 | 89/93 | 32.1±6.8 | 8.90±1.00 | 178 | 24 weeks | 4.5 | Fracture | (Goldstein *et al.*, 2007) |
| Nauck | 2007 | NCT00082407 | 501 | RCT | T2DM | 3 | Exenatide | 59±9.0 | 206/47 | 30.6±4.0 | 8.6±1.0 | 253 | Insulin | 58±9.0 | 197/51 | 30.2±4.2 | 8.6±1.1 | 248 | 52 weeks | 9.9 | Fracture | (Nauck *et al.*, 2007) |
| NCT00086515 | 2007 | NCT00086515 | 701 | RCT | T2DM | 3 | Sitagliptin | 54.4±10.4 | 259/205 | ND | 8.0±0.8 | 464 | Sulfonylurea | 54.7±9.7 | 141/96 | ND | 8.0±0.8 | 237 | 24 weeks | ND | Fracture | (NCT00086515, 2007) |
| NCT00116831 | 2008 | NCT00116831 | 668 | RCT | T2DM | 3 | Rosiglitazone | 61.8±8.35 | 233/98 | 28 | ND | 331 | Sulfonylurea | 60.2±9.05 | 221/116 | 29 | ND | 337 | 78 weeks | 4.74 | Fracture | (NCT00116831, 2008) |
| NCT00313313 | 2008 | NCT00313313 | 515 | RCT | T2DM | 3 | Saxagliptin | 55.36±9.58 | 113/135 | ND | 8.36±0.057 | 248 | Placebo | 55.06±10.69 | 123/144 | ND | 8.44±0.055 | 267 | 24 weeks | ND | Fracture | (NCT00313313, 2008) |
| NCT00367055 | 2008 | NCT00367055 | 84 | RCT | T2DM | 4 | Rosiglitazone | 58.3±8.4 | 32/11 | ND | 7.5±0.55 | 43 | Gliclazide | 58.1±8.0 | 25/16 | ND | 7.3±0.55 | 41 | 54 weeks | ND | Fracture | (NCT00367055, 2008) |
| NCT00372060 | 2008 | NCT00372060 | 134 | RCT | T2DM | 3 | Sitagliptin | 57.8±9.9 | 38/28 | ND | 7.7±0.9 | 66 | Placebo | 59.0±9.2 | 49/19 | ND | 7.6±0.8 | 68 | 12 weeks | ND | Fracture | (NCT00372060, 2008) |
| Nissen | 2008 | NCT00225277 | 543 | RCT | T2DM | 3 | Sulfonylurea | 59.7±9.1 | 180/93 | 32.0±5.2 | ND | 273 | Pioglitazone | 60.0±9.4 | 186/84 | 32.1±5.3 | ND | 270 | 172 weeks | 70.5 (median) | Fracture | (Nissen *et al.*, 2008) |
| Pratley | 2008 | NCT00286468 | 302 | RCT | T2DM | 3 | Alogliptin | 56.5±11.1 | 111/92 | 30.2±4.8 | ND | 203 | Placebo | 57.1±10.0 | 51/48 | 30.0±5.3 | ND | 99 | 26 weeks | 7.75 | Fracture | (Pratley *et al.*, 2009) |
| Raz | 2008 | NCT00337610 | 190 | RCT | T2DM | 3 | Sitagliptin | 53.6±9.5 | 29/47 | 30.1±4.4 | 9.3±0.9 | 96 | Placebo | 56.1±9.5 | 39/55 | 30.4±5.3 | 9.1±0.8 | 94 | 30 weeks | 7.8 | Fracture | (Raz *et al.*, 2008) |
| Scherbaum | 2008 | NCT00101712 | 306 | RCT | T2DM | 3 | Vildagliptin | 63.3±10.2 | 93/63 | 30.4±4.9 | 6.7±0.4 | 156 | Placebo | 62.8±11.0 | 89/61 | 30.4±4.9 | 6.8±0.4 | 150 | 52 weeks | 2.6 | Fracture | (Scherbaum *et al.*, 2008) |
| Bosi | 2009 | NCT00468039 | 594 | RCT | T2DM | 3 | Vildagliptin | 53.5±10.95 | 180/120 | 31.26±4.82 | 8.68±1.02 | 300 | Metformin | 52.4±10.71 | 171/123 | 31.31±4.58 | 8.62±0.93 | 294 | 24 weeks | 25.87 | Fracture | (Bosi *et al.*, 2009) |
| Buse | 2009 | NCT00518882 | 464 | RCT | T2DM | 3 | Liraglutide | 56.3±9.8 | 114/119 | 32.9±5.5 | 8.2±1.0 | 233 | Exenatide | 57.1±10.8 | 127/104 | 32.9±5.7 | 8.1±1.0 | 231 | 78 weeks | 8.2 | Fracture | (Buse *et al.*, 2009) |
| Garber | 2009 | NCT00294723 | 495 | RCT | T2DM | 3 | Liraglutide | 52±10.8 | 121/126 | 32.8±6.3 | 8.3±1.1 | 247 | Sulfonylurea | 53.4±10.9 | 133/115 | 33.2±5.6 | 8.4±1.2 | 248 | 52 weeks | 5.4 | Fracture | (Garber *et al.*, 2009) |
| Home | 2009 | NCT00379769 | 2222 | RCT | T2DM | 3 | Rosiglitazone | 57±8.0 | 601/516 | 32.8±5.0 | 7.8±0.7 | 1117 | Sulfonylurea | 57.2±8.1 | 584/521 | 32.7±5.2 | 7.8±0.7 | 1105 | 260 weeks | 6.2 | Fracture | (Home *et al.*, 2009) |
| Jadzinsky | 2009 | NCT00327015 | 663 | RCT | T2DM | 3 | Saxagliptin | 52.1±10.2 | 169/166 | 30.2±4.9 | 9.6±1.3 | 335 | Metformin | 51.8±10.7 | 163/165 | 30.2±4.9 | 9.4±1.3 | 328 | 24 weeks | 1.7 | Fracture | (Jadzinsky *et al.*, 2009) |
| Nauck | 2009 | NCT00318461 | 484 | RCT | T2DM | 3 | Liraglutide | 56.8±9.4 | 142/100 | 30.9±4.6 | 8.3±0.9 | 242 | Sulfonylurea | 57.3±8.8 | 139/103 | 31.2±4.6 | 8.4±0.9 | 242 | 26 weeks | 7.7 | Fracture | (Nauck *et al.*, 2009) |
| NCT00374907 | 2009 | NCT00374907 | 36 | RCT | T2DM | 3 | Saxagliptin | 58 | 8/12 | 33.36 | ND | 20 | Placebo | 55 | 6/10 | 32.31 | ND | 16 | 104 weeks | ND | Fracture | (NCT00374907, 2009) |
| NCT00601250 | 2009 | NCT00601250 | 700 | RCT | T2DM | 3 | Linagliptin | 56.5±10.1 | 278/245 | 29.85±4.84 | 8.09±0.86 | 523 | Placebo | 56.6±10.9 | 101/76 | 30.05±5.01 | 8.02±0.88 | 177 | 24 weeks | ND | Fracture | (NCT00601250, 2009) |
| NCT00736879 | 2009 | NCT00736879 | 136 | RCT | T2DM | 3 | Dapagliflozin | 51.3±11.51 | 32/36 | ND | 7.94±1.029 | 68 | Placebo | 53.5±11.08 | 37/31 | ND | 7.8±1.117 | 68 | 12 weeks | ND | Fracture | (NCT00736879, 2009) |
| Perez | 2009 | NCT00727857 | 399 | RCT | T2DM | 3 | Pioglitazone | 54.0±12.1 | 66/123 | 31.2±5.5 | 8.69±0.07 | 189 | Metformin | 53.7±12.0 | 98/112 | 30.9±5.6 | 8.65±0.07 | 210 | 24 weeks | ND | Fracture | (Perez *et al.*, 2009) |
| Tolman | 2009 | NCT00494312 | 2097 | RCT | T2DM | 4 | Pioglitazone | 54 | 601/450 | 32.5±6.1 | 9.5±2.0 | 1051 | Glibenclamide | 55 | 581/465 | 32.5±6.0 | 9.5±2.0 | 1046 | 152 weeks | 5.8 | Fracture | (Tolman *et al.*, 2009) |
| Vilsbøll | 2009 | NCT00395343 | 641 | RCT | T2DM | 3 | Sitagliptin | 58.3±9.1 | 157/165 | 31±5 | 8.7±0.9 | 322 | Placebo | 57.2±9.3 | 169/150 | 31±5 | 8.6±0.9 | 319 | 24 weeks | 12.5 | Fracture | (Vilsbøll *et al.*, 2010) |
| Aschner | 2010 | NCT00449930 | 894 | RCT | T2DM | 3 | Sitagliptin | 56.3±10.7 | 217/238 | 30.7±4.7 | 7.2±0.7 | 455 | Metformin | 55.7±10.3 | 194/245 | 30.9±4.9 | 7.2±0.7 | 439 | 24 weeks | 2.35 | Fracture | (Aschner *et al.*, 2010) |
| Bailey | 2010 | NCT00528879 | 274 | RCT | T2DM | 3 | Dapagliflozin | 55.0±9.3 | 70/67 | 31.6±4.8 | 7.99±0.90 | 137 | Placebo | 53.7±10.3 | 76/61 | 31.8±5.3 | 8.12±0.96 | 137 | 78 weeks | 5.9 | Fracture | (Bailey *et al.*, 2010) |
| Berberoglu | 2010 | NCT00440375 | 49 | RCT | T2DM | 4 | Rosiglitazone | 60.7±6.4 | 0/26 | 35.0±4.1 | 6.3±0.5 | 26 | Placebo | 59.8±7.6 | 0/23 | 34.6±3.3 | 6.3±0.5 | 23 | 104 weeks | ND | BMD/Fracture | (Berberoglu *et al.*, 2010) |
| Gill | 2010 | NCT00516074 | 54 | RCT | T2DM | 3 | Exenatide | 57±11 | 19/19 | 29.5±3.4 | 7.5±0.9 | 28 | Placebo | 54±10 | 11/15 | 30.1±3.9 | 7.1±0.7 | 26 | 12 weeks | 6.5 | Fracture | (Gill *et al.*, 2010) |
| Gruntmanis | 2010 | NCT00424762 | 111 | RCT | T2DM | 4 | Rosiglitazone | 56.7±8.8 | 33/23 | 33.9±6.4 | 7.6±1.8 | 56 | Placebo | 55.8±8.3 | 33/22 | 33.5±6.8 | 7.6±1.7 | 55 | 52 weeks | ND | Fracture | (Gruntmanis *et al.*, 2010) |
| Iwamoto | 2010 | NCT00411554 | 319 | RCT | T2DM | 3 | Sitagliptin | 60.8±10.1 | 118/45 | 24.5±3.3 | 7.8±0.9 | 163 | Voglibose | 60.6±10.0 | 94/62 | 24.8±3.6 | 7.8±0.9 | 156 | 12 weeks | 5.2 | Fracture | (Iwamoto *et al.*, 2010) |
| Kanazawa | 2010 | UMIN000001997 | 45 | RCT | T2DM | ND | Pioglitazone | 67±10 | 14/8 | 22±2.3 | 7.9±1.7 | 22 | Metformin | 66±10 | 13/10 | 24.9±3.7 | 7.9±1.3 | 23 | 52 weeks | 13 | Fracture/BMD | (Kanazawa *et al.*, 2010) |
| NCT00121667 | 2010 | NCT00121667 | 371 | RCT | T2DM | 3 | Saxagliptin | 54.66±10.08 | 83/109 | 31.68±5.15 | 8.08±0.07 | 192 | Placebo | 54.76±10.18 | 96/83 | 31.57±4.80 | 8.06±0.07 | 179 | 102 weeks | ND | Fracture | (NCT00121667, 2010) |
| NCT00434954 | 2010 | NCT00434954 | 894 | RCT | T2DM | 3 | Exenatide | 57.2±10.03 | 108/73 | 33.4±4.23 | 7.89±0.822 | 181 | Insulin | 56.9±9.94 | 96/77 | 32.9±4.37 | 7.88±0.918 | 713 | 26 weeks | 5 | Fracture | (NCT00434954, 2010) |
| NCT01453049 | 2010 | NCT01453049 | 73 | RCT | T2DM | 3 | Rosiglitazone | 53.6±10.54 | 22/13 | ND | ND | 35 | Placebo | 52.2±8.57 | 15/23 | ND | ND | 38 | 24 weeks | ND | Fracture | (NCT01453049, 2010) |
| Partley | 2010 | NCT00700817 | 440 | RCT | T2DM | 3 | Liraglutide | 55.0±9.1 | 116/105 | 33.1±5.1 | 8.4±0.7 | 221 | Sitagliptin | 55.0±9.0 | 120/99 | 32.6±5.4 | 8.5±0.7 | 219 | 26 weeks | 6.35 | Fracture | (Pratley *et al.*, 2010) |
| Prato | 2010 | NCT00621140 | 503 | RCT | T2DM | 3 | Linagliptin | 56.4±10.1 | 164/172 | 29.04±4.80 | 8.0±0.05 | 336 | Placebo | 54.4±10.3 | 79/88 | 29.08±4.84 | 8.0±0.07 | 167 | 24 weeks | ND | Fracture | (Del Prato *et al.*, 2016) |
| Rosenstock | 2010 | NCT00395512 | 327 | RCT | T2DM | 3 | Alogliptin | 52.6±10.38 | 76/88 | 31.61±5.587 | 8.80±0.988 | 164 | Pioglitazone | 51.5±10.72 | 90/73 | 30.87±4.938 | 8.76±1.005 | 163 | 26 weeks | 3.215 | Fracture | (Rosenstock *et al.*, 2010) |
| Seck | 2010 | NCT00094770 | 504 | RCT | T2DM | 3 | Sitagliptin | 57.6±8.5 | 142/106 | 30.9±4.8 | 7.3±0.6 | 248 | Sulfonylurea | 57.0±9.1 | 161/95 | 31.3±5.0 | 7.3±0.7 | 256 | 104 weeks | 5.75 | Fracture | (Seck *et al.*, 2010) |
| Arechavaleta | 2011 | NCT00701090 | 1035 | RCT | T2DM | 3 | Sitagliptin | 56.3±9.7 | 284/232 | 29.7±4.5 | 7.5±0.7 | 516 | Sulfonylurea | 56.2±10.1 | 279/240 | 30.2±4.4 | 7.5±0.8 | 519 | 30 weeks | 6.8 | Fracture | (Arechavaleta *et al.*, 2011) |
| Barzilai | 2011 | NCT00305604 | 224 | RCT | T2DM | 3 | Sitagliptin | 71.6±6.1 | 49/55 | 31.1±7.2 | 7.8±0.7 | 120 | Placebo | 72.1±6.0 | 48/54 | 30.8±5.9 | 7.8±0.8 | 104 | 24 weeks | 7.1 | Fracture | (Barzilai *et al.*, 2011) |
| Borges | 2011 | NCT00386100 | 678 | RCT | T2DM | 4 | Avandamet | 50.7±10.49 | 184/160 | 32.2±6.8 | 8.64 | 344 | Metformin | 51.5±10.52 | 176/158 | 33.1±7.1 | 8.64 | 334 | 80 weeks | 2.45 | BMD | (Borges *et al.*, 2011) |
| Bosi | 2011 | NCT00432276 | 803 | RCT | T2DM | 3 | Alogliptin | 54.3±9.86 | 210/194 | 31.5±5.25 | 8.2±0.86 | 404 | Placebo | 55.9±9.94 | 204/195 | 31.6±5.18 | 8.1±0.83 | 399 | 52 weeks | 7.2 | Fracture | (Bosi *et al.*, 2011) |
| Hollander | 2011 | NCT00295633 | 379 | RCT | T2DM | 3 | Saxagliptin | 54.9±9.7 | 106/89 | 30.0±5.8 | 8.3±1.1 | 195 | Placebo | 54.0±10.1 | 85/99 | 30.3±5.8 | 8.2±1.1 | 184 | 76 weeks | 5.2 | Fracture | (Hollander *et al.*, 2011) |
| Nauck | 2011 | NCT00660907 | 801 | RCT | T2DM | 3 | Dapagliflozin | 58.1±9.37 | 221/179 | 31.71±5.1 | 7.69±0.855 | 400 | Sulfonylurea | 58.6±9.8 | 220/181 | 31.23±5.053 | 7.74±0.886 | 401 | 52 weeks | 6.5 | Fracture | (Nauck *et al.*, 2011) |
| NCT00800683 | 2011 | NCT00800683 | 133 | RCT | T2DM | 3 | Linagliptin | 64.0±10.9 | 45/23 | 32.3±5.9 | 8.2±1.1 | 68 | Placebo | 64.9±9.6 | 35/30 | 31.7±5.9 | 8.2±0.9 | 65 | 52 weeks | ND | Fracture | (NCT00800683, 2011) |
| NCT00915772 | 2011 | NCT00915772 | 395 | RCT | T2DM | 3 | Linagliptin | 56.0±10.7 | 120/105 | 29.06±4.95 | 7.64±1.04 | 225 | Metformin | 55.6±10.7 | 93/77 | 29.33±5.22 | 7.48±0.99 | 170 | 54 weeks | ND | Fracture | (NCT00915772, 2011) |
| Nowichki | 2011 | NCT00614939 | 170 | RCT | T2DM | 3 | Saxagliptin | 66.8±8.3 | 32/53 | 31.2±6.1 | 8.5±1.2 | 85 | Placebo | 66.2±9.1 | 41/44 | 30.2±6.8 | 8.1±1.1 | 85 | 12 weeks | 17 | Fracture | (Nowicki *et al.*, 2011) |
| Owens | 2011 | NCT00602472 | 1055 | RCT | T2DM | 3 | Linagliptin | 58.3±9.9 | 371/421 | 28.4±4.8 | ND | 792 | Placebo | 57.6±9.7 | 127/136 | 28.2±4.5 | ND | 263 | 24 weeks | ND | Fracture | (Owens *et al.*, 2011) |
| Reasner | 2011 | NCT00482729 | 1246 | RCT | T2DM | 3 | Sitagliptin | 49.4±10.5 | 353/272 | 32.9±7.2 | 9.9±1.8 | 625 | Placebo | 50.0±10.5 | 356/265 | 33.7±7.8 | 9.8±1.8 | 621 | 44 weeks | 3.35 | Fracture | (Reasner *et al.*, 2011) |
| Strojeck | 2011 | NCT00680745 | 299 | RCT | T2DM | 3 | Dapagliflozin | 59.9±10.14 | 77/77 | 30.01±5.120 | 8.11±0.75 | 154 | Placebo | 60.3±10.16 | 71/74 | 29.84±5.182 | 8.15±0.74 | 145 | 24 weeks | 7.5 | Fracture | (Strojek *et al.*, 2011) |
| Yang | 2011 | NCT00661362 | 570 | RCT | T2DM | 3 | Saxagliptin | 53.8±10.4 | 136/147 | 26.3±3.6 | 7.9±0.8 | 283 | Placebo | 54.4±10.1 | 139/148 | 26.1±3.5 | 7.9±0.8 | 287 | 24 weeks | 5.1 | Fracture | (Yang *et al.*, 2011) |
| Yang | 2011 | NCT00614120 | 465 | RCT | T2DM | 3 | Liraglutide | 52.7±9.1 | 126/108 | 25.8±3.8 | 8.6±1.1 | 234 | Sulfonylurea | 53.6±9.7 | 135/96 | 25.3±3.7 | 8.5±1.1 | 231 | 16 weeks | 7.5 | Fracture | (NCT00614120, 2009) |
| Barnett | 2012 | NCT00757588 | 455 | RCT | T2DM | 3 | Saxagliptin | 57.2±9.43 | 120/184 | 32.6±5.65 | 8.7±0.9 | 304 | Placebo | 57.3±9.27 | 68/83 | 31.8±4.76 | 8.6±0.86 | 151 | 24 weeks | 12 | Fracture | (Barnett *et al.*, 2012) |
| DeFronzo | 2012 | NCT00328627 | 257 | RCT | T2DM | 3 | Alogliptin | 53.1±9.6 | 67/61 | 31.0±5.1 | 8.6±0.7 | 128 | Placebo | 55.2±9.9 | 61/68 | 30.6±4.8 | 8.5±0.6 | 129 | 26 weeks | 6.1 | Fracture | (DeFronzo *et al.*, 2012) |
| Fonseca | 2012 | NCT00688701 | 241 | RCT | T2DM | 3 | Lixisenatide | 53.8±10.9 | 63/56 | 31.7±6.6 | 8.07±0.9 | 119 | Placebo | 54.1±11.0 | 60/62 | 31.8±6.7 | 8.07±0.9 | 122 | 12 weeks | 1.25 | Fracture | (Fonseca *et al.*, 2012) |
| Frederich | 2012 | NCT00316082 | 146 | RCT | T2DM | 3 | Saxagliptin | 55.1±10.35 | 33/39 | 29.6±5.37 | 7.9±0.9 | 72 | Placebo | 55.6±10.32 | 35/39 | 31.1±4.54 | 7.8±1.0 | 74 | 24 weeks | 1.8 | Fracture | (Frederich *et al.*, 2012) |
| Gallwitz | 2012 | NCT00622284 | 1551 | RCT | T2DM | 3 | linagliptin | 59.8±9.4 | 462/314 | 30.2±4.8 | 7.7±0.9 | 776 | Sulfonylurea | 59.8±9.4 | 471/304 | 30.3±4.6 | 7.7±0.9 | 775 | 52 weeks | ND | Fracture | (Gallwitz *et al.*, 2012b) |
| Gallwitz | 2012 | NCT00359762 | 977 | RCT | T2DM | 3 | Exenatide | 56±10.0 | 272/218 | 32.6±4.2 | 7.5±0.7 | 490 | Sulfonylurea | 56±9.1 | 252/235 | 32.3±3.9 | 7.4±0.7 | 487 | 234 weeks | 5.6 | Fracture | (Gallwitz *et al.*, 2012a) |
| Henry | 2012 | NCT00643851 | 404 | RCT | T2DM | 3 | Dapagliflozin | 52.3±10.2 | 92/111 | ND | 9.1±1.4 | 203 | Metformin | 51.8±9.8 | 95/106 | ND | 9.2±1.3 | 201 | 24 weeks | 1.6 | Fracture | (Henry *et al.*, 2012) |
| Inagaki | 2012 | NCT00935532 | 427 | RCT | T2DM | 3 | Exenatide | 57.07±10.44 | 142/73 | 26.11±4.03 | 8.51±0.82 | 215 | Insulin | 56.44±11.16 | 148/64 | 26.18±3.77 | 8.50±0.79 | 212 | 26 weeks | 9.03 | Fracture | (Inagaki *et al.*, 2012) |
| Ljunggren | 2012 | NCT00855166 | 180 | RCT | T2DM | 3 | Dapagliflozin | 60.6±8.2 | 49/40 | 32.1±3.9 | 7.19±0.44 | 89 | Placebo | 60.8±6.9 | 51/40 | 31.7±3.9 | 7.16±0.53 | 91 | 50 weeks | 5.75 | BMD | (Ljunggren *et al.*, 2012) |
| NCT00484198 | 2012 | NCT00484198 | 411 | RCT | T2DM | 3 | Rivoglitazone | 55.0±10.51 | 132/142 | 29.7±5.63 | 7.7±0.53 | 274 | Placebo | 55.4±12.32 | 67/70 | 30.1±5.43 | 7.7±0.54 | 137 | 26 weeks | 4.95 | Fracture | (NCT00484198, 2012) |
| NCT00856284 | 2012 | NCT00856284 | 1759 | RCT | T2DM | 3 | Alogliptin | 55.5±9.81 | 452/433 | 31.27±5.417 | 7.61±0.606 | 885 | Sulfonylurea | 55.4±9.6 | 441/433 | 31.11±5.320 | 7.60±0.617 | 874 | 108 weeks | 5.45 | Fracture | (NCT00856284, 2012) |
| NCT01204294 | 2012 | NCT01204294 | 146 | RCT | T2DM | 3 | Linagliptin | 62.0±10.4 | 55/30 | ND | 7.87±0.75 | 85 | Metformin | 59.3±10.3 | 42/19 | ND | 8.07±1.04 | 61 | 52 weeks | ND | Fracture | (NCT01204294, 2012) |
| NCT01296412 | 2012 | NCT01296412 | 653 | RCT | T2DM | 3 | Sitagliptin | 56.9±10.0 | 178/148 | ND | 8.2±1.1 | 326 | Liraglutide | 57.6±10.8 | 180/147 | ND | 8.1±0.9 | 327 | 26 weeks | ND | Fracture | (NCT01296412, 2012) |
| NCT01545388 | 2012 | NCT01545388 | 204 | RCT | T2DM | 3 | Metformin | 59.46±9.61 | 86/52 | ND | 7.6±0.83 | 138 | Placebo | 60.05±9.30 | 38/28 | ND | 7.53±0.77 | 66 | 26 weeks | ND | Fracture | (NCT01545388, 2012) |
| Punthakee | 2012 | NCT00879970 | 791 | RCT | T2DM | 4 | Pioglitazone | 66.3±6.6 | 225/167 | 30.6±5.4 | 7.4±0.8 | 392 | Rosiglitazone | 66.5±6.4 | 238/161 | 30.7±5.3 | 7.4±0.9 | 399 | 24 weeks | 8.8 | Fracture | (Punthakee *et al.*, 2012) |
| Rosenstock | 2012 | NCT00683878 | 280 | RCT | T2DM | 3 | Dapagliflozin | 53.2±10.9 | 78/63 | ND | 8.40±1.03 | 141 | Placebo | 53.5±11.4 | 71/68 | ND | 8.34±1.00 | 139 | 48 weeks | 5.355 | Fracture | (Rosenstock *et al.*, 2012) |
| Wilding | 2012 | NCT00673231 | 399 | RCT | T2DM | 3 | Dapagliflozin | 59.8±7.64 | 100/102 | 32.95±5.032 | 8.46±0.78 | 202 | Placebo | 58.8±8.61 | 95/98 | 33.14±5.862 | 8.47±0.768 | 197 | 24 weeks | 13.55 | Fracture | (Wilding, 2012) |
| Yoon | 2012 | NCT00397631 | 317 | RCT | T2DM | 3 | Sitagliptin | 51.4±10.0 | 86/78 | 29.7±4.8 | 9.4±1.1 | 164 | Placebo | 52.3±10.0 | 90/63 | 29.9±5.3 | 9.4±1.4 | 153 | 54 weeks | 2.1 | Fracture | (Yoon *et al.*, 2012) |
| Ahrén | 2013 | NCT00712673 | 680 | RCT | T2DM | 3 | Lixisenatide | 54.65 | 212/298 | 32.85 | 8.05 | 510 | Placebo | 55±9.4 | 81/89 | 33.1±6.5 | 8.1±0.9 | 170 | 24 weeks | 3.5 | Fracture | (Ahrén *et al.*, 2013) |
| Barnett | 2013 | NCT01084005 | 241 | RCT | T2DM | 3 | Linagliptin | 74.9±4.4 | 116/46 | 29.6±4.7 | 7.8±0.8 | 162 | Placebo | 74.9±4.2 | 49/30 | 29.8±4.5 | 7.7±0.7 | 79 | 24 weeks | ND | Fracture | (Barnett *et al.*, 2013) |
| Beck | 2013 | NCT00927355 | 10 | RCT | T2DM | ND | Pioglitazone | 52±10 | 1/4 | 30±8 | 7.6±0.5 | 5 | Placebo | 60±6 | 2/3 | 33±10 | 7.6±0.4 | 5 | 26 weeks | ND | BMD | (Beck *et al.*, 2013) |
| Bilezikian | 2013 | NCT00679939 | 225 | RCT | T2DM | 3 | Rosiglitazone | 63.6±6.61 | 0/114 | 31.2±5.86 | 6.8±0.73 | 114 | Metformin | 63.8±6.52 | 0/111 | 31.5±5.79 | 6.8±0.74 | 111 | 76 weeks | 3.6 | BMD/Fracture | (Bilezikian *et al.*, 2013) |
| Bolli | 2013 | NCT00763451 | 321 | RCT | T2DM | 3 | Lixisenatide | 55.4±8.9 | 71/90 | 33.0±5.8 | 8.0±0.9 | 161 | Placebo | 58.2±9.8 | 72/88 | 32.4±5.5 | 8.0±0.8 | 160 | 112 weeks | 6.1 | Fracture | (Bolli *et al.*, 2014) |
| Buse | 2013 | NCT01029886 | 911 | RCT | T2DM | 3 | Exenatide | 57±9.4 | 254/207 | 32.3±5.6 | 8.5±1.0 | 461 | Liraglutide | 57±9.6 | 245/205 | 32.3±5.4 | 8.4±1.0 | 450 | 26 weeks | 8.5 | Fracture | (Buse *et al.*, 2013) |
| Cefalu | 2013 | NCT00968812 | 965 | RCT | T2DM | 3 | Sulfonylurea | 56.3±9.0 | 263/219 | 30.9±5.5 | 7.8±0.8 | 482 | Canagliflozin | 56.3±9.0 | 252/231 | 31.0±5.3 | 7.8±0.8 | 483 | 52 weeks | 6.6 | Fracture | (Cefalu *et al.*, 2013) |
| Dobs | 2013 | NCT00350779 | 262 | RCT | T2DM | 3 | Sitagliptin | 54.4±8.8 | 96/74 | 30.1±6.2 | 8.8±1.0 | 92 | Placebo | 54.8±9.5 | 55/37 | 30.8±5.6 | 8.7±1.0 | 170 | 54 weeks | 9.3 | Fracture | (Dobs *et al.*, 2013) |
| Ferreira | 2013 | NCT00509262 | 423 | RCT | T2DM | 3 | Sitagliptin | 64.2±10.7 | 131/80 | ND | 7.76±0.65 | 211 | Sulfonylurea | 64.2±9.4 | 122/90 | ND | 7.79±0.70 | 212 | 54 weeks | 10.4 | Fracture | (Arjona Ferreira *et al.*, 2013) |
| Fonseca | 2013 | NCT00885352 | 313 | RCT | T2DM | 3 | Sitagliptin | 55.7±8.7 | 97/60 | 29.9±5.2 | 8.8±1.0 | 157 | Placebo | 56.4±9.4 | 98/58 | 30.0±5.2 | 8.7±1.0 | 156 | 26 weeks | 9.8 | Fracture | (Fonseca *et al.*, 2013) |
| Genovese | 2013 | NCT00772174 | 213 | RCT | T2DM | 3 | Pioglitazone | 57±8.6 | 65/45 | 32.4±5.4 | 6.92±0.42 | 110 | Placebo | 57.8±8.2 | 62/41 | 32.6±5.3 | 7.02±0.46 | 103 | 24 weeks | 5.75 | Fracture | (Genovese *et al.*, 2013) |
| Göke | 2013 | NCT00575588 | 858 | RCT | T2DM | 3 | Saxagliptin | 57.5 | 212/216 | 31.5 | 7.7 | 428 | Sulfonylurea | 57.6 | 232/198 | 31.3 | 7.7 | 430 | 52 weeks | 5.5 | Fracture | (Göke *et al.*, 2013) |
| Haring | 2013 | NCT01159600 | 450 | RCT | T2DM | 3 | Empagliflozin | 57.0±9.2 | 113/112 | 28.3±5.4 | 8.07±0.81 | 225 | Placebo | 56.9±9.2 | 112/113 | 27.9±4.9 | 8.15±0.83 | 225 | 24 weeks | ND | Fracture | (Häring *et al.*, 2013) |
| Lavalle-González2013 | 2013 | NCT01106677 | 733 | RCT | T2DM | 3 | Sitagliptin | 55.5±9.6 | 172/194 | 32.0±6.1 | 7.9±0.9 | 366 | Canagliflozin | 55.3±9.2 | 165/202 | 32.4±6.4 | 7.9±0.9 | 367 | 26 weeks | 7 | Fracture | (Lavalle-González *et al.*, 2013) |
| NCT00509236 | 2013 | NCT00509236 | 426 | RCT | T2DM | 3 | Sitagliptin | 60.5±9.1 | 40/24 | ND | 7.8 | 64 | Sulfonylurea | 58.5±9.9 | 37/28 | ND | ND | 65 | 54 weeks | 17.5 | Fracture | (NCT00509236, 2013) |
| NCT01215097 | 2013 | NCT01215097 | 305 | RCT | T2DM | 3 | Linagliptin | 55.1±10.7 | 102/103 | 25.5±3.9 | 7.99±0.83 | 205 | Placebo | 56.5±8.7 | 50/50 | 25.8±4.0 | 8.0±0.8 | 100 | 24 weeks | ND | Fracture | (NCT01215097, 2013) |
| Pinget | 2013 | NCT00763815 | 484 | RCT | T2DM | 3 | Lixisenatide | 56.0±9.5 | 53/47 | 33.7±6.7 | 8.1±0.9 | 323 | Placebo | 55.3±9.5 | 51/49 | 34.4±7.0 | 8.1±0.8 | 161 | 24 weeks | 8.1 | Fracture | (Pinget *et al.*, 2013) |
| Roden | 2013 | NCT01177813 | 452 | RCT | T2DM | 3 | Empagliflozin | 56.2±11.6 | 142/82 | 28.3±5.5 | 7.87±0.88 | 224 | Placebo | 54.9±10.9 | 123/105 | 28.7±6.2 | 7.91±0.78 | 228 | 24 weeks | ND | Fracture | (Roden *et al.*, 2013) |
| Rosenstock | 2013 | NCT00121641 | 193 | RCT | T2DM | 3 | Saxagliptin | 52.72±11.27 | 45/53 | 31.71± 4.71 | 7.8±0.9 | 98 | Placebo | 53.91±12.32 | 47/48 | 30.93±4.26 | 7.9±0.9 | 95 | 24 weeks | 2.3 | Fracture | (Rosenstock *et al.*, 2013a) |
| Rosenstock | 2013 | NCT00707993 | 441 | RCT | T2DM | 3 | Alogliptin | 70.1±4.42 | 102/120 | 29.58±4.348 | 7.5±0.70 | 222 | Sulfonylurea | 69.8±4.07 | 96/123 | 30.02±4.459 | 7.45±0.634 | 219 | 52 weeks | 6.1 | Fracture | (Rosenstock *et al.*, 2013b) |
| Schernthaner | 2013 | NCT01137812 | 755 | RCT | T2DM | 3 | Sitagliptin | 56.7±9.3 | 215/163 | 31.7±6.9 | 8.1±0.9 | 378 | Canagliflozin | 56.6±9.6 | 207/170 | 31.5±6.9 | 8.1±0.9 | 377 | 52 weeks | 9.5 | Fracture | (Schernthaner *et al.*, 2013) |
| Scirica | 2013 | NCT01107886 | 16492 | RCT | T2DM | 4 | Saxagliptin | 65.1±8.5 | 5512/2768 | 31.1±5.5 | 8.0±1.4 | 8280 | Placebo | 65.0±8.6 | 5525/2687 | 31.2±5.7 | 8.0±1.4 | 8212 | 180 weeks | 10.3 | Fracture | (Scirica *et al.*, 2013) |
| Stenlöf | 2013 | NCT01081834 | 387 | RCT | T2DM | 3 | Canagliflozin | 55.1±10.8 | 81/114 | 31.3±6.6 | 8.1±1.0 | 195 | Placebo | 55.7±10.9 | 88/104 | 31.8±6.2 | 8.0±1.0 | 192 | 52 weeks | 4.35 | Fracture | (Stenlöf *et al.*, 2014) |
| White | 2013 | NCT00968708 | 5380 | RCT | T2DM | 3 | Alogliptin | 61±9.96 | 1828/873 | 29.42±5.42 | 8.07 | 2701 | Placebo | 60.7±9.88 | 1823/856 | 29.53±5.758 | 8.05 | 2679 | 178 weeks | 9.16 | Fracture | (White *et al.*, 2013) |
| Wilding | 2013 | NCT01106625 | 313 | RCT | T2DM | 3 | Canagliflozin | 57.4±10.5 | 76/81 | 33.3±6.3 | 8.1±0.9 | 157 | Placebo | 56.8±8.3 | 76/80 | 32.7±6.8 | 8.1±0.9 | 156 | 52 weeks | 9.6 | Fracture | (Wilding *et al.*, 2013) |
| Ahrén | 2014 | NCT00838903 | 609 | RCT | T2DM | 3 | Sitagliptin | 54.3±9.81 | 139/163 | 32.8±5.4 | 8.1±0.8 | 302 | Sulfonylurea | 54.4±9.97 | 158/149 | 32.5±5.5 | 8.1±0.8 | 307 | 156 weeks | 5.9 | Fracture | (Ahrén *et al.*, 2014) |
| Ahrén | 2014 | NCT00838903 | 403 | RCT | T2DM | 3 | Albiglutide | 54.3±10.12 | 135/167 | 32.7±5.6 | 8.1±0.8 | 302 | Placebo | 56.1±10.01 | 50/51 | 32.8±5.4 | 8.2±0.9 | 101 | 156 weeks | 6.35 | Fracture | (Ahrén *et al.*, 2014) |
| Barnett | 2014 | NCT01164501 | 193 | RCT | T2DM | 3 | Empagliflozin | 63.2±8.5 | 60/38 | 32.4±5.4 | 8.1±1.9 | 98 | Placebo | 67±8.6 | 56/39 | 30.8±5.6 | 8.0±0.8 | 95 | 52 weeks | ND | Fracture | (Barnett *et al.*, 2014) |
| Buse | 2014 | NCT00765817 | 259 | RCT | T2DM | 3 | Exenatide | 58.67±8.91 | 70/67 | 33.8±5.8 | 8.32±0.85 | 137 | Placebo | 59.40±9.96 | 78/44 | 33.1±6.2 | 8.50±0.96 | 122 | 30 weeks | 12 | Fracture | (Buse *et al.*, 2014) |
| Diamant | 2014 | NCT00960661 | 510 | RCT | T2DM | 3 | Exenatide | 59.5±9.6 | 128/119 | 32.7±4.7 | 8.3±1.0 | 247 | Insulin | 59.4±9.27 | 133/130 | 32.3±4.7 | 8.2±0.9 | 263 | 44 weeks | 12.35 | Fracture | (Diamant *et al.*, 2014) |
| Forst | 2014 | NCT01106690 | 228 | RCT | T2DM | 3 | Sitagliptin | 58.3±9.56 | 76/39 | 32.5±6.4 | 8.0±1.0 | 115 | Canagliflozin | 56.7±10.36 | 77/36 | 32.3±6.2 | 8.0±0.9 | 113 | 52 weeks | 10.3 | Fracture | (Forst *et al.*, 2014) |
| Gough | 2014 | NCT01336023 | 827 | RCT | T2DM | 3 | Liraglutide | 55.1±9.9 | 208/206 | 31.3±4.8 | 8.3±0.9 | 414 | Insulin | 54.9±9.7 | 200/213 | 31.2±5.3 | 8.3±1.0 | 413 | 26 weeks | 7.1 | Fracture | (Gough *et al.*, 2014) |
| Grey | 2014 | ACTRN12607000610437 | 86 | RCT | T2DM | ND | Pioglitazone | 64 | 21/22 | 31±5.0 | 7.4±3.5 | 43 | Placebo | 63 | 13/20 | 31±5.0 | 7.5±3.2 | 43 | 12 weeks | ND | Fracture/BMD | (Grey *et al.*, 2014) |
| Henry | 2014 | NCT00722371 | 461 | RCT | T2DM | 3 | Sitagliptin | 51 | 112/74 | 31.4±5.7 | 8.7±1.2 | 231 | Pioglitazone | 50.3 | 119/64 | 30.7±5.2 | 8.9±1.1 | 230 | 54 weeks | 4.1 | Fracture | (Henry *et al.*, 2014) |
| Inagaki | 2014 | NCT01413204 | 183 | RCT | T2DM | 3 | Canagliflozin | 58.4±10.4 | 59/31 | 25.59±4.20 | 8.04±0.7 | 90 | Placebo | 58.2±11.9 | 60/33 | 25.85±4.39 | 8.04±0.7 | 93 | 24 weeks | 5.175 | Fracture | (Inagaki *et al.*, 2014) |
| Jabbour | 2014 | NCT00984867 | 447 | RCT | T2DM | 3 | Dapagliflozin | 54.8±10.4 | 127/96 | ND | 7.9±0.8 | 223 | Placebo | 55±10.2 | 118/106 | ND | 8.0±0.8 | 224 | 24 weeks | 5.8 | Fracture | (Jabbour *et al.*, 2014) |
| Ji | 2014 | NCT01095653 | 260 | RCT | T2DM | 3 | Dapagliflozin | 53.0±11.07 | 84/44 | 25.17±3.29 | 8.14±0.74 | 128 | Placebo | 49.9±10.87 | 87/45 | 25.93±3.64 | 8.35±0.95 | 132 | 24 weeks | 1.22 | Fracture | (Ji *et al.*, 2014) |
| Leiter | 2014 | NCT01098539 | 495 | RCT | T2DM | 3 | Albiglutide | 63.2±8.37 | 136/113 | ND | ND | 249 | Sitagliptin | 63.5±9.02 | 130/116 | ND | ND | 246 | 52 weeks | ND | Fracture | (Leiter *et al.*, 2014) |
| Mathieu | 2014 | NCT01388361 | 177 | RCT | T2DM | 3 | Liraglutide | 61.1±9.5 | 63/25 | 32.5±5.4 | 7.7±0.6 | 88 | Insulin | 60.9±8.8 | 53/36 | 32.0±4.8 | 7.7±0.8 | 89 | 52 weeks | 12.35 | Fracture | (Mathieu *et al.*, 2014) |
| NCT00798161 | 2014 | NCT00798161 | 216 | RCT | T2DM | 3 | Metformin | 52.9±10.4 | 82/62 | 28.92±4.83 | 8.66±0.90 | 144 | Placebo | 55.7±11.0 | 36/36 | 28.62±5.17 | 8.67±0.95 | 72 | 24 weeks | ND | Fracture | (NCT00798161, 2014) |
| NCT00798161 | 2014 | NCT00798161 | 214 | RCT | T2DM | 3 | Linagliptin | 55.2±10.6 | 80/62 | 28.95±4.67 | 8.7±0.97 | 142 | Placebo | 55.7±11.0 | 36/36 | 28.62±5.17 | 8.67±0.95 | 72 | 24 weeks | ND | Fracture | (NCT00798161, 2014) |
| NCT00859898 | 2014 | NCT00859898 | 427 | RCT | T2DM | 3 | Dapagliflozin | 51.1±11.53 | 105/114 | ND | 9.05±1.279 | 219 | Metformin | 52.7±10.38 | 97/111 | ND | 9.05±1.308 | 208 | 24 weeks | 2 | Fracture | (NCT00859898, 2014) |
| NCT01042977 | 2014 | NCT01042977 | 962 | RCT | T2DM | 3 | Dapagliflozin | 63.9±7.6 | 321/159 | 33.0±5.3 | 8.0±0.8 | 480 | Placebo | 63.6±7.0 | 323/159 | 32.7±5.7 | 8.1±0.8 | 482 | 28 weeks | 13.25 | Fracture | (NCT01042977, 2014) |
| NCT01076088 | 2014 | NCT01076088 | 244 | RCT | T2DM | 3 | Sitagliptin | 51.7±10.2 | 74/46 | ND | 8.74±1.14 | 120 | Metformin | 53.0±10.3 | 75/49 | ND | 8.67±1.08 | 124 | 24 weeks | ND | Fracture | (NCT01076088, 2014) |
| NCT01076088 | 2014 | NCT01076088 | 247 | RCT | T2DM | 3 | Sitagliptin | 51.7±10.2 | 74/46 | ND | 8.74±1.14 | 120 | Placebo | 53.6±9.7 | 87/40 | ND | 8.97±1.05 | 127 | 24 weeks | ND | Fracture | (NCT01076088, 2014) |
| NCT01183013 | 2014 | NCT01183013 | 264 | RCT | T2DM | 3 | Pioglitazone | 57.2±11.1 | 70/64 | ND | 7.99±0.85 | 134 | Linagliptin | 56.3±10.1 | 79/51 | ND | 8.01±0.88 | 130 | 85 weeks | ND | Fracture | (NCT01183013, 2014) |
| NCT01558271 | 2014 | NCT01558271 | 417 | RCT | T2DM | 3 | Dulaglutide | 57.2±9.6 | 228/52 | 25.6±3.6 | 8.2±0.8 | 280 | Liraglutide | 57.9±10.4 | 113/24 | 25.5±3.5 | 8.1±0.9 | 137 | 52 weeks | 6.5 | Fracture | (NCT01558271, 2014) |
| NCT01644500 | 2014 | NCT01644500 | 481 | RCT | T2DM | 3 | Dulaglutide | 52.69±10.754 | 134/105 | 25.5±3.0 | 7.5±0.6 | 239 | Sulfonylurea | 51.97±10.052 | 130/112 | 25.2±2.8 | 7.4±0.6 | 242 | 26 weeks | 7.4 | Fracture | (NCT01644500, 2014) |
| NCT01648582 | 2014 | NCT01648582 | 503 | RCT | T2DM | 3 | Dulaglutide | 55.3±9.572 | 135/118 | 26.0±3.2 | 7.6±0.6 | 253 | Insulin | 55.44±9.197 | 139/111 | 26.1±3.3 | 7.6±0.6 | 250 | 26 weeks | 7.7 | Fracture | (NCT01648582, 2014) |
| Pratley | 2014 | NCT01023581 | 221 | RCT | T2DM | 3 | Alogliptin | 52.6±9.38 | 48/64 | 30.8±5.22 | ND | 112 | Placebo | 53.1±9.60 | 55/54 | 31.2±5.27 | ND | 109 | 26 weeks | 3.95 | Fracture | (Pratley *et al.*, 2014) |
| Ridderstrale | 2014 | NCT01167881 | 1545 | RCT | T2DM | 3 | Empagliflozin | 55.7±10.4 | 432/333 | 30.0±5.3 | 7.92±0.86 | 765 | Sulfonylurea | 56.2±10.3 | 421/359 | 30.3±5.3 | 7.92±0.86 | 780 | 104 weeks | ND | Fracture | (Ridderstråle *et al.*, 2014) |
| Rosenstock | 2014 | NCT01306214 | 374 | RCT | T2DM | 3 | Empagliflozin | 56.7±8.7 | 89/97 | 34.7±3.8 | 7.44±0.05 | 186 | Placebo | 55.3±10.1 | 113/75 | 34.7±4.3 | 7.84±0.07 | 188 | 52 weeks | ND | Fracture | (Rosenstock *et al.*, 2014d) |
| Rosenstock | 2014 | NCT00976391 | 566 | RCT | T2DM | 3 | Albiglutide | 54.8±9.1 | 132/153 | ND | 8.5±0.9 | 285 | Insulin | 56.3±8.9 | 136/145 | ND | 8.4±0.9 | 281 | 60 weeks | 11 | Fracture | (Rosenstock *et al.*, 2014a) |
| Rosenstock | 2014 | NCT01606007 | 355 | RCT | T2DM | 3 | Saxagliptin | 55±10 | 94/82 | 31.8±5.1 | 9.03±1.05 | 176 | Dapagliflozin | 54±10 | 89/90 | 31.5±5.3 | 8.87±1.16 | 179 | 24 weeks | 7.8 | Fracture | (Rosenstock *et al.*, 2014c) |
| Rosenstock | 2014 | NCT00713830 | 859 | RCT | T2DM | 3 | Lixisenatide | 57.0±9.8 | 284/290 | 30.1±6.6 | 8.3±0.9 | 574 | Placebo | 57.8±10.1 | 150/135 | 30.4±6.6 | 8.2±0.8 | 285 | 24 weeks | 9.45 | Fracture | (Rosenstock *et al.*, 2014b) |
| Schumm-Draeger | 2014 | NCT01217892 | 201 | RCT | T2DM | 3 | Dapagliflozin | 58.3±9.0 | 37/63 | 33.16±5.16 | 7.77±0.75 | 100 | Placebo | 58.5±9.4 | 47/54 | 31.74±4.69 | 7.94±0.85 | 101 | 16 weeks | 5.165 | Fracture | (Schumm-Draeger *et al.*, 2015) |
| Umpierrez | 2014 | NCT01126580 | 537 | RCT | T2DM | 3 | Dulaglutide | 56±10 | 114/155 | 34±6 | 7.6±0.9 | 269 | Metformin | 55±10 | 121/147 | 33±5 | 7.6±0.8 | 268 | 52 weeks | 3 | Fracture | (Umpierrez *et al.*, 2014) |
| Weissman | 2014 | NCT00838916 | 745 | RCT | T2DM | 3 | Albiglutide | 55.8±9.3 | 286/218 | 33.2±5.6 | 8.28±0.90 | 504 | Insulin | 54.7±9.8 | 132/109 | 33.0±5.4 | 8.36±0.95 | 241 | 52 weeks | 8.8 | Fracture | (Weissman *et al.*, 2014) |
| Wysham | 2014 | NCT01064687 | 420 | RCT | T2DM | 3 | Dulaglutide | 56±10 | 163/116 | 33±5 | 8.1±1.3 | 279 | Placebo | 55±10 | 83/58 | 33±6 | 8.1±1.3 | 141 | 26 weeks | 9 | Fracture | (Wysham *et al.*, 2014) |
| Yale | 2014 | NCT01064414 | 180 | RCT | T2DM | 3 | Canagliflozin | 69.5±8.2 | 58/32 | 32.4±5.5 | 7.9±0.9 | 90 | Placebo | 68.2±8.4 | 57/33 | 33.1±6.5 | 8.0±0.9 | 90 | 26 weeks | 16 | Fracture | (Yale *et al.*, 2014) |
| Ahmann | 2015 | NCT01617434 | 450 | RCT | T2DM | 3 | Liraglutide | 59.3±9.2 | 120/105 | 32.3±5.6 | 8.2±0.8 | 225 | Placebo | 57.5±11.1 | 136/89 | 32.2±5.7 | 8.3±0.9 | 225 | 26 weeks | 12.1 | Fracture | (Ahmann *et al.*, 2015) |
| Araki | 2015 | NCT01368081 | 199 | RCT | T2DM | 3 | Empagliflozin | 61.3±9.9 | 99/37 | 24.6±3.8 | 7.99±0.73 | 136 | Metformin | 60.0±10.2 | 47/16 | 25.2±3.6 | 7.93±0.79 | 63 | 52 weeks | ND | Fracture | (Araki *et al.*, 2015) |
| Araki | 2015 | NCT02157298 | 182 | RCT | T2DM | 4 | Dapagliflozin | 583±9.83 | 89/33 | 26.89±4.93 | 8.26±0.792 | 122 | Placebo | 57.6±9.86 | 40/20 | 26.12±3.485 | 8.52±0.937 | 60 | 52 weeks | 14.78 | Fracture | (Araki *et al.*, 2016) |
| Blonde | 2015 | NCT01191268 | 591 | RCT | T2DM | 3 | Dulaglutide | 58.9±9.6 | 160/135 | 32±5.1 | 8.46±1.08 | 295 | Insulin | 59.9±9.1 | 165/131 | 32.4±5.3 | 8.53±1.03 | 296 | 52 weeks | 12.9 | Fracture | (Blonde *et al.*, 2015) |
| Cefalu | 2015 | NCT01031680 | 914 | RCT | T2DM | 3 | Dapagliflozin | 62.8±7.0 | 309/146 | 32.6±5.9 | 8.18±0.84 | 455 | Placebo | 63±7.7 | 315/144 | 32.9±6.1 | 8.08±0.8 | 459 | 24 weeks | ND | Fracture | (Cefalu *et al.*, 2015) |
| Davies | 2015 | NCT01272232 | 635 | RCT | T2DM | 3 | Liraglutide | 55.0±10.8 | 220/203 | 37.1±6.5 | 7.9±0.8 | 423 | Placebo | 54.7±9.8 | 97/115 | 37.4±7.1 | 7.9±0.8 | 212 | 56 weeks | 7.1 | Fracture | (Davies *et al.*, 2015) |
| DeFronzo | 2015 | NCT01422876 | 265 | RCT | T2DM | 3 | Empagliflozin | 56.1±10.5 | 78/59 | 30.9±5.4 | 8.00±0.93 | 137 | Linagliptin | 56.2±10.0 | 64/64 | 30.6±5.4 | 8.02±0.90 | 128 | 24 weeks | ND | Fracture | (DeFronzo *et al.*, 2015) |
| Giorgino | 2015 | NCT01075282 | 535 | RCT | T2DM | 3 | Dulaglutide | 56±10 | 144/129 | 31±5 | 8.2±1.0 | 273 | Insulin | 57±9 | 134/128 | 32±6 | 8.1±1.0 | 262 | 52 weeks | 9 | Fracture | (Giorgino *et al.*, 2015) |
| Haering | 2015 | NCT01289990 | 450 | RCT | T2DM | 3 | Empagliflozin | 57.0±9.2 | 113/112 | 28.3±5.4 | 8.1±0.8 | 225 | Placebo | 56.9±9.2 | 112/113 | 27.9±4.9 | 8.2±0.8 | 225 | 76 weeks | ND | Fracture | (Haering *et al.*, 2015) |
| Hirose | 2015 | NCT02002221 | 156 | RCT | T2DM | 4 | Vildagliptin | 58.5±9.6 | 55/23 | 25.3±3.4 | 8.1±0.8 | 78 | Placebo | 60.1±9.1 | 56/22 | 26.0±3.1 | 8.1±0.9 | 78 | 12 weeks | 12.85 | Fracture | (Hirose *et al.*, 2015) |
| Home | 2015 | NCT00839527 | 392 | RCT | T2DM | 3 | Pioglitazone | 55.7±9.39 | 148/129 | 32.2±5.7 | 8.29±0.88 | 277 | Placebo | 55.7±9.59 | 70/45 | 31.8±4.9 | 8.26±0.98 | 115 | 156 weeks | 9.25 | Fracture | (Home *et al.*, 2015) |
| Jaiswal | 2015 | NCT00855439 | 46 | RCT | T2DM | ND | Exenatide | 51±13 | 13/9 | 35±3 | 8.2±1.1 | 22 | Glargine | 54±9 | 13/11 | 37±6 | 8.4±1.4 | 24 | 78 weeks | 7.5 | Fracture | (Jaiswal *et al.*, 2015) |
| Ji | 2015 | NCT01438814 | 689 | RCT | T2DM | 4 | Linagliptin | 53.1±10.7 | 169/175 | 29.0±5.7 | 8.0±1.0 | 344 | Placebo | 52.9±10.7 | 158/187 | 29.0±5.6 | 8.0±0.8 | 345 | 14 weeks | ND | Fracture | (Ji *et al.*, 2015) |
| Kovacs | 2015 | NCT01210001 | 330 | RCT | T2DM | 3 | Empagliflozin | 54.7±9.9 | 83/82 | 29.2±5.6 | 8.1±0.89 | 165 | Placebo | 54.6±10.5 | 73/92 | 29.3±5.4 | 8.2±0.92 | 165 | 24 weeks | ND | Fracture | (Kovacs *et al.*, 2015) |
| Mathieu | 2015 | NCT01462266 | 658 | RCT | T2DM | 3 | Sitagliptin | 59.3±8.9 | 151/178 | 31.9±5.8 | 8.7±1.0 | 329 | Placebo | 58.3±9.7 | 164/165 | 32.2±6.6 | 8.8±1.0 | 329 | 24 weeks | 13.5 | Fracture | (Mathieu *et al.*, 2015b) |
| Mathieu | 2015 | NCT01646320 | 320 | RCT | T2DM | 3 | Dapagliflozin | 55.2±8.6 | 70/90 | 31.2±4.7 | 8.24±0.96 | 160 | Placebo | 55.0±9.6 | 84/76 | 32.2±5.3 | 8.17±0.98 | 160 | 24 weeks | 7.6 | Fracture | (Mathieu *et al.*, 2015a) |
| Matthaei | 2015 | NCT01619059 | 315 | RCT | T2DM | 3 | Saxagliptin | 54.7±9.8 | 73/80 | 31.4±5.2 | 7.97±0.83 | 153 | Placebo | 54.5±9.3 | 76/86 | 31.4±5.3 | 7.86±0.93 | 162 | 24 weeks | 7.75 | Fracture | (Matthaei *et al.*, 2015) |
| NCT01733758 | 2015 | NCT01733758 | 237 | RCT | T2DM | 3 | Albiglutide | 59.6±9.0 | 125/35 | ND | ND | 160 | Placebo | 57.3±11.27 | 52/25 | ND | ND | 77 | 24 weeks | ND | Fracture | (NCT01733758, 2015) |
| Neal | 2013 | NCT01032629 | 1382 | RCT | T2DM | 3 | Canagliflozin | 62 | 659/33 | 33.0±6.5 | 8.3±0.9 | 692 | Placebo | 63 | 656/34 | 33.1±6.5 | 8.3±0.9 | 690 | 52 weeks | 16.2 | Fracture | (Neal *et al.*, 2013) |
| Pfeffer | 2015 | NCT01147250 | 6068 | RCT | T2DM | 3 | Lixisenatide | 59.9±9.7 | 2111/923 | 30.1±5.6 | 7.72±1.32 | 3034 | Placebo | 60.6±9.6 | 2096/938 | 30.2±5.8 | 7.64±1.28 | 3034 | 108 weeks | 9.3 | Fracture | (Pfeffer *et al.*, 2015) |
| Schernthaner | 2015 | NCT01006603 | 720 | RCT | T2DM | 3/4 | Saxagliptin | 72.5±5.72 | 217/143 | 29.9±5.0 | 7.58±0.67 | 360 | Sulfonylurea | 72.7±5.44 | 228/132 | 29.3±4.7 | 7.62±0.65 | 360 | 52 weeks | 7.6 | Fracture | (Schernthaner *et al.*, 2015) |
| Sheu | 2015 | NCT00954447 | 154 | RCT | T2DM | 3 | Linagliptin | 57.5±10.1 | 40/40 | 25.6±3.6 | 8.5±0.9 | 80 | Placebo | 56.7±9.6 | 33/41 | 26.2±3.4 | 8.6±0.9 | 74 | 52 weeks | ND | Fracture | (Sheu *et al.*, 2015) |
| Weinstock | 2015 | NCT00734474 | 617 | RCT | T2DM | 3 | Dulaglutide | 54±10 | 146/158 | 31.15±4.4 | 8.19±1.11 | 302 | Sitagliptin | 54±10 | 151/164 | 31.02±4.2 | 8.09±1.09 | 315 | 52 weeks | 7.09 | Fracture | (Weinstock *et al.*, 2015) |
| Weinstock | 2015 | NCT00734474 | 479 | RCT | T2DM | 3 | Dulaglutide | 54±10 | 146/158 | 31.15±4.4 | 8.19±1.11 | 302 | Placebo | 54±10 | 151/164 | 31.37±4.25 | 8.10±1.14 | 177 | 52 weeks | 7.09 | Fracture | (Weinstock *et al.*, 2015) |
| Yang | 2016 | NCT01095666 | 292 | RCT | T2DM | 3 | Dapagliflozin | 53.1±9.1 | 67/80 | 26.4±3.5 | 8.09±0.72 | 147 | Placebo | 53.5±9.2 | 86/59 | 25.7±2.9 | 8.13±0.85 | 145 | 24 weeks | 4.75 | Fracture | (Yang *et al.*, 2016) |
| Zinman | 2015 | NCT01131676 | 4678 | RCT | T2DM | 3 | Empagliflozin | 63.0±8.6 | 1653/692 | 30.6±5.2 | 8.07±0.86 | 2345 | Placebo | 63.2±8.8 | 1680/653 | 30.7±5.2 | 8.08±0.84 | 2333 | 206 weeks | ND | Fracture | (Zinman *et al.*, 2015) |
| Aroda | 2016 | NCT01664247 | 346 | RCT | T2DM | 3 | Insulin | 57.0±10.0 | 98/76 | 32.0±5.7 | 7.6±0.6 | 174 | Placebo | 57.3±9.4 | 104/68 | 32.4±5.4 | 7.6±0.6 | 172 | 26 weeks | 9.5 | Fracture | (Aroda *et al.*, 2016) |
| Bilezikian | 2016 | NCT01106651 | 573 | RCT | T2DM | 3 | Canagliflozin | 63.4±6.0 | 129/107 | 31.5±4.6 | 7.7±0.8 | 236 | Placebo | 63.2±6.2 | 143/94 | 31.8±4.7 | 7.8±0.8 | 237 | 104 weeks | 11.7 | BMD/Fracture | (Bilezikian *et al.*, 2016) |
| Dungan | 2016 | NCT01769378 | 299 | RCT | T2DM | 3 | Dulaglutide | 57.7±10.2 | 104/135 | 30.9±5.2 | 8.4±0.7 | 239 | Placebo | 58.2±7.4 | 28/32 | 32.4±5.9 | 8.4±0.7 | 60 | 24 weeks | 7.3 | Fracture | (Dungan *et al.*, 2016) |
| Frías | 2016 | NCT02229396 | 457 | RCT | T2DM | 3 | Dapagliflozin | 54.5±9.16 | 110/120 | 33.0±6.1 | 9.3±1.0 | 230 | Exenatide | 54.5±9.16 | 116/111 | 32.0±5.9 | 9.3±1.1 | 227 | 28 weeks | 7.25 | Fracture | (Frías *et al.*, 2016) |
| Hadjadj | 2016 | NCT01719003 | 328 | RCT | T2DM | 3 | Empagliflozin | 53.3±10.7 | 83/81 | 30.6±5.9 | 8.86±1.29 | 164 | Metformin | 51.6±10.8 | 92/72 | 30.5±5.9 | 8.58±1.13 | 164 | 24 weeks | ND | Fracture | (Hadjadj *et al.*, 2016) |
| Lu | 2016 | NCT01505426 | 170 | RCT | T2DM | 3 | Ipragliflozin | 53.9±11.3 | 44/43 | 26.57±4.30 | 7.74±0.78 | 87 | Placebo | 53.4±11.3 | 33/50 | 27.04±4.06 | 7.75±0.71 | 83 | 24 weeks | 6.15 | Fracture | (Lu *et al.*, 2016) |
| Marso | 2016 | NCT01720446 | 1650 | RCT | T2DM | 3 | Semaglutide | 64.6±7.3 | 495/331 | ND | ND | 826 | Placebo | 64.8±7.6 | 482/342 | ND | ND | 824 | 104 weeks | 14.1 | Fracture | (Marso *et al.*, 2016a) |
| Marso | 2016 | NCT01179048 | 9340 | RCT | T2DM | 3 | Liraglutide | 64.2±7.2 | 3011/1657 | 32.5±6.3 | 8.7±1.6 | 4668 | Placebo | 64.4±7.2 | 2992/1680 | 32.5±6.3 | 8.7±1.5 | 4672 | 182 weeks | 12.85 | Fracture | (Marso *et al.*, 2016b) |
| Moses | 2016 | NCT01076075 | 422 | RCT | T2DM | 3 | Sitagliptin | 54.4±9.6 | 95/115 | 29.5±4.8 | 8.4±0.8 | 210 | Placebo | 55.4±10.2 | 98/114 | 28.8±5.0 | 8.4±0.9 | 212 | 24 weeks | 7.7 | Fracture | (Moses *et al.*, 2016) |
| Nauck | 2016 | NCT00849017 | 202 | RCT | T2DM | 3 | Albiglutide | 53.6±10.9 | 58/43 | 33.7±5.1 | 8.0±0.8 | 101 | Placebo | 53.1±11.7 | 58/43 | 33.00±5.4 | 8.0±0.9 | 101 | 52 weeks | 3.85 | Fracture | (Nauck *et al.*, 2016) |
| Rodbard | 2016 | NCT01989754 | 213 | RCT | T2DM | 4 | Canagliflozin | 57.4±9.3 | 66/41 | 32.3±5.8 | 8.5±0.9 | 107 | Placebo | 57.5±10.1 | 55/51 | 31.7±5.5 | 8.4±0.8 | 106 | 26 weeks | 9.9 | Fracture | (Rodbard *et al.*, 2016) |
| Rosenstock | 2016 | NCT02058147 | 701 | RCT | T2DM | 3 | Lixisenatide | 58.7±8.7 | 133/101 | 32.0±4.4 | 8.3±0.7 | 234 | Insulin | 58.3±9.4 | 237/230 | 31.7±4.5 | 8.2±0.7 | 467 | 30 weeks | 8.8 | Fracture | (Rosenstock *et al.*, 2016a) |
| Rosenstock | 2016 | NCT01768559 | 596 | RCT | T2DM | 3 | Lixisenatide | 59.8±8.6 | 138/160 | 32.3±4.6 | 8.5±0.7 | 298 | Insulin | 60.2±8.6 | 135/163 | 31.9±4.4 | 8.5±0.7 | 298 | 26 weeks | 12.1 | Fracture | (Rosenstock *et al.*, 2016b) |
| Weber | 2016 | NCT01195662 | 449 | RCT | T2DM | 3 | Dapagliflozin | 56 | 118/107 | ND | 8.1±0.9 | 225 | Placebo | 57 | 129/95 | ND | 8.0±1.0 | 224 | 12 weeks | 7.5 | Fracture | (Weber *et al.*, 2016b) |
| Weber | 2016 | NCT01137474 | 613 | RCT | T2DM | 3 | Dapagliflozin | 55.6±8.4 | 179/123 | ND | 8.1±1.0 | 302 | Placebo | 56.2±8.9 | 171/140 | ND | 8.0±0.9 | 311 | 12 weeks | 7.9 | Fracture | (Weber *et al.*, 2016a) |
| Ahrén | 2017 | NCT01930188 | 816 | RCT | T2DM | 3 | Semaglutide | 54.8±10.2 | 207/202 | 32.4±6.2 | 8.0±0.9 | 409 | Sitagliptin | 54.6±10.4 | 208/199 | 32.5±5.8 | 8.2±0.9 | 407 | 56 weeks | 6.5 | Fracture | (Ahrén *et al.*, 2017) |
| Aroda | 2017 | NCT02128932 | 722 | RCT | T2DM | 3 | Semaglutide | 56.5±10.3 | 197/165 | 33.1±6.5 | 8.1±0.8 | 362 | Insulin | 56.2±10.6 | 195/165 | 33.0±6.5 | 8.1±0.9 | 360 | 30 weeks | 8.2 | Fracture | (Aroda *et al.*, 2017) |
| Bethel | 2017 | NCT00790205 | 2004 | RCT | T2DM | 3 | Sitagliptin | 78.3±3.0 | 682/288 | 29.0±4.9 | 7.19±0.46 | 970 | Placebo | 78.4±3.2 | 674/360 | 28.9±4.8 | 7.17±0.46 | 1034 | 295 weeks | 11.6 | Fracture | (Bethel *et al.*, 2017) |
| Gantz | 2017 | NCT01703208 | 4202 | RCT | T2DM | 3 | Omarigliptin | 63.7±8.5 | 1461/639 | 31.2±5.5 | 8.0±0.9 | 2100 | Placebo | 63.6±8.5 | 1487/615 | 31.4±5.6 | 8.0±0.9 | 2102 | 234 weeks | 12.05 | Fracture | (Gantz *et al.*, 2017) |
| Groop | 2017 | NCT01792518 | 360 | RCT | T2DM | 3 | Linagliptin | 61.0±10.0 | 116/66 | 28.3±4.8 | 7.82±0.87 | 182 | Placebo | 60.1±9.3 | 113/65 | 28.6±4.9 | 7.86±0.89 | 178 | 24 weeks | ND | Fracture | (Groop *et al.*, 2017) |
| Home | 2018 | NCT01717313 | 329 | RCT | T2DM | 3 | Omarigliptin | 57.4±9.2 | 95/70 | 31.1±6.1 | 8.0±0.9 | 165 | Placebo | 57.0±9.7 | 97/67 | 30.8±6.6 | 8.1±1.0 | 164 | 24 weeks | 5.55 | Fracture | (Home *et al.*, 2018) |
| Hong | 2017 | NCT02949193 | 222 | RCT | T2DM | 3 | Evogliptin | 57.6±9.4 | 51/61 | 25.6±3.7 | 7.44±0.73 | 112 | Sitagliptin | 57.3±9.3 | 52/58 | 25.3±2.7 | 7.44±0.73 | 110 | 24 weeks | 8.2 | Fracture | (Hong *et al.*, 2017) |
| Kadowaki | 2017 | NCT02354235 | 138 | RCT | T2DM | 3 | Canagliflozin | 58.4±8.9 | 54/16 | 25.53±4.21 | 8.18±0.90 | 70 | Placebo | 56.0±9.5 | 53/15 | 26.44±3.87 | 7.87±0.83 | 68 | 24 weeks | 7.42 | Fracture | (Kadowaki *et al.*, 2017) |
| Yu | 2017 | NCT02152371 | 300 | RCT | T2DM | 3 | Dulaglutide | 60.2±9.5 | 85/65 | 32.8±4.9 | 8.4±0.9 | 150 | Placebo | 60.6±10.1 | 88/62 | 32.6±4.9 | 8.3±0.8 | 150 | 28 weeks | 13.2 | Fracture | (Yu *et al.*, 2017) |
| Meneilly | 2017 | NCT01798706 | 350 | RCT | T2DM | 3 | Lixisenatide | 74.0±4.0 | 92/84 | 29.9±3.7 | 8.1±0.7 | 176 | Placebo | 74.4±3.8 | 90/84 | 30.1±4.5 | 8.1±0.7 | 174 | 24 weeks | 14.1 | Fracture | (Meneilly *et al.*, 2017) |
| Mu | 2017 | NCT01708902 | 292 | RCT | T2DM | 3 | Liagliptin | 50.8±10.5 | 76/71 | 26.2±3.9 | 8.7±0.9 | 147 | Metformin | 52.1±9.6 | 91/54 | 25.8±3.3 | 8.7±1.1 | 145 | 24 weeks | ND | Fracture | (Mu *et al.*, 2017) |
| NCT02471404 | 2017 | NCT02471404 | 626 | RCT | T2DM | 4 | Saxagliptin | 59.2±7.87 | 190/122 | ND | ND | 312 | Placebo | 57.4±9.36 | 202/112 | ND | ND | 314 | 52 weeks | ND | Fracture | (NCT02471404, 2017) |
| NCT02471404 | 2017 | NCT02471404 | 627 | RCT | T2DM | 4 | Dapagliflozin | 57.4±9.36 | 202/112 | ND | ND | 314 | Sulfonylurea | 58.6±8.38 | 208/105 | ND | ND | 313 | 52 weeks | ND | Fracture | (NCT02471404, 2017) |
| NCT02681094 | 2017 | NCT02681094 | 579 | RCT | T2DM | 3 | Saxagliptin | 57.2±10.68 | 142/148 | ND | ND | 290 | Placebo | 55.9±10.94 | 152/137 | ND | ND | 289 | 24 weeks | ND | Fracture | (NCT02681094, 2017) |
| Pratley | 2018 | NCT02099110 | 497 | RCT | T2DM | 3 | Ertuglilozin | 55.1±10.1 | 127/123 | 31.8±6.2 | 8.6±1.0 | 250 | Sitagliptin | 54.8±10.7 | 154/93 | 31.7±6.5 | 8.5±1.0 | 247 | 52 weeks | 6.65 | Fracture | (Pratley *et al.*, 2018) |
| Rodbard | 2017 | NCT01618162 | 435 | RCT | T2DM | 3 | Liraglutide | 60±9.6 | 154/135 | 31.2±4.8 | 7.9±0.6 | 289 | Placebo | 59.4±10.8 | 73/73 | 32.0±4.5 | 7.9±0.6 | 146 | 26 weeks | 9.15 | Fracture | (Rodbard *et al.*, 2017) |
| Rosenstock | 2017 | NCT02033889 | 414 | RCT | T2DM | 3 | Ertuglilozin | 56.9±9.4 | 93/112 | 31.1±4.5 | 8.1±0.9 | 205 | Placebo | 56.5±8.7 | 98/111 | 30.7±4.7 | 8.2±0.9 | 209 | 26 weeks | 8 | BMD/Fracture | (Rosenstock *et al.*, 2018) |
| Shankar | 2017 | NCT01755156 | 402 | RCT | T2DM | 3 | Omarigliptin | 57.5±8.1 | 101/100 | 32.4±5.8 | 8.1±0.9 | 201 | Placebo | 56.8±9.1 | 102/99 | 32.6±6.1 | 8.0±0.9 | 201 | 24 weeks | 7.8 | Fracture | (Shankar *et al.*, 2017b) |
| Shankar | 2017 | NCT01590797 | 467 | RCT | T2DM | 3 | Sitagliptin | 58.6±8.4 | 130/104 | 26.1±2.9 | 8.8±0.9 | 233 | Placebo | 56.7±9.1 | 116/117 | 25.9±3.0 | 8.7±0.9 | 234 | 24 weeks | 11.15 | Fracture | (Shankar *et al.*, 2017a) |
| Søfteland | 2017 | NCT01734785 | 217 | RCT | T2DM | 3 | Empagliflozin | 54.3±9.6 | 66/43 | 31.2±5.9 | 7.97±0.84 | 109 | Placebo | 55.9±9.7 | 60/48 | 29.6±5.7 | 7.97±0.85 | 108 | 24 weeks | ND | Fracture | (Søfteland *et al.*, 2017) |
| Suzuki | 2017 | NCT01584232 | 361 | RCT | T2DM | 3 | Dulaglutide | 57.5±10.5 | 125/56 | 26.1±3.6 | 8.1±0.8 | 181 | Insulin | 56.1±11.3 | 133/47 | 25.9±3.9 | 8.0±0.9 | 180 | 26 weeks | 8.85 | Fracture | (Suzuki *et al.*, 2017) |
| Terauchi | 2017 | NCT01183104 | 272 | RCT | T2DM | ND | Sitagliptin | 70.2±5.4 | 74/69 | 24.1±3.3 | 7.5±0.7 | 143 | Sulfonylurea | 70.8±5.5 | 79/50 | 24.2±3.6 | 7.5±0.7 | 129 | 52 weeks | 43.5 (median) | Fracture | (Terauchi *et al.*, 2017) |
| Vaccaro | 2017 | NCT00700856 | 3028 | RCT | T2DM | 4 | Pioglitazone | 62.4±6.4 | 909/626 | 30.2±4.4 | 8.4±5.6 | 1535 | Sulfonylurea | 62.2±6.5 | 865/628 | 30.4±4.5 | 8.5±5.8 | 1493 | 26 weeks | 8.45 | Fracture | (Vaccaro *et al.*, 2017) |
| Vianna | 2017 | NCT01679899 | 42 | RCT | T2DM | 4 | Vildagliptin | 62.8±6.4 | 0/21 | 31.7±5.8 | 7.35±0.54 | 21 | Sulfonylurea | 61±5.3 | 0/21 | 33.8±6.7 | 7.32±0.60 | 21 | 52 weeks | ND | BMD | (Vianna *et al.*, 2017) |
| Ahmann | 2018 | NCT01885208 | 809 | RCT | T2DM | 3 | Semaglutide | 56.4±10.2 | 219/185 | 34 | 8.36±0.95 | 404 | Exenatide | 56.7±11.1 | 228/177 | 33.6 | 8.33±0.96 | 405 | 56 weeks | 9.2 | Fracture | (Ahmann *et al.*, 2018) |
| Aronson | 2018 | NCT01958671 | 305 | RCT | T2DM | 3 | Ertugliflozin | 56.2±10.8 | 90/62 | 32.5±5.7 | 8.4±1.1 | 152 | Metformin | 56.1±10.9 | 82/71 | 33.3±6.8 | 8.1±0.9 | 153 | 52 weeks | 4.9 | Fracture | (Aronson *et al.*, 2018) |
| Dagogo-Jack | 2018 | NCT02036515 | 309 | RCT | T2DM | 3 | Ertugliflozin | 59.2±9.3 | 81/75 | 31.2±5.5 | 8.1±0.9 | 156 | Placebo | 58.3±9.2 | 100/53 | 30.3±6.14 | 8.0±0.9 | 153 | 26 weeks | 9.65 | Fracture | (Dagogo-Jack *et al.*, 2018) |
| Fioretto | 2018 | NCT02413398 | 321 | RCT | T2DM | 3 | Dapagliflozin | 65.3±13.2 | 91/69 | 32.6±4.7 | 8.33±1.08 | 160 | Placebo | 66.2±10.0 | 91/70 | 31.6±5.0 | 8.03±1.08 | 161 | 24 weeks | 14.4 | Fracture | (Fioretto *et al.*, 2018) |
| Grunberger | 2018 | NCT01986855 | 312 | RCT | T2DM | 3 | Ertugliflozin | 66.7±8.3 | 84/74 | 32.6±6.8 | 8.2±1.0 | 158 | Placebo | 67.5±8.9 | 72/82 | 33.2±6.1 | 8.1±0.9 | 154 | 54 weeks | 14 | Fracture | (Grunberger *et al.*, 2018) |
| Guja | 2018 | NCT02229383 | 461 | RCT | T2DM | 3 | Exenatide | 57.8±9.0 | 114/117 | 33.3±6.1 | 8.53±0.91 | 231 | Placebo | 57.6±10.3 | 107/123 | 34.1±6.6 | 8.53±0.92 | 230 | 28 weeks | 11.3 | Fracture | (Guja *et al.*, 2018) |
| Hernandez | 2018 | NCT02465515 | 9463 | RCT | T2DM | 4 | Albiglutide | 64.1±8.7 | 3304/1427 | 32.3±5.9 | 8.76±1.5 | 4731 | Placebo | 64.2±8.7 | 3265/1467 | 32.3±5.9 | 8.72±1.5 | 4732 | 140 weeks | 14.15 | Fracture | (Hernandez *et al.*, 2018) |
| Hollander | 2018 | NCT01999218 | 880 | RCT | T2DM | 3 | Ertuglilozin | 58.7±9.8 | 227/218 | 31.7±5.6 | 7.8±0.6 | 445 | Sulfonylurea | 57.9±9.1 | 224/211 | 31.2±6.4 | 7.8±0.6 | 435 | 104 weeks | 7.4 | Fracture | (Hollander *et al.*, 2018) |
| Kaku | 2019 | NCT02489968 | 215 | RCT | T2DM | 3 | Empagliflozin | 58.0±9.3 | 85/22 | 24.9±4.0 | 8.34±0.54 | 107 | Placebo | 56.3±9.9 | 85/23 | 25.1±3.6 | 8.40±0.68 | 108 | 24 weeks | 8 | Fracture | (Kaku *et al.*, 2019b) |
| Kawamori | 2018 | NCT02453555 | 175 | RCT | T2DM | 3 | Empagliflozin | 60.0±9.9 | 142/40 | 26.0±3.8 | 8.27±0.65 | 82 | Placebo | 59.8±10.8 | 72/21 | 26.6±4.5 | 8.36±0.74 | 93 | 52 weeks | 8.85 | Fracture | (Kawamori *et al.*, 2018) |
| Ludvik | 2018 | NCT02597049 | 282 | RCT | T2DM | 3 | Dulaglutide | 56.17±9.26 | 77/65 | 32.87±5.56 | 8.04±0.65 | 142 | Placebo | 57.10±9.59 | 66/74 | 32.39±4.98 | 8.05±0.66 | 140 | 24 weeks | 9.04 | Fracture | (Ludvik *et al.*, 2018) |
| NCT02564926 | 2018 | NCT02564926 | 121 | RCT | T2DM | 4 | Dapagliflozin | 54.6±9.09 | 37/23 | ND | ND | 60 | Sulfonylurea | 55.7±10.24 | 27/34 | ND | ND | 61 | 52 weeks | ND | Fracture | (NCT02564926, 2018) |
| NCT02589639 | 2018 | NCT02589639 | 180 | RCT | T2DM | 4 | Empagliflozin | 59.1±10.7 | 69/21 | ND | 8.7±0.68 | 90 | Placebo | 58.6±9.5 | 61/29 | ND | 8.74±0.72 | 90 | 53 weeks | ND | Fracture | (NCT02589639, 2018) |
| NCT02906709 | 2018 | NCT02906709 | 184 | RCT | T2DM | 4 | Omarigliptin | 61.1±11.0 | 86/37 | ND | 8.81±0.68 | 123 | Placebo | 60.9±11.7 | 47/14 | ND | 8.83±0.78 | 61 | 16 weeks | ND | Fracture | (NCT02906709, 2018) |
| Nordklint | 2018 | NCT00657943 | 407 | RCT | T2DM | 4 | Metformin | 60.4±8.7 | 137/65 | 32.2±4.2 | 8.6±1.1 | 202 | Placebo | 59.8±9.2 | 140/65 | 32.0±4.2 | 8.5±1.0 | 205 | 78 weeks | 12.9 | BMD | (Nordklint *et al.*, 2018) |
| Terauchi | 2018 | NCT02201004 | 210 | RCT | T2DM | 4 | Tofogliflozin | 59.1±10.9 | 89/51 | 25.79±3.46 | 8.53±0.76 | 140 | Placebo | 56.4±10.0 | 48/22 | 26.89±3.88 | 8.40±0.65 | 70 | 52 weeks | 13.72 | Fracture | (Terauchi *et al.*, 2018) |
| Tuttle | 2018 | NCT01621178 | 386 | RCT | T2DM | 3 | Dulaglutide | 64.7±8.8 | 104/88 | 32.1±4.8 | 8.6±0.9 | 192 | Insulin | 64.3±8.4 | 93/101 | 32.4±5.3 | 8.6±1.0 | 194 | 52 weeks | 18.15 | Fracture | (Tuttle *et al.*, 2018) |
| Araki | 2019 | NCT02240680 | 102 | RCT | T2DM | 4 | Linagliptin | 71.1±5.5 | 34/18 | 23.9±3.3 | 8.1±0.8 | 52 | Placebo | 71.1±5.6 | 36/14 | 22.9±3.0 | 8.0±0.7 | 50 | 24 weeks | ND | Fracture | (Araki *et al.*, 2019) |
| Gerstein | 2019 | NCT01394952 | 9902 | RCT | T2DM | 3 | Dulaglutide | 66.2±6.5 | 2643/2306 | 32.3±5.7 | 7.3±1.1 | 4949 | Placebo | 66.2±6.5 | 2669/2283 | 32.3±5.8 | 7.4±1.1 | 4953 | 365 weeks | 10.55 | Fracture | (Gerstein *et al.*, 2019) |
| Halvorsen | 2019 | NCT03115112 | 384 | RCT | T2DM | 3 | Bexagliflozin | 59.3±9.7 | 120/71 | 32.1±6.1 | 7.94±0.81 | 191 | Sitagliptin | 59.6±9.8 | 126/67 | 31.4±5.3 | 8.03±0.92 | 193 | 24 weeks | 8.79 | Fracture | (Halvorsen *et al.*, 2019) |
| Husain | 2019 | NCT02692716 | 3183 | RCT | T2DM | 3 | Semaglutide | 66±7 | 1084/507 | 32.3±6.6 | 8.2±1.6 | 1591 | Placebo | 66±7 | 1092/500 | 32.3±6.4 | 8.2±1.6 | 1592 | 87 weeks | 14.9 | Fracture | (Husain *et al.*, 2019) |
| Ji | 2019 | NCT02630706 | 271 | RCT | T2DM | 3 | Ertuglilozin | 56.0±9.0 | 71/65 | 26.2±3.3 | 8.1±1.0 | 136 | Placebo | 56.8±8.6 | 69/66 | 26.0±2.8 | 8.2±0.9 | 135 | 26 weeks | 6.53 | Fracture | (Ji *et al.*, 2019) |
| Kaku | 2019 | NCT02607306 | 544 | RCT | T2DM | 3 | Liraglutide | 56.8±10.1 | 192/81 | 26.5±4.5 | 8.3±1.0 | 273 | Insulin | 57.8±9.9 | 195/76 | 26.6±4.8 | 8.5±1.1 | 271 | 52 weeks | 9.55 | Fracture | (Kaku *et al.*, 2019a) |
| Lingvay | 2019 | NCT03136484 | 788 | RCT | T2DM | 3 | Semaglutide | 55.7±11.1 | 223/171 | 32.2±6.8 | 8.3±1.0 | 394 | Canagliflozin | 57.5±10.7 | 201/193 | 32.5±6.9 | 8.2±1.0 | 394 | 52 weeks | 7.3 | Fracture | (Lingvay *et al.*, 2019) |
| Matthews | 2019 | NCT01528254 | 2001 | RCT | T2DM | 4 | Vildagliptin | 54.1±9.5 | 453/545 | 31.2±4.8 | 6.7±0.4 | 998 | Placebo | 54.6±9.2 | 515/488 | 31.0±4.7 | 6.7±0.5 | 1003 | 260 weeks | 3.35 | Fracture | (Matthews *et al.*, 2019) |
| Perkovic | 2019 | NCT02065791 | 4401 | RCT | T2DM | 3 | Canagliflozin | 62.9±9.2 | 1467/732 | ND | ND | 2199 | Placebo | 63.2±9.2 | 1440/762 | ND | ND | 2202 | 239 weeks | 15.8 | Fracture | (Perkovic *et al.*, 2019) |
| Pieber | 2019 | NCT02849080 | 504 | RCT | T2DM | 3 | Semaglutide | 57±10 | 145/108 | 31.5±6.5 | 8.3±0.6 | 253 | Sitagliptin | 58±10 | 140/111 | 31.5±6.1 | 8.3±0.6 | 251 | 52 weeks | 8.8 | Fracture | (Pieber *et al.*, 2019) |
| Pratley | 2019 | NCT02863419 | 427 | RCT | T2DM | 3 | Semaglutide | 56±10 | 147/138 | 32.5±5.9 | 8.0±0.7 | 285 | Placebo | 57±10 | 74/68 | 32.9±6.1 | 7.9±0.7 | 142 | 52 weeks | 7.6 | Fracture | (Pratley *et al.*, 2019) |
| Pratley | 2019 | NCT02863419 | 426 | RCT | T2DM | 3 | Liraglutide | 56±10 | 149/135 | 33.4±6.7 | 8.0±0.7 | 284 | Placebo | 57±10 | 74/68 | 32.9±6.1 | 7.9±0.7 | 142 | 52 weeks | 7.6 | Fracture | (Pratley *et al.*, 2019) |
| Rodbard | 2019 | NCT02863328 | 821 | RCT | T2DM | 3 | Semaglutide | 57±10 | 206/205 | 32.9±6.3 | 8.1±0.9 | 411 | Empagliflozin | 58±10 | 209/201 | 32.8±5.9 | 8.1±0.9 | 410 | 57 weeks | 7.45 | Fracture | (Rodbard *et al.*, 2019) |
| Rosenstock | 2019 | NCT01897532 | 6979 | RCT | T2DM | 3 | Linagliptin | 66.1±9.1 | 2148/1346 | 31.4±5.3 | 7.9±1.0 | 3494 | Placebo | 65.6±9.1 | 2242/1243 | 31.3±5.4 | 8.0±1.0 | 3485 | 224 weeks | 14.75 | Fracture | (Rosenstock *et al.*, 2019b) |
| Rosenstock | 2019 | NCT01243424 | 6033 | RCT | T2DM | 3 | Linagliptin | 63.9±9.5 | 1838/1185 | 30.2±5.2 | 7.2±0.6 | 3023 | Sulfonylurea | 64.2±9.5 | 1781/1229 | 30.0±5.1 | 7.2±0.6 | 3010 | 433 weeks | 6.25 | Fracture | (Rosenstock *et al.*, 2019a) |
| Wiviott | 2019 | NCT01730534 | 17160 | RCT | T2DM | 3 | Dapagliflozin | 63.9±6.8 | 5411/3171 | 32.1±6.0 | 8.1±0.8 | 8582 | Placebo | 64.0±6.8 | 5327/3251 | 32.0±6.1 | 8.1±0.8 | 8578 | 271 weeks | 10.5 | Fracture | (Wiviott *et al.*, 2019) |
| Cannon | 2020 | NCT01986881 | 8246 | RCT | T2DM | 3 | Ertugliflozin | 64.4±8.1 | 3866/1633 | 31.9±5.4 | 8.2±1.0 | 5499 | Placebo | 64.4±8.0 | 1903/844 | 32.0±5.5 | 8.2±0.9 | 2747 | 312 weeks | 13 | Fracture | (Cannon *et al.*, 2020) |
| Hygum | 2020 | NCT02473809 | 60 | RCT | T2DM | 4 | Liraglutide | 62 | 16/14 | 33 | 6.7 | 30 | Placebo | 64 | 14/16 | 31.3 | 6.7 | 30 | 26 weeks | 6.5 | BMD | (Hygum *et al.*, 2020) |
| NCT02912455 | 2020 | NCT02912455 | 16 | RCT | T2DM | 4 | Canagliflozin | 58 (median) | 4/7 | 39.6 (median) | 7.2 (median) | 11 | Placebo | 44 (median) | 1/4 | 37.9 (median) | 8.2 (median) | 5 | 26 weeks | ND | Fracture | (NCT02912455, 2020) |
| Kim | 2020 | NCT02426294 | 135 | RCT | T2DM | 4 | Pioglitazone | 60.7±9.1 | 34/35 | 24.4±3.2 | 8.25±0.58 | 69 | Sulfonylurea | 58.5±10.4 | 30/33 | 24.9±2.8 | 8.16±0.61 | 66 | 26 weeks | 5.075 | Fracture | (Kim *et al.*, 2020) |
| NCT03386344 | 2020 | NCT03386344 | 251 | RCT | T2DM | 3 | Sotagliflozin | 66.1±6.7 | 69/56 | ND | 8.32±0.96 | 125 | Placebo | 66.3±5.7 | 70/56 | ND | 8.38±0.91 | 126 | 26 weeks | ND | BMD/Fracture | (NCT03386344, 2020) |
| Yabe | 2020 | NCT03015220 | 197 | RCT | T2DM | 3 | Semaglutide | 59±10 | 90/42 | 26.8±5.0 | 8.3±0.9 | 132 | Dulaglutide | 61±9 | 51/14 | 26.0±4.0 | 8.4±0.9 | 65 | 57 weeks | 9.6 | Fracture | (Yabe *et al.*, 2020) |
| Bhatt | 2021 | NCT03315143 | 10584 | RCT | T2DM | 3 | Sotagliflozin | 68.4±8.4 | 2945/2347 | 31.9 (median) | 8.3 (median) | 5292 | Placebo | 68.2±8.4 | 2885/2407 | 31.7 (median) | 8.3 (median) | 5292 | 130 weeks | ND | Fracture | (Bhatt *et al.*, 2021) |
| Kim | 2022 | NCT04470310 | 66 | RCT | T2DM | 4 | Sulfonylurea | 56.46±9.17 | 19/16 | 26.03±3.17 | 8.15±0.58 | 35 | Alogliptin | 52.68±11.93 | 14/17 | 25.85±3.94 | 8.33±0.73 | 31 | 24 weeks | 4.55 | Fracture | (Kim *et al.*, 2022) |
| NCT04537923 | 2022 | NCT04537923 | 954 | RCT | T2DM | 3 | Tirzepatide | 58.0±10.18 | 99/144 | ND | 8.89±0.97 | 243 | Insulin | 59.0±9.74 | 313/398 | ND | 8.81±0.96 | 711 | 56 weeks | ND | Fracture | (NCT04537923, 2022) |
| NCT04591626 | 2022 | NCT04591626 | 291 | RCT | T2DM | 3 | Dulaglutide | 57.8±9.33 | 92/52 | ND | 8.60±1.00 | 144 | Placebo | 58.30±9.48 | 90/57 | ND | 8.58±0.97 | 147 | 28 weeks | ND | Fracture | (NCT04591626, 2022) |
| Wada | 2022 | NCT03436693 | 308 | RCT | T2DM | 3 | Canagliflozin | 62.5±10.5 | 115/39 | 26.7±4.4 | 7.75±1.10 | 154 | Placebo | 62.4±11.1 | 129/25 | 27.1±4.5 | 7.77±1.05 | 154 | 104 weeks | 15.96 | Fracture | (Wada *et al.*, 2022) |
| Cherney | 2023 | NCT03242252 | 524 | RCT | T2DM | 3 | Sotagliflozin | 69.5±8.2 | 152/112 | 32.4±5.2 | 8.3±0.9 | 264 | Placebo | 69.3±8.1 | 149/111 | 32.5±5.2 | 8.3±1.0 | 260 | 60 weeks | 17 | Fracture | (Cherney *et al.*, 2023) |
| Halvorsen | 2023 | NCT03259789 | 317 | RCT | T2DM | 3 | Bexagliflozin | 56±10.1 | 100/58 | 29.7±6.5 | 8.66±0.87 | 158 | Placebo | 55.6±11.2 | 94/65 | 30.0±6.3 | 8.57±0.79 | 159 | 24 weeks | 9.09 | Fracture | (Halvorsen *et al.*, 2023) |
| Kim | 2023 | NCT04654390 | 270 | RCT | T2DM | 3 | Enavogliflozin | 58.1±10.0 | 73/61 | 26.1±3.7 | 7.98±0.85 | 134 | Dapagliflozin | 59.1±9.8 | 68/68 | 25.6±3.0 | 8.0±0.82 | 136 | 24 weeks | 9.85 | Fracture | (Kim *et al.*, 2023) |
| Kwak | 2023 | NCT04632862 | 167 | RCT | T2DM | 3 | Enavogliflozin | 59.9±11.5 | 47/36 | 25.6±3.1 | 7.7±0.6 | 83 | Placebo | 59.7±11.4 | 37/47 | 26.2±3.5 | 7.8±0.6 | 84 | 24 weeks | 6.35 | Fracture | (Kwak *et al.*, 2023) |
| Yabe | 2023 | NCT04531462 | 127 | RCT | T2DM | 4 | Empagliflozin | 74.2±4.9 | 48/16 | 25.7±3.3 | 7.6±0.5 | 64 | Placebo | 74.0±5.1 | 44/19 | 25.4±2.6 | 7.6±0.5 | 63 | 54 weeks | 12.1 | Fracture | (Yabe *et al.*, 2023) |

ND. No data; RCT. Randomized controlled trial; T2DM. Type 2 diabetes mellites.

Table S4. Deviance information criterion for model selection

|  | **Outcome** | **Model** | **Dbar*** | **pD**** | **Deviance Information Criterion (DIC)** |
| --- | --- | --- | --- | --- | --- |
| Bone mineral density_ specific treatment | |  |  |  |  |
| Total hip | | Fixed | 29.74 | 27.39 | 57.13 |
| Lumber spine | | Fixed | 32.22 | 21.09 | 53.32 |
| Femoral neck | | Fixed | 613.04 | 44.96 | 657.99 |
|  | Bone mineral density_ treatment type |  |  |  |  |
|  | Total hip | Fixed | 23.96 | 16.99 | 40.94 |
|  | Lumber spine | Fixed | 29.69 | 18.04 | 47.72 |
|  | Femoral neck | Fixed | 613.94 | 41.21 | 655.15 |
|  | Fracture_Specific treatment |  |  |  |  |
|  | Overall Fracture | Fixed | 387.25 | 266.15 | 653.41 |
|  | Vertebral Fracture | Fixed | 81.51 | 74.59 | 156.10 |
|  | Non vertebral Fracture | Fixed | 560.90 | 189.46 | 750.36 |
|  | Hip Fracture | Fixed | 137.28 | 91.80 | 229.08 |
|  | Vertebral + Hip Fracture | Fixed | 209.04 | 113.32 | 332.36 |
|  | Multiple Fracture | Fixed | 26.45 | 26.16 | 52.61 |
|  | Fracture_Treatment type |  |  |  |  |
|  | Overall Fracture | Fixed | 386.34 | 229.39 | 615.73 |
|  | Vertebral Fracture | Fixed | 65.64 | 54.05 | 119.69 |
|  | Non vertebral Fracture | Fixed | 744.27 | 171.08 | 915.35 |
|  | Hip Fracture | Fixed | 172.63 | 77.14 | 249.78 |
|  | Vertebral + Hip Fracture | Fixed | 271.73 | 94.30 | 366.04 |
|  | Multiple Fracture | Fixed | 22.03 | 19.96 | 41.98 |
|  | Anatomy Fracture_treatment | | | | |
|  | Vertebral Column Fracture | Fixed | 81.51 | 74.59 | 156.10 |
|  | Lower Extremity Fracture | Fixed | 191.78 | 148.17 | 339.96 |
|  | Upper Extremity Fracture | Fixed | 179.53 | 108.22 | 287.75 |
|  | Pelvic Girdle Fracture | Fixed | 74.74 | 57.81 | 132.55 |
|  | Cranial and Facial bone Fracture | Fixed | 61.30 | 49.56 | 110.86 |
|  | Thoracic Cage Fracture | Fixed | 89.27 | 77.54 | 166.81 |
|  | Anatomy Fracture_treatment type | | | | |
|  | Vertebral Column Fracture | Fixed | 65.64 | 54.05 | 119.69 |
|  | Lower Extremity Fracture | Fixed | 182.26 | 130.70 | 312.97 |
|  | Upper Extremity Fracture | Fixed | 183.72 | 91.84 | 275.56 |
|  | Pelvic Girdle Fracture | Fixed | 116.12 | 43.67 | 159.79 |
|  | Cranial and Facial bone Fracture | Fixed | 67.11 | 36.06 | 103.17 |
|  | Thoracic Cage Fracture | Fixed | 81.77 | 61.42 | 143.18 |

*sum of residual deviations

** estimate of the number of parameter

Table S5. Network meta-analysis heterogeneity/inconsistency test (Fracture)_Treatment type

|  | **Treatment1** | **Treatment2** | | **I2.pair** | | **I2.cons** | **Incons.p** |
| --- | --- | --- | --- | --- | --- | --- | --- |
|  | **Overall Fracture** |  | |  | |  |  |
|  | **Per-comparison I-squared:** |  | |  | |  |  |
|  | DPP4i | GLP1RA | | 0 | | 0 | 0.40795109 |
|  | DPP4i | Metformin | | 0 | | 0 | 0.09695259 |
|  | DPP4i | Placebo | | 0 | | 0 | 0.4618645 |
|  | DPP4i | SGLT2i | | 0 | | 0 | 0.23096485 |
|  | DPP4i | Sulfonylurea | | 0 | | 0 | 0.45989643 |
|  | DPP4i | Thiazolidinedione | | 0 | | 0 | 0.71053155 |
|  | GLP1RA | Insulin | | 0 | | 0 | 0.66637596 |
|  | GLP1RA | Metformin | | NA | | 0 | 0.82622507 |
|  | GLP1RA | Placebo | | 0 | | 0 | 0.40300259 |
|  | GLP1RA | SGLT2i | | 0 | | 0 | 0.47995885 |
|  | GLP1RA | Sulfonylurea | | 0 | | 0 | 0.86741328 |
|  | Insulin | Placebo | | NA | | 0 | 0.70468838 |
|  | Metformin | Placebo | | 0 | | 0 | 0.88021254 |
|  | Metformin | SGLT2i | | 0 | | 0 | 0.28437361 |
|  | Metformin | Thiazolidinedione | | 0 | | 59.547057 | 0.01772052 |
|  | Placebo | SGLT2i | | 0 | | 0 | NA |
|  | Placebo | Sulfonylurea | | 0 | | 0 | 0.74628031 |
|  | Placebo | Thiazolidinedione | | 0 | | 3.859228 | 0.04317441 |
|  | SGLT2i | Sulfonylurea | | 16.95503 | | 0 | 0.82109408 |
|  | Sulfonylurea | Thiazolidinedione | | 0 | | 0 | 0.88533823 |
| **Global I-squared:** | |  | |  | |  |  |
|  | |  | | 0 | | 0 |  |
|  | **Vertebral Fracture** |  | |  | |  |  |
|  | **Per-comparison I-squared:** |  | |  | |  |  |
|  | DPP4i | GLP1RA | | NA | | 0 | 0.488013 |
|  | DPP4i | Metformin | | 0 | | 0 | NA |
|  | DPP4i | Placebo | | 0 | | 0 | 0.7386033 |
|  | DPP4i | SGLT2i | | NA | | 0 | 0.9882366 |
|  | DPP4i | Sulfonylurea | | 0 | | 0 | 0.6927291 |
|  | GLP1RA | Insulin | | 0 | | 0 | NA |
|  | GLP1RA | Placebo | | 0 | | 0 | NA |
|  | Metformin | Placebo | | NA | | 0 | 0.6810781 |
|  | Placebo | SGLT2i | | 0 | | 0 | 0.8472865 |
|  | Placebo | Thiazolidinedione | | NA | | 0 | 0.8442809 |
|  | Sulfonylurea | Thiazolidinedione | | 0 | | 0 | 0.7955078 |
|  | **Global I-squared:** |  | |  | |  |  |
|  |  |  | | 0 | | 0 |  |
|  | **Non-Vertebral Fracture** |  | |  | |  |  |
|  | **Per-comparison I-squared:** |  | |  | |  |  |
|  | DPP4i | GLP1RA | | 0 | | 0 | 0.680658342 |
|  | DPP4i | Metformin | | 39.568193 | | 69.86879 | 0.000102045 |
|  | DPP4i | Placebo | | 0 | | 0 | 0.206929575 |
|  | DPP4i | SGLT2i | | 0 | | 0 | 0.424586321 |
|  | DPP4i | Sulfonylurea | | 61.46428 | | 64.36288 | 0.271638092 |
|  | DPP4i | Thiazolidinedione | | 70.218686 | | 66.03585 | 0.204819186 |
|  | GLP1RA | Insulin | | 42.618322 | | 43.65156 | NA |
|  | GLP1RA | Metformin | | NA | | 54.62185 | 0.257077377 |
|  | GLP1RA | Placebo | | 9.093542 | | 30.17129 | 0.038758698 |
|  | GLP1RA | SGLT2i | | 0 | | 0 | 0.921561379 |
|  | GLP1RA | Sulfonylurea | | 0 | | 0 | 0.526527502 |
|  | Insulin | Placebo | | NA | | 25.84878 | 0.377858115 |
|  | Metformin | Placebo | | 0 | | 42.73063 | 0.140639954 |
|  | Metformin | SGLT2i | | 95.407073 | | 94.44534 | 0.313625185 |
|  | Metformin | Thiazolidinedione | | 21.290627 | | 96.95517 | 3.55173E-05 |
|  | Placebo | SGLT2i | | 72.426813 | | 71.66436 | 0.511998702 |
|  | Placebo | Sulfonylurea | | 70.23066 | | 38.53584 | 0.919028094 |
|  | Placebo | Thiazolidinedione | | 75.562892 | | 70.7221 | 0.440741099 |
|  | SGLT2i | Sulfonylurea | | 90.612915 | | 88.91217 | 0.416965021 |
|  | Sulfonylurea | Thiazolidinedione | | 91.756779 | | 68.38095 | 0.086388507 |
|  | **Global I-squared:** |  | |  | |  |  |
|  |  |  | | 22.15 | | 25.68 |  |
| **Vertebral + Hip Fracture** | |  | |  | |  |  |
| **Per-comparison I-squared:** | |  | |  | |  |  |
| DPP4i | | GLP1RA | | 15.22221 | | 56.331547 | 0.096734633 |
| DPP4i | | Metformin | | 69.90081 | | 63.749669 | 0.548934666 |
| DPP4i | | Placebo | | 0 | | 2.931197 | 0.108342472 |
| DPP4i | | SGLT2i | | 0 | | 0 | 0.185798043 |
| DPP4i | | Sulfonylurea | | 51.04523 | | 47.730226 | 0.824626502 |
| DPP4i | | Thiazolidinedione | | 46.54332 | | 66.117956 | 0.083709191 |
| GLP1RA | | Insulin | | 30.60757 | | 33.422679 | NA |
| GLP1RA | | Metformin | | NA | | 0 | 0.564563845 |
| GLP1RA | | Placebo | | 0 | | 21.833278 | 0.003386082 |
| GLP1RA | | SGLT2i | | NA | | 0 | 0.787961231 |
| GLP1RA | | Sulfonylurea | | 67.42559 | | 72.281699 | 0.071468981 |
| Insulin | | Placebo | | NA | | 67.698044 | 0.151867855 |
| Metformin | | SGLT2i | | 50.68746 | | 69.023044 | 0.029852697 |
| Metformin | | Thiazolidinedione | | 89.12041 | | 96.871828 | 0.000178979 |
| Placebo | | SGLT2i | | 45.47738 | | 45.040779 | 0.300135386 |
| Placebo | | Sulfonylurea | | NA | | 76.263662 | 0.100179982 |
| Placebo | | Thiazolidinedione | | 0 | | 0 | 0.76001468 |
| SGLT2i | | Sulfonylurea | | 92.32471 | | 90.428936 | 0.740897173 |
| Sulfonylurea | | Thiazolidinedione | | 84.77449 | | 96.645832 | 0.01895512 |
| **Global I-squared:** | |  | |  | |  |  |
|  | |  | | 0 | | 0 |  |
| **Multiple Fracture** | |  | |  | |  |  |
| **Per-comparison I-squared:** | |  | |  | |  |  |
| DPP4i | | Placebo | | 0 | | 0 | NA |
| DPP4i | | Sulfonylurea | | 0 | | 0 | NA |
| GLP1RA | | Placebo | | 0 | | 0 | NA |
| Placebo | | SGLT2i | | 0 | | 0 | NA |
| Sulfonylurea | | Thiazolidinedione | | NA | | NA | NA |
| **Global I-squared:** | |  | |  | |  |  |
|  | |  | | 0 | | 0 |  |
| **Vertebral Column Fracture** | | |  | |  |  |  |
| **Per-comparison I-squared:** | | |  | |  |  |  |
| DPP4i | | | GLP1RA | | NA | 0 | 0.488013 |
| DPP4i | | | Metformin | | 0 | 0 | NA |
| DPP4i | | | Placebo | | 0 | 0 | 0.7386033 |
| DPP4i | | | SGLT2i | | NA | 0 | 0.9882366 |
| DPP4i | | | Sulfonylurea | | 0 | 0 | 0.6927291 |
| GLP1RA | | | Insulin | | 0 | 0 | NA |
| GLP1RA | | | Placebo | | 0 | 0 | NA |
| Metformin | | | Placebo | | NA | 0 | 0.6810781 |
| Placebo | | | SGLT2i | | 0 | 0 | 0.8472865 |
| Placebo | | | Thiazolidinedione | | NA | 0 | 0.8442809 |
| Sulfonylurea | | | Thiazolidinedione | | 0 | 0 | 0.7955078 |
| **Global I-squared:** | | |  | |  |  |  |
|  | | |  | | 0 | 0 |  |
| **Lower Extremity Fracture** | | |  | |  |  |  |
| **Per-comparison I-squared:** | | |  | |  |  |  |
| DPP4i | | | GLP1RA | | 0 | 0 | 0.623437027 |
| DPP4i | | | Metformin | | 0 | 0 | 0.612209874 |
| DPP4i | | | Placebo | | 0 | 0 | 0.009560769 |
| DPP4i | | | SGLT2i | | 0 | 0 | 0.634489217 |
| DPP4i | | | Sulfonylurea | | 0 | 0 | 0.869859005 |
| DPP4i | | | Thiazolidinedione | | NA | 0 | 0.532513412 |
| GLP1RA | | | Insulin | | 0 | 0 | NA |
| GLP1RA | | | Placebo | | 0 | 0 | 0.482068895 |
| GLP1RA | | | SGLT2i | | 0 | 0 | 0.887543928 |
| GLP1RA | | | Sulfonylurea | | 0 | 0 | 0.749102281 |
| Metformin | | | Placebo | | 0 | 0 | 0.365670655 |
| Metformin | | | SGLT2i | | NA | 0 | 0.456692199 |
| Placebo | | | SGLT2i | | 0 | 0 | 0.864375025 |
| Placebo | | | Sulfonylurea | | 0 | 0 | 0.354506583 |
| Placebo | | | Thiazolidinedione | | 0 | 0 | 0.452667027 |
| SGLT2i | | | Sulfonylurea | | 0 | 0 | 0.514849089 |
| Sulfonylurea | | | Thiazolidinedione | | 0 | 0 | 0.754544072 |
| **Global I-squared:** | | |  | |  |  |  |
|  | | |  | | 0 | 0 |  |
| **Upper Extremity Fracture** | | |  | |  |  |  |
| **Per-comparison I-squared:** | | |  | |  |  |  |
| DPP4i | | | Metformin | | 0 | 0 | 0.751295497 |
| DPP4i | | | Placebo | | 17.60377 | 11.787421 | 0.968406045 |
| DPP4i | | | SGLT2i | | 0 | 0 | 0.628404099 |
| DPP4i | | | Sulfonylurea | | 0 | 0 | 0.594828007 |
| DPP4i | | | Thiazolidinedione | | 0 | 55.087898 | 0.042259703 |
| GLP1RA | | | Insulin | | 0 | 19.478217 | 0.078643063 |
| GLP1RA | | | Metformin | | NA | 0 | 0.781766714 |
| GLP1RA | | | Placebo | | 0 | 9.931973 | 0.014770202 |
| GLP1RA | | | Sulfonylurea | | 0 | 0 | 0.545631121 |
| Insulin | | | Placebo | | NA | 59.279053 | 0.143395041 |
| Metformin | | | SGLT2i | | 0 | 0 | 0.299325424 |
| Metformin | | | Thiazolidinedione | | 0 | 0 | 0.373893793 |
| Placebo | | | SGLT2i | | 35.03823 | 49.74408 | 0.003030283 |
| Placebo | | | Thiazolidinedione | | 48.68043 | 39.455288 | 0.214571423 |
| SGLT2i | | | Sulfonylurea | | 34.33832 | 27.341606 | 0.210460247 |
| Sulfonylurea | | | Thiazolidinedione | | 32.36684 | 68.203286 | 0.046288803 |
| **Global I-squared:** | | |  | |  |  |  |
|  | | |  | | 6.52 | 10.85 |  |
| **Pelvic Girdle Fracture** | | |  | |  |  |  |
| **Per-comparison I-squared:** | | |  | |  |  |  |
| DPP4i | | | GLP1RA | | NA | 0 | 0.6718587 |
| DPP4i | | | Metformin | | 0 | 0 | 0.6882409 |
| DPP4i | | | Placebo | | 73.84899 | 69.41276 | 0.9838927 |
| DPP4i | | | SGLT2i | | NA | 0 | 0.4514888 |
| DPP4i | | | Sulfonylurea | | 0 | 27.21457 | 0.2704643 |
| GLP1RA | | | Insulin | | NA | NA | NA |
| GLP1RA | | | Placebo | | 62.48074 | 59.81882 | 0.5164228 |
| GLP1RA | | | Sulfonylurea | | NA | 62.17367 | 0.1903767 |
| Metformin | | | SGLT2i | | NA | 0 | 0.7234915 |
| Placebo | | | SGLT2i | | 50.98964 | 44.04636 | 0.4923671 |
| Placebo | | | Thiazolidinedione | | NA | 0 | 0.9786389 |
| Sulfonylurea | | | Thiazolidinedione | | NA | 0 | 0.9848073 |
| **Global I-squared:** | | |  | |  |  |  |
|  | | |  | | 0 | 0 |  |
| **Cranial and Facial bone Fracture** | | |  | |  |  |  |
| **Per-comparison I-squared:** | | |  | |  |  |  |
| DPP4i | | | GLP1RA | | NA | 0 | 0.9010279 |
| DPP4i | | | Placebo | | 0 | 0 | 0.31925152 |
| DPP4i | | | Sulfonylurea | | 86.10484 | 80.72874 | 0.33266027 |
| GLP1RA | | | Placebo | | 0 | 0 | NA |
| Metformin | | | SGLT2i | | NA | NA | NA |
| Placebo | | | SGLT2i | | 28.81334 | 35.71904 | 0.09704908 |
| Placebo | | | Thiazolidinedione | | NA | 0 | 0.62929571 |
| SGLT2i | | | Sulfonylurea | | NA | 49.77244 | 0.24814322 |
| Sulfonylurea | | | Thiazolidinedione | | NA | 0 | 0.69830468 |
| **Global I-squared:** | | |  | |  |  |  |
|  | | |  | |  |  |  |
| **Thoracic Cage Fracture** | | |  | |  |  |  |
| **Per-comparison I-squared:** | | |  | |  |  |  |
| DPP4i | | | GLP1RA | | NA | 0 | 0.5730149 |
| DPP4i | | | Metformin | | NA | 0 | 0.7046115 |
| DPP4i | | | Placebo | | 0 | 0 | 0.7302385 |
| DPP4i | | | SGLT2i | | 0 | 0 | 0.6182597 |
| DPP4i | | | Sulfonylurea | | 0 | 0 | 0.7039957 |
| GLP1RA | | | Insulin | | 0 | 0 | NA |
| GLP1RA | | | Placebo | | 0 | 0 | 0.7719881 |
| GLP1RA | | | SGLT2i | | NA | 0 | 0.3865011 |
| Metformin | | | SGLT2i | | NA | 0 | 0.7822918 |
| Placebo | | | SGLT2i | | 0 | 0 | 0.3801875 |
| Placebo | | | Sulfonylurea | | NA | 0 | 0.7885643 |
| Placebo | | | Thiazolidinedione | | 57.59791 | 37.14544 | 0.3873561 |
| SGLT2i | | | Sulfonylurea | | 0 | 0 | 0.9564269 |
| Sulfonylurea | | | Thiazolidinedione | | NA | 0 | 0.4719542 |
| **Global I-squared:** | | |  | |  |  |  |
|  | | |  | | 0 | 0 |  |

Table S6. Network meta-analysis heterogeneity/inconsistency test(Fracture)_specific treatment

|  | **Treatment1** | **Treatment2** | **I2.pair** | **I2.cons** | **Incons.p** |
| --- | --- | --- | --- | --- | --- |
| **Overall Fracture** | | | | | |
| **Per-comparison I-squared:** | | | | | |
| Albiglutide | | Insulin | 41.0175772 | 4.55199 | 0.43056439 |
| Albiglutide | | Placebo | 0 | 0 | 0.28593405 |
| Albiglutide | | Sitagliptin | 34.1466933 | 0 | 0.68391118 |
| Albiglutide | | Sulfonylurea | NA | 0 | 0.38081607 |
| Alogliptin | | Placebo | 0 | 0 | 0.90773212 |
| Alogliptin | | Sulfonylurea | 0 | 0 | 0.79590363 |
| Alogliptin | | Thiazolidinedione | NA | 0 | 0.59341324 |
| Bexagliflozin | | Placebo | NA | 0 | 0.83011051 |
| Bexagliflozin | | Sitagliptin | NA | 0 | 0.93517051 |
| Canagliflozin | | Placebo | 36.2592427 | 29.80549 | 0.83937533 |
| Canagliflozin | | Semaglutide | NA | 0 | 0.9687791 |
| Canagliflozin | | Sitagliptin | 0 | 0 | 0.8201271 |
| Canagliflozin | | Sulfonylurea | NA | 0 | 0.96847582 |
| Dapagliflozin | | Enavogliflozin | NA | 0 | 0.4202505 |
| Dapagliflozin | | Exenatide | NA | 0 | 0.69656118 |
| Dapagliflozin | | Metformin | 0 | 0 | 0.61574731 |
| Dapagliflozin | | Placebo | 0 | 0 | 0.65369819 |
| Dapagliflozin | | Saxagliptin | 0 | 0 | 0.41043229 |
| Dapagliflozin | | Sulfonylurea | 0 | 0 | 0.42087136 |
| Dulaglutide | | Insulin | 0 | 0 | 0.33331293 |
| Dulaglutide | | Liraglutide | NA | 0 | 0.69824523 |
| Dulaglutide | | Metformin | NA | 0 | 0.60105109 |
| Dulaglutide | | Placebo | 0 | 0 | 0.65589233 |
| Dulaglutide | | Semaglutide | NA | 0 | 0.91724277 |
| Dulaglutide | | Sitagliptin | NA | 0 | 0.87659252 |
| Empagliflozin | | Linagliptin | NA | 0 | 0.87263184 |
| Empagliflozin | | Metformin | 0.3582643 | 27.81642 | 0.16065763 |
| Empagliflozin | | Placebo | 0 | 0 | 0.29746764 |
| Empagliflozin | | Semaglutide | NA | 0 | 0.80246701 |
| Empagliflozin | | Sitagliptin | NA | 0 | 0.52013504 |
| Empagliflozin | | Sulfonylurea | NA | 82.065128 | 0.02432262 |
| Enavogliflozin | | Placebo | NA | 1.476604 | 0.29639056 |
| Ertugliflozin | | Metformin | NA | 28.278563 | 0.30640302 |
| Ertugliflozin | | Placebo | 0 | 0 | 0.99769866 |
| Ertugliflozin | | Sitagliptin | NA | 0 | 0.79364714 |
| Ertugliflozin | | Sulfonylurea | NA | 10.468916 | 0.3144701 |
| Evogliptin | | Sitagliptin | NA | NA | NA |
| Exenatide | | Insulin | 0 | 0 | 0.37761322 |
| Exenatide | | Liraglutide | 0 | 0 | 0.49583879 |
| Exenatide | | Placebo | 0 | 0 | 0.88495328 |
| Exenatide | | Semaglutide | NA | 0 | 0.36738251 |
| Exenatide | | Sulfonylurea | NA | 0 | 0.47409638 |
| Insulin | | Liraglutide | 0 | 0 | 0.28308181 |
| Insulin | | Lixisenatide | 0 | 0 | 0.85274188 |
| Insulin | | Placebo | NA | 0 | 0.62595098 |
| Insulin | | Semaglutide | NA | 0 | 0.934994 |
| Ipragliflozin | | Placebo | NA | NA | NA |
| Linagliptin | | Metformin | 0 | 0 | 0.18911359 |
| Linagliptin | | Placebo | 0 | 0 | 0.76697027 |
| Linagliptin | | Sulfonylurea | 51.2524124 | 11.094948 | 0.55982104 |
| Linagliptin | | Thiazolidinedione | NA | 0 | 0.71131133 |
| Liraglutide | | Placebo | 0 | 0 | 0.99257507 |
| Liraglutide | | Semaglutide | NA | 0 | 0.41906791 |
| Liraglutide | | Sitagliptin | 0 | 0 | 0.27102603 |
| Liraglutide | | Sulfonylurea | 0 | 0 | 0.6202033 |
| Lixisenatide | | Placebo | 0 | 0 | 0.75496557 |
| Metformin | | Placebo | 0 | 0 | 0.54522859 |
| Metformin | | Saxagliptin | NA | 0 | 0.76541892 |
| Metformin | | Sitagliptin | 0 | 0 | 0.68950646 |
| Metformin | | Thiazolidinedione | 0 | 0 | 0.25826778 |
| Metformin | | Vildagliptin | NA | 0 | 0.8814991 |
| Omarigliptin | | Placebo | 0 | 0 | NA |
| Placebo | | Saxagliptin | 0 | 0 | 0.46489118 |
| Placebo | | Semaglutide | 0 | 0 | 0.70617616 |
| Placebo | | Sitagliptin | 0 | 0 | 0.42772 |
| Placebo | | Sotagliflozin | 10.9321219 | 11.469101 | NA |
| Placebo | | Sulfonylurea | 0 | 0 | 0.64428681 |
| Placebo | | Thiazolidinedione | 15.205231 | 1.330786 | 0.92528319 |
| Placebo | | Tirzepatide | NA | NA | NA |
| Placebo | | Tofogliflozin | NA | NA | NA |
| Placebo | | Vildagliptin | 0 | 0 | NA |
| Saxagliptin | | Sulfonylurea | 0 | 0 | 0.3846368 |
| Semaglutide | | Sitagliptin | NA | 0 | 0.40340273 |
| Sitagliptin | | Sulfonylurea | 0 | 0 | 0.77948336 |
| Sitagliptin | | Thiazolidinedione | 0 | 0 | 0.90341882 |
| Sulfonylurea | | Thiazolidinedione | 0 | 7.541829 | 0.22042857 |
| **Global I-squared:** | | | | | |
|  | |  | 0 | 0 |  |
|  | **Vertebral Fracture** | | | | |
|  | **Per-comparison I-squared:** | | | | |
|  | Albiglutide | Placebo | 0 | 0 | NA |
|  | Alogliptin | Placebo | NA | NA | NA |
|  | Canagliflozin | Placebo | 0 | 0 | 0.9431379 |
|  | Canagliflozin | Sitagliptin | NA | 0 | 0.9830096 |
|  | Dapagliflozin | Placebo | 0 | 0 | NA |
|  | Dulaglutide | Insulin | 0 | 0 | 0.9485176 |
|  | Dulaglutide | Placebo | 0 | 0 | 0.7528382 |
|  | Dulaglutide | Sitagliptin | NA | 0 | 0.5558126 |
|  | Empagliflozin | Placebo | 0 | 0 | NA |
|  | Ertugliflozin | Placebo | 0 | 0 | NA |
|  | Evogliptin | Sitagliptin | NA | NA | NA |
|  | Exenatide | Liraglutide | NA | NA | NA |
|  | Insulin | Liraglutide | NA | 0 | 0.9958124 |
|  | Linagliptin | Metformin | NA | 0 | 0.6128017 |
|  | Linagliptin | Placebo | 0 | 0 | 0.567114 |
|  | Linagliptin | Sulfonylurea | 0 | 0 | 0.4315582 |
|  | Liraglutide | Placebo | NA | 0 | 0.9855106 |
|  | Lixisenatide | Placebo | 0 | 0 | NA |
|  | Metformin | Placebo | NA | 0 | 0.6912243 |
|  | Metformin | Sitagliptin | NA | 0 | 0.7816023 |
|  | Omarigliptin | Placebo | NA | NA | NA |
|  | Placebo | Saxagliptin | NA | 0 | 0.4382983 |
|  | Placebo | Semaglutide | 0 | 0 | NA |
|  | Placebo | Sitagliptin | 0 | 0 | 0.6535373 |
|  | Placebo | Sotagliflozin | NA | NA | NA |
|  | Placebo | Thiazolidinedione | NA | 0 | 0.8660704 |
|  | Placebo | Tirzepatide | NA | NA | NA |
|  | Placebo | Vildagliptin | 0 | 0 | NA |
|  | Saxagliptin | Sulfonylurea | NA | 5.245302 | 0.3487445 |
|  | Sulfonylurea | Thiazolidinedione | 0 | 0 | 0.7890971 |
|  | **Global I-squared:** | | | | |
|  |  |  | 0 | 0 |  |
|  | **Non-Vertebral Fracture** |  |  |  |  |
|  | **Per-comparison I-squared:** |  |  |  |  |
|  | Albiglutide | Insulin | 0 | 49.138902 | 9.67E-02 |
|  | Albiglutide | Placebo | 46.5924748 | 45.427915 | 6.75E-01 |
|  | Albiglutide | Sitagliptin | 0 | 0 | 4.42E-01 |
|  | Albiglutide | Sulfonylurea | 0 | 0 | 5.98E-01 |
|  | Alogliptin | Placebo | 33.5828332 | 71.498011 | 1.11E-01 |
|  | Alogliptin | Sulfonylurea | 0 | 22.399585 | 2.06E-01 |
|  | Canagliflozin | Placebo | 92.152376 | 89.487731 | 1.78E-01 |
|  | Canagliflozin | Semaglutide | NA | 0 | 9.35E-01 |
|  | Canagliflozin | Sitagliptin | 0.6943128 | 16.862711 | 3.39E-01 |
|  | Canagliflozin | Sulfonylurea | NA | 33.971915 | 4.65E-01 |
|  | Dapagliflozin | Metformin | NA | 0 | 5.79E-01 |
|  | Dapagliflozin | Placebo | 22.7131658 | 49.144754 | 1.47E-02 |
|  | Dapagliflozin | Saxagliptin | 0 | 0 | 8.82E-01 |
|  | Dapagliflozin | Sulfonylurea | 82.6340279 | 90.64578 | 7.25E-03 |
|  | Dulaglutide | Insulin | 45.2789745 | 37.136069 | 4.21E-01 |
|  | Dulaglutide | Liraglutide | NA | 23.616821 | 3.31E-01 |
|  | Dulaglutide | Metformin | NA | 0 | 8.22E-01 |
|  | Dulaglutide | Placebo | 0 | 25.452539 | 1.46E-01 |
|  | Dulaglutide | Sulfonylurea | NA | 0 | 7.42E-01 |
|  | Empagliflozin | Linagliptin | NA | 0 | 7.10E-01 |
|  | Empagliflozin | Metformin | NA | 95.066391 | 8.25E-03 |
|  | Empagliflozin | Placebo | 14.3708015 | 74.730333 | 7.80E-05 |
|  | Empagliflozin | Semaglutide | NA | 0 | 9.56E-01 |
|  | Empagliflozin | Sulfonylurea | NA | 90.17396 | 5.69E-02 |
|  | Enavogliflozin | Metformin | NA | 0 | 5.17E-01 |
|  | Enavogliflozin | Placebo | NA | 0 | 6.76E-01 |
|  | Enavogliflozin | Sitagliptin | NA | 0 | 7.64E-01 |
|  | Ertugliflozin | Placebo | 72.4594003 | 48.680627 | 4.77E-01 |
|  | Ertugliflozin | Sitagliptin | NA | 0 | 6.74E-01 |
|  | Ertugliflozin | Sulfonylurea | NA | 0 | 5.61E-01 |
|  | Exenatide | Insulin | 0 | 8.479711 | 8.74E-02 |
|  | Exenatide | Placebo | 0 | 0 | 5.33E-01 |
|  | Exenatide | Sulfonylurea | NA | 40.423341 | 4.08E-01 |
|  | Insulin | Liraglutide | 75.2194753 | 50.728699 | 8.77E-01 |
|  | Insulin | Lixisenatide | NA | 0 | 7.00E-01 |
|  | Insulin | Placebo | NA | 0 | 4.78E-01 |
|  | Linagliptin | Metformin | 0 | 50.926901 | 4.30E-02 |
|  | Linagliptin | Placebo | 0 | 0 | 3.99E-01 |
|  | Linagliptin | Sulfonylurea | 85.918594 | 86.260615 | 4.09E-01 |
|  | Liraglutide | Placebo | 63.1689359 | 42.520202 | 9.67E-01 |
|  | Liraglutide | Sitagliptin | NA | 0 | 5.29E-01 |
|  | Liraglutide | Sulfonylurea | 45.59433 | 29.26851 | 3.51E-01 |
|  | Lixisenatide | Placebo | 0 | 0 | NA |
|  | Metformin | Placebo | 0 | 0 | 9.00E-01 |
|  | Metformin | Saxagliptin | NA | 0 | 9.92E-01 |
|  | Metformin | Sitagliptin | 0 | 58.580678 | 5.70E-02 |
|  | Metformin | Thiazolidinedione | 38.1306585 | 92.765628 | 2.49E-03 |
|  | Metformin | Vildagliptin | NA | 0 | 6.76E-01 |
|  | Omarigliptin | Placebo | 0 | 0 | NA |
|  | Placebo | Saxagliptin | 0 | 0 | 3.54E-01 |
|  | Placebo | Semaglutide | 0 | 0 | 6.91E-01 |
|  | Placebo | Sitagliptin | 0 | 0 | 6.76E-01 |
|  | Placebo | Sotagliflozin | 8.5760845 | 30.024089 | NA |
|  | Placebo | Sulfonylurea | 67.9265062 | 36.272411 | 9.80E-01 |
|  | Placebo | Thiazolidinedione | 76.2909384 | 70.367611 | 9.61E-01 |
|  | Placebo | Tofogliflozin | NA | NA | NA |
|  | Placebo | Vildagliptin | 66.8861761 | 30.730067 | 8.71E-01 |
|  | Saxagliptin | Sulfonylurea | 79.0867536 | 72.286295 | 2.33E-01 |
|  | Semaglutide | Sitagliptin | NA | 0 | 8.15E-01 |
|  | Sitagliptin | Sulfonylurea | 32.5610738 | 21.307435 | 5.69E-01 |
|  | Sitagliptin | Thiazolidinedione | 69.4394525 | 64.140767 | 1.77E-01 |
|  | Sulfonylurea | Thiazolidinedione | 90.6330415 | 62.187647 | 2.82E-01 |
|  | **Global I-squared:** |  |  |  |  |
|  |  |  | 0 | 0 |  |
| **Hip Fracture** | |  |  |  |  |
| **Per-comparison I-squared:** | |  |  |  |  |
| Albiglutide | | Insulin | NA | 0 | 0.4913316 |
| Albiglutide | | Placebo | 0 | 0 | 0.3610492 |
| Albiglutide | | Sitagliptin | NA | 0 | 0.7947848 |
| Albiglutide | | Sulfonylurea | NA | 0 | 0.4828305 |
| Alogliptin | | Placebo | NA | 0 | 0.7472743 |
| Alogliptin | | Sulfonylurea | NA | 0 | 0.8569482 |
| Canagliflozin | | Placebo | 74.23724 | 56.08632 | 0.6160555 |
| Canagliflozin | | Semaglutide | NA | 0 | 0.5108343 |
| Canagliflozin | | Sitagliptin | NA | 0 | 0.7087029 |
| Canagliflozin | | Sulfonylurea | NA | 0 | 0.8677097 |
| Dapagliflozin | | Placebo | 0 | 0 | NA |
| Dapagliflozin | | Saxagliptin | NA | 0 | 0.7537001 |
| Dapagliflozin | | Sulfonylurea | NA | 0 | 0.4209486 |
| Dulaglutide | | Placebo | 0 | 0 | NA |
| Empagliflozin | | Metformin | NA | 0 | 0.7992746 |
| Empagliflozin | | Placebo | 0 | 0 | 0.3845097 |
| Empagliflozin | | Semaglutide | NA | 0 | 0.6320159 |
| Empagliflozin | | Sulfonylurea | NA | 46.06195 | 0.2292053 |
| Ertugliflozin | | Placebo | 0 | 0 | NA |
| Exenatide | | Insulin | NA | 0 | 0.6158585 |
| Exenatide | | Sulfonylurea | NA | 0 | 0.7224767 |
| Insulin | | Liraglutide | NA | 0 | 0.7325459 |
| Linagliptin | | Metformin | 12.39863 | 15.89931 | 0.2042878 |
| Linagliptin | | Placebo | 0 | 0 | 0.8893474 |
| Linagliptin | | Sulfonylurea | 71.87324 | 56.95441 | 0.6087555 |
| Liraglutide | | Placebo | NA | 0 | 0.9471467 |
| Liraglutide | | Sitagliptin | NA | 0 | 0.8404353 |
| Liraglutide | | Sulfonylurea | NA | 0 | 0.6618087 |
| Lixisenatide | | Placebo | 58.17489 | 55.93421 | NA |
| Metformin | | Placebo | 0 | 0 | 0.5692038 |
| Metformin | | Sitagliptin | 0 | 0 | 0.8421894 |
| Metformin | | Vildagliptin | NA | 0 | 0.9389906 |
| Omarigliptin | | Placebo | 0 | 0 | NA |
| Placebo | | Saxagliptin | 0 | 0 | 0.7900619 |
| Placebo | | Semaglutide | 0 | 0 | 0.543188 |
| Placebo | | Sitagliptin | 0 | 0 | 0.9550875 |
| Placebo | | Sotagliflozin | 0 | 0 | NA |
| Placebo | | Sulfonylurea | 0 | 0 | 0.7192996 |
| Placebo | | Thiazolidinedione | NA | 0 | 0.7611967 |
| Placebo | | Vildagliptin | 75.24799 | 50.40461 | 0.9476195 |
| Saxagliptin | | Sulfonylurea | 0 | 0 | 0.7201484 |
| Semaglutide | | Sitagliptin | NA | 0 | 0.7593105 |
| Sitagliptin | | Sulfonylurea | 0 | 0 | 0.8861927 |
| Sulfonylurea | | Thiazolidinedione | NA | 0 | 0.7393493 |
| **Global I-squared:** | |  |  |  |  |
|  | |  | 12.85 | 7.75 |  |
| **Vertebral + Hip Fracture** | |  |  |  |  |
| **Per-comparison I-squared:** | |  |  |  |  |
| Albiglutide | | Insulin | NA | 0 | 0.766245 |
| Albiglutide | | Placebo | 0 | 0 | 0.887389 |
| Albiglutide | | Sitagliptin | NA | 0 | 0.923693 |
| Albiglutide | | Sulfonylurea | NA | 0 | 0.632774 |
| Alogliptin | | Placebo | NA | 0 | 0.922532 |
| Alogliptin | | Sulfonylurea | NA | 0 | 0.963363 |
| Canagliflozin | | Placebo | 76.69407 | 62.49216 | 0.803056 |
| Canagliflozin | | Semaglutide | NA | 0 | 0.599599 |
| Canagliflozin | | Sitagliptin | 0 | 0 | 0.854274 |
| Canagliflozin | | Sulfonylurea | NA | 0 | 0.980586 |
| Dapagliflozin | | Placebo | 0 | 0 | NA |
| Dapagliflozin | | Saxagliptin | NA | 0 | 0.542398 |
| Dapagliflozin | | Sulfonylurea | NA | 0 | 0.519159 |
| Dulaglutide | | Insulin | 0 | 0 | 0.327318 |
| Dulaglutide | | Placebo | 0 | 7.038708 | 0.541301 |
| Dulaglutide | | Sitagliptin | NA | 0 | 0.83333 |
| Empagliflozin | | Metformin | NA | 10.59639 | 0.360353 |
| Empagliflozin | | Placebo | 0 | 14.83471 | 0.328746 |
| Empagliflozin | | Semaglutide | NA | 0 | 0.880645 |
| Empagliflozin | | Sulfonylurea | NA | 2.473578 | 0.387737 |
| Ertugliflozin | | Placebo | 86.04881 | 85.54019 | NA |
| Evogliptin | | Sitagliptin | NA | NA | NA |
| Exenatide | | Insulin | NA | 0 | 0.636466 |
| Exenatide | | Liraglutide | NA | 0 | 0.889199 |
| Exenatide | | Sulfonylurea | NA | 0 | 0.585551 |
| Insulin | | Liraglutide | 0 | 0 | 0.609253 |
| Linagliptin | | Metformin | 0 | 0 | 0.69299 |
| Linagliptin | | Placebo | 77.35555 | 74.00031 | 0.957188 |
| Linagliptin | | Sulfonylurea | 78.48888 | 58.42299 | 0.963045 |
| Liraglutide | | Placebo | NA | 0 | 0.648758 |
| Liraglutide | | Sitagliptin | NA | 0 | 0.676293 |
| Liraglutide | | Sulfonylurea | NA | 0 | 0.49968 |
| Lixisenatide | | Placebo | 0 | 0 | NA |
| Metformin | | Placebo | 0 | 0 | 0.64157 |
| Metformin | | Sitagliptin | 0 | 0 | 0.594999 |
| Metformin | | Vildagliptin | NA | 0 | 0.512726 |
| Omarigliptin | | Placebo | 0 | 0 | NA |
| Placebo | | Saxagliptin | 46.85984 | 59.0527 | 0.195997 |
| Placebo | | Semaglutide | 0 | 0 | 0.870515 |
| Placebo | | Sitagliptin | 0 | 4.990337 | 0.355496 |
| Placebo | | Sotagliflozin | 0 | 0 | NA |
| Placebo | | Sulfonylurea | 0 | 0 | 0.788909 |
| Placebo | | Thiazolidinedione | 0 | 0 | 0.536525 |
| Placebo | | Tirzepatide | NA | NA | NA |
| Placebo | | Vildagliptin | 0 | 0 | 0.575241 |
| Saxagliptin | | Sulfonylurea | 0 | 0 | 0.176355 |
| Semaglutide | | Sitagliptin | NA | 0 | 0.830842 |
| Sitagliptin | | Sulfonylurea | 0 | 0 | 0.653399 |
| Sulfonylurea | | Thiazolidinedione | 56.90298 | 46.51217 | 0.536862 |
| **Global I-squared:** | |  |  |  |  |
|  | |  | 0 | 0 |  |
| **Multiple Fracture** | |  |  |  |  |
| **Per-comparison I-squared:** | |  |  |  |  |
| Alogliptin | | Placebo | NA | 0 | 0.7718209 |
| Alogliptin | | Sulfonylurea | NA | 0 | 0.8152215 |
| Dapagliflozin | | Placebo | NA | NA | NA |
| Dulaglutide | | Placebo | NA | NA | NA |
| Empagliflozin | | Placebo | 0 | 0 | NA |
| Linagliptin | | Placebo | NA | 0 | 0.9246529 |
| Linagliptin | | Sulfonylurea | NA | 0 | 0.7795359 |
| Liraglutide | | Placebo | NA | NA | NA |
| Lixisenatide | | Placebo | NA | NA | NA |
| Placebo | | Saxagliptin | 0 | 0 | NA |
| Placebo | | Sotagliflozin | NA | NA | NA |
| Rosiglitazone | | Sulfonylurea | NA | NA | NA |
| **Global I-squared:** | |  |  |  |  |
|  | |  | 0 | 0 |  |
| **Vertebral Column Fracture** | | | | | |
| **Per-comparison I-squared:** | | | | | |
| Albiglutide | | Placebo | 0 | 0 | NA |
| Alogliptin | | Placebo | NA | NA | NA |
| Canagliflozin | | Placebo | 0 | 0 | 0.9431379 |
| Canagliflozin | | Sitagliptin | NA | 0 | 0.9830096 |
| Dapagliflozin | | Placebo | 0 | 0 | NA |
| Dulaglutide | | Insulin | 0 | 0 | 0.9485176 |
| Dulaglutide | | Placebo | 0 | 0 | 0.7528382 |
| Dulaglutide | | Sitagliptin | NA | 0 | 0.5558126 |
| Empagliflozin | | Placebo | 0 | 0 | NA |
| Ertugliflozin | | Placebo | 0 | 0 | NA |
| Evogliptin | | Sitagliptin | NA | NA | NA |
| Exenatide | | Liraglutide | NA | NA | NA |
| Insulin | | Liraglutide | NA | 0 | 0.9958124 |
| Linagliptin | | Metformin | NA | 0 | 0.6128017 |
| Linagliptin | | Placebo | 0 | 0 | 0.567114 |
| Linagliptin | | Sulfonylurea | 0 | 0 | 0.4315582 |
| Liraglutide | | Placebo | NA | 0 | 0.9855106 |
| Lixisenatide | | Placebo | 0 | 0 | NA |
| Metformin | | Placebo | NA | 0 | 0.6912243 |
| Metformin | | Sitagliptin | NA | 0 | 0.7816023 |
| Omarigliptin | | Placebo | NA | NA | NA |
| Placebo | | Saxagliptin | NA | 0 | 0.4382983 |
| Placebo | | Semaglutide | 0 | 0 | NA |
| Placebo | | Sitagliptin | 0 | 0 | 0.6535373 |
| Placebo | | Sotagliflozin | NA | NA | NA |
| Placebo | | Thiazolidinedione | NA | 0 | 0.8660704 |
| Placebo | | Tirzepatide | NA | NA | NA |
| Placebo | | Vildagliptin | 0 | 0 | NA |
| Saxagliptin | | Sulfonylurea | NA | 5.245302 | 0.3487445 |
| Sulfonylurea | | Thiazolidinedione | 0 | 0 | 0.7890971 |
| **Global I-squared:** | | | | | |
|  | |  | 0 | 0 |  |
| **Lower Extremity Fracture** | |  |  |  |  |
| **Per-comparison I-squared:** | |  |  |  |  |
| Albiglutide | | Insulin | 0 | 0 | 9.77E-01 |
| Albiglutide | | Placebo | 0 | 0 | 6.31E-01 |
| Albiglutide | | Sitagliptin | NA | 0 | 8.96E-01 |
| Albiglutide | | Sulfonylurea | NA | 0 | 4.46E-01 |
| Alogliptin | | Placebo | 0 | 36.761926 | 3.60E-02 |
| Alogliptin | | Sulfonylurea | NA | 58.341024 | 1.35E-01 |
| Canagliflozin | | Placebo | 19.87679 | 52.970052 | 1.20E-02 |
| Canagliflozin | | Semaglutide | NA | 0 | 6.69E-01 |
| Canagliflozin | | Sitagliptin | NA | 0 | 7.87E-01 |
| Canagliflozin | | Sulfonylurea | NA | 0 | 9.69E-01 |
| Dapagliflozin | | Placebo | 0 | 0 | NA |
| Dapagliflozin | | Saxagliptin | 0 | 0 | 3.57E-01 |
| Dapagliflozin | | Sulfonylurea | 40.20345 | 10.322067 | 9.89E-01 |
| Dulaglutide | | Insulin | 0 | 0 | 5.10E-01 |
| Dulaglutide | | Placebo | NA | NA | NA |
| Dulaglutide | | Semaglutide | NA | 0 | 9.34E-01 |
| Dulaglutide | | Sulfonylurea | NA | 0 | 9.61E-01 |
| Empagliflozin | | Linagliptin | NA | 0 | 9.80E-01 |
| Empagliflozin | | Metformin | NA | 82.812755 | 2.27E-02 |
| Empagliflozin | | Placebo | 0 | 62.391781 | 5.12E-05 |
| Empagliflozin | | Semaglutide | NA | 0 | 8.95E-01 |
| Empagliflozin | | Sulfonylurea | NA | 84.570321 | 2.08E-02 |
| Ertugliflozin | | Placebo | 0 | 0 | NA |
| Exenatide | | Insulin | 0 | 0 | 3.66E-01 |
| Exenatide | | Liraglutide | NA | 0 | 7.06E-01 |
| Exenatide | | Placebo | NA | 2.884569 | 3.78E-01 |
| Exenatide | | Sulfonylurea | NA | 0 | 7.08E-01 |
| Insulin | | Liraglutide | 0 | 0 | 7.46E-01 |
| Insulin | | Lixisenatide | NA | 0 | 6.44E-01 |
| Linagliptin | | Metformin | 24.3687 | 6.979946 | 3.13E-01 |
| Linagliptin | | Placebo | 0 | 0 | 7.86E-01 |
| Linagliptin | | Sulfonylurea | 23.05166 | 51.479427 | 1.31E-01 |
| Liraglutide | | Placebo | 0 | 0 | 2.79E-01 |
| Liraglutide | | Semaglutide | NA | 0 | 4.38E-01 |
| Liraglutide | | Sitagliptin | NA | 0 | 5.38E-01 |
| Liraglutide | | Sulfonylurea | NA | 0 | 4.54E-01 |
| Lixisenatide | | Placebo | 0 | 0 | NA |
| Metformin | | Placebo | 0 | 0 | 3.02E-01 |
| Metformin | | Sitagliptin | 0 | 18.553632 | 1.47E-01 |
| Omarigliptin | | Placebo | 0 | 0 | NA |
| Placebo | | Saxagliptin | 0 | 0 | 3.12E-02 |
| Placebo | | Semaglutide | 0 | 0 | 9.47E-01 |
| Placebo | | Sitagliptin | 0 | 35.534148 | 1.19E-02 |
| Placebo | | Sotagliflozin | 0 | 0 | NA |
| Placebo | | Sulfonylurea | 69.54408 | 39.370645 | 9.15E-01 |
| Placebo | | Thiazolidinedione | 0 | 0 | 3.99E-01 |
| Placebo | | Tofogliflozin | NA | NA | NA |
| Placebo | | Vildagliptin | 28.51042 | 28.398945 | NA |
| Saxagliptin | | Sulfonylurea | 66.07836 | 64.655755 | 1.08E-01 |
| Semaglutide | | Sitagliptin | NA | 0 | 9.09E-01 |
| Sitagliptin | | Sulfonylurea | 7.548 | 32.989095 | 1.04E-01 |
| Sitagliptin | | Thiazolidinedione | NA | 0 | 3.21E-01 |
| Sulfonylurea | | Thiazolidinedione | 80.72465 | 62.169473 | 5.98E-01 |
| **Global I-squared:** | |  |  |  |  |
|  | |  | 0 | 0 |  |
| **Upper Extremity Fracture** | |  |  |  |  |
| **Per-comparison I-squared:** | |  |  |  |  |
| Albiglutide | | Placebo | NA | 0 | 0.55530467 |
| Albiglutide | | Sulfonylurea | NA | 0 | 0.52814107 |
| Alogliptin | | Placebo | NA | 49.43008 | 0.18266261 |
| Alogliptin | | Sulfonylurea | NA | 38.94543 | 0.25281024 |
| Canagliflozin | | Placebo | 65.0361973 | 47.49968 | 0.96907173 |
| Canagliflozin | | Sitagliptin | NA | 0 | 0.99874426 |
| Canagliflozin | | Sulfonylurea | NA | 0 | 0.99161046 |
| Dapagliflozin | | Metformin | NA | 0 | 0.38826208 |
| Dapagliflozin | | Placebo | 57.2654194 | 56.01326 | 0.53773043 |
| Dapagliflozin | | Sulfonylurea | NA | 0 | 0.56818929 |
| Dulaglutide | | Insulin | 17.9183519 | 0 | 0.7645592 |
| Dulaglutide | | Liraglutide | NA | 0 | 0.50523297 |
| Dulaglutide | | Metformin | NA | 0 | 0.75169582 |
| Dulaglutide | | Placebo | 0 | 0 | 0.93461649 |
| Empagliflozin | | Placebo | 0 | 0 | 0.84708681 |
| Empagliflozin | | Sulfonylurea | NA | 0 | 0.69784144 |
| Enavogliflozin | | Metformin | NA | 0 | 0.30566473 |
| Enavogliflozin | | Placebo | NA | 0 | 0.72191048 |
| Enavogliflozin | | Sitagliptin | NA | 0 | 0.59907389 |
| Ertugliflozin | | Placebo | 73.1464265 | 55.13627 | 0.19234722 |
| Ertugliflozin | | Sitagliptin | NA | 0 | 0.78671022 |
| Ertugliflozin | | Sulfonylurea | NA | 62.79601 | 0.14590442 |
| Exenatide | | Insulin | 0 | 0 | 0.38516353 |
| Exenatide | | Placebo | NA | 0 | 0.72174637 |
| Exenatide | | Sulfonylurea | NA | 0 | 0.35601248 |
| Insulin | | Placebo | NA | 6.20074 | 0.37688428 |
| Linagliptin | | Placebo | 0 | 0 | 0.61111773 |
| Linagliptin | | Sulfonylurea | 0 | 0 | 0.65212235 |
| Liraglutide | | Placebo | 0 | 0 | 0.59314779 |
| Liraglutide | | Sulfonylurea | 0 | 0 | 0.58281496 |
| Lixisenatide | | Placebo | 0 | 0 | NA |
| Metformin | | Saxagliptin | NA | 0 | 0.70713046 |
| Metformin | | Sitagliptin | 0 | 0 | 0.57380765 |
| Metformin | | Thiazolidinedione | 0 | 0 | 0.38379395 |
| Omarigliptin | | Placebo | NA | NA | NA |
| Placebo | | Saxagliptin | 53.8125466 | 43.78935 | 0.84082135 |
| Placebo | | Semaglutide | 0 | 0 | NA |
| Placebo | | Sitagliptin | 0 | 0 | 0.6811887 |
| Placebo | | Sotagliflozin | NA | NA | NA |
| Placebo | | Thiazolidinedione | 49.0017467 | 39.1587 | 0.22354046 |
| Placebo | | Vildagliptin | NA | NA | NA |
| Saxagliptin | | Sulfonylurea | 0 | 0 | 0.56489871 |
| Sitagliptin | | Sulfonylurea | 0.8603506 | 0 | 0.57595548 |
| Sitagliptin | | Thiazolidinedione | 0 | 62.11156 | 0.02582185 |
| Sulfonylurea | | Thiazolidinedione | 34.5639346 | 65.41025 | 0.12106524 |
| **Global I-squared:** | |  |  |  |  |
|  | |  | 12.85 | 7.75 |  |
| **Pelvic Girdle Fracture** | |  |  |  |  |
| **Per-comparison I-squared:** | |  |  |  |  |
| Albiglutide | | Insulin | NA | NA | NA |
| Albiglutide | | Placebo | NA | NA | NA |
| Canagliflozin | | Placebo | 45.84549 | 16.4239 | 0.9143585 |
| Canagliflozin | | Sitagliptin | NA | 0 | 0.4746609 |
| Dapagliflozin | | Placebo | 38.15436 | 36.67281 | NA |
| Dulaglutide | | Placebo | 0 | 0 | NA |
| Empagliflozin | | Metformin | NA | 0 | 0.5968621 |
| Empagliflozin | | Placebo | 0 | 0 | 0.6960306 |
| Ertugliflozin | | Placebo | NA | NA | NA |
| Exenatide | | Sulfonylurea | NA | NA | NA |
| Linagliptin | | Metformin | NA | 14.28047 | 0.3119266 |
| Linagliptin | | Placebo | NA | 13.63603 | 0.4644016 |
| Linagliptin | | Sulfonylurea | NA | 0 | 0.8787192 |
| Liraglutide | | Placebo | NA | NA | NA |
| Lixisenatide | | Placebo | 76.07596 | 75.8947 | NA |
| Metformin | | Vildagliptin | NA | 0 | 0.6841268 |
| Omarigliptin | | Placebo | NA | NA | NA |
| Placebo | | Saxagliptin | 0 | 0 | NA |
| Placebo | | Semaglutide | NA | 0 | 0.9795692 |
| Placebo | | Sitagliptin | 0 | 0 | 0.8036544 |
| Placebo | | Sotagliflozin | 0 | 0 | NA |
| Placebo | | Thiazolidinedione | NA | 0 | 0.6060078 |
| Placebo | | Vildagliptin | NA | 0 | 0.6460678 |
| Semaglutide | | Sitagliptin | NA | 0 | 0.9817206 |
| Sitagliptin | | Sulfonylurea | NA | 3.7976 | 0.3903596 |
| Sulfonylurea | | Thiazolidinedione | NA | 0 | 0.7924347 |
| **Global I-squared:** | |  |  |  |  |
|  | |  | 9.88 | 0 |  |
| **Cranial and Facial bone Fracture** | |  |  |  |  |
| **Per-comparison I-squared:** | |  |  |  |  |
| Alogliptin | | Sulfonylurea | NA | NA | NA |
| Canagliflozin | | Placebo | 34.05849 | 7.195268 | 0.3327228 |
| Canagliflozin | | Sulfonylurea | NA | 37.683058 | 0.3321499 |
| Dapagliflozin | | Metformin | NA | NA | NA |
| Dapagliflozin | | Placebo | NA | NA | NA |
| Dulaglutide | | Placebo | 0 | 0 | NA |
| Dulaglutide | | Sitagliptin | NA | 0 | 0.9554914 |
| Empagliflozin | | Placebo | 0 | 0 | NA |
| Ertugliflozin | | Placebo | NA | NA | NA |
| Linagliptin | | Placebo | NA | 0 | 0.5667873 |
| Linagliptin | | Sulfonylurea | NA | 0 | 0.6615172 |
| Liraglutide | | Placebo | NA | NA | NA |
| Lixisenatide | | Placebo | 25.35167 | 26.313489 | NA |
| Placebo | | Saxagliptin | NA | NA | NA |
| Placebo | | Semaglutide | 0 | 0 | NA |
| Placebo | | Sitagliptin | 16.72522 | 14.229068 | NA |
| Placebo | | Sotagliflozin | NA | NA | NA |
| Placebo | | Thiazolidinedione | NA | 0 | 0.8447707 |
| Placebo | | Tirzepatide | NA | NA | NA |
| Placebo | | Vildagliptin | NA | NA | NA |
| Sulfonylurea | | Thiazolidinedione | NA | 0 | 0.9433403 |
| **Global I-squared:** | |  |  |  |  |
|  | |  | 0 | 0 |  |
| **Thoracic Cage Fracture** | |  |  |  |  |
| **Per-comparison I-squared:** | |  |  |  |  |
| Albiglutide | | Placebo | 0 | 0 | NA |
| Alogliptin | | Placebo | NA | NA | NA |
| Canagliflozin | | Placebo | 0 | 0 | NA |
| Dapagliflozin | | Exenatide | NA | 0 | 0.8639993 |
| Dapagliflozin | | Placebo | 0 | 0 | 0.8824671 |
| Dapagliflozin | | Saxagliptin | NA | 0 | 0.7370516 |
| Dapagliflozin | | Sulfonylurea | 0 | 0 | 0.707856 |
| Dulaglutide | | Insulin | 0 | 0 | NA |
| Dulaglutide | | Liraglutide | NA | 0 | 0.8742682 |
| Dulaglutide | | Placebo | 0 | 0 | 0.9900078 |
| Empagliflozin | | Linagliptin | NA | 0 | 0.8687538 |
| Empagliflozin | | Metformin | NA | 0 | 0.8654016 |
| Empagliflozin | | Placebo | 0 | 0 | 0.9227741 |
| Ertugliflozin | | Placebo | NA | 0 | 0.5283345 |
| Ertugliflozin | | Sitagliptin | NA | 0 | 0.4459547 |
| Exenatide | | Liraglutide | NA | 0 | 0.8239155 |
| Exenatide | | Placebo | NA | 0 | 0.7878575 |
| Linagliptin | | Metformin | NA | 0 | 0.9153511 |
| Linagliptin | | Placebo | 0 | 0 | 0.4670805 |
| Linagliptin | | Sulfonylurea | NA | 0 | 0.4696744 |
| Liraglutide | | Placebo | NA | 0 | 0.9006727 |
| Liraglutide | | Sitagliptin | NA | 0 | 0.8218858 |
| Lixisenatide | | Placebo | 0 | 0 | NA |
| Omarigliptin | | Placebo | 0 | 0 | NA |
| Placebo | | Saxagliptin | 0 | 0 | NA |
| Placebo | | Semaglutide | 0 | 0 | NA |
| Placebo | | Sitagliptin | 0 | 0 | 0.4949159 |
| Placebo | | Sotagliflozin | 0 | 0 | NA |
| Placebo | | Sulfonylurea | NA | 0 | 0.8656067 |
| Placebo | | Thiazolidinedione | 56.79714 | 36.39363 | 0.40657 |
| Placebo | | Vildagliptin | NA | NA | NA |
| Saxagliptin | | Sulfonylurea | NA | 0 | 0.9058514 |
| Sulfonylurea | | Thiazolidinedione | NA | 0 | 0.4222614 |
| **Global I-squared:** | |  |  |  |  |
|  | |  | 0 | 0 |  |

I2.pair: I-square of pair-wise meta-analysis; i2.cons: I-square of network meta-analysis; Incons.p: inconsistency p-values for pair-wise and network meta-analysis; NA: not applicable

Table S7. Network meta-analysis heterogeneity/inconsistency test (BMD)_Treatment type

|  | **Treatment1** | **Treatment2** | **I2.pair** | **I2.cons** | **Incons.p** |
| --- | --- | --- | --- | --- | --- |
|  | **Total hip** |  |  |  |  |
|  | **Per-comparison I-squared:** |  |  |  |  |
|  | GLP1RA | Placebo | NA | NA | NA |
|  | Metformin | Placebo | NA | 97.819774 | 0.9546497 |
|  | Metformin | Thiazolidinedione | NA | 0 | 0.928786 |
|  | Placebo | SGLT2i | 37.16162 | 37.148497 | NA |
|  | Placebo | Thiazolidinedione | 44.13465 | 1.267063 | 0.6613643 |
| **Global I-squared:** | |  |  |  |  |
|  | |  | 39.64 | 50.88 |  |
|  | **Lumber spine** |  |  |  |  |
|  | **Per-comparison I-squared:** |  |  |  |  |
|  | Metformin | Placebo | NA | 97.88428 | 0.9133141 |
|  | Metformin | Thiazolidinedione | 89.003685 | 79.92967 | 0.9263471 |
|  | Placebo | SGLT2i | 0 | 0 | NA |
|  | Placebo | Thiazolidinedione | 6.212393 | 0 | 0.5727481 |
|  | **Global I-squared:** |  |  |  |  |
|  |  |  | 41.26 | 49.99 |  |
|  | **Femoral neck** |  |  |  |  |
|  | **Per-comparison I-squared:** |  |  |  |  |
|  | Metformin | Placebo | NA | 99.96652 | 0.7129343 |
|  | Metformin | Thiazolidinedione | 99.91695 | 99.80578 | 0.7596195 |
|  | Placebo | SGLT2i | 35.20475 | 35.09255 | NA |
|  | Placebo | Thiazolidinedione | 0 | 0 | 0.6299151 |
|  | **Global I-squared:** |  |  |  |  |
|  |  |  | 80.56 | 88.85 |  |

I2.pair: I-square of pair-wise meta-analysis; i2.cons: I-square of network meta-analysis; Incons.p: inconsistency p-values for pair-wise and network meta-analysis; NA: not applicable

Table S8. Network meta-analysis heterogeneity/inconsistency test(BMD)_specific treatment

|  | **Treatment1** | **Treatment2** | | **I2.pair** | | **I2.cons** | **Incons.p** |
| --- | --- | --- | --- | --- | --- | --- | --- |
| **Total hip BMD** | |  | |  | |  |  |
| **Per-comparison I-squared:** | |  | |  | |  |  |
| Canagliflozin | | Placebo | | NA | | NA | NA |
| Dapagliflozin | | Placebo | | NA | | NA | NA |
| Ertugliflozin | | Placebo | | NA | | NA | NA |
| Liraglutide | | Placebo | | NA | | NA | NA |
| Metformin | | Placebo | | NA | | 97.565352 | 0.9705258 |
| Metformin | | Thiazolidinedione | | NA | | 0 | 0.9507434 |
| Placebo | | Sotagliflozin | | NA | | NA | NA |
| Placebo | | Thiazolidinedione | | 45.22589 | | 3.113988 | 0.699855 |
| **Global I-squared:** | | |  | |  |  |  |
|  | | |  | | 28.31 | 26.94 |  |
| **Lumber spine BMD** | |  | |  | |  |  |
| **Per-comparison I-squared:** | |  | |  | |  |  |
| Canagliflozin | | Placebo | | NA | | NA | NA |
| Dapagliflozin | | Placebo | | NA | | NA | NA |
| Ertuglilozin | | Placebo | | NA | | NA | NA |
| Metformin | | Placebo | | NA | | 99.12587 | 0.9631348 |
| Metformin | | Thiazolidinedione | | 89.265335 | | 81.49966 | 0.971384 |
| Placebo | | Sotagliflozin | | NA | | NA | NA |
| Placebo | | Thiazolidinedione | | 7.323356 | | 0 | 0.7458423 |
| **Global I-squared:** | | |  | |  |  |  |
|  | | |  | | 41.28 | 39.58 |  |
| **Femoral neck BMD** | |  | |  | |  |  |
| **Per-comparison I-squared:** | |  | |  | |  |  |
| Canagliflozin | | Placebo | | NA | | NA | NA |
| Dapagliflozin | | Placebo | | NA | | NA | NA |
| Ertugliflozin | | Placebo | | NA | | NA | NA |
| Metformin | | Placebo | | NA | | 99.9744 | 0.6998943 |
| Metformin | | Thiazolidinedione | | 99.91442 | | 99.80062 | 0.7955145 |
| Placebo | | Sotagliflozin | | NA | | NA | NA |
| Placebo | | Thiazolidinedione | | 0 | | 0 | 0.6910147 |
| Canagliflozin | | Placebo | | NA | | NA | NA |
| **Global I-squared:** | | |  | |  |  |  |
|  | | |  | | 50.23 | 58.92 |  |

I2.pair: I-square of pair-wise meta-analysis; i2.cons: I-square of network meta-analysis; Incons.p: inconsistency p-values for pair-wise and network meta-analysis; NA: not applicable

Table S9. Network estimated effect sizes (95% confidence interval) for Overall Fracture

| Albiglutide |  |  |  |  |  |  |  |  |  |  |  |  |  |  |  |  |  |  |  |  |  |  |  |  |  |  |  |
| --- | --- | --- | --- | --- | --- | --- | --- | --- | --- | --- | --- | --- | --- | --- | --- | --- | --- | --- | --- | --- | --- | --- | --- | --- | --- | --- | --- |
| 0.12  (-0.52, 0.75) | Alogliptin |  |  |  |  |  |  |  |  |  |  |  |  |  |  |  |  |  |  |  |  |  |  |  |  |  |  |
| -0.79  (-3.00, 0.99) | -0.92  (-3.20, 0.94) | Bexagliflozin |  |  |  |  |  |  |  |  |  |  |  |  |  |  |  |  |  |  |  |  |  |  |  |  |  |
| -0.53  (-1.04, 0.00) | -0.64  (-1.22, -0.08) | 0.26  (-1.55, 2.48) | Canagliflozin |  |  |  |  |  |  |  |  |  |  |  |  |  |  |  |  |  |  |  |  |  |  |  |  |
| -0.05  (-0.52, 0.41) | -0.17  (-0.69, 0.34) | 0.74  (-1.08, 2.90) | 0.47  (0.10, 0.83) | Dapagliflozin |  |  |  |  |  |  |  |  |  |  |  |  |  |  |  |  |  |  |  |  |  |  |  |
| -0.07  (-0.54, 0.39) | -0.19  (-0.72, 0.33) | 0.72  (-1.10, 2.91) | 0.46  (0.07, 0.83) | -0.02  (-0.31,0.27) | Dulaglutide |  |  |  |  |  |  |  |  |  |  |  |  |  |  |  |  |  |  |  |  |  |  |
| 0.03  (-0.60, 0.67) | -0.09  (-0.77, 0.57) | 0.82  (-1.06, 3.06) | 0.56  (-0.02, 1.15) | 0.09  (-0.43, 0.59) | 0.11  (-0.43, 0.63) | Empagliflozin |  |  |  |  |  |  |  |  |  |  |  |  |  |  |  |  |  |  |  |  |  |
| -0.11  (-1.92, 1.76) | -0.23  (-2.02, 1.67) | 0.73  (-1.81, 3.46) | 0.42  (-1.36, 2.23) | -0.05  (-1.83, 1.76) | -0.03  (-1.82, 1.74) | -0.12  (-2.02, 1.74) | Enavogliflozin |  |  |  |  |  |  |  |  |  |  |  |  |  |  |  |  |  |  |  |  |
| -0.44  (-0.94, 0.10) | -0.56  (-1.14, 0.02) | 0.36  (-1.45, 2.54) | 0.09  (-0.36, 0.54) | -0.39  (-0.75, -0.01) | -0.36  (-0.75, 0.02) | -0.47  (-1.05, 0.14) | -0.33  (-2.13, 1.48) | Ertugliflozin |  |  |  |  |  |  |  |  |  |  |  |  |  |  |  |  |  |  |  |
| 1.05  (-1.44, 4.44) | 0.92  (-1.59, 4.25) | 1.92  (-1.29, 5.69) | 1.57  (-0.91, 4.94) | 1.08  (-1.39, 4.43) | 1.10  (-1.37, 4.44) | 1.01  (-1.49, 4.36) | 1.15  (-1.93, 4.90) | 1.48  (-1.02, 4.78) | Evogliptin |  |  |  |  |  |  |  |  |  |  |  |  |  |  |  |  |  |  |
| -0.40  (-1.09, 0.30) | -0.52  (-1.27, 0.22) | 0.39  (-1.51, 2.62) | 0.13  (-0.53, 0.76) | -0.34  (-0.96, 0.24) | -0.33  (-0.93, 0.27) | -0.44  (-1.16, 0.30) | -0.28  (-2.21, 1.60) | 0.04  (-0.61, 0.68) | -1.45  (-4.71, 1.11) | Exenatide |  |  |  |  |  |  |  |  |  |  |  |  |  |  |  |  |  |
| -0.05  (-0.70, 0.56) | -0.17  (-0.89, 0.54) | 0.73  (-1.14, 2.95) | 0.48  (-0.15, 1.08) | 0.00  (-0.56, 0.55) | 0.02  (-0.51, 0.54) | -0.08  (-0.78, 0.62) | 0.06  (-1.86, 1.89) | 0.38  (-0.23, 0.97) | -1.09  (-4.42, 1.40) | 0.34  (-0.26, 0.97) | Insulin |  |  |  |  |  |  |  |  |  |  |  |  |  |  |  |  |
| 0.75  (-1.78, 4.25) | 0.63  (-1.87, 4.23) | 1.61  (-1.49, 5.62) | 1.28  (-1.26, 4.88) | 0.79  (-1.67, 4.35) | 0.82  (-1.67, 4.37) | 0.72  (-1.81, 4.28) | 0.90  (-2.28, 4.68) | 1.19  (-1.33, 4.71) | -0.28  (-4.40, 4.16) | 1.17  (-1.40, 4.78) | 0.81  (-1.79, 4.42) | Ipragliflozin |  |  |  |  |  |  |  |  |  |  |  |  |  |  |  |
| 0.18  (-0.37, 0.73) | 0.06  (-0.51, 0.62) | 0.95  (-0.85, 3.16) | 0.71  (0.25, 1.17) | 0.23  (-0.17, 0.61) | 0.25  (-0.17, 0.66) | 0.15  (-0.42, 0.71) | 0.30  (-1.54, 2.09) | 0.61  (0.14, 1.09) | -0.87  (-4.15, 1.64) | 0.58  (-0.05, 1.24) | 0.23  (-0.37, 0.85) | -0.57  (-4.20, 1.96) | Linagliptin |  |  |  |  |  |  |  |  |  |  |  |  |  |  |
| 0.40  (-0.15, 0.95) | 0.28  (-0.33, 0.87) | 1.18  (-0.63, 3.41) | 0.92  (0.44, 1.41) | 0.44  (0.02, 0.86) | 0.47  (0.04, 0.88) | 0.36  (-0.23, 0.99) | 0.51  (-1.38, 2.33) | 0.82  (0.35, 1.32) | -0.64  (-3.99, 1.81) | 0.79  (0.16, 1.45) | 0.45  (-0.15, 1.04) | -0.37  (-3.87, 2.17) | 0.22  (-0.29, 0.72) | Liraglutide |  |  |  |  |  |  |  |  |  |  |  |  |  |
| 0.20  (-0.41, 0.82) | 0.08  (-0.60, 0.74) | 0.99  (-0.87, 3.18) | 0.72  (0.17, 1.26) | 0.25  (-0.24, 0.74) | 0.27  (-0.22, 0.74) | 0.17  (-0.51, 0.79) | 0.31  (-1.55, 2.12) | 0.63  (0.06, 1.17) | -0.83  (-4.18, 1.69) | 0.59  (-0.10, 1.32) | 0.24  (-0.41, 0.91) | -0.56  (-4.12, 1.93) | 0.01  (-0.54, 0.57) | -0.20  (-0.79, 0.36) | Lixisenatide |  |  |  |  |  |  |  |  |  |  |  |  |
| 0.08  (-0.51, 0.67) | -0.04  (-0.65, 0.56) | 0.86  (-0.98, 3.08) | 0.60  (0.09, 1.13) | 0.14  (-0.34, 0.56) | 0.16  (-0.34, 0.60) | 0.05  (-0.55, 0.64) | 0.19  (-1.65, 2.00) | 0.51  (-0.01, 1.02) | -0.97  (-4.26, 1.54) | 0.47  (-0.20, 1.18) | 0.13  (-0.54, 0.77) | -0.66  (-4.25, 1.85) | -0.10  (-0.54, 0.34) | -0.31  (-0.88, 0.22) | -0.11  (-0.72, 0.49) | Metformin |  |  |  |  |  |  |  |  |  |  |  |
| -0.09  (-0.81, 0.63) | -0.21  (-0.96, 0.53) | 0.69  (-1.18, 2.97) | 0.44  (-0.23, 1.12) | -0.04  (-0.66, 0.57) | -0.02  (-0.63, 0.61) | -0.13  (-0.87, 0.65) | 0.02  (-1.92, 1.89) | 0.35  (-0.32, 1.00) | -1.13  (-4.49, 1.43) | 0.31  (-0.51, 1.14) | -0.04  (-0.82, 0.75) | -0.86  (-4.42, 1.64) | -0.26  (-0.94, 0.42) | -0.48  (-1.17, 0.20) | -0.29  (-1.02, 0.46) | -0.17  (-0.88, 0.55) | Omarigliptin |  |  |  |  |  |  |  |  |  |  |
| -0.13  (-0.55, 0.29) | -0.25  (-0.74, 0.22) | 0.67  (-1.15, 2.83) | 0.39  (0.08, 0.70) | -0.08  (-0.28, 0.11) | -0.06  (-0.27, 0.16) | -0.17  (-0.64, 0.33) | -0.02  (-1.84, 1.77) | 0.31  (-0.01, 0.63) | -1.16  (-4.51, 1.27) | 0.27  (-0.29, 0.85) | -0.08  (-0.59, 0.45) | -0.87  (-4.46, 1.58) | -0.31  (-0.66, 0.06) | -0.53  (-0.90, -0.14) | -0.33  (-0.76, 0.13) | -0.21  (-0.61, 0.21) | -0.05  (-0.62, 0.56) | Placebo |  |  |  |  |  |  |  |  |  |
| -0.22  (-0.71, 0.27) | -0.34  (-0.89, 0.22) | 0.57  (-1.26, 2.76) | 0.31  (-0.11, 0.71) | -0.17  (-0.50, 0.16) | -0.14  (-0.49, 0.19) | -0.25  (-0.78, 0.31) | -0.11  (-1.93, 1.69) | 0.21  (-0.20, 0.63) | -1.24  (-4.60, 1.23) | 0.18  (-0.43, 0.81) | -0.16  (-0.73, 0.42) | -0.96  (-4.61, 1.52) | -0.40  (-0.82, 0.04) | -0.61  (-1.07, -0.16) | -0.41  (-0.94, 0.11) | -0.29  (-0.76, 0.18) | -0.12  (-0.77, 0.52) | -0.08  (-0.35, 0.17) | Saxagliptin |  |  |  |  |  |  |  |  |
| -0.02  (-0.66, 0.64) | -0.14  (-0.81, 0.54) | 0.77  (-1.12, 3.06) | 0.51  (-0.06, 1.08) | 0.03  (-0.49, 0.59) | 0.05  (-0.46, 0.62) | -0.05  (-0.69, 0.64) | 0.09  (-1.76, 1.96) | 0.42  (-0.16, 1.00) | -1.04  (-4.38, 1.40) | 0.38  (-0.34, 1.12) | 0.04  (-0.66, 0.75) | -0.78  (-4.30, 1.78) | -0.19  (-0.79, 0.40) | -0.42  (-1.03, 0.20) | -0.21  (-0.88, 0.47) | -0.10  (-0.72, 0.53) | 0.07  (-0.67, 0.84) | 0.11  (-0.37, 0.62) | 0.20  (-0.36, 0.77) | Semaglutide |  |  |  |  |  |  |  |
| 0.11  (-0.41, 0.65) | -0.01  (-0.60, 0.58) | 0.90  (-0.88, 3.09) | 0.63  (0.17, 1.10) | 0.16  (-0.24, 0.56) | 0.18  (-0.25, 0.58) | 0.08  (-0.50, 0.67) | 0.22  (-1.63, 2.03) | 0.54  (0.07, 1.04) | -0.93  (-4.25, 1.54) | 0.51  (-0.16, 1.19) | 0.16  (-0.46, 0.79) | -0.64  (-4.18, 1.86) | -0.07  (-0.50, 0.40) | -0.28  (-0.80, 0.22) | -0.08  (-0.66, 0.49) | 0.03  (-0.46, 0.54) | 0.20  (-0.49, 0.88) | 0.24  (-0.12, 0.60) | 0.32  (-0.11, 0.77) | 0.13  (-0.46, 0.71) | Sitagliptin |  |  |  |  |  |  |
| -0.13  (-0.63, 0.36) | -0.26  (-0.81, 0.29) | 0.65  (-1.15, 2.85) | 0.39  (-0.02, 0.80) | -0.08  (-0.41, 0.24) | -0.06  (-0.40, 0.27) | -0.17  (-0.70, 0.38) | -0.03  (-1.85, 1.77) | 0.30  (-0.11, 0.72) | -1.18  (-4.53, 1.28) | 0.26  (-0.36, 0.91) | -0.08  (-0.66, 0.50) | -0.90  (-4.45, 1.59) | -0.32  (-0.77, 0.12) | -0.53  (-0.99, -0.07) | -0.33  (-0.84, 0.19) | -0.22  (-0.70, 0.28) | -0.05  (-0.69, 0.61) | -0.01  (-0.27, 0.27) | 0.08  (-0.29, 0.45) | -0.12  (-0.68, 0.46) | -0.25  (-0.70, 0.19) | Sotagliflozin |  |  |  |  |  |
| 0.10  (-0.39, 0.60) | -0.01  (-0.54, 0.52) | 0.89  (-0.93, 3.06) | 0.63  (0.21, 1.06) | 0.16  (-0.18, 0.50) | 0.18  (-0.18, 0.53) | 0.07  (-0.44, 0.60) | 0.22  (-1.64, 2.00) | 0.54  (0.11, 0.97) | -0.93  (-4.26, 1.53) | 0.50  (-0.09, 1.12) | 0.15  (-0.41, 0.72) | -0.65  (-4.27, 1.87) | -0.08  (-0.34, 0.20) | -0.29  (-0.75, 0.17) | -0.09  (-0.62, 0.46) | 0.03  (-0.37, 0.43) | 0.18  (-0.46, 0.85) | 0.24  (-0.06, 0.53) | 0.32  (-0.06, 0.71) | 0.12  (-0.44, 0.68) | -0.01  (-0.41, 0.40) | 0.24  (-0.17, 0.64) | Sulfonylurea |  |  |  |  |
| -0.45  (-1.00, 0.09) | -0.57  (-1.14, -0.01) | 0.34  (-1.48, 2.51) | 0.07  (-0.38, 0.55) | -0.39  (-0.81, -0.02) | -0.38  (-0.79, 0.02) | -0.48  (-1.05, 0.07) | -0.35  (-2.19, 1.46) | -0.02  (-0.49, 0.45) | -1.50  (-4.82, 1.02) | -0.06  (-0.68, 0.60) | -0.40  (-1.00, 0.18) | -1.20  (-4.79, 1.32) | -0.64  (-1.01, -0.27) | -0.84  (-1.37, 0.35) | -0.65  (-1.22, -0.09) | -0.53  (-0.88, -0.18) | -0.37  (-1.05, 0.33) | -0.32  (-0.69, 0.03) | -0.23  (-0.67, 0.18) | -0.44  (-1.04, 0.15) | -0.56  (-1.01, -0.13) | -0.32  (-0.76, 0.13) | -0.56  (-0.84, -0.27) | Thiazolidinedione |  |  |  |
| 0.50  (-1.68, 4.03) | 0.38  (-1.83, 3.81) | 1.36  (-1.15, 5.31) | 1.01  (-1.10, 4.40) | 0.56  (-1.62, 3.95) | 0.58  (-1.56, 4.03) | 0.46  (-1.70, 3.96) | 0.63  (-2.22, 4.41) | 0.93  (-1.23, 4.31) | -0.50  (-4.41, 3.59) | 0.90  (-1.31, 4.35) | 0.55  (-1.65, 4.03) | -0.26  (-4.40, 3.97) | 0.33  (-1.86, 3.75) | 0.10  (-2.05, 3.55) | 0.29  (-1.86, 3.73) | 0.42  (-1.74, 3.88) | 0.60  (-1.63, 4.05) | 0.62  (-1.51, 4.04) | 0.71  (-1.47, 4.17) | 0.51  (-1.70, 3.98) | 0.38  (-1.78, 3.89) | 0.63  (-1.52, 4.02) | 0.40  (-1.78, 3.82) | 0.94  (-1.20, 4.43) | Tirzepatide |  |  |
| -1.35  (-4.74, 0.72) | -1.45  (-4.99, 0.59) | -0.61  (-4.24, 2.42) | -0.81  (-4.27, 1.18) | -1.29  (-4.67, 0.71) | -1.26  (-4.68, 0.73) | -1.37  (-4.82, 0.65) | -1.29  (-5.19, 1.49) | -0.92  (-4.28, 1.11) | -2.47  (-6.97, 0.89) | -0.94  (-4.40, 1.18) | -1.29  (-4.80, 0.78) | -2.24  (-6.95, 1.11) | -1.51  (-5.00, 0.51) | -1.72  (-5.15, 0.29) | -1.54  (-4.88, 0.53) | -1.43  (-4.86, 0.68) | -1.26  (-4.73, 0.83) | -1.20  (-4.63, 0.76) | -1.12  (-4.54, 0.86) | -1.32  (-4.70, 0.75) | -1.47  (-4.86, 0.60) | -1.18  (-4.64, 0.78) | -1.44  (-4.90, 0.57) | -0.88  (-4.33, 1.16) | -1.94  (-6.78, 1.09) | Tofogliflozin |  |
| 0.55  (-0.34, 1.47) | 0.43  (-0.50, 1.38) | 1.35  (-0.60, 3.67) | 1.08  (0.26, 1.97) | 0.61  (-0.20, 1.45) | 0.62  (-0.19, 1.48) | 0.53  (-0.40, 1.50) | 0.66  (-1.32, 2.60) | 0.99  (0.13, 1.88) | -0.50  (-3.90, 2.14) | 0.96  (-0.03, 1.95) | 0.60  (-0.33, 1.57) | -0.21  (-3.91, 2.50) | 0.38  (-0.48, 1.25) | 0.15  (-0.70, 1.07) | 0.35  (-0.52, 1.28) | 0.48  (-0.39, 1.38) | 0.65  (-0.31, 1.64) | 0.68  (-0.10, 1.52) | 0.77  (-0.05, 1.62) | 0.57  (-0.35, 1.51) | 0.45  (-0.40, 1.35) | 0.68  (-0.16, 1.56) | 0.46  (-0.39, 1.33) | 1.01  (0.18, 1.89) | 0.07  (-3.43, 2.33) | 1.90  (-0.26, 5.29) | Vildagliptin |

The columns present the row drug class compared to the column drug class. The rows present the row drug class compared to the column drug class. The effect estimates are expressed as odds ratio and 95% confidence intervals. For example, the odds ratio for overall fracture with albiglutide compared to alogliptin is 0.12 (95% confidence interval -0.52 to 0.75)

Table S10. Network estimated effect sizes (95% confidence interval) for Overall Fracture_Treatment Type

| DPP-4 |  |  |  |  |  |  |  |
| --- | --- | --- | --- | --- | --- | --- | --- |
| 0.11 (-0.14, 0.37) | GLP-1 |  |  |  |  |  |  |
| -0.29 (-0.84, 0.24) | -0.40 (-0.90, 0.08) | Insulin |  |  |  |  |  |
| -0.01 (-0.38, 0.35) | -0.12 (-0.53, 0.28) | 0.28 (-0.32, 0.91) | Metformin |  |  |  |  |
| -0.46 (-0.65, -0.28) | -0.58 (-0.75, -0.41) | -0.17 (-0.68, 0.34) | -0.45 (-0.82, -0.08) | Placebo |  |  |  |
| -0.34 (-0.55, -0.13) | -0.45 (-0.67, -0.24) | -0.05 (-0.56, 0.48) | -0.32 (-0.71, 0.06) | 0.13 (0.00, 0.25) | SGLT-2 |  |  |
| -0.08 (-0.29, 0.13) | -0.19 (-0.50, 0.10) | 0.21 (-0.35, 0.78) | -0.07 (-0.45, 0.32) | 0.38 (0.13, 0.64) | 0.25 (0.00, 0.53) | Sulfonylurea |  |
| -0.46 (-0.77, -0.16) | -0.58 (-0.95, -0.23) | -0.17 (-0.77, 0.41) | -0.46 (-0.79, -0.11) | 0.00 (-0.33, 0.32) | -0.13 (-0.46, 0.21) | -0.39 (-0.67, -0.09) | Thiazolidinedione |

The columns present the row drug class compared to the column drug class. The rows present the row drug class compared to the column drug class. The effect estimates are expressed as odds ratio and 95% confidence intervals. For example, the odds ratio for overall fracture with DPP4 inhibitor compared to GLP1 receptor agonists is 0.11 (95% confidence interval -0.14 to 0.37)

Table S11. Network estimated effect sizes (95% confidence interval) for Vertebral Fracture

| Albiglutide |  |  |  |  |  |  |  |  |  |  |  |  |  |  |  |  |  |  |  |  |  |  |  |
| --- | --- | --- | --- | --- | --- | --- | --- | --- | --- | --- | --- | --- | --- | --- | --- | --- | --- | --- | --- | --- | --- | --- | --- |
| 0.14  (-2.73, 3.48) | Alogliptin |  |  |  |  |  |  |  |  |  |  |  |  |  |  |  |  |  |  |  |  |  |  |
| -0.88  (-2.39, 0.50) | -1.02  (-4.44, 1.77) | Canagliflozin |  |  |  |  |  |  |  |  |  |  |  |  |  |  |  |  |  |  |  |  |  |
| -0.37  (-1.84, 0.93) | -0.52  (-3.86, 2.10 | 0.51  (-0.57, 1.58) | Dapagliflozin |  |  |  |  |  |  |  |  |  |  |  |  |  |  |  |  |  |  |  |  |
| -0.70  (-2.10, 0.61) | -0.84  (-4.18, 1.92) | 0.18  (-0.92, 1.24) | -0.32  (-1.27, 0.64) | Dulaglutide |  |  |  |  |  |  |  |  |  |  |  |  |  |  |  |  |  |  |  |
| -0.66  (-2.53, 1.13) | -0.82  (-4.36, 2.11) | 0.21  (-1.38, 1.84 | -0.30  (-1.74, 1.27) | 0.03  (-1.52, 1.62) | Empagliflozin |  |  |  |  |  |  |  |  |  |  |  |  |  |  |  |  |  |  |
| -0.83  (-2.31, 0.53) | -0.96  (-4.33, 1.76) | 0.06  (-1.09, 1.16) | -0.45  (-1.47, 0.56) | -0.12  (-1.19, 0.90) | -0.15  (-1.77, 1.37) | Ertugliflozin |  |  |  |  |  |  |  |  |  |  |  |  |  |  |  |  |  |
| 0.17  (-2.83, 3.56) | 0.03  (-4.10, 4.28) | 1.06  (-1.83, 4.42) | 0.55  (-2.28, 3.88) | 0.87  (-1.94, 4.16) | 0.85  (-2.30, 4.38) | 0.99  (-1.89, 4.40) | Evogliptin |  |  |  |  |  |  |  |  |  |  |  |  |  |  |  |  |
| -0.65  (-4.69, 3.07) | -0.83  (-5.72, 3.65) | 0.25  (-3.74, 3.98) | -0.28  (-4.18, 3.39) | 0.06  (-3.77, 3.69) | 0.01  (-4.07, 3.82) | 0.16  (-3.68, 3.93) | -0.85  (-5.90, 3.83) | Exenatide |  |  |  |  |  |  |  |  |  |  |  |  |  |  |  |
| -0.69  (-2.87, 1.60) | -0.82  (-4.59, 2.36) | 0.19  (-1.79, 2.28) | -0.32  (-2.26, 1.72) | 0.02  (-1.79. 1.86) | -0.01  (-2.32, 2.33) | 0.14  (-1.85, 2.24) | -0.88  (-4.64, 2.46) | -0.052  (-4.02, 4.19) | Insulin |  |  |  |  |  |  |  |  |  |  |  |  |  |  |
| -0.58  (-2.07, 0.85) | -0.71  (-4.08, 2.03) | 0.30  (-0.92, 1.56) | -0.20  (-1.31, 0.94) | 0.13  (-0.99, 1.27) | 0.10  (-1.58, 1.71) | 0.24  (-0.93, 1.51) | -0.72  (-4.26, 2.17) | 0.08  (-3.66, 4.09) | 0.11  (-2.03, 2.24) | Linagliptin |  |  |  |  |  |  |  |  |  |  |  |  |  |
| -0.55  (-2.36, 1.21) | -0.69  (-4.11, 2.19) | 0.33  (-1.17, 1.88) | -0.17  (-1.64, 1.29) | 0.14  (-1.25, 1.55) | 0.12  (-1.79, 1.98) | 0.27  (-1.21, 1.82) | -0.73  (-4.33, 2.29) | 0.09  (-3.34, 3.70) | 0.14  (-1.97, 2.07) | 0.03  (-1.55, 1.61) | Liraglutide |  |  |  |  |  |  |  |  |  |  |  |  |
| -0.49  (-2.36, 1.27) | -0.61  (-3.96, 2.30) | 0.40  (-1.28, 2.02) | -0.08  (-1.69, 1.43) | 0.24  (-1.34, 1.75) | 0.20  (-1.76, 2.11) | 037  (-1.24, 1.88) | -0.64  (-4.39, 2.49) | 0.18  (-3.67, 4.31) | 0.21  (-2.15, 2.52) | 0.14  (-1.62, 1.71) | 0.08  (-1.89, 1.92) | Lixisenatide |  |  |  |  |  |  |  |  |  |  |  |
| -1.29  (-3.08, 0.33) | -1.45  (-4.96, 1.53) | -0.41  (-1.91, 1.08) | -0.91  (-2.36, 0.47) | -0.59  (-2.04, 0.85) | -0.61  (-2.54, 1.16) | -0.46  (-1.91, 0.98) | -1.48  (-4.95, 1.50) | -0.65  (-4.49, 3.44) | -0.62  (-2.96, 1.62) | -0.72  (-2.06, 0.64) | -0.73  (-2.59, 1.10) | -0.84  (2.69, 1.03) | Metformin |  |  |  |  |  |  |  |  |  |  |
| -1.69  (-5.29, 0.99) | -1.94  (-6.45, 1.89) | -0.82  (-4.47, 1.79) | -1.31  (-4.84, 1.25) | -0.99  (-4.51, 1.57) | -1.03  (-4.75, 1.77) | -0.87  (-4.40, 1.73) | -1.93  (-6.68, 1.79) | -1.15  (-6.00, 3.58) | -1.03  (-4.74, 2.06) | -1.12  (-4.61, 1.53) | -1.15  (-4.78, 1.59) | -1.24  (-4.79,1.66) | -0.41  (-4.07, 2.32) | Omarigliptin |  |  |  |  |  |  |  |  |  |
| -0.78  (-2.02, 0.32) | -0.92  (-4.23, 1.71) | 0.09  (-0.73, 0.93) | -0.41  (-1.05, 0.25) | -0.08  (-0.77, 0.60) | -0.11  (-1.54, 1.22) | 0.04  (-0.70, 0.85) | -0.94  (-4.21, 1.82) | -0.12  (-3.74, 3.66) | -0.08  (-1.94, 1.76) | -0.20  (-1.16, 0.72) | -0.23  (-1.55, 1.04) | -0.33  (-1.64, 1.11) | .515  (-0.72, 1.82) | 0.89  (-1.53, 4.38) | Placebo |  |  |  |  |  |  |  |  |
| -0.44  (-2.03, 1.09) | -0.58  (-3.90, 2.24) | 0.45  (-0.83, 1.76) | -0.07  (-1.23, 1.15) | 0.27  (-0.93, 1.47) | 0.24  (-1.44, 1.97) | 0.39  (-0.80, 1.70) | -0.59  (-4.11, 2.31) | 0.23  (-3.56, 4.14) | 0.26  (-1.91, 2.38) | 0.15  (-1.10, 1.40) | 0.10  (-1.51, 1.73) | 0.03  (-1.62, 1.77) | 0.86  (-0.65, 2.44) | 1.25  (-1.42, 4.81) | 0.35  (-0.62, 1.37) | Saxagliptin |  |  |  |  |  |  |  |
| 0.29  (-1.52, 2.15) | 0.15  (-3.42, 3.15) | 1.15  (-0.41, 2.90) | 0.66  (-0.78, 2.34) | 0.98  (-0.48, 2.61) | 0.95  (-0.99, 2.96) | 1.10  (-0.40, 2.76) | 0.10  (-3.29, 3.28) | 0.95  (-2.94, 4.92) | 0.99  (-1.40, 3.27) | 0.85  (-0.78, 2.62) | 0.83  (-0.99, 2.86) | 0.75  (-1.13, 2.85) | 1.57  (-0.28, 3.58) | 2.02  (-0.86, 5.86) | 1.05  (-0.21, 2.60) | 0.72  (-0.97, 2.52) | Semaglutide |  |  |  |  |  |  |
| -0.73  (-2.20, 0.64) | -0.87  (-4.31, 1.89) | 0.14  (-0.93, 1.32) | -0.36  (-1.42, 0.67) | -0.04  (-1.06, 1.04) | -0.05  (-1.70, 1.52) | 0.09  (-1.00, 1.24) | -0.89  (-4.16, 1.73) | -0.11  (-3.82, 3.89) | -0.05  (-2.10, 1.96) | -0.15  (-1.38, 1.04) | -0.19  (-1.75, 1.32) | -0.27  (-1.83, 1.37) | 0.54  (-0.78, 1.95) | 0.95  (-1.61, 4.52) | 0.05  (-0.78, 0.87) | -0.29  (-1.63, 0.98) | -1.00  (-2.74, 0.56) | Sitagliptin |  |  |  |  |  |
| -0.63  (-2.26, 0.93) | -0.73  (-4.25, 2.11) | 0.26  (-1.13, 1.65) | -0.24  (-1.52, 1.05) | 0.09  (-1.20, 1.41) | 0.06  (-1.79, 1.78) | 0.21  (-1.15, 1.61) | -0.76  (-4.22, 2.22) | -0.01  (-3.78, 4.04) | 0.10  (-2.13, 2.15) | -0.03  (-1.50, 1.42) | -0.06  (-1.77, 1.66) | -0.16  (-1.92, 1.72) | 0.66  (-0.99, 2.46) | 1.08  (-1.58, 4.68) | 0.16  (-0.96, 1.32) | -0.18  (-1.69, 1.32) | -0.90  (-2.76, 0.83) | 0.12  (-1.29, 1.52) | Sotagliflozin |  |  |  |  |
| -0.26  (-1.87, 1.30) | -0.41  (-3.84, 2.44) | 0.61  (-0.72, 2.01) | 0.11  (-1.10, 1.43) | 0.44  (-0.83, 1.69) | 0.41  (-1.34, 2.15) | 0.56  (-0.74, 1.95) | -0.42  (-4.04, 2.55) | 0.40  (-3.31, 4.33) | 0.42  (-1.68, 2.55) | 0.31  (-0.45, 1.08) | 0.28  (-1.33, 1.99) | 0.21  (-1.51, 2.00) | 1.03  (-0.45, 2.52) | 1.42  (-1.29, 4.96) | 0.52  (-0.53, 1.63) | 0.17  (-1.10, 1.49) | -0.55  (-2.42, 1.19) | 0.46  (-0.84, 1.82) | 0.36  (-1.20, 1.89) | Sulfonylurea |  |  |  |
| -0.22  (-2.13, 1.66) | -0.37  (-3.89, 2.64) | 0.67  (-1.03, 2.43) | 0.20  (-1.46, 1.81) | 0.50  (-1.11, 2.16) | 0.46  (-1.58, 2.52) | 0.63  (-1.00, 2.34) | -0.35  (-4.03, 2.74) | 0.46  (-3.47, 4.63) | 0.49  (-1.86, 2.85) | 0.38  (-1.00, 1.77) | 0.36  (-1.67, 2.30) | 0.28  (-1.71, 2.33) | 1.11  (-0.71, 2.91) | 1.52  (-1.43, 5.13) | 0.60  (-0.91, 2.10) | 0.24  (-1.43, 1.86) | -0.48  (-2.65, 1.50) | 0.54  (-1.13, 2.22) | 0.43  (-1.45, 2.24) | 0.07  (-1.16, 1.29) | Thiazolidinedione |  |  |
| -0.94  (-3.89, 2.48) | -1.10  (-5.20, 2.96) | -0.06  (-2.80, 3.34) | -0.57  (-3.28, 2.79) | -0.24  (-2.93, 3.22) | -0.27  (-3.33, 3.40) | -0.12  (-2.80, 3.36) | -1.08  (-5.43, 3.05) | -0.24  (-4.79, 4.73) | -0.25  (-3.54, 3.48) | -0.34  (-3.17, 2.97) | -0.38  (-3.32, 3.13) | -0.45  (-3.48, 3.18) | 0.35  (-2.56, 3.95) | 0.80  (-2.97, 5.33) | -0.18  (-2.78, 3.17) | -0.53  (-3.32, 2.94) | -1.22  (-4.29, 2.37) | -0.20  (-3.01, 3.19) | -0.33  (-3.23, 3.20) | -0.68  (-3.54, 2.75) | -0.74  (-3.71, 2.89) | Tirzepatide |  |
| -0.80  (-2.83, 1.32) | -0.90  (-4.51, 2.18) | 0.07  (-1.80, 2.08) | -0.41  (-2.26, 1.50) | -0.09  (-1.97, 1.81) | -0.10  (-2.33, 2.10) | 0.06  (-1.83, 2.02) | -0.94  (-4.74, 2.24) | -0.14  (-4.12, 4.28) | -0.10  (-2.65, 2.41) | -0.19  (-2.19, 1.78) | -0.24  (-2.43. 1.92) | -0.31  (-2.54, 1.89) | 0.52  (-1.67, 2.72) | 0.95  (-2.13, 4.71) | -0.01  (-1.78, 1.83) | -0.36  (-2.32, 1.65) | -1.08  (-3.40, 1.19) | -0.04  (-1.95, 1.91) | -0.17  (-2.19, 1.93) | -0.50  (-2.61, 1.52) | -0.57  (-2.89, 1.66) | 0.15  (-3.67, 3.36) | Vildagliptin |

The columns present the row anti-diabetic agents compared to the column anti-diabetic agents. The rows present the row drug class compared to the column individual anti-diabetic agents. The effect estimates are expressed as odds ratio and 95% confidence intervals. For example, the odd ratio for vertebral fracture with Albiglutide compared to Alogliptin is 0.14 (95% confidence interval -2.73 to 3.48).

Table S12. Network estimated effect sizes (95% confidence interval) for Vertebral Fracture_Treatment Type

| DPP4i |  |  |  |  |  |  |  |
| --- | --- | --- | --- | --- | --- | --- | --- |
| 0.25 (-0.39, 0.90) | GLP1RA |  |  |  |  |  |  |
| -1.49 (-3.16, -0.20) | -1.74 (-3.31, -0.58) | Insulin |  |  |  |  |  |
| -0.62 (-1.84, 0.57) | -0.88 (-2.20, 0.46) | 0.89 (-0.95, 2.91) | Metformin |  |  |  |  |
| -0.09 (-0.58, 0.37) | -0.35 (-0.81, 0.12) | 1.41 (0.17, 2.98) | 0.54 (-0.69, 1.76) | Placebo |  |  |  |
| 0.06 (-0.54, 0.65) | -0.20 (-0.82, 0.40) | 1.55 (0.25, 3.18) | 0.69 (-0.58, 1.95) | 0.15 (-0.23, 0.51) | SGLT2i |  |  |
| -0.85 (-1.45, -0.33) | -1.12 (-1.98, -0.28) | 0.63 (-0.77, 2.35) | -0.24 (-1.58, 1.11) | -0.77 (-1.49, -0.07) | -0.92 (-1.74, -0.12) | Sulfonylurea |  |
| -0.94 (-1.68, -0.19) | -1.20 (-2.17, -0.24) | 0.56 (-0.92, 2.30) | -0.33 (-1.74, 1.06) | -0.85 (-1.70, -0.03) | -1.00 (-1.94, -0.09) | -0.08 (-0.62, 0.47) | Thiazolidinedione |

The columns present the row treatment type compared to the column treatment type. The rows present the row drug class compared to the column treatment type. The effect estimates are expressed as odds ratio and 95% confidence intervals. For example, the odd ratio for vertebral fracture with DPP4i compared to GLP1RA is 0.25 (95% confidence interval -0.39 to 0.90).

Table S13. Network estimated effect sizes (95% confidence interval) for Hip Fracture

| Albiglutide |  |  |  |  |  |  |  |  |  |  |  |  |  |  |  |  |  |  |  |  |  |
| --- | --- | --- | --- | --- | --- | --- | --- | --- | --- | --- | --- | --- | --- | --- | --- | --- | --- | --- | --- | --- | --- |
| -0.85  (-2.41, 0.62) | Alogliptin |  |  |  |  |  |  |  |  |  |  |  |  |  |  |  |  |  |  |  |  |
| -0.05  (-0.84, 0.77) | 0.80  (-0.51, 2.23) | Canagliflozin |  |  |  |  |  |  |  |  |  |  |  |  |  |  |  |  |  |  |  |
| 0.02  (-0.77, 0.84) | 0.87  (-0.46, 2.31) | 0.06  (-0.55, 0.67) | Dapagliflozin |  |  |  |  |  |  |  |  |  |  |  |  |  |  |  |  |  |  |
| -0.18  (-0.98, 0.64) | 0.67  (-0.65, 2.16) | -0.13  (-0.74, 0.52) | -0.20  (-0.77, 0.40) | Dulaglutide |  |  |  |  |  |  |  |  |  |  |  |  |  |  |  |  |  |
| 0.92  (0.06, 1.82) | 1.77  (0.44, 3.27) | 0.97  (0.28, 1.67) | 0.90  (0.25, 1.58) | 1.10  (0.41, 1.77) | Empagliflozin |  |  |  |  |  |  |  |  |  |  |  |  |  |  |  |  |
| 0.46  (-0.37, 1.32) | 1.30  (-0.05, 2.85) | 0.51  (-0.16, 1.22) | 0.45  (-0.18, 1.10) | 0.64  (-0.02, 1.31) | -0.45  (-1.21, 0.27) | Ertugliflozin |  |  |  |  |  |  |  |  |  |  |  |  |  |  |  |
| 0.91  (-0.25, 2.28) | 1.77  (0.12, 3.63) | 0.96  (-0.19, 2.28) | 0.91  (-0.23, 2.20) | 1.10  (-0.09, 2.43) | -0.01  (-1.18, 1.35) | 0.45  (-0.74, 1.82) | Exenatide |  |  |  |  |  |  |  |  |  |  |  |  |  |  |
| -1.43  (-2.37, -0.49) | -0.57  (-2.17, 1.13) | -1.38  (-2.50, -0.29) | -1.44  (-2.56, 0.37) | -1.25  (-2.36, -0.20) | -2.34  (-3.52, -1.28) | -1.88  (-3.01, -0.78) | -2.34  (-3.73, -1.19) | Insulin |  |  |  |  |  |  |  |  |  |  |  |  |  |
| -0.39  (-1.14, 0.40) | 0.47  (-0.82, 1.89) | -0.33  (-0.97, 0.28) | -0.39  (-0.99, 0.18) | -0.21  (-0.82, 0.40) | -1.30  (-1.99, -0.64) | -0.84  (-1.52, -0.19) | -1.31  (-2.55, -0.20) | 1.05  (0.01, 2.10) | Linagliptin |  |  |  |  |  |  |  |  |  |  |  |  |
| 0.91  (0.06, 1.77) | 1.76  (0.43, 3.29) | 0.96  (0.23, 1.69) | 0.90  (0.18, 1.60) | 1.07  (0.35, 1.84) | -0.02  (-0.81, 0.78) | 0.44  (-0.34, 1.24) | -0.02  (-1.33, 1.14) | 2.33  (1.34, 3.42) | 1.28  (0.61, 1.98) | Liraglutide |  |  |  |  |  |  |  |  |  |  |  |
| 0.74  (-0.24, 1.81) | 1.59  (0.16, 3.17) | 0.77  (-0.07, 1.69) | 0.73  (-0.10, 1.61) | 0.91  (0.06, 1.82) | -0.19  (-1.08, 0.79) | 0.28  (-0.57, 1.21) | -0.18  (-1.55, 1.17) | 2.18  (0.94, 3.49) | 1.12  (0.30, 2.03) | -0.16  (-1.11, 0.83) | Lixisenatide |  |  |  |  |  |  |  |  |  |  |
| -0.80  (-1.84, 0.25) | 0.05  (-1.42, 1.68) | -0.75  (-1.65, 0.16) | -0.81  (-1.71, 0.08) | -0.62  (-1.57, 0.29) | -1.72  (-2.61, -0.85) | -1.25  (-2.17, -0.34) | -1.71  (-3.16, -0.44) | 0.62  (-0.58, 1.90) | -0.42  (-1.26, 0.43) | -1.70  (-2.68, -0.72) | -1.53  (-2.69, -0.46) | Metformin |  |  |  |  |  |  |  |  |  |
| -1.05  (-1.97, -0.13) | -0.20  (-1.60, 1.33) | -1.01  (-1.78, -0.25) | -1.06  (-1.80, -0.33) | -0.87  (-1.63, -0.12) | -1.97  (-2.80, -1.18) | -1.52  (-2.33, -0.71) | -1.96  (-3.39, -0.75) | 0.38  (-0.78, 1.54) | -0.66  (-1.45, 0.08) | -1.95  (-2.83, -1.12) | -1.79  (-2.81, -0.83) | -0.25  (-1.24, 0.74) | Omarigliptin |  |  |  |  |  |  |  |  |
| -0.30  (-0.97, 0.40) | 0.55  (-0.68, 1.96) | -0.25  (-0.70, 0.22) | 0.31  (-0.72, 0.07) | -0.12  (-0.56, 0.31) | -1.21  (-1.76, -0.70) | -0.76  (-1.28, -0.27) | -1.22  (-2.46, -0.14) | 1.13  (0.17, 2.13) | 0.08  (-0.35, 0.53) | -1.20  (-1.82, -0.63) | -1.03  (-1.83, -0.33) | 0.49  (-0.31, 1.31) | 0.75  (0.14, 1.39) | Placebo |  |  |  |  |  |  |  |
| 0.00  (-1.05, 1.09) | 0.86  (-0.62, 2.47) | 0.05  (-0.91, 1.02) | -0.02  (-0.93, 0.88) | 0.17  (-0.77, 1.11) | -0.92  (-1.91, 0.06) | -0.46  (-1.47, 0.52) | -0.93  (-2.31, 0.38) | 1.43  (0.19, 2.71) | 0.38  (-0.50, 1.30) | -0.91  (-1.91, 0.10) | -0.76  (-1.89, 0.39) | 0.80  (-0.35, 1.93) | 1.05  (0.04, 2.11) | 0.29  (-0.53, 1.13) | Saxagliptin |  |  |  |  |  |  |
| -0.18  (-1.10, 0.82) | 0.67  (-0.78, 2.29) | -0.12  (-0.92, 0.69) | -0.18  (-0.99, 0.62) | 0.01  (-0.83, 0.85) | -1.08  (-1.93, -0.24) | -0.63  (-1.49, 0.21) | -1.10  (-2.49, 0.17) | 1.25  (0.09, 2.54) | 0.21  (-0.57, 1.04) | -1.08  (-1.96, -0.16) | -0.91  (-1.95, 0.08) | 0.62  (-0.41, 1.69) | 0.89  (-0.06, 1.81) | 0.13  (-0.56, 0.83) | -0.17  (-1.26, 0.90) | Semaglutide |  |  |  |  |  |
| 0.33  (-0.43, 1.11) | 1.18  (-0.11, 2.61) | 0.38  (-0.21, 0.97) | 0.32  (-0.26, 0.88) | 0.51  (-0.11, 1.10) | -0.59  (-1.26, 0.05) | -0.14  (-0.81, 0.52) | -0.59  (-1.85, 0.53) | 1.76  (0.71, 2.83) | 0.72  (0.13, 1.27) | -0.58  (-1.28, 0.10) | -0.41  (-1.36, 0.41) | 1.12  (0.29, 2.00) | 1.38  (0.62, 2.10) | 0.63  (0.21, 1.04) | 0.33  (-0.59, 1.25) | 0.51  (-0.31, 1.24) | Sitagliptin |  |  |  |  |
| -0.26  (-1.16, 0.69) | 0.59  (-0.78, 2.14) | -0.20  (-0.98, 0.58) | -0.27  (-1.01, 0.49) | -0.08  (-0.82, 0.69) | -1.16  (-1.99, -0.38) | -0.72  (-1.49, 0.08) | -1.16  (-2.58, 0.08) | 1.18  (0.02, 2.33) | 0.13  (-0.61, 0.91) | -1.16  (-2.03, -0.30) | -1.00  (-1.99, -0.01) | 0.54  (-0.46, 1.57) | 0.80  (-0.06, 1.69) | 0.04  (-0.58, 0.68) | -0.26  (-1.27, 0.80) | -0.08  (-1.03, 0.86) | -0.58  (-1.31, 0.15) | Sotagliflozin |  |  |  |
| -0.19  (-0.94, 0.58) | 0.66  (-0.56, 2.08) | -0.14  (-0.79, 0.50) | -0.20  (-0.82, 0.40) | -0.01  (-0.66, 0.63) | -1.10  (-1.83, -0.41) | -0.65  (-1.34, 0.03) | -1.11  (-2.32, -0.05) | 1.25  (0.24, 2.28) | 0.19  (-0.27, 0.68) | -1.09  (-1.77, -0.45) | -0.92  (-1.85, -0.05) | 0.61  (-0.26, 1.49) | 0.87  (0.09, 1.66) | 0.11  (-0.37, 0.59) | -0.18  (-1.02, 0.64) | -0.01  (-0.86, 0.83) | -0.52  (-1.08, 0.06) | 0.07  (-0.71, 0.82) | Sulfonylurea |  |  |
| 0.04  (-1.25, 1.34) | 0.87  (-0.82, 2.63) | 0.08  (-1.12, 1.29) | 0.03  (-1.18, 1.21) | 0.22  (-1.00, 1.42) | -0.88  (-2.16, 0.35) | -0.43  (-1.67, 0.80) | -0.88  (-2.50, 0.62) | 1.44  (0.01, 2.97) | 0.41  (-0.81, 1.61) | -0.87  (-2.12, 0.41) | -0.70 (-2.10, 0.62) | 0.83  (-0.53, 2.21) | 1.08  (-0.23, 2.39) | 0.34  (-0.79, 1.48) | 0.03  (-1.37, 1.40) | 0.20  (-1.13, 1.52) | -0.29  (-1.47, 0.92) | 0.29  (-1.03, 1.56) | 0.22  (-0.95, 1.39) | Thiazolidinedione |  |
| 1.18  (-0.08, 2.62) | 2.05  (0.40, 3.91) | 1.24  (0.10, 2.64) | 1.18  (0.03, 2.56) | 1.35  (0.20, 2.74) | 0.27  (-0.94, 1.68) | 0.72  (-0.50, 2.18) | 0.27  (-1.38, 1.98) | 2.63  (1.13, 4.21) | 1.58  (0.42, 2.92) | 0.29  (-0.99, 1.70) | 0.44  (-0.92, 1.95) | 1.98  (0.77, 3.41) | 2.24  (1.02, 3.66) | 1.48  (0.43, 2.79) | 1.19  (-0.22, 2.72) | 1.35  (0.04, 2.81) | 0.85  (-0.28, 2.23) | 1.44  (0.21, 2.85) | 1.38  (0.22, 2.75) | 1.16  (-0.39, 2.87) | Vildagliptin |

The columns present the row anti-diabetic agents compared to the column anti-diabetic agents. The rows present the row drug class compared to the column individual anti-diabetic agents. The effect estimates are expressed as odds ratio and 95% confidence intervals. For example, the odd ratio for hip fracture with Albiglutide compared to Alogliptin is -0.85 (95% confidence interval -2.41 to 0.62).

Table S14. Network estimated effect sizes (95% confidence interval) for Hip Fracture_Treatment Type

| DPP4i |  |  |  |  |  |  |  |
| --- | --- | --- | --- | --- | --- | --- | --- |
| 0.25 (-0.09, 0.61) | GLP1RA |  |  |  |  |  |  |
| -0.22 (-1.01, 0.60) | -0.49 (-1.22, 0.26) | Insulin |  |  |  |  |  |
| -0.48 (-1.29, 0.34) | -0.74 (-1.60, 0.14) | -0.24 (-1.41, 0.89) | Metformin |  |  |  |  |
| -0.16 (-0.41, 0.09) | -0.42 (-0.69, -0.16) | 0.06 (-0.73, 0.85) | 0.31 (-0.51, 1.14) | Placebo |  |  |  |
| 0.18 (-0.14, 0.50) | -0.08 (-0.40, 0.25) | 0.41 (-0.42, 1.20) | 0.66 (-0.19, 1.49) | 0.34 (0.14, 0.55) | SGLT2i |  |  |
| -0.15 (-0.50, 0.20) | -0.41 (-0.83, 0.02) | 0.07 (-0.81, 0.91) | 0.33 (-0.54, 1.19) | 0.01 (-0.36, 0.39) | -0.33 (-0.75, 0.08) | Sulfonylurea |  |
| -0.26 (-0.69, 0.15) | -0.52 (-1.00, -0.06) | -0.05 (-0.94, 0.82) | 0.22 (-0.70, 1.10) | -0.10 (-0.55, 0.32) | -0.44 (-0.93, 0.02) | -0.12 (-0.38, 0.15) | Thiazolidinedione |

The columns present the row treatment type compared to the column treatment type. The rows present the row drug class compared to the column treatment type. The effect estimates are expressed as odds ratio and 95% confidence intervals. For example, the odd ratio for hip fracture with DPP4i compared to GLP1RA is 0.25 (95% confidence interval -0.09 to 0.61).

Table S15. Network estimated effect sizes (95% confidence interval) for Non-Vertebral Fracture

| Albiglutide |  |  |  |  |  |  |  |  |  |  |  |  |  |  |  |  |  |  |  |  |  |  |  |
| --- | --- | --- | --- | --- | --- | --- | --- | --- | --- | --- | --- | --- | --- | --- | --- | --- | --- | --- | --- | --- | --- | --- | --- |
| 0.20  (-0.46, 0.86) | Alogliptin |  |  |  |  |  |  |  |  |  |  |  |  |  |  |  |  |  |  |  |  |  |  |
| -0.08  (-0.56, 0.38) | -0.29  (-0.93, 0.33) | Canagliflozin |  |  |  |  |  |  |  |  |  |  |  |  |  |  |  |  |  |  |  |  |  |
| 0.38  (-0.08, 0.82) | 0.17  (-0.42, 0.76) | 0.46  (0.12, 0.80) | Dapagliflozin |  |  |  |  |  |  |  |  |  |  |  |  |  |  |  |  |  |  |  |  |
| 0.42  (-0.04, 0.86) | 0.21  (-0.41, 0.81) | 0.51  (0.13, 0.89) | 0.04  (-0.28, 0.38) | Dulaglutide |  |  |  |  |  |  |  |  |  |  |  |  |  |  |  |  |  |  |  |
| 0.55  (0.06, 1.00) | 0.34  (-0.27, 0.96) | 0.63  (0.25, 1.01) | 0.17  (-0.18, 0.52) | 0.12  (-0.26, 0.49) | Empagliflozin |  |  |  |  |  |  |  |  |  |  |  |  |  |  |  |  |  |  |
| 0.88  (-0.28, 2.17) | 0.68  (-0.54, 2.00) | 0.96  (-0.14, 2.19) | 0.50  (-0.58, 1.74) | 0.46  (-0.65, 1.72) | 0.34  (-0.76, 1.59) | Enavogliflozin |  |  |  |  |  |  |  |  |  |  |  |  |  |  |  |  |  |
| 0.25  (-0.26, 0.75) | 0.04  (-0.59, 0.69) | 0.33  (-0.07, 0.75) | -0.13  (-0.51, 0.25) | -0.17  (-0.58, 0.22) | -0.29  (-0.72, 0.12) | -0.63  (-1.90, 0.50) | Ertugliflozin |  |  |  |  |  |  |  |  |  |  |  |  |  |  |  |  |
| 1.22  (0.57, 1.89) | 1.01  (0.24, 1.81) | 1.30  (0.67, 1.97) | 0.84  (0.21, 1.50) | 0.80  (0.19, 1.43) | 0.67  (0.03, 1.34) | 0.32  (-1.03, 1.59) | 0.98  (0.30, 1.65) | Exenatide |  |  |  |  |  |  |  |  |  |  |  |  |  |  |  |
| -0.03  (-0.56, 0.49) | -0.23  (-0.96, 0.46) | 0.05  (-0.48, 0.57) | -0.41  (-0.91, 0.10) | -0.45  (-0.93, 0.04) | -0.58  (-1.11, -0.04) | -0.92  (-2.22, 0.27) | -0.27  (-0.84, 0.26) | -1.25  (-1.70, -0.82) | Insulin |  |  |  |  |  |  |  |  |  |  |  |  |  |  |
| 0.44  (-0.02, 0.88) | 0.22  (-0.37, 0.83) | 0.52  (0.16, 0.88) | 0.05  (-0.26, 0.38) | 0.01  (-0.34, 0.37) | -0.11  (-0.47, 0.25) | -0.45  (-1.69, 0.65) | 0.19  (-0.20, 0.58) | -0.78  (-1.43, -0.15) | 0.46  (-0.05, 0.99) | Linagliptin |  |  |  |  |  |  |  |  |  |  |  |  |  |
| 0.56  (0.05, 1.08) | 0.36  (-0.33, 1.01) | 0.64  (0.19, 1.11) | 0.18  (-0.24, 0.62) | 0.14  (-0.30, 0.58) | 0.02  (-0.44, 0.46) | -0.32  (-1.59, 0.82) | 0.31  (-0.17, 0.80) | -0.65  (-1.35, 0.01) | 0.59  (0.03, 1.14) | 0.13  (-0.32, 0.59) | Liraglutide |  |  |  |  |  |  |  |  |  |  |  |  |
| 0.76  (0.27, 1.26) | 0.56  (-0.11, 1.21) | 0.85  (0.42, 1.27) | 0.39  (-0.02, 0.78) | 0.34  (-0.07, 0.77) | 0.22  (-0.22, 0.66) | -0.13  (-1.37, 0.99) | 0.52  (0.05, 0.98) | -0.46  (-1.14, 0.19) | 0.79  (0.27, 1.33) | 0.32  (-0.08, 0.75) | 0.20  (-0.30, 0.69) | Lixisenatide |  |  |  |  |  |  |  |  |  |  |  |
| 0.80  (0.33, 1.27) | 0.60  (-0.01, 1.21) | 0.89  (0.50, 1.28) | 0.43  (0.07, 0.79) | 0.39  (0.01, 0.75) | 0.26  (-0.15, 0.65) | -0.09  (-1.31, 1.03) | 0.56  (0.15, 0.99) | -0.41  (-1.09, 0.22) | 0.84  (0.31, 1.39) | 0.37  (0.03, 0.71) | 0.24  (-0.22, 0.72) | 0.05  (-0.41, 0.49) | Metformin |  |  |  |  |  |  |  |  |  |  |
| 0.21  (-0.37, 0.79) | 0.01  (-0.71, 0.69) | 0.29  (-0.21, 0.81) | -0.17  (-0.63, 0.32) | -0.21  (-0.71, 0.30) | -0.34  (-0.84, 0.18) | -0.67  (-1.97, 0.51) | -0.04  (-0.56, 0.51) | -1.01  (-1.76, -0.29) | 0.25  (-0.39, 0.88) | -0.23  (-0.73, 0.27) | -0.35  (-0.92, 0.22) | -0.55  (-1.07, -0.01) | -0.60  (-1.11, -0.05) | Omarigliptin |  |  |  |  |  |  |  |  |  |
| 0.15  (-0.24, 0.53) | -0.05  (-0.61, 0.49) | 0.24  (-0.03, 0.52) | -0.22  (-0.44, -0.02) | -0.27  (-0.53, -0.01) | -0.40  (-0.67, -0.12) | -0.73  (-1.97, 0.33) | -0.09  (-0.41, 0.22) | -1.06  (-1.69, -0.48) | 0.18  (-0.28, 0.65) | -0.28  (-0.54, -0.03) | -0.41  (-0.79, -0.04) | -0.61  (-0.95, -0.27) | -0.66  (-0.95, -0.36) | -0.06  (-0.50, 0.36) | Placebo |  |  |  |  |  |  |  |  |
| 1.12  (0.69, 1.58) | 0.93  (0.32, 1.53) | 1.21  (0.86, 1.60) | 0.75  (0.44, 1.09) | 0.71  (0.37, 1.06) | 0.59  (0.22, 0.94) | 0.25  (-0.98, 1.34) | 0.88  (0.48, 1.29) | -0.09  (-0.75, 0.55) | 1.16  (0.65, 1.68) | 0.69  (0.36, 1.03) | 0.57  (0.12, 1.00) | 0.36  (-0.06, 0.79) | 0.32  (-0.04, 0.68) | 0.92  (0.43, 1.40) | 0.98  (0.75, 1.22) | Saxagliptin |  |  |  |  |  |  |  |
| 0.13  (-0.52, 0.76) | -0.07  (-0.82, 0.67) | 0.22  (-0.34, 0.77) | -0.25  (-0.81, 0.29) | -0.29  (-0.88, 0.27) | -0.41  (-0.98, 0.17) | -0.76  (-2.06, 0.45) | -0.11  (-0.72, 0.47) | -1.08  (-1.87, -0.30) | 0.17  (-0.53. 0.86) | -0.30  (-0.87, 0.27) | -0.43  (-1.08, 0.20) | -0.63  (-1.25, -0.01) | -0.67  (-1.26, -0.10) | -0.08  (-0.75, 0.57) | -0.02  (-0.54, 0.48) | -1.00  (-1.57, -0.43) | Semaglutide |  |  |  |  |  |  |
| 1.19  (0.76, 1.62) | 0.99  (0.39, 1.59) | 1.28  (0.93, 1.63) | 0.82  (0.49, 1.14) | 0.77  (0.42, 1.11) | 0.65  (0.26, 1.01) | 0.31  (-0.89, 1.41) | 0.94  (0.55, 1.34) | -0.03  (-0.69, 0.58) | 1.22  (0.72, 1.75) | 0.76  (0.42, 1.09) | 0.63  (0.20, 1.07) | 0.43  (0.00, 0.85) | 0.39  (0.09, 0.69) | 0.98  (0.49, 1.48) | 1.04  (0.79, 1.29) | 0.06  (-0.27, 0.40) | 1.05  (0.50, 1.64) | Sitagliptin |  |  |  |  |  |
| -0.10  (-0.58, 0.36) | -0.31  (-0.93, 0.31) | -0.02  (-0.41, 0.38) | -0.47  (-0.84, -0.11) | -0.52  (-0.91, -0.15) | -0.65  (-1.05, -0.26) | -0.98  (-2.22, 0.11) | -0.35  (-0.79, 0.08) | -1.32  (-1.99, -0.66) | -0.07  (-0.61, 0.47) | -0.53  (-0.93, -0.15) | -0.66  (-1.15, -0.19) | -0.86  (-1.31, -0.41) | -0.91  (-1.33, -0.49) | -0.31  (-0.84, 0.21) | -0.25  (-0.55, 0.04) | -1.23  (-1.62, -0.85) | -0.23  (-0.82, 0.35) | -1.29  (-1.68, -0.93) | Sotagliflozin |  |  |  |  |
| -0.15  (-0.56, 0.25) | -0.35  (-0.92, 0.21) | -0.06  (-0.39, 0.26) | -0.53  (-0.81, -0.24) | -0.56  (-0.89, -0.25) | -0.70  (-1.03, -0.37) | -1.03  (-2.26, 0.07) | -0.40  (-0.75. -0.04) | -1.37  (-2.00, -0.77) | -0.12  (-0.61, 0.37) | -0.58  (-0.84, -0.33) | -0.71  (-1.13, -0.30) | -0.91  (-1.30, -0.52) | -0.95  (-1.26, -0.66) | -0.36  (-0.86, 0.13) | -0.30  (-0.51, -0.10) | -1.27  (-1.58, -0.99) | -0.28  (-0.82, 0.28) | -1.34  (-1.61, -1.07) | -0.05  (-0.40, 0.31) | Sulfonylurea |  |  |  |
| 0.20  (-0.25, 0.65) | 0.00  (-0.60, 0.58) | 0.28  (-0.07, 0.65) | -0.17  (-0.50, 0.15) | -0.22  (-0.56, 0.11) | -0.35  (-0.72, 0.02) | -0.69  (-1.91, 0.41) | -0.05  (-0.43, 0.33) | -1.02  (-1.67, -0.40) | 0.23  (-0.29, 0.74) | -0.23  (-0.55, 0.08) | -0.36  (-0.80, 0.08) | -0.56  (-0.98, -0.14) | -0.61  (-0.87, -0.33) | -0.01  (-0.52, 0.49) | 0.05  (-0.20, 0.30) | -0.93  (-1.27, -0.60) | 0.07  (-0.50, 0.64) | -0.99  (-1.29, -0.69) | 0.30  (-0.09, 0.69) | 0.35  (0.12, 0.58) | Thiazolidinedione |  |  |
| 0.90  (-0.51, 2.26) | 0.68  (-0.76, 2.12) | 0.98  (-0.41, 2.33) | 0.52  (-0.86, 1.86) | 0.47  (-0.93, 1.83) | 0.34  (-1.06, 1.68) | -0.01  (-1.86, 1.75) | 0.64  (-0.76, 1.99) | -0.33  (-1.88, 1.11) | 0.93  (-0.52, 2.31) | 0.46  (-0.93, 1.82) | 0.33  (-1.07, 1.67) | 0.13  (-1.26, 1.49) | 0.08  (-1.30, 1.45) | 0.70  (-0.77, 2.11) | 0.74  (-0.62, 2.07) | -0.24  (-1.64, 1.10) | 0.76  (-0.67, 2.18) | -0.31  (-1.68, 1.06) | 1.00  (-0.39, 2.37) | 1.04  (-0.36, 2.37) | 0.69  (-0.69, 2.04) | Tofogliflozin |  |
| 1.74  (0.89, 2.65) | 1.53  (0.61, 2.55) | 1.82  (1.02, 2.69) | 1.35  (0.57, 2.21) | 1.32  (0.53, 2.19) | 1.19  (0.38, 2.06) | 0.85  (-0.55, 2.16) | 1.49  (0.66, 2.38) | 0.52  (-0.44, 1.54) | 1.77  (0.91, 2.70) | 1.30  (0.52, 2.16) | 1.17  (0.34, 2.06) | 0.97  (0.15, 1.86) | 0.93  (0.14, 1.78) | 1.53  (0.64, 2.46) | 1.58  (0.82, 2.40) | 0.60  (-0.21, 1.48) | 1.61  (0.71, 2.58) | 0.54  (-0.25, 1.39) | 1.84  (-1.03, 2.74) | 1.88  (1.09, 2.73) | 1.54  (0.75, 2.39) | 0.87  (-0.68, 2.44) | Vildagliptin |

The columns present the row anti-diabetic agents compared to the column anti-diabetic agents. The rows present the row drug class compared to the column individual anti-diabetic agents. The effect estimates are expressed as odds ratio and 95% confidence intervals. For example, the odd ratio for non-vertebral fracture with Albiglutide compared to Alogliptin is 0.20 (95% confidence interval -0.46 to 0.86).

Table S16. Network estimated effect sizes (95% confidence interval) for Non-Vertebral Fracture_Treatment Type

| DPP4i |  |  |  |  |  |  |  |
| --- | --- | --- | --- | --- | --- | --- | --- |
| -0.54 (-0.74, -0.34) | GLP1RA |  |  |  |  |  |  |
| -1.40 (-1.80, -1.04) | -0.86 (-1.19, -0.56) | Insulin |  |  |  |  |  |
| -0.58 (-0.83, -0.34) | -0.04 (-0.33, 0.24) | 0.82 (0.40, 1.25) | Metformin |  |  |  |  |
| -0.90 (-1.03, -0.76) | -0.36 (-0.51, -0.20) | 0.51 (0.16, 0.88) | -0.31 (-0.57, -0.06) | Placebo |  |  |  |
| -0.74 (-0.90, -0.57) | -0.20 (-0.39, -0.01) | 0.66 (0.31, 1.05) | -0.16 (-0.43, 0.11) | 0.16 (0.05, 0.27) | SGLT2i |  |  |
| -1.04 (-1.23, -0.86) | -0.50 (-0.74, -0.27) | 0.35 (-0.01, 0.76) | -0.46 (-0.73, -0.19) | -0.15 (-0.34, 0.04) | -0.31 (-0.51, -0.10) | Sulfonylurea |  |
| -0.89 (-1.11, -0.66) | -0.35 (-0.62, -0.09) | 0.51 (0.11, 0.92) | -0.31 (-0.56, -0.05) | 0.00 (-0.22, 0.24) | -0.16 (-0.40, 0.10) | 0.15 (-0.06, 0.37) | Thiazolidinedione |

The columns present the row treatment type compared to the column treatment type. The rows present the row drug class compared to the column treatment type. The effect estimates are expressed as odds ratio and 95% confidence intervals. For example, the odd ratio for lower extremity fracture with DPP4i compared to GLP1RA is -0.54 (95% confidence interval -0.74 to 0.34).

Table S17. Network estimated effect sizes (95% confidence interval) for Vertebral & Hip Fracture

| Albiglutide |  |  |  |  |  |  |  |  |  |  |  |  |  |  |  |  |  |  |  |  |  |  |  |
| --- | --- | --- | --- | --- | --- | --- | --- | --- | --- | --- | --- | --- | --- | --- | --- | --- | --- | --- | --- | --- | --- | --- | --- |
| -0.47  (-1.74, 0.71) | Alogliptin |  |  |  |  |  |  |  |  |  |  |  |  |  |  |  |  |  |  |  |  |  |  |
| -0.02  (-0.73, 0.66) | 0.45  (-0.70, 1.67) | Canagliflozin |  |  |  |  |  |  |  |  |  |  |  |  |  |  |  |  |  |  |  |  |  |
| 0.12  (-0.57, 0.77) | 0.58  (-0.55, 1.80) | 0.14  (-0.38, 0.66) | Dapagliflozin |  |  |  |  |  |  |  |  |  |  |  |  |  |  |  |  |  |  |  |  |
| -0.02  (-0.70, 0.64) | 0.44  (-0.70, 1.63) | 0.00  (-0.55, 0.51) | -0.14  (-0.64, 0.35) | Dulaglutide |  |  |  |  |  |  |  |  |  |  |  |  |  |  |  |  |  |  |  |
| 0.76  (0.00, 1.52) | 1.24  (0.03, 2.47) | 0.78  (0.17, 1.39) | 0.64  (0.07, 1.22) | 0.79  (0.19, 1.39) | Empagliflozin |  |  |  |  |  |  |  |  |  |  |  |  |  |  |  |  |  |  |
| 0.08  (-0.64, 0.78) | 0.55  (-0.59, 1.78) | 0.09  (-0.47, 0.66) | -0.05  (-0.58, 0.49) | 0.10  (-0.44, 0.63) | -0.69  (-1.31, -0.05) | Ertugliflozin |  |  |  |  |  |  |  |  |  |  |  |  |  |  |  |  |  |
| 1.80  (-0.89, 5.37) | 2.28  (-0.50, 5.93) | 1.82  (-0.81, 5.34) | 1.70  (-0.97, 5.25) | 1.83  (-0.80, 5.39) | 1.04  (-1.58, 4.59) | 1.74  (-0.93, 5.28) | Evogliptin |  |  |  |  |  |  |  |  |  |  |  |  |  |  |  |  |
| 1.05  (-0.04, 2.27) | 1.54  (0.06, 3.13) | 1.08  (0.04, 2.26) | 0.95  (-0.09, 2.10) | 1.09  (0.05, 2.24) | 0.30  (-0.78, 1.51) | 0.98  (-0.08, 2.18) | -0.72  (-4.39, 2.06) | Exenatide |  |  |  |  |  |  |  |  |  |  |  |  |  |  |  |
| -0.26  (-1.19, 0.66) | 0.20  (-1.16, 1.66) | -0.24  (-1.19, 0.69) | -0.38  (-1.32, 0.55) | -0.24  (-1.15, 0.62) | -1.01  (-2.01, -0.04) | -0.34  (-1.29, 0.63) | -2.07  (-5.79, 0.60) | -1.33  (-2.57, -0.23) | Insulin |  |  |  |  |  |  |  |  |  |  |  |  |  |  |
| -0.49  (-1.17, 0.13) | -0.03  (-1.18, 1.15) | -0.48  (-0.99, 0.02) | -0.61  (-1.08, -0.15) | -0.47  (-0.95, 0.00) | -1.26  (-1.83, -0.68) | -0.57  (-1.10, -0.05) | -2.29  (-5.85, 0.32) | -1.55  (-2.70, -0.57) | -0.23  (-1.13, 0.69) | Linagliptin |  |  |  |  |  |  |  |  |  |  |  |  |  |
| 0.87  (0.11, 1.63) | 1.34  (0.10, 2.63) | 0.88  (0.25, 1.54) | 0.74  (0.15, 1.39) | 0.89  (0.29, 1.53) | 0.10  (-0.62, 0.83) | 0.80  (0.12, 1.45) | -0.94  (-4.47, 1.64) | -0.19  (-1.36, 0.84) | 1.13  (0.20, 2.05) | 1.36  (0.79, 1.97) | Liraglutide |  |  |  |  |  |  |  |  |  |  |  |  |
| 0.80  (-0.06, 1.63) | 1.28  (-0.01, 2.61) | 0.81  (0.07, 1.59) | 0.68  (-0.05, 1.44) | 0.83  (0.09, 1.58) | 0.04  (-0.77, 0.84) | 0.72  (-0.04, 1.51) | -1.00  (-4.58, 1.68) | -0.27  (-1.54, 0.90) | 1.06  (0.00, 2.16) | 1.30  (0.59, 2.03) | -0.06  (-0.89, 0.76) | Lixisenatide |  |  |  |  |  |  |  |  |  |  |  |
| -0.34  (-1.26, 0.53) | 0.12  (-1.16, 1.47) | -0.31  (-1.12, 0.45) | -0.46  (-1.22, 0.29) | -0.32  (-1.07, 0.44) | -1.10  (-1.92, -0.28) | -0.41  (-1.20, 0.36) | -2.16  (-5.77, 0.51) | -1.41  (-2.70, -0.22) | -0.07  (-1.19, 1.03) | 0.16  (-0.55, 0.86) | -1.21  (-2.09, -0.36) | -1.14  (-2.08, -0.18) | Metformin |  |  |  |  |  |  |  |  |  |  |
| -1.00  (-1.84, -0.20) | -0.53  (-1.81, 0.81) | -0.97  (-1.71, -0.28) | -1.12  (-1.80, -0.42) | -0.97  (-1.69, -0.28) | -1.67  (-2.53, -1.00) | -1.07  (-1.79, -0.37) | -2.82  (-6.34, -0.12) | -2.07  (-3.33, -0.91) | -0.73  (-1.80, 0.30) | -0.50  (-1.19, 0.18) | -1.86  (-2.69, -1.07) | -1.80  (-2.71, -0.92) | -0.65  (-1.56, 0.25) | Omarigliptin |  |  |  |  |  |  |  |  |  |
| -0.24  (-0.83, 0.31) | 0.22  (-0.88, 1.39) | -0.22  (-0.62, 0.17) | -0.36  (-0.69, -0.01) | -0.21  (-0.57, 0.14) | -1.00  (-1.50, -0.52) | -0.32  (-0.72, 0.09) | -2.05  (-5.58, 0.52) | -1.30  (-2.40, -0.30) | 0.02  (-0.84, 0.93) | 0.26  (-0.07, 0.59) | -1.10  (-1.65, -0.60) | -1.04  (-1.70, -0.42) | 0.10  (-0.58, 0.79) | 0.76  (0.17, 1.37) | Placebo |  |  |  |  |  |  |  |  |
| 1.06  (0.32, 1.84) | 1.52  (0.32, 2.82) | 1.08  (0.44, 1.73) | 0.94  (0.35, 1.57) | 1.09  (0.46, 1.74) | -0.30  (-0.40, 1.01) | 0.98  (0.34, 1.66) | -0.77  (-4.29, 1.91) | 0.00  (-1.22, 1.06) | 1.32  (0.34, 2.35) | 1.55  (0.99, 2.17) | 0.19  (-0.53, 0.92) | 0.26  (-0.58, 1.09) | 1.40  (0.58, 2.26) | 2.05  (1.28, 2.88) | 1.30  (0.79, 1.85) | Saxagliptin |  |  |  |  |  |  |  |
| 0.14  (-0.71, 1.02) | 0.63  (-0.66, 1.91) | 0.16  (-0.53, 0.89) | 0.03  (-0.67, 0.72) | 0.17  (-0.54, 0.90) | -0.61  (-1.37, 0.15) | 0.07  (-0.63, 0.80) | -1.67  (-5.30, 0.99) | -0.91  (-2.18, 0.23) | 0.40  (-0.66, 1.51) | 0.65  (-0.04, 1.34) | -0.72  (-1.49, 0.09) | -0.65  (-1.55, 0.24) | 0.49  (-0.41, 1.43) | 1.16  (0.31, 2.00) | 0.39  (-0.23, 1.00) | -0.91  (-1.72, -0.11) | Semaglutide |  |  |  |  |  |  |
| 0.91  (0.21, 1.60) | 1.38  (0.23, 2.60) | 0.93  (0.39, 1.50) | 0.79  (0.26, 1.35) | 0.95  (0.41, 1.51) | 0.15  (-0.48, 0.80) | 0.84  (0.25, 1.44) | -0.90  (-4.44, 1.67) | -0.15  (-1.32, 0.91) | 1.18  (0.24, 2.16) | 1.41  (0.89, 1.94) | 0.05  (-0.60, 0.69) | 0.12  (-0.66, 0.87) | 1.26  (0.51, 2.02) | 1.92  (1.19, 2.66) | 1.15  (0.75, 1.58) | -0.13  (-0.84, 0.50) | 0.78  (0.06, 1.47) | Sitagliptin |  |  |  |  |  |
| -0.15  (-0.95, 0.63) | 0.30  (-0.93, 1.57) | -0.15  (-0.83, 0.55) | -0.28  (-0.91, 0.37) | -0.13  (-0.78, 0.53) | -0.92  (-1.65, -0.21) | -0.24  (-0.89, 0.46) | -1.97  (-5.45, 0.70) | -1.23  (-2.45, -0.10) | 0.10  (-0.93, 1.12) | 0.33  (-0.30, 0.99) | -1.03  (-1.80, -0.28) | -0.96  (-1.85, -0.13) | 0.18  (-0.68, 1.06) | 0.84  (0.04, 1.67) | 0.08  (-0.46, 0.62) | -1.22  (-1.97, -0.47) | -0.31  (-1.13, 0.51) | -1.08  (-1.79, -0.40) | Sotagliflozin |  |  |  |  |
| -0.03  (-0.70, 0.60) | 0.43  (-0.67, 1.59) | -0.01  (-0.56, 0.52) | -0.15  (-0.65, 0.35) | -0.01  (-0.53, 0.50) | -0.79  (-1.40, -0.20) | -0.10  (-0.65, 0.43) | -1.85  (-5.35, 0.79) | -1.09  (-2.17, -0.15) | 0.23  (-0.67, 1.15) | 0.46  (0.09, 0.85) | 0.90  (-1.51, -0.33) | -0.83  (-1.59, -0.12) | 0.30  (-0.44, 1.07) | 0.96  (0.26, 1.69) | 0.21  (-0.17, 0.59) | -1.09  (-1.70, -0.52) | -0.19  (-0.91, 0.53) | -0.95  (-1.46, -0.44) | 0.13  (-0.52, 0.78) | Sulfonylurea |  |  |  |
| -0.64  (-1.43, 0.14) | -0.17  (-1.36, 1.07) | -0.61  (-1.28, 0.06) | -0.75  (-1.39, -0.13) | -0.61  (-1.28, 0.06) | -1.39  (-2.14, -0.68) | -0.70  (-1.39, -0.03) | -2.43  (-5.98, 0.23) | -1.70  (-2.87, -0.66) | -0.37  (-1.36, 0.60) | -0.13  (-0.72, 0.46) | -1.50  (-2.23, -0.77) | -1.43  (-2.27, -0.61) | -0.29  (-1.15, 0.56) | 0.37  (-0.44, 1.20) | -0.39  (-0.95, 0.16) | -1.69  (-2.43, -0.99) | -0.78  (-1.63, 0.03) | -1.55  (-2.22, -0.91) | -0.47  (-1.28, 0.29) | -0.60  (-1.12, -0.10) | Thiazolidinedione |  |  |
| -0.46  (-3.11, 2.96) | 0.04  (-2.77, 3.65) | -0.42  (-3.04, 3.04) | -0.56  (-3.15, 2.92) | -0.42  (-3.02, 3.03) | -1.21  (-3.82, 2.24) | -0.53  (-3.12, 2.93) | -2.26  (-6.57, 1.99) | -1.52  (-4.27, 2.05) | -0.20  (-2.91, 3.30) | 0.04  (-2.53, 3.55) | -1.32  (-3.95, 2.12) | -1.24  (-3.94, 2.26) | -0.10  (-2.80, 3.40) | 0.54  (-2.12, 4.03) | -0.21  (-2.76, 3.27) | -1.50  (-4.13, 1.97) | -0.59  (-3.30, 2.86) | -1.39  (-3.95, 2.17) | -0.30  (-2.92, 3.20) | -0.43  (-3.05, 3.12) | 0.18  (-2.43, 3.69) | Tirzepatide |  |
| 0.82  (-0.16, 1.79) | 1.28  (-0.06, 2.69) | 0.84  (-0.06, 1.78) | 0.69  (-0.15, 1.59) | 0.83  (0.00, 1.78) | 0.05  (-0.83, 1.02) | 0.74  (-0.13, 1.67) | -1.01  (-4.48, 1.70) | -0.25  (-1.59, 1.04) | 1.06  (-0.09, 2.29) | 1.31  (0.50, 2.22) | -0.04  (-0.97, 0.91) | 0.02  (-1.00, 1.08) | 1.16  (0.20, 2.13) | 1.82  (0.83, 2.84) | 1.05  (0.29, 1.91) | -0.24  (-1.20, 0.76) | 0.66  (-0.32, 1.69) | -0.10  (-0.97, 0.82) | 0.96  (0.08, 1.98) | 0.84  (0.00, 1.75) | 1.44  (0.51, 2.44) | 1.25  (-2.24, 4.03) | Vildagliptin |

The columns present the row anti-diabetic agents compared to the column anti-diabetic agents. The rows present the row drug class compared to the column individual anti-diabetic agents. The effect estimates are expressed as odds ratio and 95% confidence intervals. For example, the odd ratio for vertebral and hip fracture with Albiglutide compared to Alogliptin is -0.47 (95% confidence interval -1.74 to 0.71).

Table S18. Network estimated effect sizes (95% confidence interval) for Vertebral & Hip Fracture_Treatment Type

| DPP4i |  |  |  |  |  |  |  |
| --- | --- | --- | --- | --- | --- | --- | --- |
| 0.06 (-0.25, 0.36) | GLP1RA |  |  |  |  |  |  |
| -0.54 (-1.24, 0.11) | -0.59 (-1.22, -0.01) | Insulin |  |  |  |  |  |
| -0.15 (-0.90, 0.64) | -0.21 (-1.01, 0.63) | 0.39 (-0.60, 1.44) | Metformin |  |  |  |  |
| -0.40 (-0.62, -0.19) | -0.45 (-0.71, -0.20) | 0.13 (-0.50, 0.81) | -0.25 (-1.04, 0.52) | Placebo |  |  |  |
| -0.13 (-0.41, 0.16) | -0.19 (-0.50, 0.13) | 0.40 (-0.25, 1.12) | 0.02 (-0.79, 0.81) | 0.27 (0.08, 0.46) | SGLT2i |  |  |
| -0.36 (-0.65, -0.06) | -0.41 (-0.79, -0.04) | 0.17 (-0.51, 0.94) | -0.21 (-1.07, 0.61) | 0.04 (-0.28, 0.36) | -0.23 (-0.58, 0.13) | Sulfonylurea |  |
| -0.37 (-0.73, -0.01) | -0.43 (-0.85, 0.00) | 0.16 (-0.55, 0.96) | -0.22 (-1.09, 0.63) | 0.03 (-0.33, 0.39) | -0.24 (-0.64, 0.17) | -0.01 (-0.25, 0.23) | Thiazolidinedione |

The columns present the row treatment type compared to the column treatment type. The rows present the row drug class compared to the column treatment type. The effect estimates are expressed as odds ratio and 95% confidence intervals. For example, the odd ratio for vertebral and hip fracture with DPP4i compared to GLP1RA is 0.06 (95% confidence interval -0.25 to 0.36).

Table S19. Network estimated effect sizes (95% confidence interval) for Multiple Fracture

| Alogliptin |  |  |  |  |  |  |  |  |  |  |  |
| --- | --- | --- | --- | --- | --- | --- | --- | --- | --- | --- | --- |
| -1.46  (-5.48, 1.89) | Dapagliflozin |  |  |  |  |  |  |  |  |  |  |
| -1.33  (-4.49, 1.51) | 0.15  (-3.16, 3.90) | Dulaglutide |  |  |  |  |  |  |  |  |  |
| -0.11  (-3.16, 2.97) | 1.37  (-1.84, 5.21) | 1.19  (-1.43, 4.20) | Empagliflozin |  |  |  |  |  |  |  |  |
| 0.46  (-2.05, 3.14) | 1.94  (-1.44, 6.11) | 1.81  (-1.07, 5.00) | 0.58  (-2.45, 3.68) | Linagliptin |  |  |  |  |  |  |  |
| 0.24  (-3.22, 3.98) | 1.68  (-1.85, 6.07) | 1.55  (-1.63, 5.22) | 0.35  (-2.91, 3.87) | -0.23  (-3.77, 3.54) | Liraglutide |  |  |  |  |  |  |
| -0.81  (-4.85, 2.67) | 0.72  (-3.61, 4.96) | 0.57  (-3.21, 3.97) | -0.64  (-4.53, 2.64) | -1.26  (-5.29, 2.15) | -1.03  (-5.38, 2.69) | Lixisenatide |  |  |  |  |  |
| -0.49  (-2.94, 1.70) | 0.95  (-1.58, 4.21) | 0.81  (-1.00, 2.92) | -0.35  (-2.52, 1.49) | -0.95  (-3.49, 1.20) | -0.71  (-3.75, 1.76) | 0.24  (-2.29, 3.52) | Placebo |  |  |  |  |
| -0.39  (-4.47, 3.26) | 1.13  (-3.78, 5.95) | 0.96  (-3.45, 5.14) | -0.26  (-4.80, 3.84) | -0.84  (-4.69, 2.45) | -0.65  (-5.65, 3.79) | 0.38  (-4.51, 5.47) | 0.11  (-3.94, 3.71) | Rosiglitazone |  |  |  |
| -0.25  (-2.98, 2.30) | 1.20  (-1.62, 4.69) | 1.06  (-1.22, 3.58) | -0.12  (-2.64, 2.14) | -0.72  (-3.59, 1.85) | -0.48  (-3.80, 2.36) | 0.49  (-2.36, 4.09) | 0.23  (-1.07, 1.57) | 0.11  (-3.74, 4.38) | Saxagliptin |  |  |
| 0.43  (-3.23, 4.27) | 1.90  (-1.74, 6.68) | 1.74  (-1.42, 5.72) | 0.55  (-2.90, 4.36) | -0.05  (-3.74, 3.91) | 0.20  (-3.72, 4.37) | 1.22  (-2.47, 5.79) | 0.89  (-1.68, 4.30) | 0.88  (-3.72, 5.94) | 0.67  (-2.26, 4.15) | Sotagliflozin |  |
| 0.60  (-1.54, 3.11) | 2.12  (-1.49, 6.37) | 1.99  (-1.10, 5.23) | 0.78  (-2.57, 3.99) | 0.17  (-1.85, 2.19) | 0.41  (-3.55, 4.09) | 1.43  (-2.21, 5.57) | 1.13  (-1.40, 3.80) | 0.97  (-1.62, 4.49) | 0.92  (-1.94, 3.85) | 0.22  (-3.77, 3.98) | Sulfonylurea |

The columns present the row drug class compared to the column drug class. The rows present the row drug class compared to the column drug class. The effect estimates are expressed as odds ratio and 95% confidence intervals. For example, the odds ratio for multiple fracture with alogliptin compared to dapagliflozin is -0.46 (95% confidence interval -5.48 to 1.89)

Table S20. Network estimated effect sizes (95% confidence interval) for Multiple Fracture_Treatment Type

| DPP-4 |  |  |  |  |  |
| --- | --- | --- | --- | --- | --- |
| -0.55 (-2.17, 1.11) | GLP-1 |  |  |  |  |
| -0.32 (-1.40, 0.72) | 0.21 (-1.05, 1.47) | Placebo |  |  |  |
| -0.07 (-1.72, 1.57) | 0.45 (-1.27, 2.27) | 0.25 (-1.00, 1.55) | SGLT-2 |  |  |
| 0.39 (-1.27, 2.09) | 0.93 (-1.39, 3.27) | 0.70 (-1.22, 2.73) | 0.45 (-1.84, 2.83) | Sulfonylurea |  |
| -0.41 (-4.01, 2.72) | 0.11 (-3.70, 3.56) | -0.09 (-3.79, 3.17) | -0.35 (-4.19, 3.13) | -0.79 (-4.08, 1.82) | Thiazolidinedione |

The columns present the row drug class compared to the column drug class. The rows present the row drug class compared to the column drug class. The effect estimates are expressed as odds ratio and 95% confidence intervals. For example, the odds ratio for multiple fracture with DPP4 inhibitor compared to GLP1 receptor agonists is -0.55 (95% confidence interval -2.17 to 1.11)

Table S21. Network estimated effect sizes (95% confidence interval) for Cranial and Facial bones Fracture

| Alogliptin |  |  |  |  |  |  |  |  |  |  |  |  |  |  |  |  |  |  |
| --- | --- | --- | --- | --- | --- | --- | --- | --- | --- | --- | --- | --- | --- | --- | --- | --- | --- | --- |
| -0.80  (-3.00, 0.89) | Canagliflozin |  |  |  |  |  |  |  |  |  |  |  |  |  |  |  |  |  |
| -2.64  (-5.04, -0.71) | -1.83  (-3.36, -0.47) | Dapagliflozin |  |  |  |  |  |  |  |  |  |  |  |  |  |  |  |  |
| -0.42  (-2.81, 1.52) | 0.39  (-0.90, 1.77) | 2.23  (0.83, 3.81) | Dulaglutide |  |  |  |  |  |  |  |  |  |  |  |  |  |  |  |
| -1.53  (-4.16, 0.58) | -0.70  (-2.42, 0.91) | 1.11  (-0.64, 3.05) | -1.10  (-2.91, 0.59) | Empagliflozin |  |  |  |  |  |  |  |  |  |  |  |  |  |  |
| -0.78  (-3.06, 1.02) | 0.04  (-1.24, 1.28) | 1.87  (0.48, 3.36) | -0.37  (-1.79, 0.96) | 0.72  (-0.98, 2.45) | Ertugliflozin |  |  |  |  |  |  |  |  |  |  |  |  |  |
| -1.91  (-4.11, -0.44) | -1.12  (-2.15, -0.18) | 0.68  (-0.55, 2.05) | -1.52  (-2.80, -0.38) | -0.44  (-1.96, 1.17) | -1.16  (-2.29, -0.05) | Linagliptin |  |  |  |  |  |  |  |  |  |  |  |  |
| -0.90  (-4.24, 2.76) | -0.08  (-2.89, 3.55) | 1.74  (-1.07, 5.42) | -0.46  (-3.29, 3.10) | 0.61  (-2.41, 4.21) | -0.12  (-2.93, 3.48) | 1.06  (-1.63, 4.51) | Liraglutide |  |  |  |  |  |  |  |  |  |  |  |
| -0.85  (-3.25, 1.21) | -0.03  (-1.53, 1.54) | 1.79  (0.20, 3.50) | -0.43  (-2.06, 1.16) | 0.64  (-1.14, 2.60) | -0.08  (-1.56, 1.52) | 1.10  (-0.30, 2.53) | 0.04  (-3.48, 2.97) | Lixisenatide |  |  |  |  |  |  |  |  |  |  |
| -2.78  (-6.96, 1.56) | -1.92  (-5.88, 2.16) | -0.08  (-3.64, 3.71) | -2.30  (-6.29, 1.71) | -1.24  (-5.20, 2.95) | -1.96  (-5.93, 2.06) | -0.78  (-4.66, 3.18) | -1.90  (-6.92, 2.91) | -1.86  (-5.87, 2.33) | Metformin |  |  |  |  |  |  |  |  |  |
| -1.79  (-3.94, -0.22) | -0.99  (-1.92, -0.13) | 0.82  (-0.19, 2.03) | -1.38  (-2.46, -0.45) | -0.30  (-1.72, 1.14) | -1.02  (-1.97, -0.12) | 0.14  (-0.51, 0.81) | -0.914  (-4.36, 1.69) | -0.95  (-2.25, 0.26) | 0.92  (-3.03, 4.67) | Placebo |  |  |  |  |  |  |  |  |
| -1.78  (-4.05, -0.03) | -0.98  (-2.17, 0.12) | 0.84  (-0.39, 2.19) | -1.38  (-2.67, -0.17) | -0.27  (-1.94, 1.31) | -1.01  (-2.22, 0.19) | 0.15  (-0.82, 1.16) | -0.90  (-4.55, 1.76) | -0.94  (-2.38, 0.47) | 0.92  (-3.05, 4.76) | 0.01  (-0.70, 0.73) | Saxagliptin |  |  |  |  |  |  |  |
| -1.32  (-4.18, 1.41) | -0.49  (-2.53, 1.83) | 1.34  (-0.74, 3.77) | -0.88  (-3.00, 1.41) | 0.21  (-2.04, 2.84) | -0.53  (-2.59, 1.84) | 0.63  (-1.26, 2.86) | -0.44  (-4.19, 2.96) | -0.45  (-2.67, 1.93) | 1.44  (-2.89, 5.72) | 0.50  (-1.30, 2.65) | 0.48  (-1.47, 2.76) | Semaglutide |  |  |  |  |  |  |
| -1.33  (-3.56, 0.35) | -0.52  (-1.57, 0.55) | 1.31  (0.14, 2.62) | -0.92  (-2.12, 0.14) | 0.18  (-1.32, 1.72) | -0.55  (-1.68, 0.55) | 0.60  (-0.24, 1.53) | -0.43  (-3.96, 2.27) | -0.48  (-1.88, 0.82) | 1.41  (-2.61, 5.19) | 0.47  (-0.07, 1.06) | 0.47  (-0.44, 1.39) | -0.03  (-2.31, 1.90) | Sitagliptin |  |  |  |  |  |
| -1.82  (-4.61, 0.72) | -1.02  (-2.88, 1.02) | 0.83  (-1.15, 2.98) | -1.41  (-3.33, 0.71) | -0.29  (-2.53, 1.97) | -1.03  (-2.98, 1.03) | 0.15  (-1.70, 2.06) | -0.94  (-4.67, 2.36) | -0.96  (-3.07, 1.14) | 0.92  (-3.38, 5.09) | 0.00  (-1.67, 1.86) | 0.00  (-1.85, 1.91) | -0.49  (-3.32, 2.06) | -0.47  (-2.27, 1.40) | Sotagliflozin |  |  |  |  |
| -2.19  (-4.24, -0.84) | -1.39  (-2.41, -0.51) | 0.43  (-0.88, 1.84) | -1.78  (-3.10, -0.61) | -0.71  (-2.26, 0.91) | -1.43  (-2.65, -026) | -0.26  (-0.89, 0.33) | -1.32  (-4.81, 1.42) | -1.35  (-2.87, 0.02) | 0.51  (-3.51, 4.38) | -0.40  (-1.15, 0.32) | -0.42  (-1.44, 0.62) | -0.90  (-3.17, 1.05) | -0.88  (-1.85, 0.00) | -0.41  (-2.41, 1.45) | Sulfonylurea |  |  |  |
| -2.31  (-4.43, -0.79) | -1.51  (-2.61, -0.52) | 0.30  (-1.01, 1.74) | -1.92  (-3.29, -0.66) | -0.84  (-2.49, 0.84) | -1.55  (-2.83, -0.31) | -0.38  (-1.23, 0.42) | -1.46  (-4.97, 1.32) | -1.45  (-3.02, -0.05) | 0.37  (-3.65, 4.30) | -0.53  (-1.36, 0.27) | -0.54  (-1.64, 0.54) | -1.00  (-3.34, 0.97) | -1.01  (-2.04, -0.03) | -0.54  (-2.52, 1.36) | -0.12  (-0.79, 0.53) | Thiazolidinedione |  |  |
| -1.98  (-5.22, 1.87) | -1.15  (-3.84, 2.53) | 0.71  (-2.08, 4.28) | -1.54  (-4.34, 2.08) | -0.44  (-3.36, 3.29) | -1.16  (-3.96, 2.34) | -0.02  (-2.65, 3.54) | -1.09  (-5.25, 3.16) | -1.09  (-3.96, 2.42) | 0.84  (-3.78, 5.84) | -0.16  (-2.74, 3.36) | -0.17  (-2.84, 3.44) | -0.66  (-3.93, 3.36) | -0.63  (-3.25, 2.85) | -0.14  (-3.20, 3.68) | 0.24  (-2.37, 3.83) | 0.37  (-2.28, 3.86) | Tirzepatide |  |
| -2.78  (-6.68, 0.31) | -1.93  (-5.56, 0.86) | -0.10  (-3.67, 2.75) | -2.33  (-5.95, 0.53) | -1.19  (-4.99, 1.77) | -1.98  (-5.49, 0.84) | -0.79  (-4.26, 1.96) | -1.96  (-6.58, 1.97) | -1.89  (-5.48, 1.08) | -0.03  (-5.05, 4.39) | -0.93  (-4.42, 1.71) | -0.94  (-4.54, 1.75) | -1.50  (-5.45, 1.86) | -1.41  (-4.97, 1.32) | -0.94  (-4.86, 2.19) | -0.50  (-4.04, 2.26) | -0.38  (-3.95, 2.34) | -0.89  (-5.65, 2.84) | Vildagliptin |

The columns present the row anti-diabetic agents compared to the column anti-diabetic agents. The rows present the row drug class compared to the column individual anti-diabetic agents. The effect estimates are expressed as odds ratio and 95% confidence intervals. For example, the odd ratio for cranial and facial bones fracture with Alogliptin compared to Canagliflozin is -0.80 (95% confidence interval -3.00 to 0.89).

Table S22. Network estimated effect sizes (95% confidence interval) for Cranial and Facial bones Fracture_Treatment Type

| DPP4i |  |  |  |  |  |  |
| --- | --- | --- | --- | --- | --- | --- |
| 0.82 (0.14, 1.57) | GLP1RA |  |  |  |  |  |
| 0.23 (-3.61. 3.95) | -0.60 (-4.39, 3.19) | Metformin |  |  |  |  |
| -0.13 (-0.49, 0.21) | -0.95 (-1.62, -0.36) | -0.36 (-4.15, 3.39) | Placebo |  |  |  |
| 0.30 (-0.25, 0.88) | -0.51 (-1.35, 0.25) | 0.07 (-3.69, 3.76) | 0.44 (-0.02, 0.89) | SGLT2i |  |  |
| -0.76 (-1.29, -0.26) | -1.57 (-2.47, -0.79) | -0.99 (-4.73, 2.78) | -0.62 (-1.19, -0.08) | -1.06 (-1.75, -0.39) | Sulfonylurea |  |
| -0.79 (-1.58, -0.08) | -1.61 (-2.65, -0.70) | -1.06 (-4.86, 2.62) | -0.66 (-1.44, 0.05) | -1.09 (-1.97, 0.29) | -0.05 (-0.67, 0.57) | Thiazolidinedione |

The columns present the row treatment type compared to the column treatment type. The rows present the row drug class compared to the column treatment type. The effect estimates are expressed as odds ratio and 95% confidence intervals. For example, the odd ratio for cranial and facial bones fracture with DPP4i compared to GLP1RA is 0.82 (95% confidence interval 0.14 to 1.57).

Table S23. Network estimated effect sizes (95% confidence interval) for Lower Extremity Fracture

| Albiglutide |  |  |  |  |  |  |  |  |  |  |  |  |  |  |  |  |  |  |  |  |  |  |
| --- | --- | --- | --- | --- | --- | --- | --- | --- | --- | --- | --- | --- | --- | --- | --- | --- | --- | --- | --- | --- | --- | --- |
| -0.38  (-1.18, 0.39) | Alogliptin |  |  |  |  |  |  |  |  |  |  |  |  |  |  |  |  |  |  |  |  |  |
| -0.82  (-1.51, 0.17) | -0.44  (-1.16, 0.27) | Canagliflozin |  |  |  |  |  |  |  |  |  |  |  |  |  |  |  |  |  |  |  |  |
| -0.30  (-0.91, 0.29) | 0.09  (-0.56, 0.74) | 0.52  (0.01, 1.04) | Dapagliflozin |  |  |  |  |  |  |  |  |  |  |  |  |  |  |  |  |  |  |  |
| -0.16  (-0.79, 0.45) | 0.22  (-0.45, 0.89) | 0.66  (0.14, 1.21) | 0.13  (-0.32, 0.62) | Dulaglutide |  |  |  |  |  |  |  |  |  |  |  |  |  |  |  |  |  |  |
| -0.44  (-1.13, 0.22) | -0.05  (-0.74, 0.64) | 0.39  (-0.20, 0.97) | -0.13  (-0.64, 0.36) | -0.28  (-0.83, 0.27) | Empagliflozin |  |  |  |  |  |  |  |  |  |  |  |  |  |  |  |  |  |
| -0.51  (-1.27, 0.20) | -0.13  (-0.94, 0.62) | 0.32  (-0.34, 0.95) | -0.21  (-0.81, 0.38) | -0.35  (-0.98, 0.25) | -0.08  (-0.74, 0.57) | Ertugliflozin |  |  |  |  |  |  |  |  |  |  |  |  |  |  |  |  |
| -0.68  (-1.72, 0.23) | -0.30  (-1.37, 0.68) | 0.13  (-0.84, 1.07) | -0.38  (-1.35, 0.53) | -0.52  (-1.50, 0.37) | -0.25  (-1.25, 0.71) | -0.18  (-1.19, 0.82) | Exenatide |  |  |  |  |  |  |  |  |  |  |  |  |  |  |  |
| -0.35  (-1.15, 0.47) | 0.03  (-0.87, 0.91) | 0.48  (-0.36, 1.29) | -0.05  (-0.83, 0.73) | -0.18  (-0.93, 0.54) | 0.09  (-0.72, 0.90) | 0.16  (-0.72, 1.04) | 0.33  (-0.54, 1.27) | Insulin |  |  |  |  |  |  |  |  |  |  |  |  |  |  |
| -0.53  (-1.12, 0.05) | -0.15  (-0.77, 0.50) | 0.30  (-0.20, 0.82) | -0.23  (-0.64, 0.20) | -0.37  (-0.82, 0.11) | -0.09  (-0.59, 0.40) | -0.02  (-0.61, 0.59) | 0.16  (-0.72, 1.11) | -0.19  (-0.94, 0.59) | Linagliptin |  |  |  |  |  |  |  |  |  |  |  |  |  |
| -0.14  (-0.82, 0.55) | 0.25  (-0.48, 0.98) | 0.69  (0.09, 1.31) | 0.17  (-0.39, 0.71) | 0.03  (-0.52, 0.59) | 0.30  (-0.31, 0.94) | 0.38  (-0.30. 1.07) | 0.54  (-0.35, 1.53) | 0.21  (-0.55, 1.01) | 0.39  (-0.15, 0.93) | Liraglutide |  |  |  |  |  |  |  |  |  |  |  |  |
| -0.16  (-0.96, 0.65) | 0.22  (-0.66, 1.05) | 0.66  (-0.07, 1.40) | 0.14  (-0.53, 0.82) | 0.00  (-0.68, 0.70) | 0.28  (-0.48, 1.02) | 0.35  (-0.44, 1.17) | 0.52  (-0.52, 1.61) | 0.17  (-0.69, 1.09) | 0.37  (-0.30, 1.05) | -0.03  (-0.80, 0.74) | Lixisenatide |  |  |  |  |  |  |  |  |  |  |  |
| -1.19  (-1.95, -0.45) | -0.81  (-1.60, -0.02) | -0.37  (-1.07, 0.32) | -0.89  (-1.54, -0.27) | -1.03  (-1.69, 0.37) | -0.75  (-1.35, -0.21) | 0.69  (-1.42, 0.06) | -0.50  (-1.54, 0.52) | -0.85  (-1.74, 0.04) | -0.67  (-1.24, -0.08) | -1.06  (-1.81, -0.35) | -1.04  (-1.84, -0.20) | Metformin |  |  |  |  |  |  |  |  |  |  |
| -0.28  (-1.17, 0.61) | 0.10  (-0.84, 1.00) | 0.54  (-0.27, 1.36) | 0.02  (-0.79, 0.80) | -0.11  (-0.94, 0.69) | 0.16  (-0.69, 1.01) | 0.23  (-0.63, 1.13) | 0.40  (-0.72, 1.55) | 0.07  (-0.96, 1.07) | 0.25  (-0.56, 1.03) | -0.14  (-1.01, 0.69) | -0.12  (-1.04, 0.84) | 0.92  (-0.01, 1.83) | Omarigliptin |  |  |  |  |  |  |  |  |  |
| -0.32  (-0.85, 0.19) | 0.06  (-0.52, 0.65) | 0.50  (0.11, 0.92) | -0.02  (-0.32, 0.29) | -0.15  (-0.51, 0.18) | 0.12  (-0.29, 0.55) | 0.19  (-0.31, 0.70) | 0.36  (-0.48, 1.29) | 0.03  (-0.66, 0.75) | 0.21  (-0.11, 0.52) | -0.18  (-0.65, 0.27) | -0.15  (-0.77, 0.45) | 0.88  (0.32, 1.45) | -0.04  (-0.75, 0.70) | Placebo |  |  |  |  |  |  |  |  |
| -0.22  (-0.89, 0.42) | 0.16  (-0.51, 0.84) | 0.61  (0.05, 1.17) | 0.08  (-0.38, 0.55) | -0.05  (-0.56, 0.47) | 0.22  (-0.33, 0.78) | 0.29  (-0.35, 0.93) | 0.46  (-0.44, 1.44) | 0.13  (-0.65, 0.91) | 0.31  (-0.16. 0.77) | -0.08  (-0.69, 0.51) | -0.06  (-0.76, 0.64) | 0.98  (0.31, 1.65) | 0.05  (-0.76, 0.89) | 0.10  (-0.27, 0.48) | Saxagliptin |  |  |  |  |  |  |  |
| -0.29  (-1.06, 0.47) | 0.09  (-0.72, 0.89) | 0.53  (-0.11, 1.17) | 0.01  (-0.64, 0.64) | -0.13  (-0.77, 0.52) | 0.15  (-0.54, 0.84) | 0.22  (-0.54, 0.98) | 0.40  (-0.61, 1.44) | 0.07  (-0.82, 0.95) | 0.24  (-0.41, 0.86) | -0.16  (-0.84, 0.53) | -0.12  (-0.96, 0.72) | 0.90  (0.13, 1.69) | -0.01  (-0.91, 0.91) | 0.02  (-0.54, 0.59) | -0.08  (-0.74, 0.62) | Semaglutide |  |  |  |  |  |  |
| -0.01  (-0.62, 0.57) | 0.37  (-0.28, 1.03) | 0.81  (0.30, 1.33) | 0.29  (-0.16, 0.74) | 0.15  (-0.32, 0.62) | 0.42  (-0.08, 0.94) | 0.50  (-0.12, 1.10) | 0.67  (-0.25, 1.64) | 0.34  (-0.46, 1.12) | 0.52  (0.10, 0.93) | 0.13  (-0.44, 0.67) | 0.14  (-0.54, 0.85) | 1.18  (0.62, 1.78) | 0.26  (-0.52, 1.08) | 0.30  (-0.03, 0.64) | 0.21  (-0.28, 0.70) | 0.28  (-0.35, 0.91) | Sitagliptin |  |  |  |  |  |
| -0.30  (-0.99, 0.39) | 0.08  (-0.64, 0.79) | 0.52  (-0.05, 1.13) | 0.00  (-0.53, 0.52) | -0.13  (-0.67, 0.41) | 0.14  (-0.45, 0.74) | 0.21  (-0.44, 0.90) | 0.39  (-0.56, 1.40) | 0.05  (-0.75, 0.91) | 0.23  (-0.29, 0.75) | -0.16  (-0.79, 0.48) | -0.14  (-0.87, 0.62) | 0.90  (0.20, 1.60) | -0.01  (-0.86, 0.82) | 0.02  (-0.40, 0.46) | -0.08  (-0.64, 0.48) | -0.01  (-0.71, 0.70) | -0.28  (-0.83, 0.24) | Sotagliflozin |  |  |  |  |
| -0.80  (-1.36, -0.26) | -0.42  (-1.03, 0.16) | 0.02  (-0.46, 0.51) | -0.50  (-0.90, -0.11) | -0.64  (-1.09, -0.19) | -0.37  (-0.84, 0.10) | -0.29  (-0.90, 0.30) | -0.12  (-0.97, 0.78) | -0.45  (-1.22, 0.29) | -0.27  (-0.60, 0.05) | -0.67  (-1.20, -0.16) | -0.64  (-1.32, 0.02) | 0.39  (-0.20, 0.99) | -0.53  (-1.31, 0.28) | -0.49  (-0.78, -0.20) | -0.58  (-1.01, -0.17) | -0.51  (-1.13, 0.11) | -0.79  (-1.17, -0.41) | -0.51  (-1.02, -0.01) | Sulfonylurea |  |  |  |
| -0.23  (-0.99, 0.54) | 0.16  (-0.65, 0.96) | 0.60  (-0.13, 1.31) | 0.08  (-0.58. 0.76) | -0.07  (-0.75, 0.64) | 0.21  (-0.49, 0.94) | 0.29  (-0.49, 1.07) | 0.48  (-0.55, 1.50) | 0.11  (-0.79, 1.03) | 0.30  (-0.32, 0.94) | -0.10  (-0.82, 0.65) | -0.06  (-0.93, 0.78) | 0.97  (0.17, 1.76) | 0.04  (-0.89, 1.02) | 0.09  (-0.51, 0.70) | -0.01  (-0.69, 0.68) | 0.06  (-0.76, 0.88) | -0.22  (-0.87, 0.43) | 0.07  (-0.66, 0.80) | 0.58  (0.03, 1.15) | Thiazolidinedione |  |  |
| -1.58  (-4.87, 0.55) | -1.20  (-4.54, 0.96) | -0.76  (-4.01, 1.34) | -1.25  (-4.56, 0.81) | -1.41  (-4.72, 0.69) | -1.14  (-4.44, 1.02) | -1.06  (-4.30, 1.10) | -0.88  (-4.26, 1.35) | -1.22  (-4.50, 0.97) | -1.04  (-4.35, 1.05) | -1.44  (-4.70, 0.65) | -1.41  (-1.79, 0.71) | -0.38  (-3.67, 1.80) | -1.28  (-4.62, 0.90) | -1.25  (-4.54, 0.80) | -1.35  (-4.60, 0.75) | -1.29  (-4.53, 0.85) | -1.55  (-4.82, 0.52) | -1.27  (-4.59, 0.82) | -0.77  (-4.05, 1.31) | -1.34  (-4.65, 0.79) | Tofogliflozin |  |
| -0.01  (-1.67, 1.69) | 0.37  (-1.31, 2.12) | 0.81  (-0.79, 2.49) | 0.30  (-1.32, 1.97) | 0.16  (-1.43, 1.84) | 0.43  (-1.20, 2.11) | 0.51  (-1.14, 2.21) | 0.69  (-1.05, 2.54) | 0.35  (-1.35, 2.08) | 0.52  (-1.07, 2.20) | 0.11  (-1.53, 1.86) | 0.15  (-1.55, 1.83) | 1.19  (-0.49, 2.92) | 0.25  (-1.46, 2.05) | 0.30  (-1.27, 1.93) | 0.20  (-1.38, 1.90) | 0.28  (-1.34, 2.03) | 0.00  (-1.63, 1.70) | 0.29  (-1.36, 1.96) | 0.79  (-0.79, 2.48) | 0.23  (-1.43, 1.95) | 1.61  (-1.03, 5.17) | Vildagliptin |

The columns present the row anti-diabetic agents compared to the column anti-diabetic agents. The rows present the row drug class compared to the column individual anti-diabetic agents. The effect estimates are expressed as odds ratio and 95% confidence intervals. For example, the odd ratio for lower extremity fracture with Albiglutide compared to Alogliptin is -0.38 (95% confidence interval -1.18 to 0.39).

Table S24. Network estimated effect sizes (95% confidence interval) for Lower Extremity Fracture_Treatment Type

| DPP4i |  |  |  |  |  |  |  |
| --- | --- | --- | --- | --- | --- | --- | --- |
| 0.22 (-0.04, 0.48) | GLP1RA |  |  |  |  |  |  |
| -1.24 (-1.84, -0.68) | -1.47 (-1.98, 0.99) | Insulin |  |  |  |  |  |
| -0.72 (-1.29, -0.14) | -0.94 (-1.57, -0.32) | 0.53 (-0.26, 1.32) | Metformin |  |  |  |  |
| -0.04 (-0.22, 0.14) | -0.27 (-0.48, -0.05) | 1.20 (0.66, 1.78) | 0.68 (0.10, 1.26) | Placebo |  |  |  |
| -0.05 (-0.28, 0.17) | -0.28 (-0.54, -0.02) | 1.18 (0.63, 1.78) | 0.66 (0.06, 1.24) | -0.01 (-0.18, 0.15) | SGLT2i |  |  |
| -0.78 (-1.01, -0.55) | -1.01 (-1.30, -0.72) | 0.46 (-0.11, 1.08) | -0.06 (-0.67, 0.52) | -0.74 (-0.99, 0.49) | -0.72 (-1.00, -0.46) | Sulfonylurea |  |
| -0.76 (-1.23, -0.31) | -0.98 (-1.49, -0.50) | 0.48 (-0.23, 1.21) | -0.04 (-0.78, 0.65) | -0.72 (-1.20, 0.27) | -0.70 (-1.21, -0.24) | 0.02 (-0.44, 0.46) | Thiazolidinedione |

The columns present the row treatment type compared to the column treatment type. The rows present the row drug class compared to the column treatment type. The effect estimates are expressed as odds ratio and 95% confidence intervals. For example, the odd ratio for lower extremity fracture with DPP4i compared to GLP1RA is 0.22 (95% confidence interval -0.04 to 0.48).

Table S25. Network estimated effect sizes (95% confidence interval) for Pelvic Girdle Fracture

| Albiglutide |  |  |  |  |  |  |  |  |  |  |  |  |  |  |  |  |  |  |  |  |
| --- | --- | --- | --- | --- | --- | --- | --- | --- | --- | --- | --- | --- | --- | --- | --- | --- | --- | --- | --- | --- |
| 1.92  (0.40, 3.72) | Canagliflozin |  |  |  |  |  |  |  |  |  |  |  |  |  |  |  |  |  |  |  |
| 1.25  (-0.26, 2.98) | -0.69  (-1.62, 0.17) | Dapagliflozin |  |  |  |  |  |  |  |  |  |  |  |  |  |  |  |  |  |  |
| 0.97  (-0.48. 2.77) | -0.95  (-1.86 -0.08) | -0.27  (-1.12, 0.59) | Dulaglutide |  |  |  |  |  |  |  |  |  |  |  |  |  |  |  |  |  |
| 2.90  (1.34, 4.72) | 0.97  (-0.13, 2.12) | 1.65  (0.65, 2.77) | 1.91  (0.89, 3.04) | Empagliflozin |  |  |  |  |  |  |  |  |  |  |  |  |  |  |  |  |
| 2.41  (0.80, 4.22) | 0.47  (-0.56, 1.46) | 1.14  (0.18, 2.15) | 1.42  (0.45, 2.40) | -0.49  (-1.75, 0.62) | Ertugliflozin |  |  |  |  |  |  |  |  |  |  |  |  |  |  |  |
| 3.93  (1.36, 7.70) | 1.95  (-0.27, 5.43) | 2.61  (0.42, 6.12) | 2.89  (0.71, 6.29) | 0.97  (-1.32, 4.41) | 1.48  (-0.81, 5.00) | Exenatide |  |  |  |  |  |  |  |  |  |  |  |  |  |  |
| -2.52  (-4.56, -1.17) | -4.51  (-7.09, 2.42) | -3.83  (-6.45, 1.76) | -3.55  (-6.16, -1.48) | -5.46  (-8.20, -3.39) | -4.98  (-7.62, -2.84) | -6.55  (-10.54, -3.54) | Insulin |  |  |  |  |  |  |  |  |  |  |  |  |  |
| 1.810  (0.28, 3.63) | -0.13  (-1.15, 0.91) | 0.58  (-0.42, 1.52) | 0.85  (-0.13, 1.82) | -1.07  (-2.24, 0.01) | -0.58  (-1.66, 0.51) | -2.04  (-5.46, -0.03) | 4.37  (2.25, 7.13) | Linagliptin |  |  |  |  |  |  |  |  |  |  |  |  |
| 2.88  (0.91, 5.38) | 0.92  (-0.62, 2.98) | 1.61  (0.09, 3.63) | 1.90  (0.38, 3.87) | -0.06  (-1.68, 2.06) | 0.45  (-1.13, 2.60) | -1.00  (-4.55, 1.83) | 5.47  (3.00, 8.61) | 1.03  (-0.50, 3.15) | Liraglutide |  |  |  |  |  |  |  |  |  |  |  |
| 2.63  (0.89, 4.69) | 0.68  (-0.57, 2.13) | 1.37  (0.18, 2.74) | 1.63  (0.44, 3.04) | -0.27  (-1.74, 1.18) | 0.23  (-1.11, 1.65) | -1.27  (-4.65, 1.19) | 5.22  (2.95, 8.11) | 0.80  (-0.51, 2.24) | -0.24  (-2.43, 1.61) | Lixisenatide |  |  |  |  |  |  |  |  |  |  |
| 0.75  (-1.19, 2.85) | -1.18  (-2.78, 0.26) | -0.49  (-2.07, 0.89) | -0.21  (-1.79, 1.20) | -2.13  (-3.68, -0.83) | -1.65  (-3.25, -0.17) | -3.13  (-6.78, -0.72) | 3.32  (0.97, 6.25) | -1.05  (-2.52, 0.24) | -2.09  (-4.53, -0.20) | -1.89  (-3.71, -0.19) | Metformin |  |  |  |  |  |  |  |  |  |
| 0.15  (-1.35, 1.96) | -1.78  (-2.78, -0.86) | -1.09  (-2.02, -0.16) | -0.82  (-1.75, 0.09) | -2.73  (-3.91, -1.66) | -2.2  (-3.28, -1.23) | -3.71  (-7.16, -1.50) | 2.72  (0.62, 5.40) | -1.66,  (-2.73, -0.59) | -2.70  (-4.78, 1.13) | -2.45  (-3.87, -1.20) | -0.61  (-2.09, 1.00) | Omarigliptin |  |  |  |  |  |  |  |  |
| 0.92  (-0.39, 2.62) | -1.00  (-1.72, -0.34) | -0.32  (-0.92, 0.27) | -0.05  (-0.66, 0.52) | -1.95  (-2.91, -1.16) | -1.47  (-2.27, -0.71) | -2.94  (-6.33, -0.85) | 3.50  (1.53, 6.06) | -0.89  (-1.68, -0.11) | -1.91  (-3.87, -0.55) | -1.68  (-3.00, -0.65) | 0.17  (-1.13, 1.62) | 0.75  (0.08, 1.48) | Placebo |  |  |  |  |  |  |  |
| 1.80  (0.33, 3.55) | -0.12  (-1.04, 0.73) | 0.56  (-0.29, 1.44) | 0.82  (0.00, 1.69) | -1.08  (-2.19, -0.06) | -0.60  (-1.58, 0.38) | -2.08  (-5.50, 0.09) | 4.40  (2.31, 6.96) | -0.01  (-1.00, 0.98) | -1.03  (-3.04, 0.45) | -0.80  (-2.22, 0.39) | 1.04  (-0.36, 2.57) | 1.64  (0.73, 2.59) | 0.87  (0.28, 1.50) | Saxagliptin |  |  |  |  |  |  |
| 0.96  (-0.85, 3.01) | -0.96  (-2.35, 0.34) | -0.29  (-1.62, 1.05) | 0.01  (-1.36, 1.29) | -1.92  (-3.45, -0.49) | -1.42  (-2.86, -0.03) | -2.94  (-6.40, -0.47) | 3.55  (1.22, 6.35) | -0.84  (-2.25, 0.52) | -1.90  (-4.15, -0.11) | -1.67  (-3.40, -0.05) | 0.22  (-1.55, 2.05) | 0.80  (-0.59, 2.16) | 0.04  (-1.12, 1.20) | -0.83  (-2.15, 0.46) | Semaglutide |  |  |  |  |  |
| 1.88  (0.43, 3.66) | -0.04  (-0.90, 0.82) | 0.65  (-0.17, 1.49) | 0.91  (0.09, 1.76) | -0.99  (-2.10, 0.01) | -0.50  (-1.50, 0.50) | -2.00  (-5.33, 0.18) | 4.49  (2.45, 7.04) | 0.08  (-0.81, 1.01) | -0.96  (-2.98, 0.59) | -0.71  (-2.12, 0.45) | 1.13  (-0.27, 2.67) | 1.73  (0.83, 2.65) | 0.96  (0.38, 1.60) | 0.10  (-0.76, 0.95) | 0.93  (-0.31, 2.21) | Sitagliptin |  |  |  |  |
| 1.37  (-0.36, 3.29) | -0.56  (-1.81, 0.68) | 0.12  (-1.08, 1.35) | 0.38  (-0.84, 1.61) | -1.53  (-2.92, -0.16) | -1.02  (-2.34, 0.29) | -2.51  (-5.99, -0.10) | 3.96  (1.72, 6.75) | -0.45  (-1.77, 0.89) | -1.48  (-3.68, 0.30) | -1.24  (-2.93, 0.27) | 0.61  (-1.07, 2.42) | 1.21  (-0.06, 2.50) | 0.44  (-0.60, 1.50) | -0.43  (-1.66, 0.79) | 0.42  (-1.18, 1.98) | -0.52  (-1.75, 0.69) | Sotagliflozin |  |  |  |
| 1.12  (-0.42, 3.06) | -0.78  (-1.88, 0.27) | -0.10  (-1.14, 0.92) | 0.17  (-0.87, 1.18) | -1.75  (-3.04, -0.57) | -1.25  (-2.42, -0.10) | -2.67  (-6.04, -0.82) | 3.71  (1.59, 6.45) | -0.66  (-1.36, 0.04) | -1.70  (-3.87, -0.16) | -1.48  (-2.94, -0.11) | 0.39  (-1.01, 1.94) | 0.99  (-0.11, 2.09) | 0.23  (-0.63, 1.08) | -0.65  (-1.68, 0.37) | 0.19  (-1.24, 1.60) | -0.75  (-1.71, 0.18) | -0.22  (-1.58, 1.13) | Sulfonylurea |  |  |
| 1.45  (-0.23, 3.42) | -0.49  (-1.59, 0.68) | 0.22  (-0.89, 1.38) | 0.49  (-0.65, 1.63) | -1.44  (-2.77, -0.15) | -0.92  (-2.19, 0.27) | -2.38  (-5.79, -0.37) | 4.02  (1.83, 6.76) | -0.35  (-1.28, 0.61) | -1.41  (-3.54, 0.31) | -1.17  (-2.70, 0.32) | 0.70  (-0.79, 2.35) | 1.29  (0.16, 2.50) | 0.53  (-0.39, 1.49) | -0.33  (-1.48, 0.82) | 0.50  (-1.00, 1.97) | -0.44  (-1.48, 0.64) | 0.09  (-1.32, 1.52) | 0.31  (-0.42, 1.08) | Thiazolidinedione |  |
| 3.23  (1.21, 5.88) | 1.27  (-0.27, 3.44) | 1.97  (0.43, 4.06) | 2.22  (-0.71, 4.30) | 0.31  (-1.34, 2.40) | 0.82  (-0.81, 2.92) | -0.62  (-4.18, 2.10) | 5.84  (3.31, 8.96) | 1.41  (-0.18, 3.49) | 0.38  (-2.07, 2.73) | 0.61  (-1.33, 2.87) | 2.47  (0.87, 4.71) | 3.06  (1.45, 5.14) | 2.28  (0.87, 4.26) | 1.42  (-0.11, 3.48) | 2.26  (0.43, 4.57) | 1.30  (-0.18, 3.41) | 1.86  (0.04, 4.11) | 2.08  (0.45, 4.22) | 1.76  (0.00, 3.95) | Vildagliptin |

The columns present the row anti-diabetic agents compared to the column anti-diabetic agents. The rows present the row drug class compared to the column individual anti-diabetic agents. The effect estimates are expressed as odds ratio and 95% confidence intervals. For example, the odd ratio for pelvic girdle fracture with Albiglutide compared to Alogliptin is -0.38 (95% confidence interval -1.18 to 0.39).

Table S26. Network estimated effect sizes (95% confidence interval) for Pelvic Girdle Fracture_Treatment Type

| DPP4i |  |  |  |  |  |  |  |
| --- | --- | --- | --- | --- | --- | --- | --- |
| 0.03 (-0.42, 0.49) | GLP1RA |  |  |  |  |  |  |
| -2.45 (-4.42, -0.96) | -2.48 (-4.38, 1.06) | Insulin |  |  |  |  |  |
| -0.96 (-1.88, -0.07) | -0.99 (-2.00, -0.04) | 1.50 (-0.25, 3.71) | Metformin |  |  |  |  |
| -0.56 (-0.83, -0.28) | -0.59 (-0.97, -0.21) | 1.89 (0.41, 3.89) | 0.40 (-0.49, 1.33) | Placebo |  |  |  |
| -0.34 (-0.72, 0.01) | -0.38 (-0.84, 0.07) | 2.10 (0.63, 4.06) | 0.62 (-0.28, 1.54) | 0.21 (-0.04, 0.47) | SGLT2i |  |  |
| -0.87 (-1.50, -0.29) | -0.90 (-1.61, -0.24) | 1.57 (0.03, 3.66) | 0.08 (-0.99, 1.16) | -0.32 (-0.95, 0.30) | -0.53 (-1.23, 0.14) | Sulfonylurea |  |
| -0.43 (-1.30, 0.42) | -0.47 (-1.36, 0.42) | 2.02 (0.31, 4.21) | 0.52 (0.69, 1.77) | 0.12 (-0.74, 0.97) | -0.08 (-0.98, 0.79) | 0.44 (-0.27, 1.18) | Thiazolidinedione |

The columns present the row treatment type compared to the column treatment type. The rows present the row drug class compared to the column treatment type. The effect estimates are expressed as odds ratio and 95% confidence intervals. For example, the odd ratio for pelvic girdle fracture with DPP4i compared to GLP1RA is 0.03 (95% confidence interval -0.42 to 0.49).

Table S27. Network estimated effect sizes (95% confidence interval) for Upper Extremity Fracture

| Albiglutide |  |  |  |  |  |  |  |  |  |  |  |  |  |  |  |  |  |  |  |  |  |  |
| --- | --- | --- | --- | --- | --- | --- | --- | --- | --- | --- | --- | --- | --- | --- | --- | --- | --- | --- | --- | --- | --- | --- |
| 1.13  (0.03, 2.36) | Alogliptin |  |  |  |  |  |  |  |  |  |  |  |  |  |  |  |  |  |  |  |  |  |
| 0.18  (-0.59, 1.02) | -0.94  (-2.03, 0.02) | Canagliflozin |  |  |  |  |  |  |  |  |  |  |  |  |  |  |  |  |  |  |  |  |
| 0.56  (-0.20, 1.35) | -0.57  (-1.61, 0.40) | 0.37  (-0.17, 0.92) | Dapagliflozin |  |  |  |  |  |  |  |  |  |  |  |  |  |  |  |  |  |  |  |
| 0.51  (-0.29, 1.29) | -0.62  (-1.65, 0.33) | 0.32  (-0.25, 0.91) | -0.05  (-0.59, 0.49) | Dulaglutide |  |  |  |  |  |  |  |  |  |  |  |  |  |  |  |  |  |  |
| 0.57  (-0.23, 1.38) | -0.56  (-1.60, 0.40) | 0.38  (-0.21, 0.94) | 0.01  (-0.53, 0.53) | 0.05  (-0.51, 0.60) | Empagliflozin |  |  |  |  |  |  |  |  |  |  |  |  |  |  |  |  |  |
| -0.15  (-1.72, 1.40) | -1.27  (-2.93, 0.33) | -0.33  (-1.79, 1.09) | -0.71  (-2.11, 0.68) | -0.65  (-2.09, 0.75) | -0.71  (-2.13, 0.66) | Enavogliflozin |  |  |  |  |  |  |  |  |  |  |  |  |  |  |  |  |
| 0.60  (-0.24, 1.48) | -0.51  (-1.62, 0.46) | 0.42  (-0.22, 1.06) | 0.05  (-0.54, 0.65) | 0.09  (-0.54, 0.73) | 0.04  (-0.59, 0.69) | 0.75  (-0.66, 2.24) | Ertugliflozin |  |  |  |  |  |  |  |  |  |  |  |  |  |  |  |
| -0.10  (-1.17, 0.97) | -1.22  (-2.44, -0.06) | -0.28  (-1.18, 0.59) | -0.66  (-1.51, 0.22) | -0.60  (-1.46, 0.21) | -0.67  (-1.54, 0.21) | 0.05  (-1.49, 1.60) | -0.71  (-1.61, 0.25) | Exenatide |  |  |  |  |  |  |  |  |  |  |  |  |  |  |
| -0.65  (-1.68, 0.38) | -1.79  (-2.98, -0.63) | -0.84  (-1.69, 0.01) | -1.21  (-2.04, -0.38) | -1.15  (-1.97, -0.39) | -1.21  (-2.09, -0.36) | -0.51  (-2.08, 1.10) | -1.25  (-2.18, -0.34) | -0.54  (-1.19, 0.05) | Insulin |  |  |  |  |  |  |  |  |  |  |  |  |  |
| 0.87  (0.09, 1.69) | -0.26  (-1.30, 0.71) | 0.68  (0.10, 1.26) | 0.31  (-0.24, 0.86) | 0.36  (-0.20, 0.92) | 0.30  (-0.26, 0.88) | 1.03  (-0.37, 2.43) | 0.26  (-0.36, 0.90) | 0.97  (0.12, 1.84) | 1.51  (0.68, 2.39) | Linagliptin |  |  |  |  |  |  |  |  |  |  |  |  |
| 0.98  (0.11, 1.85) | -0.15  (-1.26, 0.86) | 0.79  (0.13, 1.46) | 0.42  (-0.20, 1.06) | 0.46  (-0.16, 1.13) | 0.40  (-0.22, 1.06) | 1.13  (-0.29, 2.60) | 0.36  (-0.33, 1.09) | 1.07  (0.15, 2.01) | 1.62  (0.74, 2.53) | 0.11  (-0.55, 0.74) | Liraglutide |  |  |  |  |  |  |  |  |  |  |  |
| 1.02  (0.17, 1.90) | -0.12  (-1.18, 0.89) | 0.82  (0.19, 1.47) | 0.46  (-0.15, 1.07) | 0.50  (-0.13, 1.15) | 0.45  (-0.19, 1.08) | 1.16  (-0.25, 2.58) | 0.41  (-0.27, 1.11) | 1.12  (0.20, 2.02) | 1.66  (0.76, 2.58) | 0.14  (-0.50, 0.78) | 0.03  (-0.71, 0.74) | Lixisenatide |  |  |  |  |  |  |  |  |  |  |
| 0.61  (-0.27, 1.50) | -0.52  (-1.62, 0.52) | 0.41  (-0.23, 1.07) | 0.05  (-0.55, 0.66) | 0.09  (-0.50, 0.72) | 0.04  (-0.59, 0.73) | 0.76  (-0.58, 2.11) | 0.00  (-0.71, 0.71) | 0.69  (-0.20, 1.67) | 1.25  (0.38, 2.19) | -0.27  (-0.90, 0.38) | -0.37  (-1.07, 0.36) | -0.41  (-1.12, 0.30) | Metformin |  |  |  |  |  |  |  |  |  |
| 0.91  (-0.16, 2.03) | -0.22  (-1.52, 1.01) | 0.72  (-0.18, 1.70) | 0.35  (-0.51, 1.29) | 0.40  (-0.49, 1.35) | 0.33  (-0.53, 1.31) | 1.06  (-0.51, 2.64) | 0.31  (-0.65, 1.27) | 1.01  (-0.11, 2.20) | 1.56  (0.40, 2.74) | 0.03  (-0.87, 0.99) | -0.07  (-1.04, 0.93) | -0.11  (-1.02, 0.91) | 0.30  (-0.68, 1.35) | Omarigliptin |  |  |  |  |  |  |  |  |
| 0.25  (-0.42, 0.96) | -0.87  (-1.87, 0.02) | 0.07  (-0.36, 0.47) | -0.31  (-0.65, 0.05) | -0.26  (-0.66, 0.15) | -0.31  (-0.71, 0.09) | 0.41  (-0.95, 1.79) | -0.35  (-0.85, 0.14) | 0.35  (-0.43, 1.13) | 0.90  (0.14, 1.68) | -0.62  (-1.04, -0.20) | -0.73  (-1.26, -0.22) | -0.76  (-1.26, -0.28) | -0.36  (-0.88, 0.16) | -0.65  (-1.52, 0.13) | Placebo |  |  |  |  |  |  |  |
| 0.66  (-0.12, 1.43) | -0.47  (-1.49, 0.46) | 0.47  (-0.05, 1.00) | 0.10  (-0.39, 0.58) | 0.14  (-0.38, 0.68) | 0.09  (-0.42, 0.63) | 0.80  (-0.56, 2.23) | 0.05  (-0.54, 0.66) | 0.75  (-0.11, 1.62) | 1.30  (0.48, 2.16) | -0.21  (-0.73, 0.30) | -0.33  (-0.93, 0.30) | -0.36  (-0.96, 0.25) | 0.05  (-0.55, 0.62) | -0.26  (-1.18, 0.61) | 0.41  (0.05, 0.75) | Saxagliptin |  |  |  |  |  |  |
| 0.39  (-0.86, 1.60) | -0.75  (-2.16, 0.59) | 0.19  (-0.92, 1.25) | -0.19  (-1.24, 0.88) | -0.14  (-1.25, 0.92) | -0.18  (-1.27, 0.85) | 0.54  (-1.14, 2.24) | -0.24  (-1.33, 0.84) | 0.46  (-0.79, 1.79) | 1.01  (-0.20, 2.30) | -0.49  (-1.59, 0.58) | -0.60  (-1.76, 0.52) | -0.64  (-1.75, 0.45) | -0.23  (-1.36, 0.87) | -0.54  (-1.85, 0.73) | 0.13  (-0.88, 1.10) | -0.28  (-1.33, 0.75) | Semaglutide |  |  |  |  |  |
| 0.97  (0.21, 1.78) | -0.15  (-1.18, 0.81) | 0.79  (0.24, 1.34) | 0.41  (-0.08, 0.92) | 0.46  (-0.09, 1.01) | 0.42  (-0.12, 0.97) | 1.12  (-0.19, 2.48) | 0.37  (-0.25, 0.99) | 1.07  (0.21, 1.94) | 1.62  (0.80, 2.49) | 0.10  (-0.44, 0.66) | 0.00  (-0.64, 0.64) | -0.03  (-0.66, 0.59) | 0.37  (-0.21, 0.93) | 0.07  (-0.89, 0.94) | 0.72  (0.36, 1.11) | 0.32  (-0.17, 0.82) | 0.60  (-0.47, 1.66) | Sitagliptin |  |  |  |  |
| 0.03  (-0.77, 0.84) | -1.11  (-2.14, -0.12) | -0.16  (-0.73, 0.40) | -0.54  (-1.05, 0.02) | -0.49  (-1.08, 0.09) | -0.54  (-1.12, 0.02) | 0.16  (-1.21, 1.59) | -0.58  (-1.22, 0.06) | 0.12  (-0.78, 1.02) | 0.68  (-0.19, 1.52) | -0.85  (-1.44, -0.28) | -0.95  (-1.62, -0.29) | -0.98  (-1.63, -0.37) | -0.58  (-1.25, 0.07) | -0.89  (-1.83, 0.01) | -0.23  (-0.63, 0.18) | -0.62  (-1.17, -0.10) | -0.36  (-1.39, 0.73) | -0.95  (-1.51, -0.41) | Sotagliflozin |  |  |  |
| 0.38  (-0.34, 1.11) | -0.75  (-1.75, 0.13) | 0.18  (-0.30, 0.68) | -0.19  (-0.62, 0.26) | -0.14  (-0.64, 0.36) | -0.19  (-0.68, 0.31) | 0.52  (-0.82, 1.91) | -0.24  (-0.79, 0.32) | 0.46  (-0.31, 1.27) | 1.02  (0.23, 1.83) | -0.50  (-0.91, -0.10) | -0.60  (-1.16, -0.06) | -0.64  (-1.22, -0.07) | -0.23  (-0.79, 0.31) | -0.53  (-1.46, 0.33) | 0.12  (-0.20, 0.43) | -0.29  (-0.71, 0.14) | 0.00  (-1.05, 1.04) | -0.61  (-1.05, -0.18) | 0.34  (-0.18, 0.87) | Sulfonylurea |  |  |
| 0.08  (-0.73, 0.90) | -1.05  (-2.10, -0.05) | -0.11  (-0.72, 0.51) | -0.48  (-1.02, 0.06) | -0.43  (-1.00, 0.17) | -0.49  (-1.08, 0.11) | 0.23  (-1.12, 1.61) | -0.52  (-1.20, 0.13) | 0.17  (-0.69, 1.05) | 0.72  (-0.12, 1.60) | -0.79  (-1.35, -0.24) | -0.90  (-1.58, -0.24) | -0.94  (-1.60, -0.26) | -0.53  (-1.02, -0.05) | -0.83  (-1.85, 0.09) | -0.18  (-0.62, 0.27) | -0.57  (-1.11, -0.05) | -0.30  (-1.39, 0.80) | -0.89  (-1.41, -0.42) | 0.05  (-0.56, 0.64) | -0.28  (-0.72, 0.13) | Thiazolidinedione |  |
| 1.24  (0.06, 2.54) | 0.12  (-1.28, 1.56) | 1.05  (-0.01, 2.28) | 0.69  (-0.38, 1.87) | 0.74  (-0.31, 1.92) | 0.68  (-0.39, 1.90) | 1.40  (-0.26, 3.17) | 0.64  (-0.46, 1.86) | 1.34  (0.06, 2.74) | 1.90  (0.62, 3.22) | 0.38  (-0.71, 1.62) | 0.27  (-0.84, 1.50) | 0.23  (-0.84, 1.50) | 0.65  (-0.51, 1.88) | 0.35  (-0.98, 1.66) | 0.99  (0.03, 2.15) | 0.59  (-0.45, 1.78) | 0.88  (-0.52, 2.39) | 0.27  (-0.79, 1.45) | 1.23  (-0.14, 2.42) | 0.88  (-0.18, 2.06) | 1.18  (0.08, 2.38) | Vildagliptin |

The columns present the row anti-diabetic agents compared to the column anti-diabetic agents. The rows present the row drug class compared to the column individual anti-diabetic agents. The effect estimates are expressed as odds ratio and 95% confidence intervals. For example, the odd ratio for upper extremity fracture with Albiglutide compared to Alogliptin is 1.13 (95% confidence interval 0.03 to 2.36).

Table S28. Network estimated effect sizes (95% confidence interval) for Upper Extremity Fracture_Treatment Type

| DPP4i |  |  |  |  |  |  |  |
| --- | --- | --- | --- | --- | --- | --- | --- |
| -0.25 (-0.55, 0.06) | GLP1RA |  |  |  |  |  |  |
| -1.06 (-1.68, -0.47) | -0.81 (-1.38, -0.28) | Insulin |  |  |  |  |  |
| -0.23 (-0.73, 0.26) | 0.02 (-0.53, 0.53) | 0.84 (0.08, 1.56) | Metformin |  |  |  |  |
| -0.61 (-0.83, -0.39) | -0.36 (-0.61, -0.13) | 0.44 (-0.09, 1.03) | -0.38 (-0.87, 0.12) | Placebo |  |  |  |
| -0.49 (-0.75, -0.22) | -0.24 (-0.53, 0.05) | 0.57 (0.00, 1.19) | -0.25 (-0.78, 0.26) | 0.13 (-0.06, 0.30) | SGLT2i |  |  |
| -0.48 (-0.78, -0.19) | -0.24 (-0.58, 0.10) | 0.57 (-0.03, 1.21) | -0.26 (-0.76, 0.26) | 0.13 (-0.18, 0.42) | 0.00 (-0.32, 0.32) | Sulfonylurea |  |
| -0.79 (-1.23, -0.35) | -0.53 (-1.02, -0.06) | 0.27 (-0.44, 1.00) | -0.56 (-1.04, -0.09) | -0.17 (-0.61, 0.26) | -0.30 (-0.77, 0.15) | -0.30 (-0.72, 0.12) | Thiazolidinedione |

The columns present the row treatment type compared to the column treatment type. The rows present the row drug class compared to the column treatment type. The effect estimates are expressed as odds ratio and 95% confidence intervals. For example, the odd ratio for upper extremity fracture with DPP4i compared to GLP1RA is -0.25 (95% confidence interval -0.55 to 0.06).

Table S29. Network estimated effect sizes (95% confidence interval) for Vertebral Column Fracture

| Albiglutide |  |  |  |  |  |  |  |  |  |  |  |  |  |  |  |  |  |  |  |  |  |  |  |
| --- | --- | --- | --- | --- | --- | --- | --- | --- | --- | --- | --- | --- | --- | --- | --- | --- | --- | --- | --- | --- | --- | --- | --- |
| 0.14  (-2.73, 3.48) | Alogliptin |  |  |  |  |  |  |  |  |  |  |  |  |  |  |  |  |  |  |  |  |  |  |
| -0.88  (-2.39, 0.50) | -1.02  (-4.44, 1.77) | Canagliflozin |  |  |  |  |  |  |  |  |  |  |  |  |  |  |  |  |  |  |  |  |  |
| -0.37  (-1.84, 0.93) | -0.52  (-3.86, 2.10 | 0.51  (-0.57, 1.58) | Dapagliflozin |  |  |  |  |  |  |  |  |  |  |  |  |  |  |  |  |  |  |  |  |
| -0.70  (-2.10, 0.61) | -0.84  (-4.18, 1.92) | 0.18  (-0.92, 1.24) | -0.32  (-1.27, 0.64) | Dulaglutide |  |  |  |  |  |  |  |  |  |  |  |  |  |  |  |  |  |  |  |
| -0.66  (-2.53, 1.13) | -0.82  (-4.36, 2.11) | 0.21  (-1.38, 1.84 | -0.30  (-1.74, 1.27) | 0.03  (-1.52, 1.62) | Empagliflozin |  |  |  |  |  |  |  |  |  |  |  |  |  |  |  |  |  |  |
| -0.83  (-2.31, 0.53) | -0.96  (-4.33, 1.76) | 0.06  (-1.09, 1.16) | -0.45  (-1.47, 0.56) | -0.12  (-1.19, 0.90) | -0.15  (-1.77, 1.37) | Ertugliflozin |  |  |  |  |  |  |  |  |  |  |  |  |  |  |  |  |  |
| 0.17  (-2.83, 3.56) | 0.03  (-4.10, 4.28) | 1.06  (-1.83, 4.42) | 0.55  (-2.28, 3.88) | 0.87  (-1.94, 4.16) | 0.85  (-2.30, 4.38) | 0.99  (-1.89, 4.40) | Evogliptin |  |  |  |  |  |  |  |  |  |  |  |  |  |  |  |  |
| -0.65  (-4.69, 3.07) | -0.83  (-5.72, 3.65) | 0.25  (-3.74, 3.98) | -0.28  (-4.18, 3.39) | 0.06  (-3.77, 3.69) | 0.01  (-4.07, 3.82) | 0.16  (-3.68, 3.93) | -0.85  (-5.90, 3.83) | Exenatide |  |  |  |  |  |  |  |  |  |  |  |  |  |  |  |
| -0.69  (-2.87, 1.60) | -0.82  (-4.59, 2.36) | 0.19  (-1.79, 2.28) | -0.32  (-2.26, 1.72) | 0.02  (-1.79. 1.86) | -0.01  (-2.32, 2.33) | 0.14  (-1.85, 2.24) | -0.88  (-4.64, 2.46) | -0.052  (-4.02, 4.19) | Insulin |  |  |  |  |  |  |  |  |  |  |  |  |  |  |
| -0.58  (-2.07, 0.85) | -0.71  (-4.08, 2.03) | 0.30  (-0.92, 1.56) | -0.20  (-1.31, 0.94) | 0.13  (-0.99, 1.27) | 0.10  (-1.58, 1.71) | 0.24  (-0.93, 1.51) | -0.72  (-4.26, 2.17) | 0.08  (-3.66, 4.09) | 0.11  (-2.03, 2.24) | Linagliptin |  |  |  |  |  |  |  |  |  |  |  |  |  |
| -0.55  (-2.36, 1.21) | -0.69  (-4.11, 2.19) | 0.33  (-1.17, 1.88) | -0.17  (-1.64, 1.29) | 0.14  (-1.25, 1.55) | 0.12  (-1.79, 1.98) | 0.27  (-1.21, 1.82) | -0.73  (-4.33, 2.29) | 0.09  (-3.34, 3.70) | 0.14  (-1.97, 2.07) | 0.03  (-1.55, 1.61) | Liraglutide |  |  |  |  |  |  |  |  |  |  |  |  |
| -0.49  (-2.36, 1.27) | -0.61  (-3.96, 2.30) | 0.40  (-1.28, 2.02) | -0.08  (-1.69, 1.43) | 0.24  (-1.34, 1.75) | 0.20  (-1.76, 2.11) | 037  (-1.24, 1.88) | -0.64  (-4.39, 2.49) | 0.18  (-3.67, 4.31) | 0.21  (-2.15, 2.52) | 0.14  (-1.62, 1.71) | 0.08  (-1.89, 1.92) | Lixisenatide |  |  |  |  |  |  |  |  |  |  |  |
| -1.29  (-3.08, 0.33) | -1.45  (-4.96, 1.53) | -0.41  (-1.91, 1.08) | -0.91  (-2.36, 0.47) | -0.59  (-2.04, 0.85) | -0.61  (-2.54, 1.16) | -0.46  (-1.91, 0.98) | -1.48  (-4.95, 1.50) | -0.65  (-4.49, 3.44) | -0.62  (-2.96, 1.62) | -0.72  (-2.06, 0.64) | -0.73  (-2.59, 1.10) | -0.84  (2.69, 1.03) | Metformin |  |  |  |  |  |  |  |  |  |  |
| -1.69  (-5.29, 0.99) | -1.94  (-6.45, 1.89) | -0.82  (-4.47, 1.79) | -1.31  (-4.84, 1.25) | -0.99  (-4.51, 1.57) | -1.03  (-4.75, 1.77) | -0.87  (-4.40, 1.73) | -1.93  (-6.68, 1.79) | -1.15  (-6.00, 3.58) | -1.03  (-4.74, 2.06) | -1.12  (-4.61, 1.53) | -1.15  (-4.78, 1.59) | -1.24  (-4.79,1.66) | -0.41  (-4.07, 2.32) | Omarigliptin |  |  |  |  |  |  |  |  |  |
| -0.78  (-2.02, 0.32) | -0.92  (-4.23, 1.71) | 0.09  (-0.73, 0.93) | -0.41  (-1.05, 0.25) | -0.08  (-0.77, 0.60) | -0.11  (-1.54, 1.22) | 0.04  (-0.70, 0.85) | -0.94  (-4.21, 1.82) | -0.12  (-3.74, 3.66) | -0.08  (-1.94, 1.76) | -0.20  (-1.16, 0.72) | -0.23  (-1.55, 1.04) | -0.33  (-1.64, 1.11) | .515  (-0.72, 1.82) | 0.89  (-1.53, 4.38) | Placebo |  |  |  |  |  |  |  |  |
| -0.44  (-2.03, 1.09) | -0.58  (-3.90, 2.24) | 0.45  (-0.83, 1.76) | -0.07  (-1.23, 1.15) | 0.27  (-0.93, 1.47) | 0.24  (-1.44, 1.97) | 0.39  (-0.80, 1.70) | -0.59  (-4.11, 2.31) | 0.23  (-3.56, 4.14) | 0.26  (-1.91, 2.38) | 0.15  (-1.10, 1.40) | 0.10  (-1.51, 1.73) | 0.03  (-1.62, 1.77) | 0.86  (-0.65, 2.44) | 1.25  (-1.42, 4.81) | 0.35  (-0.62, 1.37) | Saxagliptin |  |  |  |  |  |  |  |
| 0.29  (-1.52, 2.15) | 0.15  (-3.42, 3.15) | 1.15  (-0.41, 2.90) | 0.66  (-0.78, 2.34) | 0.98  (-0.48, 2.61) | 0.95  (-0.99, 2.96) | 1.10  (-0.40, 2.76) | 0.10  (-3.29, 3.28) | 0.95  (-2.94, 4.92) | 0.99  (-1.40, 3.27) | 0.85  (-0.78, 2.62) | 0.83  (-0.99, 2.86) | 0.75  (-1.13, 2.85) | 1.57  (-0.28, 3.58) | 2.02  (-0.86, 5.86) | 1.05  (-0.21, 2.60) | 0.72  (-0.97, 2.52) | Semaglutide |  |  |  |  |  |  |
| -0.73  (-2.20, 0.64) | -0.87  (-4.31, 1.89) | 0.14  (-0.93, 1.32) | -0.36  (-1.42, 0.67) | -0.04  (-1.06, 1.04) | -0.05  (-1.70, 1.52) | 0.09  (-1.00, 1.24) | -0.89  (-4.16, 1.73) | -0.11  (-3.82, 3.89) | -0.05  (-2.10, 1.96) | -0.15  (-1.38, 1.04) | -0.19  (-1.75, 1.32) | -0.27  (-1.83, 1.37) | 0.54  (-0.78, 1.95) | 0.95  (-1.61, 4.52) | 0.05  (-0.78, 0.87) | -0.29  (-1.63, 0.98) | -1.00  (-2.74, 0.56) | Sitagliptin |  |  |  |  |  |
| -0.63  (-2.26, 0.93) | -0.73  (-4.25, 2.11) | 0.26  (-1.13, 1.65) | -0.24  (-1.52, 1.05) | 0.09  (-1.20, 1.41) | 0.06  (-1.79, 1.78) | 0.21  (-1.15, 1.61) | -0.76  (-4.22, 2.22) | -0.01  (-3.78, 4.04) | 0.10  (-2.13, 2.15) | -0.03  (-1.50, 1.42) | -0.06  (-1.77, 1.66) | -0.16  (-1.92, 1.72) | 0.66  (-0.99, 2.46) | 1.08  (-1.58, 4.68) | 0.16  (-0.96, 1.32) | -0.18  (-1.69, 1.32) | -0.90  (-2.76, 0.83) | 0.12  (-1.29, 1.52) | Sotagliflozin |  |  |  |  |
| -0.26  (-1.87, 1.30) | -0.41  (-3.84, 2.44) | 0.61  (-0.72, 2.01) | 0.11  (-1.10, 1.43) | 0.44  (-0.83, 1.69) | 0.41  (-1.34, 2.15) | 0.56  (-0.74, 1.95) | -0.42  (-4.04, 2.55) | 0.40  (-3.31, 4.33) | 0.42  (-1.68, 2.55) | 0.31  (-0.45, 1.08) | 0.28  (-1.33, 1.99) | 0.21  (-1.51, 2.00) | 1.03  (-0.45, 2.52) | 1.42  (-1.29, 4.96) | 0.52  (-0.53, 1.63) | 0.17  (-1.10, 1.49) | -0.55  (-2.42, 1.19) | 0.46  (-0.84, 1.82) | 0.36  (-1.20, 1.89) | Sulfonylurea |  |  |  |
| -0.22  (-2.13, 1.66) | -0.37  (-3.89, 2.64) | 0.67  (-1.03, 2.43) | 0.20  (-1.46, 1.81) | 0.50  (-1.11, 2.16) | 0.46  (-1.58, 2.52) | 0.63  (-1.00, 2.34) | -0.35  (-4.03, 2.74) | 0.46  (-3.47, 4.63) | 0.49  (-1.86, 2.85) | 0.38  (-1.00, 1.77) | 0.36  (-1.67, 2.30) | 0.28  (-1.71, 2.33) | 1.11  (-0.71, 2.91) | 1.52  (-1.43, 5.13) | 0.60  (-0.91, 2.10) | 0.24  (-1.43, 1.86) | -0.48  (-2.65, 1.50) | 0.54  (-1.13, 2.22) | 0.43  (-1.45, 2.24) | 0.07  (-1.16, 1.29) | Thiazolidinedione |  |  |
| -0.94  (-3.89, 2.48) | -1.10  (-5.20, 2.96) | -0.06  (-2.80, 3.34) | -0.57  (-3.28, 2.79) | -0.24  (-2.93, 3.22) | -0.27  (-3.33, 3.40) | -0.12  (-2.80, 3.36) | -1.08  (-5.43, 3.05) | -0.24  (-4.79, 4.73) | -0.25  (-3.54, 3.48) | -0.34  (-3.17, 2.97) | -0.38  (-3.32, 3.13) | -0.45  (-3.48, 3.18) | 0.35  (-2.56, 3.95) | 0.80  (-2.97, 5.33) | -0.18  (-2.78, 3.17) | -0.53  (-3.32, 2.94) | -1.22  (-4.29, 2.37) | -0.20  (-3.01, 3.19) | -0.33  (-3.23, 3.20) | -0.68  (-3.54, 2.75) | -0.74  (-3.71, 2.89) | Tirzepatide |  |
| -0.80  (-2.83, 1.32) | -0.90  (-4.51, 2.18) | 0.07  (-1.80, 2.08) | -0.41  (-2.26, 1.50) | -0.09  (-1.97, 1.81) | -0.10  (-2.33, 2.10) | 0.06  (-1.83, 2.02) | -0.94  (-4.74, 2.24) | -0.14  (-4.12, 4.28) | -0.10  (-2.65, 2.41) | -0.19  (-2.19, 1.78) | -0.24  (-2.43. 1.92) | -0.31  (-2.54, 1.89) | 0.52  (-1.67, 2.72) | 0.95  (-2.13, 4.71) | -0.01  (-1.78, 1.83) | -0.36  (-2.32, 1.65) | -1.08  (-3.40, 1.19) | -0.04  (-1.95, 1.91) | -0.17  (-2.19, 1.93) | -0.50  (-2.61, 1.52) | -0.57  (-2.89, 1.66) | 0.15  (-3.67, 3.36) | Vildagliptin |

The columns present the row anti-diabetic agents compared to the column anti-diabetic agents. The rows present the row drug class compared to the column individual anti-diabetic agents. The effect estimates are expressed as odds ratio and 95% confidence intervals. For example, the odd ratio for vertebral column fracture with Albiglutide compared to Alogliptin is 0.14 (95% confidence interval -2.73 to 3.48).

Table S30. Network estimated effect sizes (95% confidence interval) for Vertebral Column Fracture_Treatment Type

| DPP4i |  |  |  |  |  |  |  |
| --- | --- | --- | --- | --- | --- | --- | --- |
| 0.25 (-0.39, 0.90) | GLP1RA |  |  |  |  |  |  |
| -1.49 (-3.16, -0.20) | -1.74 (-3.31, -0.58) | Insulin |  |  |  |  |  |
| -0.62 (-1.84, 0.57) | -0.88 (-2.20, 0.46) | 0.89 (-0.95, 2.91) | Metformin |  |  |  |  |
| -0.09 (-0.58, 0.37) | -0.35 (-0.81, 0.12) | 1.41 (0.17, 2.98) | 0.54 (-0.69, 1.76) | Placebo |  |  |  |
| 0.06 (-0.54, 0.65) | -0.20 (-0.82, 0.40) | 1.55 (0.25, 3.18) | 0.69 (-0.58, 1.95) | 0.15 (-0.23, 0.51) | SGLT2i |  |  |
| -0.85 (-1.45, -0.33) | -1.12 (-1.98, -0.28) | 0.63 (-0.77, 2.35) | -0.24 (-1.58, 1.11) | -0.77 (-1.49, -0.07) | -0.92 (-1.74, -0.12) | Sulfonylurea |  |
| -0.94 (-1.68, -0.19) | -1.20 (-2.17, -0.24) | 0.56 (-0.92, 2.30) | -0.33 (-1.74, 1.06) | -0.85 (-1.70, -0.03) | -1.00 (-1.94, -0.09) | -0.08 (-0.62, 0.47) | Thiazolidinedione |

The columns present the row treatment type compared to the column treatment type. The rows present the row drug class compared to the column treatment type. The effect estimates are expressed as odds ratio and 95% confidence intervals. For example, the odd ratio for vertebral column fracture with DPP4i compared to GLP1RA is 0.25 (95% confidence interval -0.39 to 0.90).

Table S31. Network estimated effect sizes (95% confidence interval) for Femoral Neck BMD

| Canagliflozin |  |  |  |  |  |  |
| --- | --- | --- | --- | --- | --- | --- |
| 1.00 (-0.23, 2.22) | Dapagliflozin |  |  |  |  |  |
| 0.46 (-0.56, 1.45) | -0.55 (-1.62, 0.53) | Ertugliflozin |  |  |  |  |
| 1.00 (0.17, 1.84) | -0.01 (-0.96, 0.90) | 0.54 (-0.04, 1.12) | Metformin |  |  |  |
| 0.40 (-0.43, 1.24) | -0.61 (-1.56, 0.30) | -0.06 (-0.64, 0.52) | -0.60 (-0.61, -0.59) | Placebo |  |  |
| -0.34 (-1.61, 0.96) | -1.34 (-2.68, -0.01) | -0.78 (-1.93, 0.34) | -1.33 (-2.32, -0.35) | -0.73 (-1.72, 0.25) | Sotagliflozin |  |
| 0.80 (-0.04, 1.65) | -0.21 (-1.16, 0.70) | 0.34 (-0.23, 0.92) | -0.20 (-0.26, -0.14) | 0.40 (0.34, 0.46) | 1.13 (0.15, 2.13) | Thiazolidinedione |

The columns present the row drug class compared to the column drug class. The rows present the row drug class compared to the column drug class. The effect estimates are expressed as mean difference and 95% confidence intervals. For example, the mean difference for femoral neck BMD with canagliflozin compared to dapagliflozin is 1.00 (95% confidence interval -0.23 to 2.22)

Table S32. Network estimated effect sizes (95% confidence interval) for Femoral Neck BMD_Treatment Type

| Metformin |  |  |  |
| --- | --- | --- | --- |
| -0.60 (-0.61, -0.59) | Placebo |  |  |
| -0.67 (-1.04, -0.28) | -0.07 (-0.44, 0.32) | SGLT-2 |  |
| -0.20 (-0.26, -0.14) | 0.40 (0.34, 0.46) | 0.47 (0.08, 0.85) | Thiazolidinedione |

The columns present the row drug class compared to the column drug class. The rows present the row drug class compared to the column drug class. The effect estimates are expressed as mean difference and 95% confidence intervals. For example, the mean difference for femoral neck BMD with metformin compared to placebo is -0.60 (95% confidence interval -0.61 to -0.59)

Table S33. Network estimated effect sizes (95% confidence interval) for Total Hip BMD

| Canagliflozin |  |  |  |  |  |  |
| --- | --- | --- | --- | --- | --- | --- |
| -0.72  (-2.17, 0.78) | Dapagliflozin |  |  |  |  |  |
| -0.75  (-1.45, -0.06) | -0.04  (-1.49, 1.41) | Ertugliflozin |  |  |  |  |
| -0.40  (-0.96, 0.16) | 0.32  (-1.11, 1.69) | 0.36  (-0.07, 0.77) | Metformin |  |  |  |
| -0.50  (-1.06, 0.06) | 0.22  (-1.21, 1.59) | 0.26  (-0.17, 0.67) | -0.10  (-0.11, -0.10) | Placebo |  |  |
| -0.65  (-1.51, 0.24) | 0.07  (-1.50, 1.61) | 0.11  (-0.68, 0.87) | -0.24  (-0.92, 0.40) | -0.14  (-0.82, 0.50) | Sotagliflozin |  |
| 0.50  (-0.07, 1.07) | 1.21  (-0.22, 2.62) | 1.25  (0.82, 1.69) | 0.90  (0.77, 1.02) | 1.00  (0.87, 1.12) | 1.13  (0.48, 1.80) | Thiazolidinedione |

The columns present the row drug class compared to the column drug class. The rows present the row drug class compared to the column drug class. The effect estimates are expressed as mean difference and 95% confidence intervals. For example, the mean difference for total hip BMD with canagliflozin compared to dapagliflozin is -0.72 (95% confidence interval -2.17 to 0.78)

Table S34. Network estimated effect sizes (95% confidence interval) for Total Hip BMD_Treatment Type

| GLP-1 |  |  |  |  |
| --- | --- | --- | --- | --- |
| 0.80 (0.78, 0.82) | Metformin |  |  |  |
| 0.70 (0.68, 0.72) | -0.10 (-0.11, -0.09) | Placebo |  |  |
| 0.68 (0.38, 0.96) | -0.12 (-0.42, 0.16) | -0.02 (-0.32, 0.26) | SGLT-2 |  |
| 1.69 (1.57, 1.82) | 0.89 (0.77, 1.02) | 0.99 (0.87, 1.12) | 1.02 (0.70, 1.34) | Thiazolidinedione |

The columns present the row drug class compared to the column drug class. The rows present the row drug class compared to the column drug class. The effect estimates are expressed as mean difference and 95% confidence intervals. For example, the mean difference for total hip BMD with GLP1 receptor agonists compared to metformin is 0.80 (95% confidence interval 0.78 to 0.82)

Table S35. Network estimated effect sizes (95% confidence interval) for Lumber spine BMD

| Canagliflozin |  |  |  |  |  |  |
| --- | --- | --- | --- | --- | --- | --- |
| -0.41 (-1.67, 0.85) | Dapagliflozin |  |  |  |  |  |
| -0.24 (-1.25, 0.76) | 0.16 (-0.93, 1.28) | Ertuglilozin |  |  |  |  |
| -0.48 (-1.32, 0.32) | -0.09 (-1.02, 0.83) | -0.24 (-0.84, 0.34) | Metformin |  |  |  |
| -0.29 (-1.13, 0.50) | 0.10 (-0.83, 1.02) | -0.05 (-0.65, 0.54) | 0.19 (0.18, 0.20) | Placebo |  |  |
| -0.06 (-1.29, 1.17) | 0.33 (-0.98, 1.64) | 0.20 (-0.91, 1.25) | 0.44 (-0.47, 1.32) | 0.25 (-0.66, 1.13) | Sotagliflozin |  |
| 0.81 (-0.05, 1.61) | 1.20 (0.26, 2.14) | 1.05 (0.45, 1.63) | 1.29 (1.21, 1.37) | 1.10 (1.02, 1.18) | 0.85 (-0.04, 1.76) | Thiazolidinedione |

The columns present the row drug class compared to the column drug class. The rows present the row drug class compared to the column drug class. The effect estimates are expressed as mean difference and 95% confidence intervals. For example, the mean difference for lumber spine BMD with canagliflozin compared to dapagliflozin is -0.41 (95% confidence interval -1.67 to 0.85)

Table S36. Network estimated effect sizes (95% confidence interval) for Lumber Spine BMD_Treatment Type

| Metformin |  |  |  |
| --- | --- | --- | --- |
| 0.19 (0.18, 0.20) | Placebo |  |  |
| 0.30 (-0.10, 0.69) | 0.11 (-0.29, 0.50) | SGLT-2 |  |
| 1.28 (1.20, 1.36) | 1.09 (1.02, 1.17) | 0.98 (0.58, 1.39) | Thiazolidinedione |

The columns present the row drug class compared to the column drug class. The rows present the row drug class compared to the column drug class. The effect estimates are expressed as mean difference and 95% confidence intervals. For example, the mean difference for lumber spine BMD with metformin compared to placebo is 0.19 (95% confidence interval 0.18 to 0.20)

Table S37. Subgroup analysis of osteoporosis parameters by treatment type based on 52 weeks follow-up duration

|  | Group | Measures | GLP1RA | | SGLT2i | | DPP4i | | Thiazolidinedione | | | |
| --- | --- | --- | --- | --- | --- | --- | --- | --- | --- | --- | --- | --- |
|  |  |  | <52 weeks | ≥ 52 weeks | <52 weeks | ≥ 52 weeks | <52 weeks | ≥ 52 weeks | <52 weeks | | ≥ 52 weeks | |
|  | Vertebral Fracture | Effect [95%CI] | 0.67 [0.06, 7.39] | 0.64 [0.39, 1.06] | 0.80 [0.21, 3.04] | 0.82 [0.47, 1.44] | 1.01 [0.16, 6.24] | 0.97 [0.56, 1.71] | Within only one study | | | |
|  |  | n studies | 1 | 9 | 4 | 10 | 2 | 8 |  |  |  |  |
|  |  | $I^{2}$ | NA | 0 | 0 | 0 | 0 | 0 |  |  |  |  |
|  |  | Subgroup difference | 0.98 | | 0.97 | | 0.97 | |  |  |  |  |
|  |  | Overall P | 0.08 | | 0.44 | | 0.93 | |  |  |  |  |
|  | Non-Vertebral Fracture | Effect [95%CI] | 0.66 [0.35, 1.27] | 0.85 [0.68, 1.05] | 0.73 [0.43, 1.26] | 1.07 [0.88, 1.31] | 0.71 [0.41, 1.22] | 1.00 [0.80, 1.24] | 0.97 [0.13, 7.51] | | 1.28 [0.23, 7.10] | |
|  |  | n studies | 9 | 9 | 15 | 17 | 17 | 12 | 2 | | 2 | |
|  |  | $I^{2}$ | 0 | 0 | 0 | 0 | 0 | 0 | 63 | | 0 | |
|  |  | Subgroup difference | 0.49 | | 0.20 | | 0.25 | | 0.82 | | | |
|  |  | Overall P | 0.07 | | 0.80 | | 0.64 | | 0.86 | | | |
|  | Hip Fracture | Effect [95%CI] | 1.47 [0.24, 9.10] | 0.88 [0.66, 1.18] | 0.84 [0.27, 2.61] | 0.91 [0.71, 1.17] | 0.92 [0.41, 2.07] | 1.02 [0.75, 1.38] | Within only one study | | | |
|  |  | n studies | 2 | 7 | 5 | 12 | 9 | 9 |  |  |  |  |
|  |  | $I^{2}$ | 0 | 0 | 0 | 0 | 0 | 0 |  |  |  |  |
|  |  | Subgroup difference | 0.58 | | 0.89 | | 0.83 | |  |  |  |  |
|  |  | Overall P | 0.43 | | 0.44 | | 0.97 | |  |  |  |  |
| Vertebral + Hip Fracture | | Effect [95%CI] | 1.10 [0.26, 4.71] | 0.82 [0.64, 1.05] | 0.84 [0.37, 1.92] | 0.90 [0.73, 1.11] | 0.94 [0.46, 1.93] | 0.98 [0.75, 1.28] | - | | 0.67 [0.13, 3.49] | |
|  |  | n studies | 3 | 9 | 7 | 12 | 10 | 12 | - | | 2 | |
|  |  | $I^{2}$ | 0 | 0 | 0 | 0 | 0 | 0 | - | | 0 | |
|  |  | Subgroup difference | 0.69 | | 0.87 | | 0.93 | | NA | | | |
|  |  | Overall P | 0.12 | | 0.29 | | 0.84 | | 0.64 | | | |
|  | Multiple Fracture | Effect [95%CI] | 1.00 [0.09,11.08] | 1.26 [0.32,5.05] | - | 0.83 [0.24,2.85] | 0.66 [0.11,4.09] | 0.71 [0.22,2.25] | - | | | |
|  |  | n studies | 1 | 2 | - | 4 | 2 | 2 |  |  |  |  |
|  |  | $I^{2}$ | NA | 0 | - | 0 | 0 | 0 |  |  |  |  |
|  |  | Subgroup difference | 0.87 | | NA | | 0.95 | |  |  |  |  |
|  |  | Overall P | 0.78 | | 0.76 | | 0.47 | |  |  |  |  |
|  | Overall Fracture | Effect [95%CI] | 0.97 [0.57,1.67] | 0.81 [0.64,1.02] | 1.00 [0.85,1.16] | 0.78 [0.50,1.22] | 0.78 [0.54,1.14] | 0.91 [0.72,1.15] | 1.68 [0.56,5.06] | | 1.13 [0.22,5.87] | |
|  |  | n studies | 17 | 13 | 20 | 28 | 38 | 13 | 4 | | 3 | |
|  |  | $I^{2}$ | 0 | 0 | 0 | 0 | 0 | 0 | 0 | | 50 | |
|  |  | Subgroup difference | 0.53 | | 0.31 | | 0.52 | | 0.69 | | | |
|  |  | Overall P | 0.08 | | 0.64 | | 0.17 | | 0.63 | | | |
| Vertebral Column Fracture | | Effect [95%CI] | 0.67 [0.06, 7.39] | 0.64 [0.39, 1.06] | 0.80 [0.21, 3.04] | 0.82 [0.47, 1.44] | 1.01 [0.16, 6.24] | 0.97 [0.56, 1.71] | Within only one study | | |  |
|  |  | n studies | 1 | 9 | 4 | 10 | 2 | 8 |  |  |  |  |
|  |  | $I^{2}$ | NA | 0 | 0 | 0 | 0 | 0 |  |  |  |  |
|  |  | Subgroup difference | 0.98 | | 0.97 | | 0.97 | |  |  |  |  |
|  |  | Overall P | 0.08 | | 0.44 | | 0.93 | |  |  |  |  |
| Lower Extremity Fracture | | Effect [95%CI] | 0.77 [0.32, 1.89] | 0.85 [0.67, 1.07] | 0.82 [0.47, 1.44] | 1.04 [0.86, 1.25] | 0.79 [0.49, 1.27] | 1.08 [0.84, 1.40] | 1.26 [0.25, 6.47] | 0.83 [0.07, 9.23] | |  |
|  |  | n studies | 5 | 8 | 14 | 17 | 23 | 13 | 2 | 1 | |  |
|  |  | $I^{2}$ | 0 | 0 | 0 | 0 | 0 | 0 | 37 | NA | |  |
|  |  | Subgroup difference | 0.84 | | 0.45 | | 0.25 | | 0.78 | | |  |
|  |  | Overall P | 0.14 | | 0.89 | | 0.96 | | 0.76 | | |  |
| Upper Extremity Fracture | | Effect [95%CI] | 0.70 [0.30, 1.68] | 0.85 [0.62, 1.15] | 0.62 [0.23, 1.66] | 1.01 [0.78, 1.32] | 0.57 [0.23, 1.42] | 0.90 [0.62, 1.30] | 0.25 [0.02, 2.80] | 2.00 [0.18, 22.71] | |  |
|  |  | n studies | 7 | 8 | 5 | 13 | 6 | 8 | 1 | 1 | |  |
|  |  | $I^{2}$ | 0 | 0 | 0 | 0 | 0 | 0 | NA | NA | |  |
|  |  | Subgroup difference | 0.69 | | 0.35 | | 0.36 | | 0.24 | | |  |
|  |  | Overall P | 0.21 | | 0.86 | | 0.33 | | 0.69 | | |  |
| Pelvic Girdle Fracture | | Effect [95%CI] | 1.47 [0.24, 9.09] | 0.99 [0.56, 1.76] | 0.66 [0.11, 4.10] | 0.83 [0.56, 1.23] | 1.99 [0.18, 22.03] | 0.92 [0.57, 1.47] | Within only one study | | |  |
|  |  | n studies | 2 | 5 | 2 | 9 | 1 | 6 |  |  |  |  |
|  |  | $I^{2}$ | 0 | 30 | 0 | 0 | NA | 0 |  |  |  |  |
|  |  | Subgroup difference | 0.69 | | 0.81 | | 0.54 | |  |  |  |  |
|  |  | Overall P | 0.96 | | 0.32 | | 0.81 | |  |  |  |  |
| Cranial and Facial bone Fracture | | Effect [95%CI] | 1.15 [0.21, 6.31] | 0.78 [0.34, 1.78] | 1.88 [0.17, 20.88] | 1.50 [0.80, 2.82] | 0.50 [0.09, 2.74] | 0.84 [0.44, 1.63] | Within only one study | | |  |
|  |  | n studies | 2 | 7 | 1 | 7 | 2 | 4 |  |  |  |  |
|  |  | $I^{2}$ | 0 | 0 | NA | 0 | 0 | 0 |  |  |  |  |
|  |  | Subgroup difference | 0.69 | | 0.86 | | 0.57 | |  |  |  |  |
|  |  | Overall P | 0.65 | | 0.18 | | 0.45 | |  |  |  |  |
| Thoracic Cage Fracture | | Effect [95%CI] | 0.58 [0.19,1.82] | 0.78 [0.45,1.38] | 0.51 [0.05,5.69] | 1.10 [0.71,1.70] | 0.79 [0.33,1.89] | 0.78 [0.41,1.49] | - | 0.54 [0.06,4.96] | |  |
|  |  | n studies | 4 | 7 | 1 | 9 | 8 | 7 | - | 2 | |  |
|  |  | $I^{2}$ | 0 | 0 | NA | 0 | 0 | 0 | - | 56 | |  |
|  |  | Subgroup difference | 0.65 | | 0.54 | | 0.97 | | NA | | |  |
|  |  | Overall P | 0.24 | | 0.74 | | 0.36 | | 0.24 | | |  |

Table S38. Subgroup analysis of osteoporosis parameters by treatment type based on 78 weeks follow-up duration

| Group | Measures | GLP1RA | | SGLT2i | | DPP4i | | Thiazolidinedione | |  |
| --- | --- | --- | --- | --- | --- | --- | --- | --- | --- | --- |
|  |  | <78 weeks | ≥ 78 weeks | <78 weeks | ≥ 78 weeks | <78 weeks | ≥ 78 weeks | <78 weeks | ≥ 78 weeks |  |
| Vertebral Fracture | Effect [95%CI] | 0.99 [0.18, 5.41] | 0.62 [0.37, 1.03] | 1.13 [0.52, 2.43] | 0.62 [0.31, 1.25] | 0.72 [0.20, 2.60] | 1.04 [0.58, 1.89] | Within only one study | |  |
|  | n studies | 2 | 8 | 8 | 6 | 4 | 6 |  |  |  |
|  | $I^{2}$ | 0 | 0 | 0 | 0 | 0 | 0 |  |  |  |
|  | Subgroup difference | 0.61 | | 0.26 | | 0.60 | |  |  |  |
|  | Overall P | 0.08 | | 0.44 | | 0.93 | |  |  |  |
| Non-Vertebral Fracture | Effect [95%CI] | 0.66 [0.37, 1.17] | 0.85 [0.69, 1.06] | 0.85 [0.41, 1.24] | 1.01 [0.76, 1.11] | 0.73 [0.45, 1.19] | 1.01 [0.81, 1.26] | 1.17 [0.29, 4.74] | 0.83 [0.07, 9.23] |  |
|  | n studies | 11 | 7 | 27 | 6 | 20 | 9 | 3 | 1 |  |
|  | $I^{2}$ | 0 | 0 | 0 | 0 | 0 | 0 | 33 | NA |  |
|  | Subgroup difference | 0.42 | | 0.63 | | 0.24 | | 0.81 | |  |
|  | Overall P | 0.07 | | 0.80 | | 0.64 | | 0.86 | |  |
| Hip Fracture | Effect [95%CI] | 1.47 [0.24, 9.10] | 0.88 [0.66, 1.18] | 1.02 [0.58, 1.78] | 0.89 [0.67, 1.16] | 1.06 [0.51, 2.20] | 1.00 [0.73, 1.36] | Within only one study | |  |
|  | n studies | 2 | 7 | 11 | 6 | 11 | 7 |  |  |  |
|  | $I^{2}$ | 0 | 0 | 0 | 0 | 0 | 0 |  |  |  |
|  | Subgroup difference | 0.58 | | 0.67 | | 0.87 | |  |  |  |
|  | Overall P | 0.43 | | 0.44 | | 0.97 | |  |  |  |
| Vertebral + Hip Fracture | Effect [95%CI] | 1.19 [0.34, 4.12] | 0.81 [0.63, 1.04] | 1.05 [0.67, 1.64] | 0.86 [0.68, 1.08] | 0.97 [0.52, 1.80] | 0.98 [0.74, 1.28] | - | 0.67 [0.13, 3.49] |  |
|  | n studies | 4 | 8 | 13 | 6 | 13 | 8 | - | 2 |  |
|  | $I^{2}$ | 0 | 0 | 0 | 0 | 0 | 0 | - | 0 |  |
|  | Subgroup difference | 0.56 | | 0.42 | | 0.98 | | NA | |  |
|  | Overall P | 0.12 | | 0.29 | | 0.84 | | 0.64 | |  |
| Multiple Fracture | Effect [95%CI] | 1.00 [0.09,11.08] | 1.26 [0.32,5.05] | 0.97 [0.06,15.66] | 0.79 [0.20,3.17] | 0.66 [0.11,4.09] | 0.71 [0.22,2.25] | - | |  |
|  | n studies | 1 | 2 | 1 | 3 | 2 | 2 |  |  |  |
|  | $I^{2}$ | NA | 0 | NA | 0 | 0 | 0 |  |  |  |
|  | Subgroup difference | 0.87 | | 0.90 | | 0.95 | |  |  |  |
|  | Overall P | 0.78 | | 0.76 | | 0.47 | |  |  |  |
| Overall Fracture | Effect [95%CI] | 1.00 [0.62,1.61] | 0.79 [0.61,1.02] | 0.81 [0.60,1.10] | 1.03 [0.85,1.25] | 0.83 [0.60,1.16] | 0.89 [0.71,1.14] | 1.88 [0.70,5.08] | 0.78 [0.09,6.81] |  |
|  | n studies | 22 | 8 | 41 | 7 | 47 | 6 | 5 | 2 |  |
|  | $I^{2}$ | 0 | 2 | 0 | 8 | 0 | 7 | 0 | 60 |  |
|  | Subgroup difference | 0.40 | | 0.19 | | 0.73 | | 0.47 | |  |
|  | Overall P | 0.08 | | 0.64 | | 0.17 | | 0.63 | |  |
| Vertebral Column Fracture | Effect [95%CI] | 0.99 [0.18, 5.41] | 0.62 [0.37, 1.03] | 1.13 [0.52, 2.43] | 0.62 [0.31, 1.25] | 0.72 [0.20, 2.60] | 1.04 [0.58, 1.89] | Within only one study | | |
|  | n studies | 2 | 8 | 8 | 6 | 4 | 6 |  |  |  |
|  | $I^{2}$ | 0 | 0 | 0 | 0 | 0 | 0 |  |  |  |
|  | Subgroup difference | 0.61 | | 0.26 | | 0.60 | |  |  |  |
|  | Overall P | 0.08 | | 0.44 | | 0.93 | |  |  |  |
| Lower Extremity Fracture | Effect [95%CI] | 0.72 [0.32, 1.62] | 0.86 [0.68, 1.08] | 1.15 [0.81, 1.62] | 0.97 [0.79, 1.19] | 0.84 [0.56, 1.28] | 1.07 [0.80, 1.42] | 1.26 [0.25, 6.47] | 0.83 [0.07, 9.23] | |
|  | n studies | 6 | 7 | 24 | 7 | 28 | 8 | 2 | 1 | |
|  | $I^{2}$ | 0 | 0 | 0 | 3 | 0 | 18 | 37 | NA | |
|  | Subgroup difference | 0.68 | | 0.41 | | 0.36 | | 0.78 | | |
|  | Overall P | 0.14 | | 0.89 | | 0.96 | | 0.76 | | |
| Upper Extremity Fracture | Effect [95%CI] | 0.72 [0.34, 1.53] | 0.85 [0.62, 1.16] | 1.02 [0.61, 1.71] | 0.98 [0.71, 1.36] | 0.66 [0.28, 1.56] | 0.88 [0.61, 1.28] | 0.71 [0.09, 5.37] | - | |
|  | n studies | 8 | 7 | 12 | 6 | 7 | 7 | 2 | - | |
|  | $I^{2}$ | 0 | 0 | 0 | 24 | 0 | 0 | 29 | - | |
|  | Subgroup difference | 0.69 | | 0.89 | | 0.54 | | NA | | |
|  | Overall P | 0.21 | | 0.86 | | 0.33 | | 0.69 | | |
| Pelvic Girdle Fracture | Effect [95%CI] | 1.47 [0.24, 9.09] | 0.99 [0.56, 1.76] | 1.02 [0.43, 2.42] | 0.78 [0.51, 1.20] | 1.99 [0.18, 22.03] | 0.92 [0.57, 1.47] | Within only one study | | |
|  | n studies | 2 | 5 | 5 | 6 | 1 | 6 |  |  |  |
|  | $I^{2}$ | 0 | 30 | 0 | 0 | NA | 0 |  |  |  |
|  | Subgroup difference | 0.69 | | 0.59 | | 0.54 | |  |  |  |
|  | Overall P | 0.96 | | 0.32 | | 0.81 | |  |  |  |
| Cranial and Facial bones Fracture | Effect [95%CI] | 1.07 [0.31, 3.71] | 0.74 [0.29, 1.85] | 1.46 [0.41, 5.22] | 1.54 [0.77, 3.09] | 0.50 [0.09, 2.74] | 0.84 [0.44, 1.63] | Within only one study | | |
|  | n studies | 4 | 5 | 3 | 5 | 2 | 4 |  |  |  |
|  | $I^{2}$ | 0 | 0 | 0 | 0 | 0 | 0 |  |  |  |
|  | Subgroup difference | 0.63 | | 0.94 | | 0.57 | |  |  |  |
|  | Overall P | 0.65 | | 0.18 | | 0.45 | |  |  |  |
| Thoracic Cage Fracture | Effect [95%CI] | 0.73 [0.26,2.05] | 0.74 [0.41,1.32] | 0.97 [0.36,2.59] | 1.12 [0.66,1.91] | 0.88 [0.39,1.99] | 0.72 [0.37,1.42] | 2.00 [0.18,22.72] | 0.20 [0.04,1.12] | |
|  | n studies | 5 | 6 | 5 | 5 | 9 | 6 | 1 | 1 | |
|  | $I^{2}$ | 0 | 0 | 0 | 29 | 0 | 0 | NA | NA | |
|  | Subgroup difference | 0.98 | | 0.80 | | 0.71 | | 0.13 | | |
|  | Overall P | 0.24 | | 0.74 | | 0.36 | | 0.24 | | |

Table S39. Subgroup analysis of osteoporosis parameters by treatment type based on 104 weeks follow-up duration

| Group | Measures | GLP1RA | | SGLT2i | | DPP4i | | Thiazolidinedione | |  |
| --- | --- | --- | --- | --- | --- | --- | --- | --- | --- | --- |
|  |  | <104 weeks | ≥ 104 weeks | <104 weeks | ≥ 104 weeks | <104 weeks | ≥ 104 weeks | <104 weeks | ≥ 104 weeks |  |
| Vertebral Fracture | Effect [95%CI] | 0.67 [0.17, 2.60] | 0.64 [0.38, 1.08] | 1.13 [0.52, 2.43] | 0.62 [0.31, 1.25] | 0.72 [0.20, 2.60] | 1.04 [0.58, 1.89] | Within only one study | |  |
|  | n studies | 3 | 7 | 8 | 6 | 4 | 6 |  |  |  |
|  | $I^{2}$ | 0 | 0 | 0 | 0 | 0 | 0 |  |  |  |
|  | Subgroup difference | 0.95 | | 0.26 | | 0.60 | |  |  |  |
|  | Overall P | 0.08 | | 0.44 | | 0.93 | |  |  |  |
| Non-Vertebral Fracture | Effect [95%CI] | 0.73 [0.45, 1.17] | 0.85 [0.68, 1.07] | 1.01 [0.74, 1.17] | 0.98 [0.20, 1.41] | 0.76 [0.48, 1.22] | 1.00 [0.80, 1.25] | 1.17 [0.29, 4.74] | 0.83 [0.07, 9.23] |  |
|  | n studies | 12 | 6 | 27 | 6 | 21 | 8 | 3 | 1 |  |
|  | $I^{2}$ | 0 | 0 | 0 | 0 | 0 | 0 | 33 | NA |  |
|  | Subgroup difference | 0.57 | | 0.21 | | 0.64 | | 0.81 | |  |
|  | Overall P | 0.07 | | 0.80 | | 0.31 | | 0.86 | |  |
| Hip Fracture | Effect [95%CI] | 1.06 [0.52, 2.16] | 0.86 [0.63, 1.18] | 1.02 [0.58, 1.78] | 0.89 [0.67, 1.16] | 1.11 [0.55, 2.23] | 0.98 [0.72, 1.35] | Within only one study | |  |
|  | n studies | 3 | 6 | 11 | 6 | 12 | 6 |  |  |  |
|  | $I^{2}$ | 0 | 0 | 0 | 0 | 0 | 0 |  |  |  |
|  | Subgroup difference | 0.60 | | 0.67 | | 0.75 | |  |  |  |
|  | Overall P | 0.43 | | 0.44 | | 0.97 | |  |  |  |
| Vertebral + Hip Fracture | Effect [95%CI] | 0.94 [0.51, 1.76] | 0.80 [0.62, 1.05] | 1.05 [0.67, 1.64] | 0.86 [0.68, 1.08] | 1.01 [0.55, 1.84] | 0.97 [0.73, 1.27] | - | 0.67 [0.13, 3.49] |  |
|  | n studies | 5 | 7 | 13 | 6 | 15 | 7 | - | 2 |  |
|  | $I^{2}$ | 0 | 0 | 0 | 0 | 0 | 0 | - | 0 |  |
|  | Subgroup difference | 0.64 | | 0.42 | | 0.90 | | NA | |  |
|  | Overall P | 0.12 | | 0.29 | | 0.84 | | 0.64 | |  |
| Multiple Fracture | Effect [95%CI] | 1.00 [0.09,11.08] | 1.26 [0.32,5.05] | 0.97 [0.06,15.66] | 0.79 [0.20,3.17] | 0.66 [0.11,4.09] | 0.71 [0.22,2.25] | - | |  |
|  | n studies | 1 | 2 | 1 | 3 | 2 | 2 |  |  |  |
|  | $I^{2}$ | NA | 0 | NA | 0 | 0 | 0 |  |  |  |
|  | Subgroup difference | 0.87 | | 0.90 | | 0.95 | |  |  |  |
|  | Overall P | 0.78 | | 0.76 | | 0.47 | |  |  |  |
| Overall Fracture | Effect [95%CI] | 0.96 [0.64,1.44] | 0.78 [0.58,1.03] | 0.81 [0.60,1.10] | 1.03 [0.85,1.25] | 0.85 [0.61,1.19] | 0.87 [0.67,1.14] | 1.88 [0.70,5.08] | 0.78 [0.09,6.81] |  |
|  | n studies | 23 | 7 | 42 | 6 | 46 | 5 | 5 | 2 |  |
|  | $I^{2}$ | 0 | 16 | 0 | 23 | 0 | 8 | 0 | 60 |  |
|  | Subgroup difference | 0.41 | | 0.19 | | 0.92 | | 0.47 | |  |
|  | Overall P | 0.08 | | 0.64 | | 0.17 | | 0.63 | |  |
| Vertebral Column Fracture | Effect [95%CI] | 0.67 [0.17, 2.60] | 0.64 [0.38, 1.08] | 1.13 [0.52, 2.43] | 0.62 [0.31, 1.25] | 0.72 [0.20, 2.60] | 1.04 [0.58, 1.89] | Within only one study | | |
|  | n studies | 3 | 7 | 8 | 6 | 4 | 6 |  |  |  |
|  | $I^{2}$ | 0 | 0 | 0 | 0 | 0 | 0 |  |  |  |
|  | Subgroup difference | 0.95 | | 0.26 | | 0.60 | |  |  |  |
|  | Overall P | 0.08 | | 0.44 | | 0.93 | |  |  |  |
| Lower Extremity Fracture | Effect [95%CI] | 0.79 [0.43, 1.45] | 0.85 [0.67, 1.09] | 1.15 [0.81, 1.62] | 0.97 [0.79, 1.19] | 0.84 [0.56, 1.28] | 1.07 [0.80, 1.42] | 1.26 [0.25, 6.47] | 0.83 [0.07, 9.23] | |
|  | n studies | 7 | 6 | 24 | 7 | 28 | 8 | 2 | 1 | |
|  | $I^{2}$ | 0 | 0 | 0 | 0 | 0 | 18 | 37 | NA | |
|  | Subgroup difference | 0.83 | | 0.41 | | 0.36 | | 0.78 | | |
|  | Overall P | 0.14 | | 0.89 | | 0.96 | | 0.76 | | |
| Upper Extremity Fracture | Effect [95%CI] | 0.75 [0.40, 1.42] | 0.85 [0.62, 1.18] | 1.02 [0.61, 1.71] | 0.98 [0.71, 1.36] | 0.74 [0.33, 1.66] | 0.87 [0.60, 1.26] | 0.71 [0.09, 5.37] | - | |
|  | n studies | 9 | 6 | 12 | 6 | 8 | 6 | 2 | - | |
|  | $I^{2}$ | 0 | 0 | 0 | 24 | 0 | 0 | 29 | - | |
|  | Subgroup difference | 0.73 | | 0.89 | | 0.73 | | NA | | |
|  | Overall P | 0.21 | | 0.86 | | 0.33 | | 0.69 | | |
| Pelvic Girdle Fracture | Effect [95%CI] | 1.59 [0.52, 4.90] | 0.89 [0.39, 2.01] | 1.02 [0.43, 2.42] | 0.78 [0.51, 1.20] | 1.93 [0.35, 10.59] | 0.89 [0.55, 1.44] | Within only one study | | |
|  | n studies | 3 | 4 | 5 | 6 | 2 | 5 |  |  |  |
|  | $I^{2}$ | 0 | 42 | 0 | 0 | 0 | 0 |  |  |  |
|  | Subgroup difference | 0.41 | | 0.59 | | 0.39 | |  |  |  |
|  | Overall P | 0.96 | | 0.32 | | 0.81 | |  |  |  |
| Cranial and Facial bones Fracture | Effect [95%CI] | 0.91 [0.30, 2.75] | 0.79 [0.29, 2.14] | 1.46 [0.41, 5.22] | 1.54 [0.77, 3.09] | 0.50 [0.09, 2.74] | 0.84 [0.44, 1.63] | Within only one study | | |
|  | n studies | 5 | 4 | 3 | 5 | 2 | 4 |  |  |  |
|  | $I^{2}$ | 0 | 0 | 0 | 0 | 0 | 0 |  |  |  |
|  | Subgroup difference | 0.85 | | 0.94 | | 0.57 | |  |  |  |
|  | Overall P | 0.65 | | 0.18 | | 0.45 | |  |  |  |
| Thoracic Cage Fracture | Effect [95%CI] | 0.85 [0.33,2.21] | 0.70 [0.38,1.27] | 0.97 [0.36,2.59] | 1.12 [0.66,1.91] | 0.88 [0.39,1.99] | 0.72 [0.37,1.42] | 2.00 [0.18,22.72] | 0.20 [0.04,1.12] | |
|  | n studies | 5 | 5 | 5 | 5 | 9 | 6 | 1 | 1 | |
|  | $I^{2}$ | 0 | 0 | 0 | 29 | 0 | 0 | NA | NA | |
|  | Subgroup difference | 0.72 | | 0.80 | | 0.71 | | 0.13 | | |
|  | Overall P | 0.24 | | 0.74 | | 0.36 | | 0.24 | | |

Table S40. Publication bias of meta-analysis results between anti-diabetic agents

| **Outcome** | **Method** | **t** | **df** | **p-value** |
| --- | --- | --- | --- | --- |
| Bone mineral density |  |  |  |  |
| Total hip | Egger | 0.36 | 6 | 0.7344 |
| Lumber spine | Egger | 2.18 | 6 | 0.0717 |
| Femoral neck | Egger | -0.65 | 5 | 0.5437 |
| Fracture |  |  |  |  |
| Overall Fracture | Egger | -1.66 | 135 | 0.1001 |
| Vertebral Fracture | Egger | 0.05 | 33 | 0.9623 |
| Non-Vertebral Fracture | Egger | -2.07 | 82 | 0.0814 |
| Hip Fracture | Egger | 0.11 | 43 | 0.9106 |
| Vertebral+Hip Fracture | Egger | -0.11 | 53 | 0.9107 |
| Multiple Fracture | Egger | -0.73 | 9 | 0.4843 |
| Anatomy Fracture | | | | |
| Vertebral Column Fracture | Egger | -0.36 | 33 | 0.7201 |
| Lower Extremity Fracture | Egger | -1.40 | 81 | 0.1667 |
| Upper Extremity Fracture | Egger | -0.89 | 47 | 0.3796 |
| Pelvic Girdle Fracture | Egger | 0.47 | 24 | 0.6419 |
| Cranial and Facial bones Fracture | Egger | -1.16 | 22 | 0.2589 |
| Multiple Fracture | Egger | -0.73 | 9 | 0.4843 |
| Thoracic Cage Fracture | Egger | -0.59 | 36 | 0.5610 |

Table S41. Sensitivity analysis that excludes studies with the most participants

|  | Group | Measures | GLP1RA | SGLT2i | DPP4i | Thiazolidinedione | Metformin |
| --- | --- | --- | --- | --- | --- | --- | --- |
| Femoral neck BMD | | Effect [95%CI] | NA | 0.03[-0.09,0.16] | NA | -0.10[-0.62,0.43] | NA |
|  |  | n studies | NA | 4 | NA | 2 | NA |
|  |  | n participants | NA | 1,168 | NA | 56 | NA |
|  |  | $I^{2}$ | NA | 31 | NA | 0 | NA |
| Total hip BMD | | Effect [95%CI] | 2.50 | 0.00[-0.15,0.16] | NA | -0.35[-0.77,0.07] | NA |
|  |  | n studies | 1 | 4 | NA | 2 | NA |
|  |  | n participants | 60 | 1,168 | NA | 135 | NA |
|  |  | $I^{2}$ | NA | 38 | NA | 32 | NA |
| Lumber spine BMD | | Effect [95%CI] | NA | 0.04[-0.08,0.15] | NA | 0.49[-0.03,1.01] | NA |
|  |  | n studies | NA | 4 | NA | 3 | NA |
|  |  | n participants | NA | 1,177 | NA | 143 | NA |
|  |  | $I^{2}$ | NA | 0 | NA | 36 | NA |
| Vertebral Fracture | | Effect [95%CI] | 0.64 [0.39, 1.05] | 0.88 [0.51, 1.53] | 0.98 [0.57, 1.67] | 0.41 [0.03,6.66] | NA |
|  |  | n studies | 10 | 13 | 10 | 1 | NA |
|  |  | n participants | 42,078 | 34,612 | 50,869 | 392 | NA |
|  |  | $I^{2}$ | 0 | 0 | 0 | NA | NA |
| Non-Vertebral Fracture | | Effect [95%CI] | 0.83 [0.67, 1.01] | 1.03 [0.88, 1.21\] | 0.95 [0.75, 1.21] | 1.09 [0.36, 3.30] | NA |
|  |  | n studies | 18 | 33 | 28 | 4 | NA |
|  |  | n participants | 45,023 | 57,082 | 43,014 | 995 | NA |
|  |  | $I^{2}$ | 0 | 0 | 0 | 1 | NA |
| Hip Fracture | | Effect [95%CI] | 0.89 [0.67, 1.19] | 0.97 [0.72, 1.32] | 1.01 [0.76, 1.34] | 0.87 [0.11, 6.77] | NA |
|  |  | n studies | 9 | 16 | 18 | 1 | NA |
|  |  | n participants | 40,594 | 35,089 | 54,092 | 49 | NA |
|  |  | $I^{2}$ | 0 | 0 | 0 | NA | NA |
| Vertebral + Hip Fracture | | Effect [95%CI] | 0.82 [0.65, 1.05] | 0.97 [0.76, 1.25] | 0.98 [0.76, 1.25] | 0.67 [0.13, 3.49] | NA |
|  |  | n studies | 12 | 18 | 22 | 2 | NA |
|  |  | n participants | 42,710 | 36,151 | 69,671 | 441 | NA |
|  |  | $I^{2}$ | 0 | 0 | 0 | 0 | NA |
|  | Multiple Fracture | Effect [95%CI] | 1.19[0.36,3.96] | 0.60[0.14,2.55] | 0.70[0.26,1.85] | NA | NA |
|  |  | n studies | 3 | 3 | 4 | NA | NA |
|  |  | n participants | 19,716 | 15,707 | 22,367 | NA | NA |
|  |  | $I^{2}$ | 0 | 0 | 0 | NA | NA |
| Vertebral Column Fracture | | Effect [95%CI] | 0.64 [0.39, 1.05] | 0.88 [0.51, 1.53] | 0.98 [0.57, 1.67] | 0.41 [0.03,6.66] | NA |
|  |  | n studies | 10 | 13 | 10 | 1 | NA |
|  |  | n participants | 42,078 | 34,612 | 50,869 | 392 | NA |
|  |  | $I^{2}$ | 0 | 0 | 0 | NA | NA |
| Lower Extremity Fracture | | Effect [95%CI] | 0.83 [0.66,1.06] | 0.98 [0.76, 1.27] | 0.83 [0.62, 1.11] | 1.82 [0.46, 7.31] | NA |
|  |  | n studies | 12 | 30 | 35 | 2 | NA |
|  |  | n participants | 32,912 | 39,847 | 45,156 | 478 | NA |
|  |  | $I^{2}$ | 0 | 0 | 0 | 0 | NA |
| Upper Extremity Fracture | | Effect [95%CI] | 0.80 [0.55, 1.16] | 1.01 [0.79, 1.31] | 0.78 [0.54, 1.10] | 2.00 [0.18, 22.72] | NA |
|  |  | n studies | 15 | 18 | 14 | 1 | NA |
|  |  | n participants | 43,853 | 51,846 | 52,692 | 111 | NA |
|  |  | $I^{2}$ | 0 | 0 | 0 | NA | NA |
| Pelvic Girdle Fracture | | Effect [95%CI] | 1.00 [0.53, 1.89] | 0.83 [0.53, 1.32] | 0.88 [0.49, 1.55] | 0.88 [0.11, 6.77] | NA |
|  |  | n studies | 6 | 12 | 7 | 1 | NA |
|  |  | n participants | 38,541 | 49,918 | 45,216 | 49 | NA |
|  |  | $I^{2}$ | 17 | 0 | 0 | NA | NA |
| Cranial and Facial bones Fracture | | Effect [95%CI] | 0.78 [0.33, 1.85] | 1.26 [0.62, 2.56] | 0.74 [0.37, 1.47] | NA | NA |
|  |  | n studies | 8 | 7 | 5 | NA | NA |
|  |  | n participants | 22,698 | 31,656 | 25,188 | NA | NA |
|  |  | $I^{2}$ | 0 | 0 | 0 | NA | NA |
| Thoracic Cage Fracture | | Effect [95%CI] | 0.74[0.45,1.23] | 1.25[0.77,2.02] | 0.78[0.47,1.32] | 0.54[0.06,4.96] | NA |
|  |  | n studies | 11 | 9 | 15 | 2 | NA |
|  |  | n participants | 32,378 | 32,463 | 54,563 | 503 | NA |
|  |  | $I^{2}$ | 0 | 0 | 0 | 38 | NA |
|  | Overall Fracture | Effect [95%CI] | 0.84[0.69,1.02] | 1.03[0.79,10.73] | 0.87[0.71,1.06] | 1.33[0.54,3.29] | 0.96[0.09,10.73] |
|  |  | n studies | 30 | 47 | 51 | 7 | 1 |
|  |  | n participants | 49,588 | 40,557 | 47,285 | 1,340 | 204 |
|  |  | $I^{2}$ | 0 | 0 | 0 | 10 | NA |

Table S42. GRADE summary

| Outcome | No.of participants | Risk of bias | Inconsistency | Indirectness | Imprecision | Other considerations | Effect (95%CI) | Quality of  Evidence |
| --- | --- | --- | --- | --- | --- | --- | --- | --- |
| BMD | | | | | | | | |
| Femoral neck | 1,631 | Not serious | Not serious | Not serious | Not serious | None | SMD 0.02 (-0.07, 0.12) | ⨁⨁⨁○ MODERATE |
| Lumber spine | 1,727 | Not serious | Not serious | Not serious | Not serious | None | SMD 0.06 (-0.03, 0.16) | ⨁⨁⨁○ MODERATE |
| Total hip | 1,770 | Not serious | Serious | Not serious | Not serious | None | SMD -0.12 (-1.09, 0.85) | ⨁⨁⨁○ MODERATE |
| Fracture | | | | | | | | |
| Overall | 200,971 | Not serious | Not serious | Not serious | Not serious | None | OR 0.92 (0.84, 1.01) | ⨁⨁⨁⨁  HIGH |
| Vertebral | 160,683 | Not serious | Not serious | Not serious | Not serious | None | OR 0.81 (0.62, 1.04) | ⨁⨁⨁⨁  HIGH |
| Non-Vertebral | 186,142 | Not serious | Not serious | Not serious | Not serious | None | OR 0.95 (0.85, 1.06) | ⨁⨁⨁⨁  HIGH |
| Vertebral + Hip | 187,065 | Not serious | Not serious | Not serious | Not serious | None | OR 0.89 [0.78, 1.02] | ⨁⨁⨁⨁  HIGH |
| Hip | 161,405 | Not serious | Not serious | Not serious | Not serious | None | OR 0.93 (0.80, 1.09) | ⨁⨁⨁⨁  HIGH |
| Multiple | 74,933 | Not serious | Not serious | Not serious | Serious | None | OR 0.85 (0.45, 1.62) | ⨁⨁⨁○ MODERATE |
| Anatomy Fracture | | | | | | | | |
| Vertebral Column | 162,326 | Not serious | Not serious | Not serious | Not serious | None | OR 0.78 (0.58, 1.05) | ⨁⨁⨁⨁  HIGH |
| Lower Extremity | 162,326 | Not serious | Not serious | Not serious | Not serious | None | OR 0.96 (0.85, 1.08) | ⨁⨁⨁⨁  HIGH |
| Upper Extremity | 148,908 | Not serious | Not serious | Not serious | Not serious | None | OR 0.90 (0.77, 1.06) | ⨁⨁⨁⨁  HIGH |
| Pelvic Girdle | 133,724 | Not serious | Not serious | Not serious | Not serious | None | OR 0.91 (0.71, 1.16) | ⨁⨁⨁⨁  HIGH |
| Cranial and Facial bones | 123,475 | Not serious | Not serious | Not serious | Serious | None | OR 1.01 (0.70, 1.47) | ⨁⨁⨁○ MODERATE |
| Thoracic Cage | 137,050 | Not serious | Not serious | Not serious | Not serious | None | OR 0.87 (0.67, 1.13) | ⨁⨁⨁⨁  HIGH |

Table S43. Meta-regression of BMD

|  | | Estimate | SE | Zvalue | Pvalue | Lower CI | Upper CI |
| --- | --- | --- | --- | --- | --- | --- | --- |
| Femoral neck BMD | Intrcpt | 0.011 | 0.111 | 0.099 | 0.921 | -0.206 | 0.228 |
|  | Medication duration | 0.000 | 0.002 | 0.117 | 0.907 | -0.003 | 0.003 |
| Total hip BMD | Intrcpt | 0.302 | 1.031 | 0.292 | 0.770 | -1.719 | 2.322 |
|  | Medication duration | -0.007 | 0.016 | -0.471 | 0.638 | -0.038 | 0.023 |
| Lumber spine BMD | Intrcpt | 0.107 | 0.097 | 1.107 | 0.268 | -0.083 | 0.297 |
|  | Medication duration | -0.001 | 0.001 | -0.549 | 0.583 | -0.004 | 0.002 |

**Figure S1. Network plot for site of fracture between specific anti-diabetic agents.**

The size of each node represents the total number of patients who were randomized to receive treatment. The width of each edge represents the number of trials that were conducted in the pairwise comparison; a. Vertebral Fracture b. Non-Vertebral Fracture c. Hip Fracture d. Vertebral and Hip Fracture e. Multiple Fracture

**Figure S2. Network plot for anatomy fracture site between specific anti-diabetic agents.**

The size of each node represents the total number of patients who were randomized to receive treatment. The width of each edge represents the number of trials that were conducted in the pairwise comparison; a. Vertebral Column Fracture b. Lower Extremity Fracture c. Upper Extremity d. Pelvic Girdle e. Cranial and facial bones Fracture f. Thoracic Cage Fracture

**Figure S3. Network plot for BMD between specific anti-diabetic agents.**

The size of each node represents the total number of patients who were randomized to receive treatment. The width of each edge represents the number of trials that were conducted in the pairwise comparison; a. Total hip b. Lumber spine c. Femoral neck

**Figure S4. Network plot for site of fracture between type of anti-diabetic agents**

The size of each node represents the total number of patients who were randomized to receive treatment. The width of each edge represents the number of trials that were conducted in the pairwise comparison; a. Vertebral Fracture b. Non-Vertebral Fracture c. Hip Fracture d. Vertebral and Hip Fracture e. Multiple Fracture

**Figure** **S5.** **Network plot for anatomy fracture site between specific anti-diabetic agents.**

The size of each node represents the total number of patients who were randomized to receive treatment. The width of each edge represents the number of trials that were conducted in the pairwise comparison; a. Vertebral Column Fracture b. Lower Extremity Fracture c. Upper Extremity d. Pelvic Girdle e. Cranial and facial bones Fracture f. Thoracic Cage Fracture

**Figure S6. Network plot for site of BMD between type of anti-diabetic agents.**

The size of each node represents the total number of patients who were randomized to receive treatment. The width of each edge represents the number of trials that were conducted in the pairwise comparison; a. Total hip b. Lumber spine c. Femoral neck

**Figure S7. Forest plot of network meta-analysis results for site of fracture between specific anti-diabetic agents.**

a. Vertebral Fracture b. Non-Vertebral Fracture c. Hip Fracture d. Vertebral and Hip Fracture ; Results are presented as odds risks (ORs) with 95% confidence intervals (CIs). Treatments are ranked according to their relative effectiveness profiles compared to the reference treatment. Plots depict specific fracture sites. Vertical dashed lines indicate the reference line of Placebo (OR=1). Treatments whose credible intervals do not cross the reference line indicate significant differences in fracture risk compared with the reference treatment.

**Figure S7. Forest plot of network meta-analysis results for site of fracture between specific anti-diabetic agents (Continuous).**

e. Multiple Fracture f. Overall Fracture; Results are presented as odds risks (ORs) with 95% confidence intervals (CIs). Treatments are ranked according to their relative effectiveness profiles compared to the reference treatment. Plots depict specific fracture sites. Vertical dashed lines indicate the reference line of Placebo (OR=1). Treatments whose credible intervals do not cross the reference line indicate significant differences in fracture risk compared with the reference treatment.

**Figure S8. Forest plot of network meta-analysis results for anatomy fracture site between specific anti-diabetic agents.**

a. Vertebral Column Fracture b. Lower Extremity Fracture c. Upper Extremity d. Pelvic Girdle e. Cranial and facial bones Fracture f. Thoracic Cage Fracture; Results are presented as odds risks (ORs) with 95% confidence intervals (CIs). Treatments are ranked according to their relative effectiveness profiles compared to the reference treatment. Plots depict specific fracture sites. Vertical dashed lines indicate the reference line of Placebo (OR=1). Treatments whose credible intervals do not cross the reference line indicate significant differences in fracture risk compared with the reference treatment.

**Figure S9. Forest plot of network meta-analysis results for BMD between type of anti-diabetic agents.**

a. Total hip b. Lumber spine c. Femoral neck; Results are presented as standard mean difference (SMD) with 95% confidence intervals (CIs). Treatments are ranked according to their relative effectiveness profiles compared to the reference treatment. Plots depict specific sites. Vertical dashed lines indicate the reference line of Placebo. Treatments whose credible intervals do not cross the reference line indicate significant differences in fracture risk compared with the reference treatment.

**Figure S10. Forest plot of network meta-analysis results for BMD between specific anti-diabetic agents**

a. Total hip b. Lumber spine c. Femoral neck; Results are presented as standard mean difference (SMD) with 95% confidence intervals (CIs). Treatments are ranked according to their relative effectiveness profiles compared to the reference treatment. Plots depict specific sites. Vertical dashed lines indicate the reference line of Placebo. Treatments whose credible intervals do not cross the reference line indicate significant differences in fracture risk compared with the reference treatment.

**Figure S11. Funnel plot of network meta-analysis results for site of fracture between specific anti-diabetic agents.**

a. Vertebral Fracture b. Non-Vertebral Fracture c. Hip Fracture d. Vertebral and Hip Fracture e. Multiple Fracture f. Overall Fracture

**Figure S12. Funnel plot of network meta-analysis results for anatomy fracture site between specific anti-diabetic agents.**

a. Vertebral Column Fracture b. Lower Extremity Fracture c. Upper Extremity d. Pelvic Girdle e. Cranial and facial bones Fracture f. Thoracic Cage Fracture

**Figure S13. Funnel plot of network meta-analysis results for site of fracture between type of anti-diabetic agents.**

a. Vertebral Fracture b. Non-Vertebral Fracture c. Hip Fracture d. Vertebral and Hip Fracture e. Multiple Fracture f. Overall Fracture

**Figure S14. Funnel plot of network meta-analysis results for anatomy fracture site between type of anti-diabetic agents.**

a. Vertebral Column Fracture b. Lower Extremity Fracture c. Upper Extremity d. Pelvic Girdle e. Cranial and facial bones Fracture f. Thoracic Cage Fracture

**Figure S15. Funnel plot of network meta-analysis results for BMD between specific anti-diabetic agents**

a. Total hip b. Lumber spine c. Femoral neck

**Figure S16. Funnel plot of network meta-analysis results for BMD between type of anti-diabetic agents**

a. Total hip b. Lumber spine c. Femoral neck

Figure S17. Risk of bias assessment (Traffic-light plot)

Figure S17. Risk of bias assessment (Traffic-light plot)

Figure S17. Risk of bias assessment (Traffic-light plot)

Figure S17. Risk of bias assessment (Traffic-light plot)

Figure S17. Risk of bias assessment (Traffic-light plot)

Figure S18. Risk of bias assessment (Summary plot)

Ahmann, A., Rodbard, H. W., Rosenstock, J., Lahtela, J. T., De Loredo, L., Tornøe, K., Boopalan, A., and Nauck, M. A. (2015) Efficacy and safety of liraglutide versus placebo added to basal insulin analogues (with or without metformin) in patients with type 2 diabetes: a randomized, placebo-controlled trial. Diabetes Obes Metab*,* 17**,** 1056-64 10.1111/dom.12539.

Ahmann, A. J., Capehorn, M., Charpentier, G., Dotta, F., Henkel, E., Lingvay, I., Holst, A. G., Annett, M. P., and Aroda, V. R. (2018) Efficacy and Safety of Once-Weekly Semaglutide Versus Exenatide ER in Subjects With Type 2 Diabetes (SUSTAIN 3): A 56-Week, Open-Label, Randomized Clinical Trial. Diabetes Care*,* 41**,** 258-266 10.2337/dc17-0417.

Ahrén, B., Johnson, S. L., Stewart, M., Cirkel, D. T., Yang, F., Perry, C., and Feinglos, M. N. (2014) HARMONY 3: 104-week randomized, double-blind, placebo- and active-controlled trial assessing the efficacy and safety of albiglutide compared with placebo, sitagliptin, and glimepiride in patients with type 2 diabetes taking metformin. Diabetes Care*,* 37**,** 2141-8 10.2337/dc14-0024.

Ahrén, B., Leguizamo Dimas, A., Miossec, P., Saubadu, S., and Aronson, R. (2013) Efficacy and safety of lixisenatide once-daily morning or evening injections in type 2 diabetes inadequately controlled on metformin (GetGoal-M). Diabetes Care*,* 36**,** 2543-50 10.2337/dc12-2006.

Ahrén, B., Masmiquel, L., Kumar, H., Sargin, M., Karsbøl, J. D., Jacobsen, S. H., and Chow, F. (2017) Efficacy and safety of once-weekly semaglutide versus once-daily sitagliptin as an add-on to metformin, thiazolidinediones, or both, in patients with type 2 diabetes (SUSTAIN 2): a 56-week, double-blind, phase 3a, randomised trial. Lancet Diabetes Endocrinol*,* 5**,** 341-354 10.1016/s2213-8587(17)30092-x.

Araki, E., Onishi, Y., Asano, M., Kim, H., Ekholm, E., Johnsson, E., and Yajima, T. (2016) Efficacy and safety of dapagliflozin in addition to insulin therapy in Japanese patients with type 2 diabetes: Results of the interim analysis of 16-week double-blind treatment period. J Diabetes Investig*,* 7**,** 555-64 10.1111/jdi.12453.

Araki, E., Tanizawa, Y., Tanaka, Y., Taniguchi, A., Koiwai, K., Kim, G., Salsali, A., Woerle, H. J., and Broedl, U. C. (2015) Long-term treatment with empagliflozin as add-on to oral antidiabetes therapy in Japanese patients with type 2 diabetes mellitus. Diabetes, Obesity and Metabolism*,* 17**,** 665-674 <https://doi.org/10.1111/dom.12464>.

Araki, E., Unno, Y., Tanaka, Y., Sakamoto, W., and Miyamoto, Y. (2019) Long-Term Efficacy and Safety of Linagliptin in a Japanese Population with Type 2 Diabetes Aged ≥ 60 Years Treated with Basal Insulin: A Randomised Trial. Adv Ther*,* 36**,** 2697-2711 10.1007/s12325-019-01065-7.

Arechavaleta, R., Seck, T., Chen, Y., Krobot, K. J., O'neill, E. A., Duran, L., Kaufman, K. D., Williams-Herman, D., and Goldstein, B. J. (2011) Efficacy and safety of treatment with sitagliptin or glimepiride in patients with type 2 diabetes inadequately controlled on metformin monotherapy: a randomized, double-blind, non-inferiority trial. Diabetes Obes Metab*,* 13**,** 160-8 10.1111/j.1463-1326.2010.01334.x.

Arjona Ferreira, J. C., Marre, M., Barzilai, N., Guo, H., Golm, G. T., Sisk, C. M., Kaufman, K. D., and Goldstein, B. J. (2013) Efficacy and safety of sitagliptin versus glipizide in patients with type 2 diabetes and moderate-to-severe chronic renal insufficiency. Diabetes Care*,* 36**,** 1067-73 10.2337/dc12-1365.

Aroda, V. R., Bailey, T. S., Cariou, B., Kumar, S., Leiter, L. A., Raskin, P., Zacho, J., Andersen, T. H., and Philis-Tsimikas, A. (2016) Effect of adding insulin degludec to treatment in patients with type 2 diabetes inadequately controlled with metformin and liraglutide: a double-blind randomized controlled trial (BEGIN: ADD TO GLP-1 Study). Diabetes Obes Metab*,* 18**,** 663-70 10.1111/dom.12661.

Aroda, V. R., Bain, S. C., Cariou, B., Piletič, M., Rose, L., Axelsen, M., Rowe, E., and Devries, J. H. (2017) Efficacy and safety of once-weekly semaglutide versus once-daily insulin glargine as add-on to metformin (with or without sulfonylureas) in insulin-naive patients with type 2 diabetes (SUSTAIN 4): a randomised, open-label, parallel-group, multicentre, multinational, phase 3a trial. The Lancet Diabetes & Endocrinology*,* 5**,** 355-366 <https://doi.org/10.1016/S2213-8587(17)30085-2>.

Aronson, R., Frias, J., Goldman, A., Darekar, A., Lauring, B., and Terra, S. G. (2018) Long-term efficacy and safety of ertugliflozin monotherapy in patients with inadequately controlled T2DM despite diet and exercise: VERTIS MONO extension study. Diabetes, Obesity and Metabolism*,* 20**,** 1453-1460 10.1111/dom.13251.

Aschner, P., Katzeff, H. L., Guo, H., Sunga, S., Williams-Herman, D., Kaufman, K. D., Goldstein, B. J., and Group, F. T. S. S. (2010) Efficacy and safety of monotherapy of sitagliptin compared with metformin in patients with type 2 diabetes. Diabetes, Obesity and Metabolism*,* 12**,** 252-261 <https://doi.org/10.1111/j.1463-1326.2009.01187.x>.

Aschner, P., Kipnes, M. S., Lunceford, J. K., Sanchez, M., Mickel, C., Williams-Herman, D. E., and Group, F. T. S. S. (2006) Effect of the Dipeptidyl Peptidase-4 Inhibitor Sitagliptin as Monotherapy on Glycemic Control in Patients With Type 2 Diabetes. Diabetes Care*,* 29**,** 2632-2637 10.2337/dc06-0703.

Bailey, C. J., Gross, J. L., Pieters, A., Bastien, A., and List, J. F. (2010) Effect of dapagliflozin in patients with type 2 diabetes who have inadequate glycaemic control with metformin: a randomised, double-blind, placebo-controlled trial. Lancet*,* 375**,** 2223-33 10.1016/s0140-6736(10)60407-2.

Barnett, A. H., Charbonnel, B., Donovan, M., Fleming, D., and Chen, R. (2012) Effect of saxagliptin as add-on therapy in patients with poorly controlled type 2 diabetes on insulin alone or insulin combined with metformin. Curr Med Res Opin*,* 28**,** 513-23 10.1185/03007995.2012.665046.

Barnett, A. H., Huisman, H., Jones, R., Von Eynatten, M., Patel, S., and Woerle, H. J. (2013) Linagliptin for patients aged 70 years or older with type 2 diabetes inadequately controlled with common antidiabetes treatments: a randomised, double-blind, placebo-controlled trial. Lancet*,* 382**,** 1413-23 10.1016/s0140-6736(13)61500-7.

Barnett, A. H., Mithal, A., Manassie, J., Jones, R., Rattunde, H., Woerle, H. J., and Broedl, U. C. (2014) Efficacy and safety of empagliflozin added to existing antidiabetes treatment in patients with type 2 diabetes and chronic kidney disease: a randomised, double-blind, placebo-controlled trial. The Lancet Diabetes & Endocrinology*,* 2**,** 369-384 <https://doi.org/10.1016/S2213-8587(13)70208-0>.

Barzilai, N., Guo, H., Mahoney, E. M., Caporossi, S., Golm, G. T., Langdon, R. B., Williams-Herman, D., Kaufman, K. D., Amatruda, J. M., Goldstein, B. J., and Steinberg, H. (2011) Efficacy and tolerability of sitagliptin monotherapy in elderly patients with type 2 diabetes: a randomized, double-blind, placebo-controlled trial. Curr Med Res Opin*,* 27**,** 1049-58 10.1185/03007995.2011.568059.

Beck, G. R., Jr., Khazai, N. B., Bouloux, G. F., Camalier, C. E., Lin, Y., Garneys, L. M., Siqueira, J., Peng, L., Pasquel, F., Umpierrez, D., Smiley, D., and Umpierrez, G. E. (2013) The effects of thiazolidinediones on human bone marrow stromal cell differentiation in vitro and in thiazolidinedione-treated patients with type 2 diabetes. Transl Res*,* 161**,** 145-55 10.1016/j.trsl.2012.08.006.

Berberoglu, Z., Yazici, A. C., and Demirag, N. G. (2010) ORIGINAL ARTICLE: Effects of rosiglitazone on bone mineral density and remodelling parameters in Postmenopausal diabetic women: a 2-year follow-up study. Clinical Endocrinology*,* 73**,** 305-312 <https://doi.org/10.1111/j.1365-2265.2010.03784.x>.

Bethel, M. A., Engel, S. S., Green, J. B., Huang, Z., Josse, R. G., Kaufman, K. D., Standl, E., Suryawanshi, S., Van De Werf, F., Mcguire, D. K., Peterson, E. D., Holman, R. R., and Group, F. T. T. S. (2017) Assessing the Safety of Sitagliptin in Older Participants in the Trial Evaluating Cardiovascular Outcomes with Sitagliptin (TECOS). Diabetes Care*,* 40**,** 494-501 10.2337/dc16-1135.

Bhatt, D. L., Szarek, M., Pitt, B., Cannon, C. P., Leiter, L. A., Mcguire, D. K., Lewis, J. B., Riddle, M. C., Inzucchi, S. E., Kosiborod, M. N., Cherney, D. Z. I., Dwyer, J. P., Scirica, B. M., Bailey, C. J., Díaz, R., Ray, K. K., Udell, J. A., Lopes, R. D., Lapuerta, P., and Gabriel Steg, P. (2021) Sotagliflozin in patients with diabetes and chronic kidney disease. New England Journal of Medicine*,* 384**,** 129-139 10.1056/NEJMoa2030186.

Bilezikian, J. P., Josse, R. G., Eastell, R., Lewiecki, E. M., Miller, C. G., Wooddell, M., Northcutt, A. R., Kravitz, B. G., Paul, G., Cobitz, A. R., Nino, A. J., and Fitzpatrick, L. A. (2013) Rosiglitazone decreases bone mineral density and increases bone turnover in postmenopausal women with type 2 diabetes mellitus. J Clin Endocrinol Metab*,* 98**,** 1519-28 10.1210/jc.2012-4018.

Bilezikian, J. P., Watts, N. B., Usiskin, K., Polidori, D., Fung, A., Sullivan, D., and Rosenthal, N. (2016) Evaluation of Bone Mineral Density and Bone Biomarkers in Patients With Type 2 Diabetes Treated With Canagliflozin. J Clin Endocrinol Metab*,* 101**,** 44-51 10.1210/jc.2015-1860.

Blonde, L., Jendle, J., Gross, J., Woo, V., Jiang, H., Fahrbach, J. L., and Milicevic, Z. (2015) Once-weekly dulaglutide versus bedtime insulin glargine, both in combination with prandial insulin lispro, in patients with type 2 diabetes (AWARD-4): a randomised, open-label, phase 3, non-inferiority study. The Lancet*,* 385**,** 2057-2066 <https://doi.org/10.1016/S0140-6736(15)60936-9>.

Bolli, G. B., Munteanu, M., Dotsenko, S., Niemoeller, E., Boka, G., Wu, Y., and Hanefeld, M. (2014) Efficacy and safety of lixisenatide once daily vs. placebo in people with Type 2 diabetes insufficiently controlled on metformin (GetGoal-F1). Diabet Med*,* 31**,** 176-84 10.1111/dme.12328.

Borges, J. L. C., Bilezikian, J. P., Jones-Leone, A. R., Acusta, A. P., Ambery, P. D., Nino, A. J., Grosse, M., Fitzpatrick, L. A., and Cobitz, A. R. (2011) A randomized, parallel group, double-blind, multicentre study comparing the efficacy and safety of Avandamet (rosiglitazone/metformin) and metformin on long-term glycaemic control and bone mineral density after 80 weeks of treatment in drug-naïve type 2 diabetes mellitus patients. Diabetes, Obesity and Metabolism*,* 13**,** 1036-1046 <https://doi.org/10.1111/j.1463-1326.2011.01461.x>.

Bosi, E., Dotta, F., Jia, Y., and Goodman, M. (2009) Vildagliptin plus metformin combination therapy provides superior glycaemic control to individual monotherapy in treatment-naive patients with type 2 diabetes mellitus. Diabetes, Obesity and Metabolism*,* 11**,** 506-515 <https://doi.org/10.1111/j.1463-1326.2009.01040.x>.

Bosi, E., Ellis, G. C., Wilson, C. A., and Fleck, P. R. (2011) Alogliptin as a third oral antidiabetic drug in patients with type 2 diabetes and inadequate glycaemic control on metformin and pioglitazone: a 52-week, randomized, double-blind, active-controlled, parallel-group study. Diabetes, Obesity and Metabolism*,* 13**,** 1088-1096 <https://doi.org/10.1111/j.1463-1326.2011.01463.x>.

Buse, J. B., Han, J., Miller, S., Macconell, L., Pencek, R., and Wintle, M. (2014) Addition of exenatide BID to insulin glargine: a post-hoc analysis of the effect on glycemia and weight across a range of insulin titration. Curr Med Res Opin*,* 30**,** 1209-18 10.1185/03007995.2014.896329.

Buse, J. B., Nauck, M., Forst, T., Sheu, W. H., Shenouda, S. K., Heilmann, C. R., Hoogwerf, B. J., Gao, A., Boardman, M. K., Fineman, M., Porter, L., and Schernthaner, G. (2013) Exenatide once weekly versus liraglutide once daily in patients with type 2 diabetes (DURATION-6): a randomised, open-label study. Lancet*,* 381**,** 117-24 10.1016/s0140-6736(12)61267-7.

Buse, J. B., Rosenstock, J., Sesti, G., Schmidt, W. E., Montanya, E., Brett, J. H., Zychma, M., and Blonde, L. (2009) Liraglutide once a day versus exenatide twice a day for type 2 diabetes: a 26-week randomised, parallel-group, multinational, open-label trial (LEAD-6). Lancet*,* 374**,** 39-47 10.1016/s0140-6736(09)60659-0.

Cannon, C. P., Pratley, R., Dagogo-Jack, S., Mancuso, J., Huyck, S., Masiukiewicz, U., Charbonnel, B., Frederich, R., Gallo, S., Cosentino, F., Shih, W. J., Gantz, I., Terra, S. G., Cherney, D. Z. I., and Mcguire, D. K. (2020) Cardiovascular Outcomes with Ertugliflozin in Type 2 Diabetes. N Engl J Med*,* 383**,** 1425-1435 10.1056/NEJMoa2004967.

Cefalu, W. T., Leiter, L. A., De Bruin, T. W., Gause-Nilsson, I., Sugg, J., and Parikh, S. J. (2015) Dapagliflozin's Effects on Glycemia and Cardiovascular Risk Factors in High-Risk Patients With Type 2 Diabetes: A 24-Week, Multicenter, Randomized, Double-Blind, Placebo-Controlled Study With a 28-Week Extension. Diabetes Care*,* 38**,** 1218-27 10.2337/dc14-0315.

Cefalu, W. T., Leiter, L. A., Yoon, K.-H., Arias, P., Niskanen, L., Xie, J., Balis, D. A., Canovatchel, W., and Meininger, G. (2013) Efficacy and safety of canagliflozin versus glimepiride in patients with type 2 diabetes inadequately controlled with metformin (CANTATA-SU): 52 week results from a randomised, double-blind, phase 3 non-inferiority trial. The Lancet*,* 382**,** 941-950 <https://doi.org/10.1016/S0140-6736(13)60683-2>.

Cherney, D. Z. I., Ferrannini, E., Umpierrez, G. E., Peters, A. L., Rosenstock, J., Powell, D. R., Davies, M. J., Banks, P., and Agarwal, R. (2023) Efficacy and safety of sotagliflozin in patients with type 2 diabetes and stage 3 chronic kidney disease. Diabetes, Obesity and Metabolism*,* 25**,** 1646-1657 10.1111/dom.15019.

Dagogo-Jack, S., Liu, J., Eldor, R., Amorin, G., Johnson, J., Hille, D., Liao, Y., Huyck, S., Golm, G., Terra, S. G., Mancuso, J. P., Engel, S. S., and Lauring, B. (2018) Efficacy and safety of the addition of ertugliflozin in patients with type 2 diabetes mellitus inadequately controlled with metformin and sitagliptin: The VERTIS SITA2 placebo-controlled randomized study. Diabetes Obes Metab*,* 20**,** 530-540 10.1111/dom.13116.

Davies, M. J., Bergenstal, R., Bode, B., Kushner, R. F., Lewin, A., Skjøth, T. V., Andreasen, A. H., Jensen, C. B., Defronzo, R. A., and Group, F. T. N.-S. (2015) Efficacy of Liraglutide for Weight Loss Among Patients With Type 2 Diabetes: The SCALE Diabetes Randomized Clinical Trial. JAMA*,* 314**,** 687-699 10.1001/jama.2015.9676.

Defronzo, R. A., Burant, C. F., Fleck, P., Wilson, C., Mekki, Q., and Pratley, R. E. (2012) Efficacy and Tolerability of the DPP-4 Inhibitor Alogliptin Combined with Pioglitazone, in Metformin-Treated Patients with Type 2 Diabetes. The Journal of Clinical Endocrinology & Metabolism*,* 97**,** 1615-1622 10.1210/jc.2011-2243.

Defronzo, R. A., Lewin, A., Patel, S., Liu, D., Kaste, R., Woerle, H. J., and Broedl, U. C. (2015) Combination of Empagliflozin and Linagliptin as Second-Line Therapy in Subjects With Type 2 Diabetes Inadequately Controlled on Metformin. Diabetes Care*,* 38**,** 384-393 10.2337/dc14-2364.

Del Prato, S., Patel, S., Crowe, S., and Von Eynatten, M. (2016) Efficacy and safety of linagliptin according to patient baseline characteristics: A pooled analysis of three phase 3 trials. Nutrition, Metabolism and Cardiovascular Diseases*,* 26**,** 886-892 <https://doi.org/10.1016/j.numecd.2016.06.015>.

Diamant, M., Nauck, M. A., Shaginian, R., Malone, J. K., Cleall, S., Reaney, M., De Vries, D., Hoogwerf, B. J., Macconell, L., and Wolffenbuttel, B. H. (2014) Glucagon-like peptide 1 receptor agonist or bolus insulin with optimized basal insulin in type 2 diabetes. Diabetes Care*,* 37**,** 2763-73 10.2337/dc14-0876.

Dobs, A. S., Goldstein, B. J., Aschner, P., Horton, E. S., Umpierrez, G. E., Duran, L., Hill, J. S., Chen, Y., Golm, G. T., Langdon, R. B., Williams-Herman, D. E., Kaufman, K. D., Amatruda, J. M., and Ferreira, J. C. (2013) Efficacy and safety of sitagliptin added to ongoing metformin and rosiglitazone combination therapy in a randomized placebo-controlled 54-week trial in patients with type 2 diabetes. J Diabetes*,* 5**,** 68-79 10.1111/j.1753-0407.2012.00223.x.

Dungan, K. M., Weitgasser, R., Perez Manghi, F., Pintilei, E., Fahrbach, J. L., Jiang, H. H., Shell, J., and Robertson, K. E. (2016) A 24-week study to evaluate the efficacy and safety of once-weekly dulaglutide added on to glimepiride in type 2 diabetes (AWARD-8). Diabetes Obes Metab*,* 18**,** 475-82 10.1111/dom.12634.

Fioretto, P., Del Prato, S., Buse, J. B., Goldenberg, R., Giorgino, F., Reyner, D., Langkilde, A. M., Sjöström, C. D., and Sartipy, P. (2018) Efficacy and safety of dapagliflozin in patients with type 2 diabetes and moderate renal impairment (chronic kidney disease stage 3A): The DERIVE Study. Diabetes Obes Metab*,* 20**,** 2532-2540 10.1111/dom.13413.

Fonseca, V., Schweizer, A., Albrecht, D., Baron, M. A., Chang, I., and Dejager, S. (2007) Addition of vildagliptin to insulin improves glycaemic control in type 2 diabetes. Diabetologia*,* 50**,** 1148-55 10.1007/s00125-007-0633-0.

Fonseca, V., Staels, B., Morgan, J. D., 2nd, Shentu, Y., Golm, G. T., Johnson-Levonas, A. O., Kaufman, K. D., Goldstein, B. J., and Steinberg, H. (2013) Efficacy and safety of sitagliptin added to ongoing metformin and pioglitazone combination therapy in a randomized, placebo-controlled, 26-week trial in patients with type 2 diabetes. J Diabetes Complications*,* 27**,** 177-83 10.1016/j.jdiacomp.2012.09.007.

Fonseca, V. A., Alvarado-Ruiz, R., Raccah, D., Boka, G., Miossec, P., and Gerich, J. E. (2012) Efficacy and safety of the once-daily GLP-1 receptor agonist lixisenatide in monotherapy: a randomized, double-blind, placebo-controlled trial in patients with type 2 diabetes (GetGoal-Mono). Diabetes Care*,* 35**,** 1225-31 10.2337/dc11-1935.

Forst, T., Guthrie, R., Goldenberg, R., Yee, J., Vijapurkar, U., Meininger, G., and Stein, P. (2014) Efficacy and safety of canagliflozin over 52 weeks in patients with type 2 diabetes on background metformin and pioglitazone. Diabetes Obes Metab*,* 16**,** 467-77 10.1111/dom.12273.

Frederich, R., Mcneill, R., Berglind, N., Fleming, D., and Chen, R. (2012) The efficacy and safety of the dipeptidyl peptidase-4 inhibitor saxagliptin in treatment-naïve patients with type 2 diabetes mellitus: a randomized controlled trial. Diabetol Metab Syndr*,* 4**,** 36 10.1186/1758-5996-4-36.

Frías, J. P., Guja, C., Hardy, E., Ahmed, A., Dong, F., Öhman, P., and Jabbour, S. A. (2016) Exenatide once weekly plus dapagliflozin once daily versus exenatide or dapagliflozin alone in patients with type 2 diabetes inadequately controlled with metformin monotherapy (DURATION-8): a 28 week, multicentre, double-blind, phase 3, randomised controlled trial. The Lancet Diabetes & Endocrinology*,* 4**,** 1004-1016 <https://doi.org/10.1016/S2213-8587(16)30267-4>.

Gallwitz, B., Guzman, J., Dotta, F., Guerci, B., Simó, R., Basson, B. R., Festa, A., Kiljański, J., Sapin, H., Trautmann, M., and Schernthaner, G. (2012a) Exenatide twice daily versus glimepiride for prevention of glycaemic deterioration in patients with type 2 diabetes with metformin failure (EUREXA): an open-label, randomised controlled trial. Lancet*,* 379**,** 2270-8 10.1016/s0140-6736(12)60479-6.

Gallwitz, B., Rosenstock, J., Rauch, T., Bhattacharya, S., Patel, S., Von Eynatten, M., Dugi, K. A., and Woerle, H. J. (2012b) 2-year efficacy and safety of linagliptin compared with glimepiride in patients with type 2 diabetes inadequately controlled on metformin: a randomised, double-blind, non-inferiority trial. Lancet*,* 380**,** 475-83 10.1016/s0140-6736(12)60691-6.

Gantz, I., Chen, M., Suryawanshi, S., Ntabadde, C., Shah, S., O'neill, E. A., Engel, S. S., Kaufman, K. D., and Lai, E. (2017) A randomized, placebo-controlled study of the cardiovascular safety of the once-weekly DPP-4 inhibitor omarigliptin in patients with type 2 diabetes mellitus. Cardiovasc Diabetol*,* 16**,** 112 10.1186/s12933-017-0593-8.

Garber, A., Henry, R., Ratner, R., Garcia-Hernandez, P. A., Rodriguez-Pattzi, H., Olvera-Alvarez, I., Hale, P. M., Zdravkovic, M., and Bode, B. (2009) Liraglutide versus glimepiride monotherapy for type 2 diabetes (LEAD-3 Mono): a randomised, 52-week, phase III, double-blind, parallel-treatment trial. The Lancet*,* 373**,** 473-481 <https://doi.org/10.1016/S0140-6736(08)61246-5>.

Genovese, S., Passaro, A., Brunetti, P., Comaschi, M., Cucinotta, D., Egan, C. G., Chinea, B., Bravi, F., and Di Pietro, C. (2013) Pioglitazone randomised Italian study on metabolic syndrome (PRISMA): Effect of pioglitazone withmetformin on HDL-C levels in type 2 diabetic patients. Journal of Endocrinological Investigation*,* 36**,** 606-616 10.3275/8895.

Gerstein, H. C., Colhoun, H. M., Dagenais, G. R., Diaz, R., Lakshmanan, M., Pais, P., Probstfield, J., Riesmeyer, J. S., Riddle, M. C., Rydén, L., Xavier, D., Atisso, C. M., Dyal, L., Hall, S., Rao-Melacini, P., Wong, G., Avezum, A., Basile, J., Chung, N., Conget, I., Cushman, W. C., Franek, E., Hancu, N., Hanefeld, M., Holt, S., Jansky, P., Keltai, M., Lanas, F., Leiter, L. A., Lopez-Jaramillo, P., Cardona Munoz, E. G., Pirags, V., Pogosova, N., Raubenheimer, P. J., Shaw, J. E., Sheu, W. H., and Temelkova-Kurktschiev, T. (2019) Dulaglutide and cardiovascular outcomes in type 2 diabetes (REWIND): a double-blind, randomised placebo-controlled trial. Lancet*,* 394**,** 121-130 10.1016/s0140-6736(19)31149-3.

Gill, A., Hoogwerf, B. J., Burger, J., Bruce, S., Macconell, L., Yan, P., Braun, D., Giaconia, J., and Malone, J. (2010) Effect of exenatide on heart rate and blood pressure in subjects with type 2 diabetes mellitus: a double-blind, placebo-controlled, randomized pilot study. Cardiovasc Diabetol*,* 9**,** 6 10.1186/1475-2840-9-6.

Giorgino, F., Benroubi, M., Sun, J. H., Zimmermann, A. G., and Pechtner, V. (2015) Efficacy and Safety of Once-Weekly Dulaglutide Versus Insulin Glargine in Patients With Type 2 Diabetes on Metformin and Glimepiride (AWARD-2). Diabetes Care*,* 38**,** 2241-9 10.2337/dc14-1625.

Göke, B., Gallwitz, B., Eriksson, J. G., Hellqvist, Å., and Gause-Nilsson, I. (2013) Saxagliptin vs. glipizide as add-on therapy in patients with type 2 diabetes mellitus inadequately controlled on metformin alone: long-term (52-week) extension of a 52-week randomised controlled trial. Int J Clin Pract*,* 67**,** 307-16 10.1111/ijcp.12119.

Goldstein, B. J., Feinglos, M. N., Lunceford, J. K., Johnson, J., and Williams-Herman, D. E. (2007) Effect of initial combination therapy with sitagliptin, a dipeptidyl peptidase-4 inhibitor, and metformin on glycemic control in patients with type 2 diabetes. Diabetes Care*,* 30**,** 1979-87 10.2337/dc07-0627.

Gough, S. C. L., Bode, B., Woo, V., Rodbard, H. W., Linjawi, S., Poulsen, P., Damgaard, L. H., and Buse, J. B. (2014) Efficacy and safety of a fixed-ratio combination of insulin degludec and liraglutide (IDegLira) compared with its components given alone: results of a phase 3, open-label, randomised, 26-week, treat-to-target trial in insulin-naive patients with type 2 diabetes. The Lancet Diabetes & Endocrinology*,* 2**,** 885-893 <https://doi.org/10.1016/S2213-8587(14)70174-3>.

Grey, A., Bolland, M., Fenwick, S., Horne, A., Gamble, G., Drury, P. L., and Reid, I. R. (2014) The skeletal effects of pioglitazone in type 2 diabetes or impaired glucose tolerance: a randomized controlled trial. European Journal of Endocrinology*,* 170**,** 255-262 10.1530/eje-13-0793.

Groop, P. H., Cooper, M. E., Perkovic, V., Hocher, B., Kanasaki, K., Haneda, M., Schernthaner, G., Sharma, K., Stanton, R. C., Toto, R., Cescutti, J., Gordat, M., Meinicke, T., Koitka-Weber, A., Thiemann, S., and Von Eynatten, M. (2017) Linagliptin and its effects on hyperglycaemia and albuminuria in patients with type 2 diabetes and renal dysfunction: the randomized MARLINA-T2D trial. Diabetes Obes Metab*,* 19**,** 1610-1619 10.1111/dom.13041.

Grunberger, G., Camp, S., Johnson, J., Huyck, S., Terra, S. G., Mancuso, J. P., Jiang, Z. W., Golm, G., Engel, S. S., and Lauring, B. (2018) Ertugliflozin in Patients with Stage 3 Chronic Kidney Disease and Type 2 Diabetes Mellitus: The VERTIS RENAL Randomized Study. Diabetes Ther*,* 9**,** 49-66 10.1007/s13300-017-0337-5.

Gruntmanis, U., Fordan, S., Ghayee, H. K., Abdullah, S. M., See, R., Ayers, C. R., and Mcguire, D. K. (2010) The Peroxisome Proliferator-Activated Receptor-γ Agonist Rosiglitazone Increases Bone Resorption in Women with Type 2 Diabetes: A Randomized, Controlled Trial. Calcified Tissue International*,* 86**,** 343-349 10.1007/s00223-010-9352-5.

Guja, C., Frías, J. P., Somogyi, A., Jabbour, S., Wang, H., Hardy, E., and Rosenstock, J. (2018) Effect of exenatide QW or placebo, both added to titrated insulin glargine, in uncontrolled type 2 diabetes: The DURATION-7 randomized study. Diabetes, Obesity and Metabolism*,* 20**,** 1602-1614 <https://doi.org/10.1111/dom.13266>.

Hadjadj, S., Rosenstock, J., Meinicke, T., Woerle, H. J., and Broedl, U. C. (2016) Initial Combination of Empagliflozin and Metformin in Patients With Type 2 Diabetes. Diabetes Care*,* 39**,** 1718-28 10.2337/dc16-0522.

Haering, H. U., Merker, L., Christiansen, A. V., Roux, F., Salsali, A., Kim, G., Meinicke, T., Woerle, H. J., and Broedl, U. C. (2015) Empagliflozin as add-on to metformin plus sulphonylurea in patients with type 2 diabetes. Diabetes Res Clin Pract*,* 110**,** 82-90 10.1016/j.diabres.2015.05.044.

Halvorsen, Y.-D., Lock, J. P., Zhou, W., Zhu, F., and Freeman, M. W. (2019) A 24-week, randomized, double-blind, active-controlled clinical trial comparing bexagliflozin with sitagliptin as an adjunct to metformin for the treatment of type 2 diabetes in adults. Diabetes, Obesity and Metabolism*,* 21**,** 2248-2256 <https://doi.org/10.1111/dom.13801>.

Halvorsen, Y. D., Conery, A. L., Lock, J. P., Zhou, W., and Freeman, M. W. (2023) Bexagliflozin as an adjunct to metformin for the treatment of type 2 diabetes in adults: A 24-week, randomized, double-blind, placebo-controlled trial. Diabetes, Obesity and Metabolism*,* 25**,** 2954-2962 10.1111/dom.15192.

Häring, H. U., Merker, L., Seewaldt-Becker, E., Weimer, M., Meinicke, T., Woerle, H. J., and Broedl, U. C. (2013) Empagliflozin as add-on to metformin plus sulfonylurea in patients with type 2 diabetes: a 24-week, randomized, double-blind, placebo-controlled trial. Diabetes Care*,* 36**,** 3396-404 10.2337/dc12-2673.

Heine, R. J., Van Gaal, L. F., Johns, D., Mihm, M. J., Widel, M. H., and Brodows, R. G. (2005) Exenatide versus insulin glargine in patients with suboptimally controlled type 2 diabetes: a randomized trial. Ann Intern Med*,* 143**,** 559-69 10.7326/0003-4819-143-8-200510180-00006.

Henry, R. R., Murray, A. V., Marmolejo, M. H., Hennicken, D., Ptaszynska, A., and List, J. F. (2012) Dapagliflozin, metformin XR, or both: initial pharmacotherapy for type 2 diabetes, a randomised controlled trial. Int J Clin Pract*,* 66**,** 446-56 10.1111/j.1742-1241.2012.02911.x.

Henry, R. R., Staels, B., Fonseca, V. A., Chou, M. Z., Teng, R., Golm, G. T., Langdon, R. B., Kaufman, K. D., Steinberg, H., and Goldstein, B. J. (2014) Efficacy and safety of initial combination treatment with sitagliptin and pioglitazone--a factorial study. Diabetes Obes Metab*,* 16**,** 223-30 10.1111/dom.12194.

Hernandez, A. F., Green, J. B., Janmohamed, S., D'agostino, R. B., Sr., Granger, C. B., Jones, N. P., Leiter, L. A., Rosenberg, A. E., Sigmon, K. N., Somerville, M. C., Thorpe, K. M., Mcmurray, J. J. V., and Del Prato, S. (2018) Albiglutide and cardiovascular outcomes in patients with type 2 diabetes and cardiovascular disease (Harmony Outcomes): a double-blind, randomised placebo-controlled trial. Lancet*,* 392**,** 1519-1529 10.1016/s0140-6736(18)32261-x.

Hirose, T., Suzuki, M., and Tsumiyama, I. (2015) Efficacy and Safety of Vildagliptin as an Add-on to Insulin with or without Metformin in Japanese Patients with Type 2 Diabetes Mellitus: A 12-week, Double-Blind, Randomized Study. Diabetes Ther*,* 6**,** 559-571 10.1007/s13300-015-0147-6.

Hollander, P., Liu, J., Hill, J., Johnson, J., Jiang, Z. W., Golm, G., Huyck, S., Terra, S. G., Mancuso, J. P., Engel, S. S., and Lauring, B. (2018) Ertugliflozin Compared with Glimepiride in Patients with Type 2 Diabetes Mellitus Inadequately Controlled on Metformin: The VERTIS SU Randomized Study. Diabetes Ther*,* 9**,** 193-207 10.1007/s13300-017-0354-4.

Hollander, P. L., Jia, L., Frederich, R., Allen, E., and Chen, R. (2011) Safety and efficacy of saxagliptin added to thiazolidinedione over 76 weeks in patients with type 2 diabetes mellitus. Diabetes and Vascular Disease Research*,* 8**,** 125-135 10.1177/1479164111404575.

Home, P., Shankar, R. R., Gantz, I., Iredale, C., O'neill, E. A., Jain, L., Pong, A., Suryawanshi, S., Engel, S. S., Kaufman, K. D., and Lai, E. (2018) A randomized, double-blind trial evaluating the efficacy and safety of monotherapy with the once-weekly dipeptidyl peptidase-4 inhibitor omarigliptin in people with type 2 diabetes. Diabetes Res Clin Pract*,* 138**,** 253-261 10.1016/j.diabres.2017.10.018.

Home, P. D., Pocock, S. J., Beck-Nielsen, H., Curtis, P. S., Gomis, R., Hanefeld, M., Jones, N. P., Komajda, M., and Mcmurray, J. J. (2009) Rosiglitazone evaluated for cardiovascular outcomes in oral agent combination therapy for type 2 diabetes (RECORD): a multicentre, randomised, open-label trial. Lancet*,* 373**,** 2125-35 10.1016/s0140-6736(09)60953-3.

Home, P. D., Shamanna, P., Stewart, M., Yang, F., Miller, M., Perry, C., and Carr, M. C. (2015) Efficacy and tolerability of albiglutide versus placebo or pioglitazone over 1 year in people with type 2 diabetes currently taking metformin and glimepiride: HARMONY 5. Diabetes Obes Metab*,* 17**,** 179-87 10.1111/dom.12414.

Hong, S. M., Park, C. Y., Hwang, D. M., Han, K. A., Lee, C. B., Chung, C. H., Yoon, K. H., Mok, J. O., Park, K. S., and Park, S. W. (2017) Efficacy and safety of adding evogliptin versus sitagliptin for metformin-treated patients with type 2 diabetes: A 24-week randomized, controlled trial with open label extension. Diabetes, Obesity and Metabolism*,* 19**,** 654-663 10.1111/dom.12870.

Husain, M., Birkenfeld, A. L., Donsmark, M., Dungan, K., Eliaschewitz, F. G., Franco, D. R., Jeppesen, O. K., Lingvay, I., Mosenzon, O., Pedersen, S. D., Tack, C. J., Thomsen, M., Vilsbøll, T., Warren, M. L., and Bain, S. C. (2019) Oral Semaglutide and Cardiovascular Outcomes in Patients with Type 2 Diabetes. N Engl J Med*,* 381**,** 841-851 10.1056/NEJMoa1901118.

Hygum, K., Harsløf, T., Jørgensen, N. R., Rungby, J., Pedersen, S. B., and Langdahl, B. L. (2020) Bone resorption is unchanged by liraglutide in type 2 diabetes patients: A randomised controlled trial. Bone*,* 132**,** 115197 10.1016/j.bone.2019.115197.

Inagaki, N., Atsumi, Y., Oura, T., Saito, H., and Imaoka, T. (2012) Efficacy and Safety Profile of Exenatide Once Weekly Compared With Insulin Once Daily in Japanese Patients With Type 2 Diabetes Treated With Oral Antidiabetes Drug(s): Results From a 26-Week, Randomized, Open-Label, Parallel-Group, Multicenter, Noninferiority Study. Clinical Therapeutics*,* 34**,** 1892-1908.e1 <https://doi.org/10.1016/j.clinthera.2012.07.007>.

Inagaki, N., Kondo, K., Yoshinari, T., Takahashi, N., Susuta, Y., and Kuki, H. (2014) Efficacy and safety of canagliflozin monotherapy in Japanese patients with type 2 diabetes inadequately controlled with diet and exercise: a 24-week, randomized, double-blind, placebo-controlled, Phase III study. Expert Opinion on Pharmacotherapy*,* 15**,** 1501-1515 10.1517/14656566.2014.935764.

Iwamoto, Y., Tajima, N., Kadowaki, T., Nonaka, K., Taniguchi, T., Nishii, M., Arjona Ferreira, J. C., and Amatruda, J. M. (2010) Efficacy and safety of sitagliptin monotherapy compared with voglibose in Japanese patients with type 2 diabetes: a randomized, double-blind trial. Diabetes, Obesity and Metabolism*,* 12**,** 613-622 <https://doi.org/10.1111/j.1463-1326.2010.01197.x>.

Jabbour, S. A., Hardy, E., Sugg, J., Parikh, Shamik, and Group, F. T. S. (2014) Dapagliflozin Is Effective as Add-on Therapy to Sitagliptin With or Without Metformin: A 24-Week, Multicenter, Randomized, Double-Blind, Placebo-Controlled Study. Diabetes Care*,* 37**,** 740-750 10.2337/dc13-0467.

Jadzinsky, M., Pfützner, A., Paz-Pacheco, E., Xu, Z., Allen, E., and Chen, R. (2009) Saxagliptin given in combination with metformin as initial therapy improves glycaemic control in patients with type 2 diabetes compared with either monotherapy: a randomized controlled trial. Diabetes Obes Metab*,* 11**,** 611-22 10.1111/j.1463-1326.2009.01056.x.

Jaiswal, M., Martin, C. L., Brown, M. B., Callaghan, B., Albers, J. W., Feldman, E. L., and Pop-Busui, R. (2015) Effects of exenatide on measures of diabetic neuropathy in subjects with type 2 diabetes: results from an 18-month proof-of-concept open-label randomized study. Journal of Diabetes and its Complications*,* 29**,** 1287-1294 <https://doi.org/10.1016/j.jdiacomp.2015.07.013>.

Ji, L., Liu, Y., Miao, H., Xie, Y., Yang, M., Wang, W., Mu, Y., Yan, P., Pan, S., Lauring, B., Liu, S., Huyck, S., Qiu, Y., and Terra, S. G. (2019) Safety and efficacy of ertugliflozin in Asian patients with type 2 diabetes mellitus inadequately controlled with metformin monotherapy: VERTIS Asia. Diabetes Obes Metab*,* 21**,** 1474-1482 10.1111/dom.13681.

Ji, L., Ma, J., Li, H., Mansfield, T. A., T'joen C, L., Iqbal, N., Ptaszynska, A., and List, J. F. (2014) Dapagliflozin as monotherapy in drug-naive Asian patients with type 2 diabetes mellitus: a randomized, blinded, prospective phase III study. Clin Ther*,* 36**,** 84-100.e9 10.1016/j.clinthera.2013.11.002.

Ji, L., Zinman, B., Patel, S., Ji, J., Bailes, Z., Thiemann, S., and Seck, T. (2015) Efficacy and safety of linagliptin co-administered with low-dose metformin once daily versus high-dose metformin twice daily in treatment-naïve patients with type 2 diabetes: a double-blind randomized trial. Adv Ther*,* 32**,** 201-15 10.1007/s12325-015-0195-3.

Kadowaki, T., Inagaki, N., Kondo, K., Nishimura, K., Kaneko, G., Maruyama, N., Nakanishi, N., Iijima, H., Watanabe, Y., and Gouda, M. (2017) Efficacy and safety of canagliflozin as add-on therapy to teneligliptin in Japanese patients with type 2 diabetes mellitus: Results of a 24-week, randomized, double-blind, placebo-controlled trial. Diabetes, Obesity and Metabolism*,* 19**,** 874-882 10.1111/dom.12898.

Kahn, S. E., Haffner, S. M., Heise, M. A., Herman, W. H., Holman, R. R., Jones, N. P., Kravitz, B. G., Lachin, J. M., O'neill, M. C., Zinman, B., and Viberti, G. (2006) Glycemic Durability of Rosiglitazone, Metformin, or Glyburide Monotherapy. New England Journal of Medicine*,* 355**,** 2427-2443 10.1056/NEJMoa066224.

Kaku, K., Araki, E., Tanizawa, Y., Ross Agner, B., Nishida, T., Ranthe, M., and Inagaki, N. (2019a) Superior efficacy with a fixed-ratio combination of insulin degludec and liraglutide (IDegLira) compared with insulin degludec and liraglutide in insulin-naïve Japanese patients with type 2 diabetes in a phase 3, open-label, randomized trial. Diabetes Obes Metab*,* 21**,** 2674-2683 10.1111/dom.13856.

Kaku, K., Haneda, M., Tanaka, Y., Lee, G., Shiki, K., Miyamoto, Y., Solimando, F., Lee, J., Lee, C., and George, J. (2019b) Linagliptin as add-on to empagliflozin in a fixed-dose combination in Japanese patients with type 2 diabetes: Glycaemic efficacy and safety profile in a two-part, randomized, placebo-controlled trial. Diabetes Obes Metab*,* 21**,** 136-145 10.1111/dom.13496.

Kanazawa, I., Yamaguchi, T., Yano, S., Yamamoto, M., Yamauchi, M., Kurioka, S., and Sugimoto, T. (2010) Baseline atherosclerosis parameter could assess the risk of bone loss during pioglitazone treatment in type 2 diabetes mellitus. Osteoporos Int*,* 21**,** 2013-8 10.1007/s00198-009-1161-1.

Kawamori, R., Haneda, M., Suzaki, K., Cheng, G., Shiki, K., Miyamoto, Y., Solimando, F., Lee, C., Lee, J., and George, J. (2018) Empagliflozin as add-on to linagliptin in a fixed-dose combination in Japanese patients with type 2 diabetes: Glycaemic efficacy and safety profile in a 52-week, randomized, placebo-controlled trial. Diabetes Obes Metab*,* 20**,** 2200-2209 10.1111/dom.13352.

Kim, H. J., Jeong, I. K., Hur, K. Y., Kim, S. K., Noh, J. H., Chun, S. W., Kang, E. S., Rhee, E. J., and Choi, S. H. (2022) Comparison of Efficacy of Glimepiride, Alogliptin, and Alogliptin-Pioglitazone as the Initial Periods of Therapy in Patients with Poorly Controlled Type 2 Diabetes Mellitus: An Open-Label, Multicenter, Randomized, Controlled Study. Diabetes and Metabolism Journal*,* 46**,** 689-700 10.4093/dmj.2021.0183.

Kim, J. M., Kim, S. S., Kim, J. H., Kim, M. K., Kim, T. N., Lee, S. H., Lee, C. W., Park, J. Y., Kim, E. S., Lee, K. J., Choi, Y. S., Kim, D. K., and Kim, I. J. (2020) Efficacy and safety of pioglitazone versus glimepiride after metformin and alogliptin combination therapy: A randomized, open-label, multicenter, parallel-controlled study. Diabetes and Metabolism Journal*,* 44**,** 67-77 10.4093/DMJ.2018.0274.

Kim, K. S., Han, K. A., Kim, T. N., Park, C. Y., Park, J. H., Kim, S. Y., Kim, Y. H., Song, K. H., Kang, E. S., Kim, C. S., Koh, G., Kang, J. G., Kim, M. K., Han, J. M., Kim, N. H., Mok, J. O., Lee, J. H., Lim, S., Kim, S. S., Kim, T. H., Won, K. C., Lee, K. Y., Cho, J. H., Han, J. Y., Kim, S. H., Nah, J. J., Song, H. R., Lee, S. E., and Kim, S. (2023) Efficacy and safety of enavogliflozin versus dapagliflozin added to metformin plus gemigliptin treatment in patients with type 2 diabetes: A double-blind, randomized, comparator-active study: ENHANCE-D study. Diabetes and Metabolism*,* 49 10.1016/j.diabet.2023.101440.

Kovacs, C. S., Seshiah, V., Merker, L., Christiansen, A. V., Roux, F., Salsali, A., Kim, G., Stella, P., Woerle, H.-J., and Broedl, U. C. (2015) Empagliflozin as Add-on Therapy to Pioglitazone With or Without Metformin in Patients With Type 2 Diabetes Mellitus. Clinical Therapeutics*,* 37**,** 1773-1788.e1 <https://doi.org/10.1016/j.clinthera.2015.05.511>.

Kwak, S. H., Han, K. A., Kim, K. S., Yu, J. M., Kim, E., Won, J. C., Kang, J. G., Chung, C. H., Oh, S., Choi, S. H., Won, K. C., Kim, S. G., Cho, S. A., Cho, B. Y., and Park, K. S. (2023) Efficacy and safety of enavogliflozin, a novel SGLT2 inhibitor, in Korean people with type 2 diabetes: A 24-week, multicentre, randomized, double-blind, placebo-controlled, phase III trial. Diabetes, Obesity and Metabolism*,* 25**,** 1865-1873 10.1111/dom.15046.

Lavalle-González, F. J., Januszewicz, A., Davidson, J., Tong, C., Qiu, R., Canovatchel, W., and Meininger, G. (2013) Efficacy and safety of canagliflozin compared with placebo and sitagliptin in patients with type 2 diabetes on background metformin monotherapy: a randomised trial. Diabetologia*,* 56**,** 2582-92 10.1007/s00125-013-3039-1.

Leiter, L. A., Carr, M. C., Stewart, M., Jones-Leone, A., Scott, R., Yang, F., and Handelsman, Y. (2014) Efficacy and Safety of the Once-Weekly GLP-1 Receptor Agonist Albiglutide Versus Sitagliptin in Patients With Type 2 Diabetes and Renal Impairment: A Randomized Phase III Study. Diabetes Care*,* 37**,** 2723-2730 10.2337/dc13-2855.

Lingvay, I., Catarig, A. M., Frias, J. P., Kumar, H., Lausvig, N. L., Le Roux, C. W., Thielke, D., Viljoen, A., and Mccrimmon, R. J. (2019) Efficacy and safety of once-weekly semaglutide versus daily canagliflozin as add-on to metformin in patients with type 2 diabetes (SUSTAIN 8): a double-blind, phase 3b, randomised controlled trial. Lancet Diabetes Endocrinol*,* 7**,** 834-844 10.1016/s2213-8587(19)30311-0.

Ljunggren, Ö., Bolinder, J., Johansson, L., Wilding, J., Langkilde, A. M., Sjöström, C. D., Sugg, J., and Parikh, S. (2012) Dapagliflozin has no effect on markers of bone formation and resorption or bone mineral density in patients with inadequately controlled type 2 diabetes mellitus on metformin. Diabetes Obes Metab*,* 14**,** 990-9 10.1111/j.1463-1326.2012.01630.x.

Lu, C. H., Min, K. W., Chuang, L. M., Kokubo, S., Yoshida, S., and Cha, B. S. (2016) Efficacy, safety, and tolerability of ipragliflozin in Asian patients with type 2 diabetes mellitus and inadequate glycemic control with metformin: Results of a phase 3 randomized, placebo-controlled, double-blind, multicenter trial. Journal of Diabetes Investigation*,* 7**,** 366-373 10.1111/jdi.12422.

Ludvik, B., Frías, J. P., Tinahones, F. J., Wainstein, J., Jiang, H., Robertson, K. E., García-Pérez, L.-E., Woodward, D. B., and Milicevic, Z. (2018) Dulaglutide as add-on therapy to SGLT2 inhibitors in patients with inadequately controlled type 2 diabetes (AWARD-10): a 24-week, randomised, double-blind, placebo-controlled trial. The Lancet Diabetes & Endocrinology*,* 6**,** 370-381 <https://doi.org/10.1016/S2213-8587(18)30023-8>.

Marso, S. P., Bain, S. C., Consoli, A., Eliaschewitz, F. G., Jódar, E., Leiter, L. A., Lingvay, I., Rosenstock, J., Seufert, J., Warren, M. L., Woo, V., Hansen, O., Holst, A. G., Pettersson, J., and Vilsbøll, T. (2016a) Semaglutide and Cardiovascular Outcomes in Patients with Type 2 Diabetes. New England Journal of Medicine*,* 375**,** 1834-1844 10.1056/NEJMoa1607141.

Marso, S. P., Daniels, G. H., Brown-Frandsen, K., Kristensen, P., Mann, J. F. E., Nauck, M. A., Nissen, S. E., Pocock, S., Poulter, N. R., Ravn, L. S., Steinberg, W. M., Stockner, M., Zinman, B., Bergenstal, R. M., and Buse, J. B. (2016b) Liraglutide and Cardiovascular Outcomes in Type 2 Diabetes. New England Journal of Medicine*,* 375**,** 311-322 10.1056/NEJMoa1603827.

Mathieu, C., Ranetti, A. E., Li, D., Ekholm, E., Cook, W., Hirshberg, B., Chen, H., Hansen, L., and Iqbal, N. (2015a) Randomized, Double-Blind, Phase 3 Trial of Triple Therapy With Dapagliflozin Add-on to Saxagliptin Plus Metformin in Type 2 Diabetes. Diabetes Care*,* 38**,** 2009-2017 10.2337/dc15-0779.

Mathieu, C., Rodbard, H. W., Cariou, B., Handelsman, Y., Philis-Tsimikas, A., Ocampo Francisco, A. M., Rana, A., Zinman, B., and Group, O. B. O. T. B. V. a.-O. S. (2014) A comparison of adding liraglutide versus a single daily dose of insulin aspart to insulin degludec in subjects with type 2 diabetes (BEGIN: VICTOZA ADD-ON). Diabetes, Obesity and Metabolism*,* 16**,** 636-644 <https://doi.org/10.1111/dom.12262>.

Mathieu, C., Shankar, R. R., Lorber, D., Umpierrez, G., Wu, F., Xu, L., Golm, G. T., Latham, M., Kaufman, K. D., and Engel, S. S. (2015b) A Randomized Clinical Trial to Evaluate the Efficacy and Safety of Co-Administration of Sitagliptin with Intensively Titrated Insulin Glargine. Diabetes Ther*,* 6**,** 127-42 10.1007/s13300-015-0105-3.

Matthaei, S., Catrinoiu, D., Celiński, A., Ekholm, E., Cook, W., Hirshberg, B., Chen, H., Iqbal, N., and Hansen, L. (2015) Randomized, Double-Blind trial of triple therapy with saxagliptin add-on to dapagliflozin plus metformin inpatientswith type 2 Diabetes. Diabetes Care*,* 38**,** 2018-2024 10.2337/dc15-0811.

Matthews, D. R., Paldánius, P. M., Proot, P., Chiang, Y., Stumvoll, M., and Del Prato, S. (2019) Glycaemic durability of an early combination therapy with vildagliptin and metformin versus sequential metformin monotherapy in newly diagnosed type 2 diabetes (VERIFY): a 5-year, multicentre, randomised, double-blind trial. Lancet*,* 394**,** 1519-1529 10.1016/s0140-6736(19)32131-2.

Meneilly, G. S., Roy-Duval, C., Alawi, H., Dailey, G., Bellido, D., Trescoli, C., Manrique Hurtado, H., Guo, H., Pilorget, V., Perfetti, R., Simpson, H., and Investigators, O. B. O. T. G.-O. T. (2017) Lixisenatide Therapy in Older Patients With Type 2 Diabetes Inadequately Controlled on Their Current Antidiabetic Treatment: The GetGoal-O Randomized Trial. Diabetes Care*,* 40**,** 485-493 10.2337/dc16-2143.

Moses, R. G., Round, E., Shentu, Y., Golm, G. T., O'neill, E. A., Gantz, I., Engel, S. S., Kaufman, K. D., and Goldstein, B. J. (2016) A randomized clinical trial evaluating the safety and efficacy of sitagliptin added to the combination of sulfonylurea and metformin in patients with type 2 diabetes mellitus and inadequate glycemic control. Journal of Diabetes*,* 8**,** 701-711 <https://doi.org/10.1111/1753-0407.12351>.

Mu, Y., Pan, C., Fan, B., Hehnke, U., Zhang, X., Zhang, X., Wang, X., Liu, J., Zhang, Y., Du, J., Ma, J., and Gong, Y. (2017) Efficacy and safety of linagliptin/metformin single-pill combination as initial therapy in drug-naïve Asian patients with type 2 diabetes. Diabetes Research and Clinical Practice*,* 124**,** 48-56 <https://doi.org/10.1016/j.diabres.2016.11.026>.

Nauck, M., Frid, A., Hermansen, K., Shah, N. S., Tankova, T., Mitha, I. H., Zdravkovic, M., Düring, M., and Matthews, D. R. (2009) Efficacy and safety comparison of liraglutide, glimepiride, and placebo, all in combination with metformin, in type 2 diabetes: the LEAD (liraglutide effect and action in diabetes)-2 study. Diabetes Care*,* 32**,** 84-90 10.2337/dc08-1355.

Nauck, M. A., Del Prato, S., Meier, J. J., Durán-García, S., Rohwedder, K., Elze, M., and Parikh, S. J. (2011) Dapagliflozin versus glipizide as add-on therapy in patients with type 2 diabetes who have inadequate glycemic control with metformin: a randomized, 52-week, double-blind, active-controlled noninferiority trial. Diabetes Care*,* 34**,** 2015-22 10.2337/dc11-0606.

Nauck, M. A., Duran, S., Kim, D., Johns, D., Northrup, J., Festa, A., Brodows, R., and Trautmann, M. (2007) A comparison of twice-daily exenatide and biphasic insulin aspart in patients with type 2 diabetes who were suboptimally controlled with sulfonylurea and metformin: a non-inferiority study. Diabetologia*,* 50**,** 259-267 10.1007/s00125-006-0510-2.

Nauck, M. A., Stewart, M. W., Perkins, C., Jones-Leone, A., Yang, F., Perry, C., Reinhardt, R. R., and Rendell, M. (2016) Efficacy and safety of once-weekly GLP-1 receptor agonist albiglutide (HARMONY 2): 52 week primary endpoint results from a randomised, placebo-controlled trial in patients with type 2 diabetes mellitus inadequately controlled with diet and exercise. Diabetologia*,* 59**,** 266-274 10.1007/s00125-015-3795-1.

Nct00086515 (2007) NCT00086515.

Nct00116831 (2008) NCT00116831.

Nct00121667 (2010) NCT00121667.

Nct00313313 (2008) NCT00313313.

Nct00367055 (2008) NCT00367055.

Nct00372060 (2008) NCT00372060.

Nct00374907 (2009) NCT00374907.

Nct00434954 (2010) NCT00434954.

Nct00484198 (2012) NCT00484198.

Nct00509236 (2013) NCT00509236.

Nct00601250 (2009) NCT00601250.

Nct00614120 (2009) NCT00614120.

Nct00736879 (2009) NCT00736879.

Nct00798161 (2014) NCT00798161.

Nct00800683 (2011) NCT00800683.

Nct00856284 (2012) NCT00856284.

Nct00859898 (2014) NCT00859898.

Nct00915772 (2011) NCT00915772.

Nct01042977 (2014) NCT01042977.

Nct01076088 (2014) NCT01076088.

Nct01183013 (2014) NCT01183013.

Nct01204294 (2012) NCT01204294.

Nct01215097 (2013) NCT01215097.

Nct01296412 (2012) NCT01296412.

Nct01453049 (2010) NCT01453049.

Nct01545388 (2012) NCT01545388.

Nct01558271 (2014) NCT01558271.

Nct01644500 (2014) NCT01644500.

Nct01648582 (2014) NCT01648582.

Nct01733758 (2015) NCT01733758.

Nct02471404 (2017) NCT02471404.

Nct02564926 (2018) NCT02564926.

Nct02589639 (2018) NCT02589639.

Nct02681094 (2017) NCT02681094.

Nct02906709 (2018) NCT02906709.

Nct02912455 (2020) NCT02912455.

Nct03386344 (2020) NCT03386344.

Nct04537923 (2022) NCT04537923.

Nct04591626 (2022) NCT04591626.

Neal, B., Perkovic, V., De Zeeuw, D., Mahaffey, K. W., Fulcher, G., Stein, P., Desai, M., Shaw, W., Jiang, J., Vercruysse, F., Meininger, G., and Matthews, D. (2013) Rationale, design, and baseline characteristics of the Canagliflozin Cardiovascular Assessment Study (CANVAS)--a randomized placebo-controlled trial. Am Heart J*,* 166**,** 217-223.e11 10.1016/j.ahj.2013.05.007.

Nissen, S. E., Nicholls, S. J., Wolski, K., Nesto, R., Kupfer, S., Perez, A., Jure, H., De Larochellière, R., Staniloae, C. S., Mavromatis, K., Saw, J., Hu, B., Lincoff, A. M., Tuzcu, E. M., and Periscope Investigators, F. T. (2008) Comparison of Pioglitazone vs Glimepiride on Progression of Coronary Atherosclerosis in Patients With Type 2 Diabetes: The PERISCOPE Randomized Controlled Trial. JAMA*,* 299**,** 1561-1573 10.1001/jama.299.13.1561.

Nordklint, A. K., Almdal, T. P., Vestergaard, P., Lundby-Christensen, L., Boesgaard, T. W., Breum, L., Gade-Rasmussen, B., Sneppen, S. B., Gluud, C., Hemmingsen, B., Jensen, T., Krarup, T., Madsbad, S., Mathiesen, E. R., Perrild, H., Tarnow, L., Thorsteinsson, B., Vestergaard, H., Lund, S. S., and Eiken, P. (2018) The effect of metformin versus placebo in combination with insulin analogues on bone mineral density and trabecular bone score in patients with type 2 diabetes mellitus: a randomized placebo-controlled trial. Osteoporos Int*,* 29**,** 2517-2526 10.1007/s00198-018-4637-z.

Nowicki, M., Rychlik, I., Haller, H., Warren, M. L., Suchower, L., and Gause-Nilsson, I. (2011) Saxagliptin improves glycaemic control and is well tolerated in patients with type 2 diabetes mellitus and renal impairment. Diabetes Obes Metab*,* 13**,** 523-32 10.1111/j.1463-1326.2011.01382.x.

Owens, D. R., Swallow, R., Dugi, K. A., and Woerle, H. J. (2011) Efficacy and safety of linagliptin in persons with type 2 diabetes inadequately controlled by a combination of metformin and sulphonylurea: a 24-week randomized study. Diabet Med*,* 28**,** 1352-61 10.1111/j.1464-5491.2011.03387.x.

Perez, A., Zhao, Z., Jacks, R., and Spanheimer, R. (2009) Efficacy and safety of pioglitazone/metformin fixed-dose combination therapy compared with pioglitazone and metformin monotherapy in treating patients with T2DM. Curr Med Res Opin*,* 25**,** 2915-23 10.1185/03007990903350011.

Perkovic, V., Jardine, M. J., Neal, B., Bompoint, S., Heerspink, H. J. L., Charytan, D. M., Edwards, R., Agarwal, R., Bakris, G., Bull, S., Cannon, C. P., Capuano, G., Chu, P. L., De Zeeuw, D., Greene, T., Levin, A., Pollock, C., Wheeler, D. C., Yavin, Y., Zhang, H., Zinman, B., Meininger, G., Brenner, B. M., and Mahaffey, K. W. (2019) Canagliflozin and Renal Outcomes in Type 2 Diabetes and Nephropathy. N Engl J Med*,* 380**,** 2295-2306 10.1056/NEJMoa1811744.

Pfeffer, M. A., Claggett, B., Diaz, R., Dickstein, K., Gerstein, H. C., Køber, L. V., Lawson, F. C., Ping, L., Wei, X., Lewis, E. F., Maggioni, A. P., Mcmurray, J. J., Probstfield, J. L., Riddle, M. C., Solomon, S. D., and Tardif, J. C. (2015) Lixisenatide in Patients with Type 2 Diabetes and Acute Coronary Syndrome. N Engl J Med*,* 373**,** 2247-57 10.1056/NEJMoa1509225.

Pieber, T. R., Bode, B., Mertens, A., Cho, Y. M., Christiansen, E., Hertz, C. L., Wallenstein, S. O. R., and Buse, J. B. (2019) Efficacy and safety of oral semaglutide with flexible dose adjustment versus sitagliptin in type 2 diabetes (PIONEER 7): a multicentre, open-label, randomised, phase 3a trial. Lancet Diabetes Endocrinol*,* 7**,** 528-539 10.1016/s2213-8587(19)30194-9.

Pinget, M., Goldenberg, R., Niemoeller, E., Muehlen-Bartmer, I., Guo, H., and Aronson, R. (2013) Efficacy and safety of lixisenatide once daily versus placebo in type 2 diabetes insufficiently controlled on pioglitazone (GetGoal-P). Diabetes Obes Metab*,* 15**,** 1000-7 10.1111/dom.12121.

Pratley, R., Amod, A., Hoff, S. T., Kadowaki, T., Lingvay, I., Nauck, M., Pedersen, K. B., Saugstrup, T., and Meier, J. J. (2019) Oral semaglutide versus subcutaneous liraglutide and placebo in type 2 diabetes (PIONEER 4): a randomised, double-blind, phase 3a trial. Lancet*,* 394**,** 39-50 10.1016/s0140-6736(19)31271-1.

Pratley, R. E., Eldor, R., Raji, A., Golm, G., Huyck, S. B., Qiu, Y., Sunga, S., Johnson, J., Terra, S. G., Mancuso, J. P., Engel, S. S., and Lauring, B. (2018) Ertugliflozin plus sitagliptin versus either individual agent over 52 weeks in patients with type 2 diabetes mellitus inadequately controlled with metformin: The VERTIS FACTORIAL randomized trial. Diabetes Obes Metab*,* 20**,** 1111-1120 10.1111/dom.13194.

Pratley, R. E., Fleck, P., and Wilson, C. (2014) Efficacy and safety of initial combination therapy with alogliptin plus metformin versus either as monotherapy in drug-naïve patients with type 2 diabetes: a randomized, double-blind, 6-month study. Diabetes, Obesity and Metabolism*,* 16**,** 613-621 <https://doi.org/10.1111/dom.12258>.

Pratley, R. E., Kipnes, M. S., Fleck, P. R., Wilson, C., and Mekki, Q. (2009) Efficacy and safety of the dipeptidyl peptidase-4 inhibitor alogliptin in patients with type 2 diabetes inadequately controlled by glyburide monotherapy. Diabetes Obes Metab*,* 11**,** 167-76 10.1111/j.1463-1326.2008.01016.x.

Pratley, R. E., Nauck, M., Bailey, T., Montanya, E., Cuddihy, R., Filetti, S., Thomsen, A. B., Søndergaard, R. E., and Davies, M. (2010) Liraglutide versus sitagliptin for patients with type 2 diabetes who did not have adequate glycaemic control with metformin: a 26-week, randomised, parallel-group, open-label trial. The Lancet*,* 375**,** 1447-1456 <https://doi.org/10.1016/S0140-6736(10)60307-8>.

Punthakee, Z., Bosch, J., Dagenais, G., Diaz, R., Holman, R., Probstfield, J. L., Ramachandran, A., M. C. Riddle, M. C., Rydén, L. E., Zinman, B., Afzal, A., Yusuf, S., and Gerstein, H. C. (2012) Design, history and results of the Thiazolidinedione Intervention with vitamin D Evaluation (TIDE) randomised controlled trial. Diabetologia*,* 55**,** 36-45 10.1007/s00125-011-2357-4.

Raz, I., Chen, Y., Wu, M., Hussain, S., Kaufman, K. D., Amatruda, J. M., Langdon, R. B., Stein, P. P., and Alba, M. (2008) Efficacy and safety of sitagliptin added to ongoing metformin therapy in patients with type 2 diabetes. Current Medical Research and Opinion*,* 24**,** 537-550 10.1185/030079908X260925.

Reasner, C., Olansky, L., Seck, T. L., Williams-Herman, D. E., Chen, M., Terranella, L., Johnson-Levonas, A. O., Kaufman, K. D., and Goldstein, B. J. (2011) The effect of initial therapy with the fixed-dose combination of sitagliptin and metformin compared with metformin monotherapy in patients with type 2 diabetes mellitus. Diabetes Obes Metab*,* 13**,** 644-52 10.1111/j.1463-1326.2011.01390.x.

Ridderstråle, M., Andersen, K. R., Zeller, C., Kim, G., Woerle, H. J., and Broedl, U. C. (2014) Comparison of empagliflozin and glimepiride as add-on to metformin in patients with type 2 diabetes: a 104-week randomised, active-controlled, double-blind, phase 3 trial. The Lancet Diabetes & Endocrinology*,* 2**,** 691-700 <https://doi.org/10.1016/S2213-8587(14)70120-2>.

Rodbard, H. W., Bode, B. W., Harris, S. B., Rose, L., Lehmann, L., Jarlov, H., Thurman, J., Liraglutide, T. D. a. O., and Investigators, I. D. I. T. (2017) Safety and efficacy of insulin degludec/liraglutide (IDegLira) added to sulphonylurea alone or to sulphonylurea and metformin in insulin-naïve people with Type 2 diabetes: the DUAL IV trial. Diabetic Medicine*,* 34**,** 189-196 <https://doi.org/10.1111/dme.13256>.

Rodbard, H. W., Rosenstock, J., Canani, L. H., Deerochanawong, C., Gumprecht, J., Lindberg, S., Lingvay, I., Søndergaard, A. L., Treppendahl, M. B., and Montanya, E. (2019) Oral Semaglutide Versus Empagliflozin in Patients With Type 2 Diabetes Uncontrolled on Metformin: The PIONEER 2 Trial. Diabetes Care*,* 42**,** 2272-2281 10.2337/dc19-0883.

Rodbard, H. W., Seufert, J., Aggarwal, N., Cao, A., Fung, A., Pfeifer, M., and Alba, M. (2016) Efficacy and safety of titrated canagliflozin in patients with type 2 diabetes mellitus inadequately controlled on metformin and sitagliptin. Diabetes, Obesity and Metabolism*,* 18**,** 812-819 10.1111/dom.12684.

Roden, M., Weng, J., Eilbracht, J., Delafont, B., Kim, G., Woerle, H. J., and Broedl, U. C. (2013) Empagliflozin monotherapy with sitagliptin as an active comparator in patients with type 2 diabetes: a randomised, double-blind, placebo-controlled, phase 3 trial. Lancet Diabetes Endocrinol*,* 1**,** 208-19 10.1016/s2213-8587(13)70084-6.

Rosenstock, J., Aronson, R., Grunberger, G., Hanefeld, M., Piatti, P., Serusclat, P., Cheng, X., Zhou, T., Niemoeller, E., Souhami, E., Davies, M., and Investigators, O. B. O. T. L.-O. T. (2016a) Benefits of LixiLan, a Titratable Fixed-Ratio Combination of Insulin Glargine Plus Lixisenatide, Versus Insulin Glargine and Lixisenatide Monocomponents in Type 2 Diabetes Inadequately Controlled on Oral Agents: The LixiLan-O Randomized Trial. Diabetes Care*,* 39**,** 2026-2035 10.2337/dc16-0917.

Rosenstock, J., Brazg, R., Andryuk, P. J., Lu, K., and Stein, P. (2006) Efficacy and safety of the dipeptidyl peptidase-4 inhibitor sitagliptin added to ongoing pioglitazone therapy in patients with type 2 diabetes: a 24-week, multicenter, randomized, double-blind, placebo-controlled, parallel-group study. Clin Ther*,* 28**,** 1556-68 10.1016/j.clinthera.2006.10.007.

Rosenstock, J., Fonseca, V. A., Gross, J. L., Ratner, R. E., Ahrén, B., Chow, F. C. C., Yang, F., Miller, D., Johnson, S. L., Stewart, M. W., Leiter, L. A., and Group, F. T. H. S. (2014a) Advancing Basal Insulin Replacement in Type 2 Diabetes Inadequately Controlled With Insulin Glargine Plus Oral Agents: A Comparison of Adding Albiglutide, a Weekly GLP-1 Receptor Agonist, Versus Thrice-Daily Prandial Insulin Lispro. Diabetes Care*,* 37**,** 2317-2325 10.2337/dc14-0001.

Rosenstock, J., Frias, J., Páll, D., Charbonnel, B., Pascu, R., Saur, D., Darekar, A., Huyck, S., Shi, H., Lauring, B., and Terra, S. G. (2018) Effect of ertugliflozin on glucose control, body weight, blood pressure and bone density in type 2 diabetes mellitus inadequately controlled on metformin monotherapy (VERTIS MET). Diabetes Obes Metab*,* 20**,** 520-529 10.1111/dom.13103.

Rosenstock, J., Gross, J. L., Aguilar-Salinas, C., Hissa, M., Berglind, N., Ravichandran, S., and Fleming, D. (2013a) Long-term 4-year safety of saxagliptin in drug-naive and metformin-treated patients with Type 2 diabetes. Diabet Med*,* 30**,** 1472-6 10.1111/dme.12267.

Rosenstock, J., Guerci, B., Hanefeld, M., Gentile, S., Aronson, R., Tinahones, F. J., Roy-Duval, C., Souhami, E., Wardecki, M., Ye, J., Perfetti, R., and Heller, S. (2016b) Prandial Options to Advance Basal Insulin Glargine Therapy: Testing Lixisenatide Plus Basal Insulin Versus Insulin Glulisine Either as Basal-Plus or Basal-Bolus in Type 2 Diabetes: The GetGoal Duo-2 Trial. Diabetes Care*,* 39**,** 1318-28 10.2337/dc16-0014.

Rosenstock, J., Hanefeld, M., Shamanna, P., Min, K. W., Boka, G., Miossec, P., Zhou, T., Muehlen-Bartmer, I., and Ratner, R. E. (2014b) Beneficial effects of once-daily lixisenatide on overall and postprandial glycemic levels without significant excess of hypoglycemia in type 2 diabetes inadequately controlled on a sulfonylurea with or without metformin (GetGoal-S). J Diabetes Complications*,* 28**,** 386-92 10.1016/j.jdiacomp.2014.01.012.

Rosenstock, J., Hansen, L., Zee, P., Li, Y., Cook, W., Hirshberg, B., and Iqbal, N. (2014c) Dual Add-on Therapy in Type 2 Diabetes Poorly Controlled With Metformin Monotherapy: A Randomized Double-Blind Trial of Saxagliptin Plus Dapagliflozin Addition Versus Single Addition of Saxagliptin or Dapagliflozin to Metformin. Diabetes Care*,* 38**,** 376-383 10.2337/dc14-1142.

Rosenstock, J., Inzucchi, S. E., Seufert, J., Fleck, P. R., Wilson, C. A., and Mekki, Q. (2010) Initial combination therapy with alogliptin and pioglitazone in drug-naïve patients with type 2 diabetes. Diabetes Care*,* 33**,** 2406-2408 10.2337/dc10-0159.

Rosenstock, J., Jelaska, A., Frappin, G., Salsali, A., Kim, G., Woerle, H. J., Broedl, U. C., and Investigators, O. B. O. T. E.-R. M. T. (2014d) Improved Glucose Control With Weight Loss, Lower Insulin Doses, and No Increased Hypoglycemia With Empagliflozin Added to Titrated Multiple Daily Injections of Insulin in Obese Inadequately Controlled Type 2 Diabetes. Diabetes Care*,* 37**,** 1815-1823 10.2337/dc13-3055.

Rosenstock, J., Kahn, S. E., Johansen, O. E., Zinman, B., Espeland, M. A., Woerle, H. J., Pfarr, E., Keller, A., Mattheus, M., Baanstra, D., Meinicke, T., George, J. T., Von Eynatten, M., Mcguire, D. K., and Marx, N. (2019a) Effect of Linagliptin vs Glimepiride on Major Adverse Cardiovascular Outcomes in Patients With Type 2 Diabetes: The CAROLINA Randomized Clinical Trial. Jama*,* 322**,** 1155-1166 10.1001/jama.2019.13772.

Rosenstock, J., Perkovic, V., Johansen, O. E., Cooper, M. E., Kahn, S. E., Marx, N., Alexander, J. H., Pencina, M., Toto, R. D., Wanner, C., Zinman, B., Woerle, H. J., Baanstra, D., Pfarr, E., Schnaidt, S., Meinicke, T., George, J. T., Von Eynatten, M., and Mcguire, D. K. (2019b) Effect of Linagliptin vs Placebo on Major Cardiovascular Events in Adults With Type 2 Diabetes and High Cardiovascular and Renal Risk: The CARMELINA Randomized Clinical Trial. Jama*,* 321**,** 69-79 10.1001/jama.2018.18269.

Rosenstock, J., Vico, M., Wei, L., Salsali, A., and List, J. F. (2012) Effects of dapagliflozin, an SGLT2 inhibitor, on HbA(1c), body weight, and hypoglycemia risk in patients with type 2 diabetes inadequately controlled on pioglitazone monotherapy. Diabetes Care*,* 35**,** 1473-8 10.2337/dc11-1693.

Rosenstock, J., Wilson, C., and Fleck, P. (2013b) Alogliptin versus glipizide monotherapy in elderly type 2 diabetes mellitus patients with mild hyperglycaemia: a prospective, double-blind, randomized, 1-year study. Diabetes Obes Metab*,* 15**,** 906-14 10.1111/dom.12102.

Scherbaum, W. A., Schweizer, A., Mari, A., Nilsson, P. M., Lalanne, G., Jauffret, S., and Foley, J. E. (2008) Efficacy and tolerability of vildagliptin in drug-naïve patients with type 2 diabetes and mild hyperglycaemia*. Diabetes, Obesity and Metabolism*,* 10**,** 675-682 <https://doi.org/10.1111/j.1463-1326.2008.00850.x>.

Schernthaner, G., Durán-Garcia, S., Hanefeld, M., Langslet, G., Niskanen, L., Östgren, C. J., Malvolti, E., and Hardy, E. (2015) Efficacy and tolerability of saxagliptin compared with glimepiride in elderly patients with type 2 diabetes: A randomized, controlled study (GENERATION). Diabetes, Obesity and Metabolism*,* 17**,** 630-638 10.1111/dom.12461.

Schernthaner, G., Gross, J. L., Rosenstock, J., Guarisco, M., Fu, M., Yee, J., Kawaguchi, M., Canovatchel, W., and Meininger, G. (2013) Canagliflozin compared with sitagliptin for patients with type 2 diabetes who do not have adequate glycemic control with metformin plus sulfonylurea: a 52-week randomized trial. Diabetes Care*,* 36**,** 2508-15 10.2337/dc12-2491.

Schumm-Draeger, P. M., Burgess, L., Korányi, L., Hruba, V., Hamer-Maansson, J. E., and De Bruin, T. W. (2015) Twice-daily dapagliflozin co-administered with metformin in type 2 diabetes: a 16-week randomized, placebo-controlled clinical trial. Diabetes Obes Metab*,* 17**,** 42-51 10.1111/dom.12387.

Scirica, B. M., Bhatt, D. L., Braunwald, E., Steg, P. G., Davidson, J., Hirshberg, B., Ohman, P., Frederich, R., Wiviott, S. D., Hoffman, E. B., Cavender, M. A., Udell, J. A., Desai, N. R., Mosenzon, O., Mcguire, D. K., Ray, K. K., Leiter, L. A., and Raz, I. (2013) Saxagliptin and cardiovascular outcomes in patients with type 2 diabetes mellitus. New England Journal of Medicine*,* 369**,** 1317-1326 10.1056/NEJMoa1307684.

Seck, T., Nauck, M., Sheng, D., Sunga, S., Davies, M. J., Stein, P. P., Kaufman, K. D., and Amatruda, J. M. (2010) Safety and efficacy of treatment with sitagliptin or glipizide in patients with type 2 diabetes inadequately controlled on metformin: a 2-year study. Int J Clin Pract*,* 64**,** 562-76 10.1111/j.1742-1241.2010.02353.x.

Shankar, R. R., Bao, Y., Han, P., Hu, J., Ma, J., Peng, Y., Wu, F., Xu, L., Engel, S. S., and Jia, W. (2017a) Sitagliptin added to stable insulin therapy with or without metformin in Chinese patients with type 2 diabetes. J Diabetes Investig*,* 8**,** 321-329 10.1111/jdi.12585.

Shankar, R. R., Inzucchi, S. E., Scarabello, V., Gantz, I., Kaufman, K. D., Lai, E., Ceesay, P., Suryawanshi, S., and Engel, S. S. (2017b) A randomized clinical trial evaluating the efficacy and safety of the once-weekly dipeptidyl peptidase-4 inhibitor omarigliptin in patients with type 2 diabetes inadequately controlled on metformin monotherapy. Curr Med Res Opin*,* 33**,** 1853-1860 10.1080/03007995.2017.1335637.

Sheu, W. H., Park, S. W., Gong, Y., Pinnetti, S., Bhattacharya, S., Patel, S., Seck, T., and Woerle, H. J. (2015) Linagliptin improves glycemic control after 1 year as add-on therapy to basal insulin in Asian patients with type 2 diabetes mellitus. Curr Med Res Opin*,* 31**,** 503-12 10.1185/03007995.2015.1010638.

Søfteland, E., Meier, J. J., Vangen, B., Toorawa, R., Maldonado-Lutomirsky, M., and Broedl, U. C. (2017) Empagliflozin as Add-on Therapy in Patients With Type 2 Diabetes Inadequately Controlled With Linagliptin and Metformin: A 24-Week Randomized, Double-Blind, Parallel-Group Trial. Diabetes Care*,* 40**,** 201-209 10.2337/dc16-1347.

Stenlöf, K., Cefalu, W. T., Kim, K. A., Jodar, E., Alba, M., Edwards, R., Tong, C., Canovatchel, W., and Meininger, G. (2014) Long-term efficacy and safety of canagliflozin monotherapy in patients with type 2 diabetes inadequately controlled with diet and exercise: findings from the 52-week CANTATA-M study. Curr Med Res Opin*,* 30**,** 163-75 10.1185/03007995.2013.850066.

Strojek, K., Yoon, K. H., Hruba, V., Elze, M., Langkilde, A. M., and Parikh, S. (2011) Effect of dapagliflozin in patients with type 2 diabetes who have inadequate glycaemic control with glimepiride: a randomized, 24-week, double-blind, placebo-controlled trial. Diabetes, Obesity and Metabolism*,* 13**,** 928-938 <https://doi.org/10.1111/j.1463-1326.2011.01434.x>.

Suzuki, S., Oura, T., Takeuchi, M., and Boye, K. S. (2017) Evaluation of the impact of once weekly dulaglutide on patient-reported outcomes in Japanese patients with type 2 diabetes: comparisons with liraglutide, insulin glargine, and placebo in two randomized studies. Health Qual Life Outcomes*,* 15**,** 123 10.1186/s12955-017-0696-7.

Terauchi, Y., Tamura, M., Senda, M., Gunji, R., and Kaku, K. (2018) Long-term safety and efficacy of tofogliflozin as add-on to insulin in patients with type 2 diabetes: Results from a 52-week, multicentre, randomized, double-blind, open-label extension, Phase 4 study in Japan (J-STEP/INS). Diabetes, Obesity and Metabolism*,* 20**,** 1176-1185 10.1111/dom.13213.

Terauchi, Y., Yamada, Y., Ishida, H., Ohsugi, M., Kitaoka, M., Satoh, J., Yabe, D., Shihara, N., and Seino, Y. (2017) Efficacy and safety of sitagliptin as compared with glimepiride in Japanese patients with type 2 diabetes mellitus aged ≥ 60 years (START-J trial). Diabetes Obes Metab*,* 19**,** 1188-1192 10.1111/dom.12933.

Tolman, K. G., Freston, J. W., Kupfer, S., and Perez, A. (2009) Liver safety in patients with type 2 diabetes treated with pioglitazone: results from a 3-year, randomized, comparator-controlled study in the US. Drug Saf*,* 32**,** 787-800 10.2165/11316510-000000000-00000.

Tuttle, K. R., Lakshmanan, M. C., Rayner, B., Busch, R. S., Zimmermann, A. G., Woodward, D. B., and Botros, F. T. (2018) Dulaglutide versus insulin glargine in patients with type 2 diabetes and moderate-to-severe chronic kidney disease (AWARD-7): a multicentre, open-label, randomised trial. The Lancet Diabetes & Endocrinology*,* 6**,** 605-617 <https://doi.org/10.1016/S2213-8587(18)30104-9>.

Umpierrez, G., Tofé Povedano, S., Pérez Manghi, F., Shurzinske, L., and Pechtner, V. (2014) Efficacy and safety of dulaglutide monotherapy versus metformin in type 2 diabetes in a randomized controlled trial (AWARD-3). Diabetes Care*,* 37**,** 2168-76 10.2337/dc13-2759.

Vaccaro, O., Masulli, M., Nicolucci, A., Bonora, E., Del Prato, S., Maggioni, A. P., Rivellese, A. A., Squatrito, S., Giorda, C. B., Sesti, G., Mocarelli, P., Lucisano, G., Sacco, M., Signorini, S., Cappellini, F., Perriello, G., Babini, A. C., Lapolla, A., Gregori, G., Giordano, C., Corsi, L., Buzzetti, R., Clemente, G., Di Cianni, G., Iannarelli, R., Cordera, R., La Macchia, O., Zamboni, C., Scaranna, C., Boemi, M., Iovine, C., Lauro, D., Leotta, S., Dall'aglio, E., Cannarsa, E., Tonutti, L., Pugliese, G., Bossi, A. C., Anichini, R., Dotta, F., Di Benedetto, A., Citro, G., Antenucci, D., Ricci, L., Giorgino, F., Santini, C., Gnasso, A., De Cosmo, S., Zavaroni, D., Vedovato, M., Consoli, A., Calabrese, M., Di Bartolo, P., Fornengo, P., Riccardi, G., Vaccaro, O., Masulli, M., Nicolucci, A., Maggioni, A. P., Sesti, G., Mocarelli, P., Lucisano, G., Sacco, M., Signorini, S., Cappellini, F., Riccardi, G., Boemi, M., D'angelo, F., Giansanti, R., Tanase, L., Lanari, L., Testa, I., Ricci, L., Pancani, F., Ranchelli, A., Vagheggi, P., Scatona, A., Fontana, L., Giorgino, F., Laviola, L., Tarantino, L., Ippolito, C., Gigantelli, V., Manicone, M., Conte, E., Trevisan, R., Scaranna, C., Rota, R., Corsi, A., Dodesini, A. R., Reggiani, G. M., Montesi, L., Mazzella, N., Forlani, G., Caselli, C., Di Luzio, R., Mazzotti, A., Aiello, A., Barrea, A., Musto, A., et al. (2017) Effects on the incidence of cardiovascular events of the addition of pioglitazone versus sulfonylureas in patients with type 2 diabetes inadequately controlled with metformin (TOSCA.IT): a randomised, multicentre trial. The Lancet Diabetes & Endocrinology*,* 5**,** 887-897 <https://doi.org/10.1016/S2213-8587(17)30317-0>.

Vianna, A. G. D., De Lacerda, C. S., Pechmann, L. M., Polesel, M. G., Marino, E. C., Borba, V. Z. C., and Barreto, F. C. (2017) Vildagliptin has the same safety profile as a sulfonylurea on bone metabolism and bone mineral density in post-menopausal women with type 2 diabetes: a randomized controlled trial. Diabetol Metab Syndr*,* 9**,** 35 10.1186/s13098-017-0232-2.

Vilsbøll, T., Rosenstock, J., Yki-Järvinen, H., Cefalu, W. T., Chen, Y., Luo, E., Musser, B., Andryuk, P. J., Ling, Y., Kaufman, K. D., Amatruda, J. M., Engel, S. S., and Katz, L. (2010) Efficacy and safety of sitagliptin when added to insulin therapy in patients with type 2 diabetes. Diabetes Obes Metab*,* 12**,** 167-77 10.1111/j.1463-1326.2009.01173.x.

Wada, T., Mori-Anai, K., Takahashi, A., Matsui, T., Inagaki, M., Iida, M., Maruyama, K., and Tsuda, H. (2022) Effect of canagliflozin on the decline of estimated glomerular filtration rate in chronic kidney disease patients with type 2 diabetes mellitus: A multicenter, randomized, double-blind, placebo-controlled, parallel-group, phase III study in Japan. Journal of Diabetes Investigation*,* 13**,** 1981-1989 10.1111/jdi.13888.

Weber, M. A., Mansfield, T. A., Alessi, F., Iqbal, N., Parikh, S., and Ptaszynska, A. (2016a) Effects of dapagliflozin on blood pressure in hypertensive diabetic patients on renin-angiotensin system blockade. Blood Press*,* 25**,** 93-103 10.3109/08037051.2015.1116258.

Weber, M. A., Mansfield, T. A., Cain, V. A., Iqbal, N., Parikh, S., and Ptaszynska, A. (2016b) Blood pressure and glycaemic effects of dapagliflozin versus placebo in patients with type 2 diabetes on combination antihypertensive therapy: a randomised, double-blind, placebo-controlled, phase 3 study. Lancet Diabetes Endocrinol*,* 4**,** 211-220 10.1016/s2213-8587(15)00417-9.

Weinstock, R. S., Guerci, B., Umpierrez, G., Nauck, M. A., Skrivanek, Z., and Milicevic, Z. (2015) Safety and efficacy of once-weekly dulaglutide versus sitagliptin after 2 years in metformin-treated patients with type 2 diabetes (AWARD-5): a randomized, phase III study. Diabetes Obes Metab*,* 17**,** 849-58 10.1111/dom.12479.

Weissman, P. N., Carr, M. C., Ye, J., Cirkel, D. T., Stewart, M., Perry, C., and Pratley, R. (2014) HARMONY 4: randomised clinical trial comparing once-weekly albiglutide and insulin glargine in patients with type 2 diabetes inadequately controlled with metformin with or without sulfonylurea. Diabetologia*,* 57**,** 2475-2484 10.1007/s00125-014-3360-3.

White, W. B., Cannon, C. P., Heller, S. R., Nissen, S. E., Bergenstal, R. M., Bakris, G. L., Perez, A. T., Fleck, P. R., Mehta, C. R., Kupfer, S., Wilson, C., Cushman, W. C., and Zannad, F. (2013) Alogliptin after acute coronary syndrome in patients with type 2 diabetes. N Engl J Med*,* 369**,** 1327-35 10.1056/NEJMoa1305889.

Wilding, J. (2012) Long-Term Efficacy of Dapagliflozin in Patients With Type 2 Diabetes Mellitus Receiving High Doses of Insulin. Annals of Internal Medicine*,* 156**,** 405-415 10.7326/0003-4819-156-6-201203200-00003 %m 22431673.

Wilding, J. P., Charpentier, G., Hollander, P., González-Gálvez, G., Mathieu, C., Vercruysse, F., Usiskin, K., Law, G., Black, S., Canovatchel, W., and Meininger, G. (2013) Efficacy and safety of canagliflozin in patients with type 2 diabetes mellitus inadequately controlled with metformin and sulphonylurea: a randomised trial. Int J Clin Pract*,* 67**,** 1267-82 10.1111/ijcp.12322.

Wiviott, S. D., Raz, I., Bonaca, M. P., Mosenzon, O., Kato, E. T., Cahn, A., Silverman, M. G., Zelniker, T. A., Kuder, J. F., Murphy, S. A., Bhatt, D. L., Leiter, L. A., Mcguire, D. K., Wilding, J. P. H., Ruff, C. T., Gause-Nilsson, I. a. M., Fredriksson, M., Johansson, P. A., Langkilde, A. M., and Sabatine, M. S. (2019) Dapagliflozin and Cardiovascular Outcomes in Type 2 Diabetes. N Engl J Med*,* 380**,** 347-357 10.1056/NEJMoa1812389.

Wysham, C., Blevins, T., Arakaki, R., Colon, G., Garcia, P., Atisso, C., Kuhstoss, D., and Lakshmanan, M. (2014) Efficacy and Safety of Dulaglutide Added Onto Pioglitazone and Metformin Versus Exenatide in Type 2 Diabetes in a Randomized Controlled Trial (AWARD-1). Diabetes Care*,* 37**,** 2159-2167 10.2337/dc13-2760.

Yabe, D., Nakamura, J., Kaneto, H., Deenadayalan, S., Navarria, A., Gislum, M., and Inagaki, N. (2020) Safety and efficacy of oral semaglutide versus dulaglutide in Japanese patients with type 2 diabetes (PIONEER 10): an open-label, randomised, active-controlled, phase 3a trial. Lancet Diabetes Endocrinol*,* 8**,** 392-406 10.1016/s2213-8587(20)30074-7.

Yabe, D., Shiki, K., Homma, G., Meinicke, T., Ogura, Y., and Seino, Y. (2023) Efficacy and safety of the sodium-glucose co-transporter-2 inhibitor empagliflozin in elderly Japanese adults (≥65 years) with type 2 diabetes: A randomized, double-blind, placebo-controlled, 52-week clinical trial (EMPA-ELDERLY). Diabetes, Obesity and Metabolism*,* 25**,** 3538-3548 10.1111/dom.15249.

Yale, J. F., Bakris, G., Cariou, B., Nieto, J., David-Neto, E., Yue, D., Wajs, E., Figueroa, K., Jiang, J., Law, G., Usiskin, K., and Meininger, G. (2014) Efficacy and safety of canagliflozin over 52 weeks in patients with type 2 diabetes mellitus and chronic kidney disease. Diabetes, Obesity and Metabolism*,* 16**,** 1016-1027 10.1111/dom.12348.

Yang, W., Han, P., Min, K.-W., Wang, B., Mansfield, T., T'joen, C., Iqbal, N., Johnsson, E., and Ptaszynska, A. (2016) Efficacy and safety of dapagliflozin in Asian patients with type 2 diabetes after metformin failure: A randomized controlled trial. Journal of Diabetes*,* 8**,** 796-808 <https://doi.org/10.1111/1753-0407.12357>.

Yang, W., Pan, C. Y., Tou, C., Zhao, J., and Gause-Nilsson, I. (2011) Efficacy and safety of saxagliptin added to metformin in Asian people with type 2 diabetes mellitus: a randomized controlled trial. Diabetes Res Clin Pract*,* 94**,** 217-24 10.1016/j.diabres.2011.07.035.

Yoon, K. H., Steinberg, H., Teng, R., Golm, G. T., Lee, M., O'neill, E. A., Kaufman, K. D., and Goldstein, B. J. (2012) Efficacy and safety of initial combination therapy with sitagliptin and pioglitazone in patients with type 2 diabetes: a 54-week study. Diabetes, Obesity and Metabolism*,* 14**,** 745-752 <https://doi.org/10.1111/j.1463-1326.2012.01594.x>.

Yu, M., Brunt, K. V., Milicevic, Z., Varnado, O., and Boye, K. S. (2017) Patient-reported Outcomes in Patients with Type 2 Diabetes Treated with Dulaglutide Added to Titrated Insulin Glargine (AWARD-9). Clin Ther*,* 39**,** 2284-2295 10.1016/j.clinthera.2017.10.002.

Zinman, B., Wanner, C., Lachin, J. M., Fitchett, D., Bluhmki, E., Hantel, S., Mattheus, M., Devins, T., Johansen, O. E., Woerle, H. J., Broedl, U. C., and Inzucchi, S. E. (2015) Empagliflozin, cardiovascular outcomes, and mortality in type 2 diabetes. New England Journal of Medicine*,* 373**,** 2117-2128 10.1056/NEJMoa1504720.
